# Hemipiperazines as peptide-derived molecular photoswitches with low-nanomolar cytotoxicity

**DOI:** 10.1038/s41467-022-33750-7

**Published:** 2022-10-14

**Authors:** Susanne Kirchner, Anna-Lena Leistner, Peter Gödtel, Angelika Seliwjorstow, Sven Weber, Johannes Karcher, Martin Nieger, Zbigniew Pianowski

**Affiliations:** 1 Institute of Organic Chemistry, Karlsruhe Institute of Technology, 76131 Karlsruhe, Germany; 2 Department of Chemistry, University of Helsinki, FIN-00014 Helsinki, Finland; 3 Institute of Biological and Chemical Systems—FMS, Karlsruhe Institute of Technology, 76344 Eggenstein-Leopoldshafen, Germany

**Keywords:** Targeted therapies, Single-molecule fluorescence, Small molecules, Drug delivery

## Abstract

Molecular photoswitches transform light energy into reversible structural changes. Their combination with known pharmacophores often allows for photomodulation of the biological activity. The effort to apply such compounds in photopharmacology as light-activated pro-drugs is, however, hampered by serious activity reduction upon pharmacophore modifications, or limited biostability. Here we report that a potent antimitotic agent plinabulin and its derivatives demonstrate up to 56-fold reversible activity photomodulation. Alternatively, irreversible photoactivation with cyan light can enhance the cytotoxicity up to three orders of magnitude—all without compromising the original activity level, as the original pharmacophore structure is unchanged. This occurs due to the presence of a peptide-derived photoswitchable motif hemipiperazine inside the plinabulin scaffold. Furthermore, we systematically describe photochromism of these thermally stable and biocompatible hemipiperazines, as well as a photoswitchable fluorophore derived from plinabulin. The latter may further expand the applicability of hemipiperazine photochromism towards super-resolution microscopy.

## Abstract (web summary)

The development of photochromic systems is an important and growing area of research, in particular for bioactive molecular photoswitches. Here, the authors report on photopharmacological antimitotic agents, operational under visible light, based on a peptide-derived hemipiperazine photochrome.

## Introduction

Light is an ideal reagent, which can be applied with high precision without permanent contamination of the target. Molecular photoswitches^1^ can reversibly transform light energy into functions at the microscopic and macroscopic level, resulting in smart materials^2,3^, switchable magnets^4^, or molecular motors^5^. Light-triggered modulation of biological activity can be used to design photopharmacological agents^6^, which may elicit a therapeutic effect upon local photoactivation. This was demonstrated by incorporating photoswitches (mostly azobenzenes) into the structure of antibiotics^7^, dihydrofolate reductase (DHFR) inhibitors^8^, histone deacetylase (HDAC) inhibitors^9^, or antimitotic^10,11^ agents, including several combretastatin analogues^12–16^. The photochromic antimitotic agents were applied e.g., in studies of embryonic development^17,18^. However, the intended therapeutic use as anticancer photopharmacological agents is hampered by: (a) significant decrease in activity (often above 100-fold) caused by necessary modifications of the pharmacophore structure with the photochromic fragment, (b) low difference between the activity of photoisomers, (c) short lifetime of the active form, (d) activation with UV light that poorly penetrates the human body, or by a combination of these factors, in addition to the known metabolic instability of many azobenzenes^19^. Therefore, searching for alternative bioactive photochromic systems is of utmost importance for the progress in practical implementation of photopharmacology^13,16^.

**Fig. 1:**
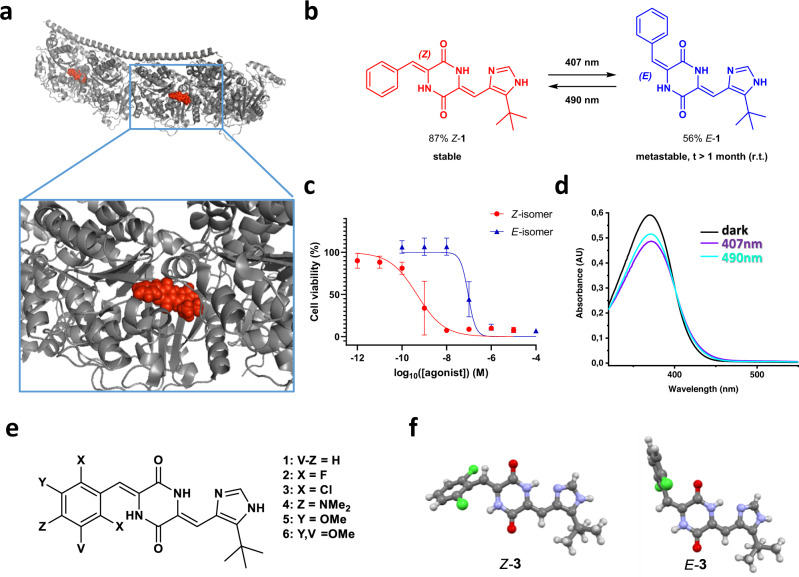
Plinabulin (1) and its derivatives as photopharmacological agents. **a** *Z*-**1** *(red)* binds β-tubulin in αβ-tubulin heterodimers *(grey)* (PDB:5c8y)^20^. **b** irradiation of the thermodynamically stable *Z-***1** (*red*) with violet light (<410 nm) produces up to 56% of the less active *E-***1** (*blue*), which can be isolated and stored at ambient conditions for many days (t_1/2_ > 1 month); irradiation of *E-***1** *(blue)* with cyan light (490 nm) regenerates up to 87% of the active *Z-***1**. **c** Viability of HT-29 cells after treatment with the HPLC-purified *Z*- and *E*-isomers of **1** for 48 h. Data were plotted against the log of agonist concentration (log_10_([agonist]) (M)) with mean and SD values in GraphPad Prism. Number of independent experiments (six technical replicates per test): *N* = 3. **d** UV-Vis spectra of **1** as a purified *Z-*isomer (dark), and at respective photostationary states (407 nm and 490 nm). **e** structures of the plinabulin derivatives **1-6** discussed in this report - if not defined otherwise, substituents **X**, **Y**, **V**, and **Z** are hydrogen atoms (H). **f** X-ray structures of the chlorinated *Z-***3** and *E-***3** illustrate the geometry difference upon isomerization, which in turn influences the biological activity.

Plinabulin (**1**) is a low-nanomolar inhibitor of tubulin polymerization^20^, currently in the third phase of clinical trials against non-small cell lung cancer (NSCLC) and chemotherapy-induced neutropenia (CIN)^21^. It binds in the vicinity of the colchicine binding domain of β-tubulin in αβ-tubulin heterodimers (Fig. 1a), at a site and with kinetics distinct from other tubulin binders^22^. Compound **1** consists of two arylidene residues attached to a cyclic dipeptide core (2,5-diketopiperazine, DKP). DKP itself is an important pharmacophore and a constitutive part of numerous biocompatible smart materials^23^, including photochromic hydrogels investigated in our group^24–26^. Due to structural similarity with the gelators, we have initially used **1** as a bioactive cargo for drug-release formulations^26^.

*E/Z*-photoisomerization of arylidene substituents adjacent to heterocyclic 5-membered-ring systems is known for indigoids^27^, hydantoins^28^, or isoindolinones^29^. However, to our best knowledge no systematic studies have been published yet on the photochromism of arylidene-substituted heterocyclic 6-membered-rings—such as that occurring in **1**—although a few scattered publications reported photosensitivity^30,31^ (sometimes referred as photolysis^32^) of such systems - mostly in natural products.

Here we show that **1** exhibits an unexpected type of photochromism. Without modification of the original highly active structure, this compound can be reversibly switched with visible light between two thermally stable compositions of photoisomers with distinctly different activity. In that sense, it successfully addresses all the aforementioned limitations. The isolated less active photoisomer of plinabulin or its derivatives can be treated as pro-drug due to its thermal stability, and activated with cyan light. Irreversible but therapeutically meaningful increase of cytotoxicity in such setups can reach three orders of magnitude. Further, the photoswitchable fragment embedded in plinabulin—called hemipiperazine (HPI) - is isolated, and its photochromism is systematically characterized. Ultimately, a photoswitchable fluorophore (locked plinabulin) is demonstrated when rotation inside the plinabulin structure is restricted with an additional bond.

## Results and Discussion

### Photochromism of plinabulin and its derivatives—activity photomodulation with visible light

Upon control experiments for light-induced release of **1** encapsulated inside DKP-based photochromic hydrogels, we observed its reversible photoswitching between two forms *Z-***1** and *E*-**1** (Fig. 1b, Supplementary Fig. 4 and Table 1). These forms were sufficiently stable to be isolated and characterized with NOESY NMR spectroscopy (Supplementary Figs. 91, 92). The switching process was identified as a light-triggered *Z/E-*isomerization of the arylidene group attached to the cyclic dipeptide core.

**Table 1:** Photostationary state (PSS) composition of the compounds 1-6

Compound	365 nm (% of *E*-Isomer)	407 nm (% of *E*-Isomer)	430 nm (% of *E*-Isomer)	450 nm (% of *E*-Isomer)	470 nm (% of *E*-Isomer)	490 nm (% of *E*-Isomer)	523 nm (% of *E*-Isomer)
**1**	62	56	42	38	19	13	–
**2**	59	64	62	61	58	56	–
**3**	48	44	41	39	34	29	–
**4**	61	55	46	19	17	12	10
**5**	63	54	42	24	19	16	–
**6**	63	52	39	31	17	11	–

Collected data from the ^1^H NMR (in DMSO-*d*_6_) determination of the *E/Z*-isomer ratio of the respective compounds (**1-6**) upon irradiation with the indicated wavelengths of light until equilibrium (PSS), based on individually collected data for each of the compounds depicted on the Supplementary Figs. 34–39.

As this molecular photoswitch is composed from a hemistilbene unit attached to 2,5-diketopiperazine, we abbreviated it as hemipiperazine (HPI). Interestingly, under investigated conditions the *Z*/*E-*photoisomerization of **1** occurs solely at the C=C bond on the benzylidene group, while the heterocyclic arylidene substituent of **1** remains inert to light. This is probably a result (as analysed below) of the relative geometric configuration of chromophores—known to affect photoisomerization efficiency in other multiphotochromic systems^33^.

**Fig. 2:**
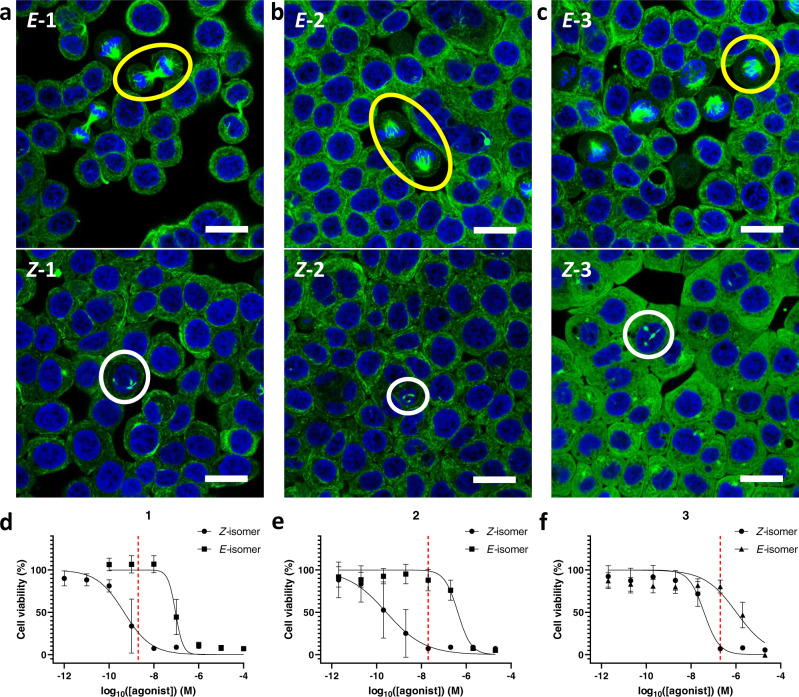
Immunofluorescence imaging. Immunofluorescence imaging of microtubule network structure in presence of the compounds **1**, **2**, and **3**. Confocal microscopy assessment of cellular microtubule networks after treatment with the respective photoisomers of **a** **1** (2 nM), **b** **2** (20 nM), and **c** **3** (200 nM). (HT-29 cells, 6 h treatment, green: α-tubulin stain for microtubule polymer network, blue: DAPI nuclear counterstain, all scale bars 20 µm). The results were processed and visualized with Leica Application Suite X 3.5.7.23225 from Leica Microsystems CMS GmbH. Correctly formed mitotic spindles are marked with yellow circles (the used concentrations of *E*-isomers are not cytotoxic) while white circles highlight abnormal tubulin aggregates (*Z*-isomers at the same concentrations showed cytotoxicity); N = 1. Viability of HT-29 cells after treatment with the HPLC-purified *Z*- and *E*-isomers of **1** (**d**), **2** (**e**) and **3** (**f**) for 48 h. Data were plotted against the log of agonist concentration (log_10_([agonist]) (M)) with mean and SD values in GraphPad Prism. Number of independent experiments (six technical replicates per test): **1** N = 3; **2-*Z*** N = 4; **2-*E*** N = 3; **3** N = 3. The dashed vertical line indicates the concentration used in the respective pair of immunofluorescence experiments depicted above the plot.

*Z-***1** is the thermodynamically stable form. However, the thermal decay of purified *E-***1** in darkness is so slow, that no detectable formation of the active *Z-***1** isomer in aqueous buffer at 37 °C occurs for a period of at least 32 hours (Supplementary Fig. 51e). Even at 150 °C the half-life of *E-***1** in solution exceeds 8 h (Supplementary Fig. 51a). In contrast to the aforementioned azobenzene-decorated pharmacophores^7,12^, our lead structure is not modified, therefore the active isomer does not lose its initial potency (Supplementary Table 1). Cell viability MTT assays against HT-29 human colon cancer cells (as in^34^) demonstrated that the respective IC_50_ values of the purified photoisomers differ by two orders of magnitude (IC_50_(*Z-***1**) = 0.47 nM, vs. IC_50_(*E-***1**) = 92 nM) (see Figs. 1c, 2d and Supplementary Table 1) due to light-induced geometry changes (illustrated on Fig. 1f). Hence, we consider the *E-*isomer to be a metastable prodrug, which can be isolated in vitro, stored in darkness for long periods of time (below 1% back-switching over 2 months in the freezer), and activated on demand (e.g. in vivo) with cyan light to a mixture containing maximally 87% of the active *Z-***1** form (IC_50_(PSS_490 nm_) = 1.08 nM, Supplementary Table 2). Thus, the efficient unidirectional 85-fold activity increase can be achieved with light, representing the practical therapeutic window for future medical applications (Supplementary Fig. 2).

The reversible switching of **1** can be also achieved solely with light. This can be of interest e.g. for precise control of in vivo photomodulation of microtubule dynamics. Alternating cyan (490 nm) and violet (410 nm) light results in a reversible 11-fold activity photomodulation (IC_50_(PSS_410 nm_) = 12.2 nM, 56% *E-***1**, Supplementary Table 2, Supplementary Fig. 2). In case of less complex biological setups, where application of UV light (365 nm) is tolerable, the activity can be reversibly photomodulated to even larger extent (IC_50_(PSS_365 nm_) = 44.9 nM, 62% *E-***1**), reaching 41-fold activity modulation by alternating 490 nm/365 nm irradiation (Supplementary Table 2, Supplementary Fig. 2)

The concept of photopharmacology was previously realized by modifications (addition, or replacement of a fragment of the original structure) of pharmacophores with known photochromic motifs—mostly azobenzenes, which provide large geometry and polarity photomodulation combined with well-understood pathways of tuning photophysical properties^6^. Light-induced isomerization of a known photochromic motif (stilbene) in an unmodified pharmacophore has been reported for combretastatin-4A for UV-light activation with mercury lamp^35^, or a two-photon approach^36^. In 2018, a 43-fold increase in activity upon irradiation with UV light (385 nm) of the heterostilbene fragment was reported for a VEGFR2 kinase inhibitor and an FDA-approved drug Axitinib. Its isomerization was, however, irreversible in aqueous condition due to the competing formation of an inactive dimer^37^. Our current observations reveal that plinabulin—a photopharmacological agent with ADME properties previously validated in human clinical trials - is addressable entirely within the visible range of light and in the reversible manner. Additionally, the reversible photomodulation of activity is realized with an unexpected photochromic motif—the hemipiperazine.

It is also worth mentioning, that the light frequencies applied to reversibly photoswitch between photoisomers of **1** correspond nicely with laser frequencies used in fluorescent confocal microscopy (405, 488, 561, and 642 nm). The violet and cyan channels could be used to switch between the photostationary states of **1** with majority of *E-* or *Z-*isomer, respectively. And their steep decay of absorption just below 500 nm (Fig. 1d and Supplementary Fig. 4) enables at the same time independent imaging of cellular processes or subcellular structures with the green and/or red channel, e.g. parallel YFP/RFP in vivo imaging (514/561 nm)^14,16^.

No degradation of **1** was noticed in presence of blue or cyan light (450–490 nm)—the range applicable for the potential in vivo activation. Upon irradiation at and below 430 nm (where the less bioactive *E-*isomer is predominantly generated), slight photodegradation of **1** has been observed in presence of oxygen in diluted samples (Supplementary Fig. 59). To explain the possible degradation pathway, we have performed an assay based on the bicyclic ozonide formation from 2,5-diphenylfuran (DPF)—a well-established literature method of colorimetric singlet oxygen detection^38^. No singlet oxygen generation has been observed upon prolonged irradiation (60 min.) of DPF and *Z*-**1** with 490 nm light (Supplementary Fig. 70a). Irradiation of DPF and *Z*-**1** with 410 nm light caused minimal degradation of the DPF probe (initially observed after 5 min of constant irradiation) that would be consistent with the singlet oxygen generation mechanism (Supplementary Fig. 70b). However, in comparison with efficient singlet oxygen generators (like methylene blue, Q.Y. = 50%, which degraded DPF within 1 s in our setup, Supplementary Fig. 70d), the rate of singlet oxygen production by irradiation of **1** with <430 nm light is negligibly small (Q.Y. < 0.1%), which would disqualify **1** as a potential photodynamic therapy agent. And considering the efficient intracellular systems that neutralize radical oxygen species, which are present at significantly higher concentrations (e.g. 0.5–10 mM glutathione) than the efficient concentrations of plinabulin, this degradation pathway will likely be insignificant for potential reversible in vivo applications.

To fully prevent the formation of singlet oxygen-derived degradation products, the precise *E/Z*-ratio values at PSS have been determined either in degassed solvents, or in presence of reducing agents—like ascorbic acid (Supplementary Fig. 60). Under reducing conditions, ten subsequent switching cycles (407/490 nm) have been performed (Supplementary Fig. 60b). In contrary to most azobenzene-based systems, **1** was also resistant (for at least 16 h) on a mixture of reduced glutathione (10 mM) with TCEP (5 mM) (Supplementary Fig. 69), which is an established model of intracellular reducing conditions.

Solubility in aqueous media is another important issue raised in the context of therapeutic applications of photoswitchable cytotoxic agents. **1** exhibits limited water solubility (7.5 ± 1.7 µmol/L, or 2.5 mg/L^26^) that, however, still exceeds the IC_50_ value of *Z-***1** by four orders of magnitude. Moreover, our group has earlier demonstrated^26^ a hydrogel composition with significantly increased loading capacity of **1** (>350 µmol/L, or 118 mg/L), which—upon formulation into injectable microgels—may increase the delivery efficiency of **1** even further.

Encouraged by these results, we have synthesized a short series (**2-6**, Fig. 1e) of plinabulin analogues with a substituted phenyl ring, in order to assess their photophysical properties and the scope of light-driven cytotoxicity photomodulation. Initially, we demonstrate an extreme example: the 2,6-difluoroplinabulin **2** is the only hemipiperazine system among all described in this article, which shows negligible *E/Z*-isomer ratio differences upon photoswitching (56-64% of *E*-**2** upon photoequilibration in the whole range 365-490 nm) (Table 1)—primarily due to the insignificant band separation between photoisomers. On the other hand, the thermal stability of *E-***2** is even higher than that of *E-***1** (Supplementary Fig. 51c). Due to the >1000-fold activity difference between its isolated thermally stable isomers (IC_50_(*Z-***2**) = 0.27 nM, vs. IC_50_(*E-***2**) = 420 nM) (Fig. 2e and Supplementary Table 1), the purified *E-***2** isomer can be decently, although irreversibly, photoactivated with cyan light (IC_50_ = 29 nM for the *E/Z*-mixture at the PSS_490nm_) (Supplementary Fig. 2 and Supplementary Table 2).

The 2,6-dichloroplinabulin **3** exhibited significantly lower cytotoxicity, but its highest thermal stability of all the examined plinabulin derivatives enabled independent structural X-ray characterization of both photoisomers, (Fig. 1f) which confirmed our initial NOESY analysis. Moreover, we have confirmed by immunostaining (Fig. 2) that the compounds *Z-***2** and *Z-***3** inhibit the microtubule dynamics in a similar way as **1**, above their respective IC_50_ concentrations. The top panels of Fig. 2a–c show HT-29 cells treated with *E-*isomers of **1-3** below their IC_50_ values—we can see correctly formed mitotic spindles (encircled yellow), which lead to the regular cell division process. If the same cell type is treated with *Z-*isomers of **1-3** at the identical concentrations (bottom panels of Fig. 2a–c)—now above the respective IC_50_ values – we do not observe correct spindle formation, but rather abnormal tubulin aggregation (examples encircled white) that is consistent with the mitotic arrest caused by active plinabulin derivatives. The concentration of compound used for the immunostaining in each pair of the isomers has been visualized with the red dashed line overlaid on the respective MTT plot (Fig. 2d-f).

**Fig. 3:**
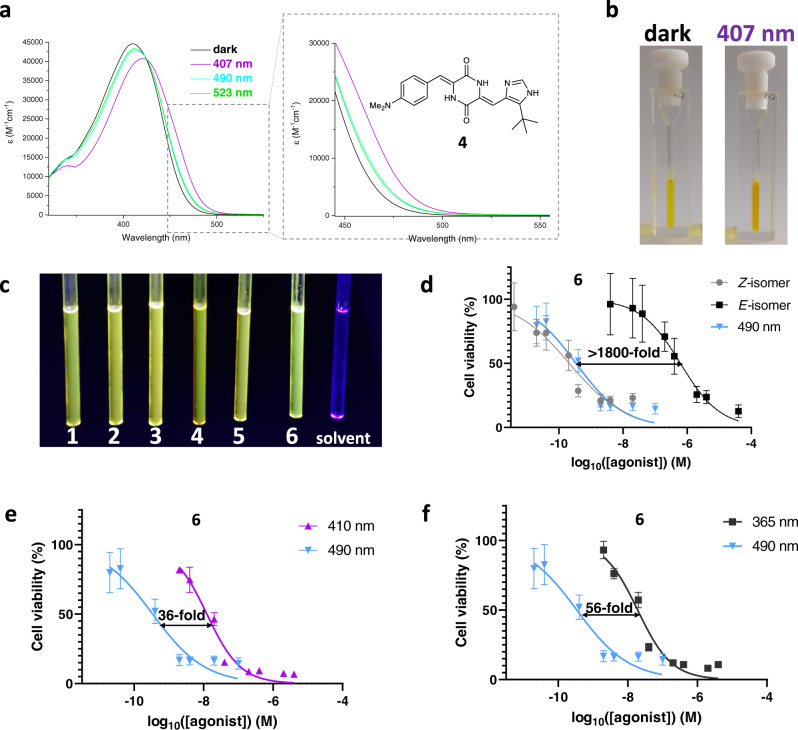
Plinabulin derivatives – photochromism, fluorescence and cytotoxicity photomodulation. **a** UV-Vis spectra of the 4-(*N,N-*dimethylamino)-plinabulin **4** as a purified *Z-*isomer (dark), and at respective photostationary states (407 nm, 490 nm, and 523 nm); **b** photochromism of **4** – the HPLC-purified dark sample containing >95% *Z-***4**, and the sample equilibrated at 407 nm (55% of the *E-***4**); **c** fluorescence of compounds **1-6** upon excitation with UV light (365 nm); 30-60 mM solutions in DMSO, compared with a solvent sample; **d** demonstration of a potential prodrug application of *E-***6** – the HPLC-isolated thermally metastable *E-***6** (IC_50_ = 618 nM) can be irreversibly converted with cyan light (490 nm) into a mixture containing 86% *Z-***6** with the IC_50_ = 0.34 nM (>1800-fold activity enhancement). Data were plotted against the log of agonist concentration (log_10_([agonist]) (M)) with mean and SD values in GraphPad Prism. Number of independent experiments (six technical replicates per test): **6-*Z*** N = 2, **6-*E*** N = 2, **6** 490 nm N = 1; **e** mutual photoequilibration with alternating cyan (490 nm) and violet (410 nm) light results in a reversible 36-fold difference in the bioactivity of **6**. Data were plotted against the log of agonist concentration (log_10_([agonist]) (M)) with mean and SD values in GraphPad Prism. Number of independent experiments (six technical replicates per test): N = 1; **f** mutual photoequilibration with alternating cyan (490 nm) and UV (365 nm) light results in a reversible 56-fold difference in the bioactivity of **6**. Data were plotted against the log of agonist concentration (log_10_([agonist]) (M)) with mean and SD values in GraphPad Prism. Number of independent experiments (six technical replicates per test): N = 1.

The 4-(*N,N-*dimethylamino)-plinabulin **4** undergoes an efficient *E*→*Z* isomerization with green light (523 nm), on which the derivatives **1-3** were inert (Fig. 3a and Supplementary Fig. 7, Table 1). Yet, the thermal lifetime of the *E-***4** is significantly shorter (Supplementary Fig. 51a) and the compound (both isomers) practically lost its activity (Supplementary Table 1 and Supplementary Fig. 1).

Finally, we have identified two derivatives—the 3-methoxyplinabulin **5** and the 3,5-dimethoxyplinabulin **6**—which were similar in potency to the *Z-***1**, but superior in the activity photomodulation (over three orders of magnitude difference between cytotoxicity of the respective *Z-* and *E-*isomers) (Supplementary Fig. 1 and Supplementary Table 1). Noteworthy, the isolated metastable *E-***6** (IC_50_ = 618 nM) upon irradiation with cyan light produced an equilibrium mixture (86% *Z-***6**) with the IC_50_ = 0.34 nM (Fig. 3d and Supplementary Table 2). This >1000-fold difference reflects the practical therapeutic window that may be achieved inside of a living organism using visible-light activation (e.g. targeting retina cancers through transparent eye tissues). In this mode of use, the *E-***6** profits from the one-time activity enhancement, analogous to irreversible photouncaging processes. However, we avoid typical disadvantages of caged pro-drugs, such as slow rates of post-illumination cleavage of cages, concomitant production of toxic by-products, or non-optical drug release mechanisms (e.g. enzymatic hydrolysis).

The reversible photoisomerization of **6** has been also demonstrated with alternating violet (410 nm) and cyan (490 nm) light. In that case, we observed the 36-fold activity difference between the respective equilibrium mixtures of photoisomers: IC_50_(PSS_410 nm_) = 12.4 nM, 52% *E-***6**, vs. IC_50_(PSS_490 nm_) = 0.34 nM, 14% *E-***6** (Fig. 3e and Supplementary Table 2). Eventually, alternation of UV (365 nm) and cyan (490 nm) light may provide >50-fold reversible activity photomodulation in UV-compatible systems (Fig. 3f and Supplementary Table 2).

At this point, we became intrigued by the apparent systematic discrepancy between the IC_50_ values (15–45 nM) reported in the literature^34^ for the purified *Z*-isomers of **1**, **5**, and **6**, and the results (0.09-0.47 nM) of our MTT assays (performed by us in darkness, using the same cell type). Therefore, we exposed our purified samples of the active *Z-*isomers to daylight for one day prior to performing MTT assays, which generated over 15% of the *E*-forms, and significantly reduced the samples’ activity (Supplementary Fig. 3 and Supplementary Table 3). After prolonged daylight exposure, the content of the less active *E*-isomers gradually reached 30–34% over three days. Thus, we think that the literature-reported values may have resulted from unintentional exposure of previously purified samples to daylight upon performance of their MTT assays. This situation may be analogous to the report from the Peifer group on Axitinib^37^, where a panel of 300 photochromic kinase inhibitors returned an almost identical selectivity profile for each *Z-* and *E-*isomer, as unintended exposure to ambient light caused a premature *E/Z* equilibration before execution of the assays.

Further analysis of the photophysical properties of **1-6** revealed minor solvatochromic effects (most pronounced for **4**) as well as significant acidochromism (Supplementary Figs. 22–28). The protonation also significantly decreased the lifetime of the *E-*isomers (Supplementary Fig. 51d) relative to neutral conditions. Finally, the compounds **1-6** exhibited moderate fluorescence emission (*Φ*_F_ between 2% and 6%, Fig. 3c and Supplementary Fig. 31) with slight bathochromic shift of the emission maximum (up to 21 nm) upon *Z-*to*-E* isomerization. All these preliminary observations open up exciting perspectives for systematic investigations of the photochromism in arylidene-substituted DKPs with various complexity, as exemplified below with a collection of simple carbocyclic hemipiperazines, as well as the locked plinabulin system.

### Hemipiperazine (HPI) as a molecular photoswitch and its photochromism

**Fig. 4:**
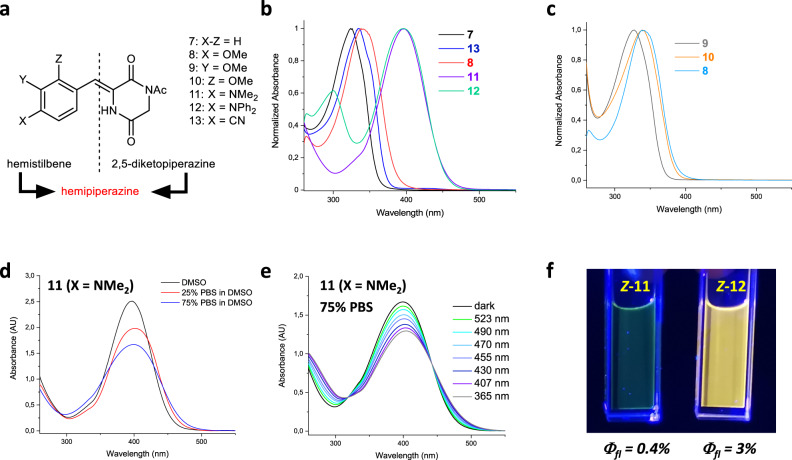
Photophysical characterization of hemipiperazines 7-13. **a** structures of hemipiperazines **7-13** (3-arylidene-2,5-diketopiperazines; hemistilbene + 2,5-diketopiperazine) investigated in this report; **b** UV-Vis spectra of selected HPIs (*Z-*isomers of **7**, **8**, **11-13**) in DMSO; **c** influence of the substitution pattern on the bathochromic shift of the λ_max_ for *Z-*isomers of **8-10** (in DMSO); **d** solvatochromism of **11** in aqueous solutions; **e** photochromism of **11** in aqueous solution containing 25% DMSO; **f** comparison of the fluorescence intensity between *Z-***11** and *Z-***12** in DMSO.

Inspired by the photoisomerization of plinabulin derivatives **1-6**, we decided to systematically explore the core motif responsible for their photochromism—the 3-arylidene-2,5-diketopiperazines, abbreviated as hemipiperazines (HPI). In order to do that, we synthesized a collection of simple HPI derivatives **7-13** (Fig. 4a) bearing a phenyl ring with or without substituents, devoid of the original imidazole pharmacophore of plinabulin. The compounds **7-13** have been isolated as thermally stable *Z-*isomers and their absorption spectra have been recorded. The most electron-rich substituents caused a distinct bathochromic shift of the *λ*_max_ (>70 nm for -NMe_2_ **11** and –NPh_2_ **12**) relative to the unsubstituted *Z-***7** (Fig. 4b). The conjugated *para-* and *ortho-*methoxy substituents (**8, 10**) also slightly red-shifted the *λ*_max_ while the non-conjugated *meta*-isomer **9** did not show any bathochromic shift (Fig. 4c).

All the compounds **7-13** demonstrated reversible photochromic behaviour (Supplementary Figs. 10–20 and  40–47) without significant fatigue upon 10 isomerization cycles (Supplementary Figs. 61–67). The compounds **7-10** and **13** were sensitive on UV (365 nm) and violet (407 nm) light, whereas compounds **11** and **12** efficiently isomerised within the range of 365–523 nm (and were slightly more prone to photooxidation if air oxygen is not eliminated by degassing or reducing agents). The photoconversion of unsubstituted **7** and the EWG-substituted **13** was low (<20% *E*), it slightly increased (>25% *E*) for the methoxy-substituted **8-10**. For the electron-rich **11**, the photoequilibria could reach up to 71% of *E-***11** with UV light, and as much as 97% *Z-***11** with green light (Supplementary Figs. 16, 45, Table 2). **11** was efficiently photoisomerized in aqueous solutions (like PBS – phosphate-buffered saline pH 7.4 – doped with 25% DMSO for sufficient solubility of **11**, Fig. 4e). While **12** demonstrated photochromism similar to **11** (Supplementary Figs. 19, 20), its fluorescence was significantly enhanced (Fig. 4f and Supplementary Fig. 32). Thermal stability of all the *E-*isomers of HPI was extremely high—after one week of incubation at 60 °C, decay of the shortest-living *E-***12** was below 13%, while the less electron-rich HPIs showed between 0.8% and 4% of the *E*→*Z* isomerization. With a considerable error margin, the decay can be extrapolated to half-lives spanning from one month to over one year, respectively (Supplementary Figs. 52–57 and Supplementary Table 6). Measurements at higher temperatures were inconclusive, due to emerging thermal decomposition. For the compound **12**, we have also determined photoisomerization quantum yields: *Φ*_*Z*→*E*_ = 16 ± 1 % (398 nm) and *Φ*_*E*→*Z*_ = 15 ± 1 % (520 nm) (values comparable to the *Φ*_*E*→*Z*_ of the π-π* transition determined for the azobenzene chromophore^39^) (Supplementary Fig. 50).

**Table 2:** Photostationary state (PSS) composition of the compounds 7-14

Compound	365 nm (% of *E*-Isomer)	407 nm (% of *E*-Isomer)	430 nm (% of *E*-Isomer)	450 nm (% of *E*-Isomer)	470 nm (% of *E*-Isomer)	490 nm (% of *E*-Isomer)	523 nm (% of *E*-Isomer)
**7**	19	6	–	–	–	–	–
7*	10	2	–	–	–	–	–
8*	29	3	<1	–	–	–	–
9*	31	6	–	–	–	–	–
10*	34	5	<1	–	–	–	–
**11**	71	62	53	20	13	7	3
**12**	58	54	47	26	19	15	14
13*	12	4	–	–	–	–	–
**14**	67	<5	–	–	–	–	–

Collected data from the ^1^H NMR determination of the *E/Z*-isomer ratio of the respective compounds (**7-14**) upon irradiation with the indicated wavelengths of light until equilibrium (PSS), based on individually collected data for each of the compounds, depicted on the Supplementary Figs. 40–48.

*in CD_2_Cl_2_, otherwise in DMSO-*d*_6_.

**Fig. 5:**
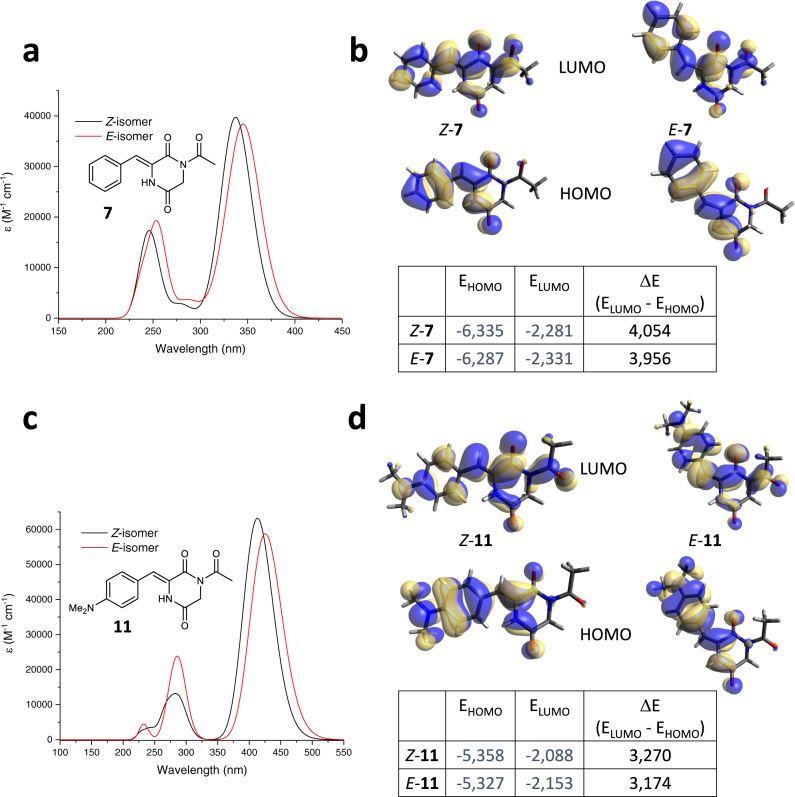
Theoretical calculations for selected HPI photoswitches. **a** Absorption spectra of the *Z-* and *E-*isomers of the unsubstituted HPI **7**. **b** selected orbital energies, as well as the electron distribution of HOMO and LUMO orbitals calculated for *Z-* and *E-*isomers of **7**. **c** Absorption spectra of the *Z-* and *E-*isomers of the electron-rich dimethylamino-substituted HPI **11**. **d** selected orbital energies, as well as the electron distribution of HOMO and LUMO orbitals calculated for *Z-* and *E-*isomers of **11**. All the results demonstrated on this figure have been calculated using B3LYP-GD3BJ/6-311G(d,p) PCM(DMSO) level of theory.

Next, we employed the B3LYP-GD3BJ/6-311 G(d,p) PCM(DMSO) level of theory, previously used for modelling of hemithioindigo photoswitches^40^, for theoretical calculations of the structures, absorption spectra, and orbital energies (including visualization of the electron distribution of the HOMO and LUMO orbitals) for the *Z-* and *E-*isomers of the HPIs **7-13** (Supplementary Information, section Theoretically obtained geometries and Supplementary Table 7). The calculated UV-Vis spectra (Supplementary Figs. 169–175) corroborated well with our experimental data. Selected calculation results for the unsubstituted HPI **7** and the dimethylamino-substituted HPI **11** have been collected at Fig. 5.

### Locked plinabulin—a hemipiperazine system with photomodulation of fluorescence

**Fig. 6:**
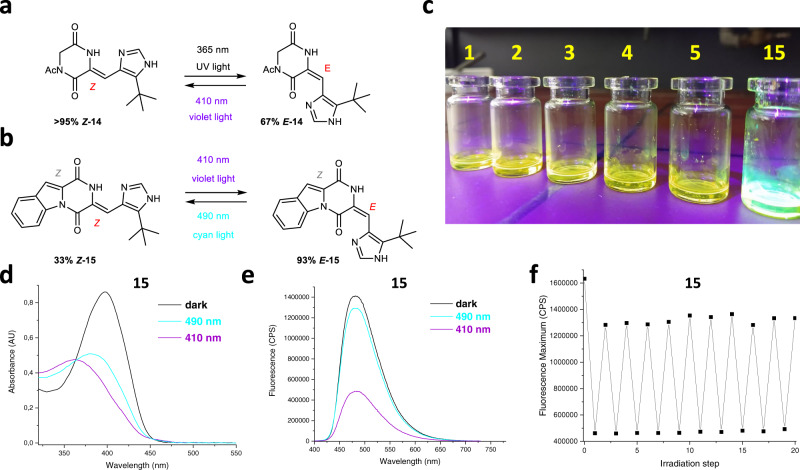
Further developments of photochromism in the plinabulin system. **a** The isolated heteroarylidene fragment **14** of the original plinabulin **1** structure can be mutually photoisomerized with UV and violet light, although the same chromophore remains inert towards photoisomerization within the complete structure of **1**. **b** Upon stabilization of the carbocyclic arylidene in the *Z-*configuration with an additional bond, the resulting locked plinabulin **15** shows photoisomerization of the heteroarylidene C=C bond. **c** The locked system **15** additionally shows hypsochromic shift and enhanced quantum yield of light emission in comparison to plinabulin derivatives **1-5**. **d** Photochromism of **15** upon mutual photoisomerization with visible light: 410/490 nm (200 µM of **15** in saturated solution of ascorbic acid in DMSO). **e** Photomodulation of the fluorescence intensity of **15** at the respective photostationary states achieved with 410 nm or with 490 nm (200 µM of **15** in saturated solution of ascorbic acid in DMSO). **f** 10 cycles of fluorescence photomodulation in **15** with visible light irradiation: 410/490 nm (200 µM of **15** in saturated solution of ascorbic acid in DMSO).

Encouraged by the biological activity photomodulation of plinabulin derivatives **1-6**, as well as the photochromism in carbocyclic arylidene-substituted DKPs **7-13**, we wanted to further elaborate on the initial observation that the photoisomerization of **1-6** occurs only at the carbocyclic arylidene C=C bond, but not on the heteroarylidene site (Fig. 1b). To clarify if the heteroarylidene group installed on a DKP ring is at all capable of *Z*→*E-*isomerization, we examined photochromism of the intermediate **14** (Fig. 6a), which is the synthetic precursor for plinabulin **1** and its derivatives **2-6**. We observed efficient (67% *E-***14**) photoisomerization with 365 nm, almost fully (>95% *Z-***14**) reverted with 407 nm irradiation (Table 2, Supplementary Fig. 48). Thermal lifetime of the isolated *E-***14** was slightly (c.a. 4-5-times) shorter than that of *E-***1** (Supplementary Fig. 51b). These experiments demonstrated that the heteroarylidene substituent present in plinabulin is, in principle, capable of photoisomerization—even if in the particular case of plinabulin derivatives **1-6** it was not observed. Such effects were previously reported for other multinary photochromic systems, and assigned to mutual orientation of both chromophores^33^.

Thus, we decided to lock the carbocyclic arylidene double bond in the *Z*-configuration by virtue of an additional single bond in its *ortho*-position. The resulting compound *Z****-*15** (Fig. 6b) demonstrated significantly increased fluorescence level (Q.Y. = 24%) and hypsochromic shift of emission in comparison to the unrestrained plinabulin derivatives **1-6** (Fig. 6c and Supplementary Fig. 33a). Upon stabilization with ascorbic acid, *Z−***15** can be photoisomerized without degradation with violet light (410 nm) to the mixture containing 93% of *E***-15**. Upon cyclization, **15** entirely lost its cytotoxic activity (IC_50_ > 10 µM in MTT assays against HT-29, for both pure *Z-***15**, as well as the irradiated mixture containing 93% of *E-***15**). Thermal stability of the *E-***15** at ambient temperatures was also very high (below 1% decay at 37 °C over 6 hours, Supplementary Fig. 58). Cyan light (490 nm) produces 33% of *Z-***15** (Fig. 6d, Supplementary Fig. 21 and Supplementary Fig. 49). Interestingly, the isomerization between respective photostationary states causes >3-fold change in the fluorescence intensity (Fig. 6e, Supplementary Fig. 33b), which is maintained upon 10 photoisomerization cycles (410/490 nm, Fig. 6f).

Hell, Betzig, and Moerner received in 2014 Nobel prize in chemistry for the development of super-resolution fluorescence microscopy, which can overcome the diffraction limit and enable accurate imaging of e.g. subcellular structures. Visualization of the structures is commonly realized with genetically encoded fluorophores (e.g. GFP), but small-molecule photoswitchable fluorophores recently emerged as alternative labels in this methodology. Therefore, we postulate that upon further optimization of the photophysical properties, the locked plinabulin **15** can become a prototype for a family of biocompatible photoswitchable fluorophores, that may complement the photoswitches currently applied in super-resolution microscopy (diarylethenes, spirooxazines)^41–44^.

To conclude, upon investigation of a potent antimitotic agent plinabulin **1** we discovered its unexpected photochromism, which was caused by an unknown molecular photoswitch—hemipiperazine (HPI)—embedded in its structure. The geometry changes, reversibly induced upon the photoswitching of **1** and its derivatives with visible light, are responsible for substantial activity differences between their isomers, as well as between their mixtures obtained at the respective photostationary states. High thermal stability of the less active isomers renders them pro-drugs suitable for purification, prolonged storage in darkness, and local in vivo activation resulting in over 1000-fold increase in potency. Importantly in this context, our observation makes plinabulin **1**, which is investigated in the third phase of human clinical trials, a photopharmacological agent reversibly triggered with visible light. It means that its ADME properties, as well as the potential influence on the human organism are already well-understood. Thus, we suggest that the direct therapeutic use of **1** may be based on irreversible photoactivation of the previously purified *E-*isomer. For example, an intravenous injection of the purified *E*-**1** (IC_50_ = 92 nM) as a pro-drug, and its local activation to 85% *Z-***1** (IC_50_ = 1.08 nM) with cyan light laser (488 nm) in spatial proximity of a retina cancer in an eye is a plausible proposal of an antitumor therapy (an 81-fold activity difference). Using the purified *E*-**6** (IC_50_ = 618 nM) as a pro-drug and its photoactivation to 86% *Z-***6** (IC_50_ = 0.34 nM) under the same conditions would provide even larger activity difference (>1800-fold, Fig. 3d), but would require additional clinical trials for this particular structural modification. Yet, the compound **6** may prove itself more useful than **1** for in vivo experiments that require reversible and strictly controlled modulation of microtubule dynamics with high spatiotemporal precision (e.g. in developmental biology). There, the substantial activity difference between two photostationary states (36-fold, Fig. 3e), which can be achieved with common laser emitters (407 nm, 488 nm) used as standard in fluorescent microscopy, would enable targeting with single-cell precision, and still leave the YFP (514 nm) and RFP (561 nm) channels available for imaging purpose.

Earlier photopharmacology reports were based on the approach where established photochromic motifs are either attached to, or replace a fragment of the structure in bioactive compounds. While it was extremely successful in various areas (antimicrobials, cytotoxic agents, photocontrol of ion channels), that concept assumed inevitable activity losses due to modification of the optimal pharmacophore. Here, we demonstrate the paradigm advance: the losses are not implied in our case. While discovery of photochromism in other unmodified drugs or bioactive natural products will remain a rare event, our publication should prompt at least re-examination of known cases, where such behavior might have been misinterpreted as photodegradation or photolysis.

The hemipiperazine (HPI) photochromic scaffold identified by us in plinabulin is based on the cyclic dipeptide motif (DKP), which occurs in numerous natural metabolites and is an important pharmacophore applied over a broad range of therapeutic indications^23^. The arylidene-DKP has been identified i.a. in antimicrobial albonoursin, cytotoxic phomazine B, or nocazines^23^. Thus, HPIs may in the future find broad applications in light-triggered activity modulation of bioactive or therapeutic agents—also targeting a broad range of non-cancer diseases—and complement the established photochromes (such as less polar azobenzenes or diarylethenes) currently used in such systems. To clarify the scope and limitations of HPI photochromism in this context, we have investigated its simplest representatives—the 3-arylidene-2,5-diketopiperazines with an illustrative range of substituents attached to the benzylidene group. Distinct photochromism upon mutual irradiation with UV and violet light, although with low photoconversions, has been observed for electron-poor and neutral systems. Yet, more electron-rich systems (compounds **11** and **12**) exhibited significant bathochromic shift and fair mutual photoconversions upon irradiation with visible light frequencies (green/violet), with quantum yields comparable to the established classes of photoswitches. The experimental absorption spectra nicely corroborated with the computational results, which opens the way for computational validation of photophysical properties in potentially bioactive systems prior to their synthesis.

Overall, our experiments and calculations demonstrated that the HPI motif follows the general trends reported for other *E/Z-*photoisomerizations observed in arylidene-substituted heterocyclic systems^27–29,40^— including the broad range of optimization in regards of its photoconversion, lifetime of the metastable isomer, or red-shifting of the λ_max_. Thus, we see an ample possibility of fine-tuning the photochromism for particular (e.g. in vivo) applications—for instance by introducing push-pull substitution patterns, or heteroarylidene residues instead of the carbocyclic systems demonstrated above. Here, particularly attractive seem to be modifications of the plinabulin scaffold oriented at further bathochromic shift of the activation frequency up to the therapeutic window range (>600 nm), which may enable treatment of solid tumours inside of a human body.

It has to be underlined, that HPIs show much higher level of biocompatibility in comparison to the established classes of photoswitches. In particular, HPIs remain operational in aqueous solutions (Fig. 4e). HPIs (and plinabulin) are also stable in presence of reducing agents, which is an important feature for intracellular applications in presence of millimolar physiological concentrations of glutathione—being an intrinsic limiting factor e.g., for azobenzene-based systems. Last, but not least, the cyclic dipeptide motif of HPI is prone to extensive hydrogen bonding—a feature that can be used for molecular recognition in photopharmacology, but also for the construction of supramolecular HPI-based light-triggered smart materials.

Finally, to indicate the applicability of HPI photochromism outside of photopharmacology, we have demonstrated—in the last section of our report— the locked plinabulin system, which exhibits reversible photomodulation of its fluorescence level upon photoisomerization with visible light. As photoswitchable fluorophores find increasing applications in super-resolution microscopy for imaging of biological structures, we envision HPI-based systems as being capable to complement the established photoswitches for such an application, too.

## Methods

### Data reporting

For activity determination of the purified thermally stable isomers (compounds 1-6) by viability assays, we used at least three independent series of experiments performed on different days and by more than one researcher. Activity of mixtures (e.g. various photostationary states) was determined using single or double series of experiments and additionally corroborated with the actual ratio of photoisomers (measured with HPLC) with previously determined activities. No statistical methods were used to predetermine sample size. The experiments were not randomized and the investigators were not blinded to allocation during experiments and outcome assessment.

### Compound synthesis and characterisation

All reagents and starting materials are commercially available. They were acquired from vendors: Sigma-Aldrich, Fluorochem, ChemPur, Alfa Aesar or Bepharm, and were used as supplied unless indicated otherwise. All experiments were performed under ambient atmosphere and in deionized water (Millipore) unless otherwise noted. All experiments containing compounds that can photoisomerize with visible light frequencies were performed under conditions minimizing exposition on daylight, like using glassware wrapped with aluminium foil, brown glassware, or working without sunlight in a room with dimmed lights. If air- and moisture-sensitive reagents were used, we performed the reactions under argon atmosphere using oven-dried glassware and common Schlenk-techniques. Liquids were added via steel cannulas and solids were added directly in powdered form. Column chromatography was performed using Silica gel 60 Å (40–63 μm particle size) (Sigma-Aldrich). Analytical thin layer chromatography was performed with silica coated aluminum plates (silica 60, F_254_, layer thickness: 0.25 mm) with fluorescence indicator by Merck. Detection proceeded under UV light at λ = 254 nm and λ = 365 nm. Preparative Reversed Phase High Performance Liquid Chromatography (RP-HPLC) was performed using the Puriflash™ 4125 system from Interchim, equipped with InterSoft® V5.1.08 software. A VDSpher® C_18_-M-SE precolumn (10 µm, 40 × 16 mm) followed by a VDSPher® C_18_-M-SE separation column (10 µm, 250 × 20 mm, VDS Optilab) was used as the stationary phase. We used a linear gradient of acetonitrile and double distilled water, both supplemented with 0.1% trifluoroacetic acid (TFA), at a flow rate of 15 mL/min as the mobile phase. The separation method (duration, gradient) was adapted to the respective compound. *NMR spectra* were recorded using the following devices: ^1^H NMR: Bruker Avance 400 (400 MHz), ^13^C NMR: Avance 400 (101 MHz), ^19^F NMR: Bruker AM 400 (377 MHz). The following solvents from Eurisotop were used: CDCl_3_, DMSO-*d*_6_. Chemical shifts *δ* were expressed in parts per million (ppm) and referenced to CDCl_3_ (^1^H: *δ* = 7.26 ppm, ^13^C: *δ* = 77.0 ppm), DMSO-*d*_6_ (^1^H: *δ* = 2.50 ppm, ^13^C: *δ* = 39.43 ppm)^45^. ^19^F NMR spectra were not referenced. All NMR spectra were processed using MestReNova v14.1.2 (Mestrelab Research S.L.). We recorded mass spectra using a Finnigan MAT 95 mass spectrometer using electron ionization-mass spectrometry (EI-MS) or fast atom bombardment-mass spectroscopy (FAB-MS). For FAB measurements, m-nitrobenzyl alcohol (3-NBA) was used as the matrix. Calibration was carried out using premixed calibration solutions (Thermo Fisher Scientific). The molecular fragments are stated as ratio of mass per charge m/z. IR spectra were recorded on a Bruker IFS 88 using attenuated total reflection (ATR). The intensities of the absolute peaks are given as follows: vs = very strong 0–9% T, s = strong 10–39% T, m=medium 40-69% T, w=weak 70-89% T, vw=very weak 90-100% T. All spectra were recorded at room temperature. Analytical High Performance Liquid Chromatography (HLPC) was performed using a 1200 Series from Agilent Technologies. The flow rate was 1.0 mL/min on an YMC C18-column JH08S04-2546WT with 250 mm length × 4.8 mm diameter and a column bead of 4 µm diameter. ChemStation for LC 3D systems (Agilent Technologies) was used for data extraction. Alternatively, Thermofisher UltiMate 3000 system containing a degaser, pump, autosampler, column compartment, and diode array detector was used. There, the flow rate was 1 mL/min on a stationary PerfectSil Target (MZ-Analytik) C18 column (3-5 µm, 4.0 mm × 250 mm). Chromeleon 7 software was used for data extraction. All chemical structures were drawn in Chemdraw Professional 20.1.0.110 (PerkinElmer Informatics, Inc.).

### Photocharacterisation

Sample irradiation for photoisomerization and measurements of photostationary states was performed using LEDs with emission maxima of 523 nm, 407 nm, and 365 nm from LED Engin, with 380 nm, 430 nm, 450 nm, and 490 nm from Avonec, and with 470 nm from OSRAM. For the time of irradiation, samples were maintained at constant temperature (22 ± 2 °C) using a metal cooling block. Irradiation intensities of the respective LEDs were determined using the PowerMax USB (type PS19Q) sensor device (Coherent®) in five independent measurements. The detector (diameter 19 mm) was located at a distance of 55 mm from the light source, identical to the position of irradiated samples. The results are presented in Supplementary Table 4. UV-Vis absorption spectra between 200 nm and 800 nm were recorded on a Lambda 750 (PerkinElmer) UV-Vis spectrophotometer and an UV/Vis/NIR spectrometer Cary 500 (Varian), referenced against pure solvent. The spectra were measured in Quartz cuvettes of 10 mm or 2 mm optical path length at 20 °C. Baseline correction was performed manually to correct for solvent composition. Fluorescence spectra were recorded on a Fluoromax-4 (Jobin Yvon-HORIBA) equipped with a Haake AC200 thermostat from Thermo Scientific at 20 °C. The spectra were recorded using FluorEssence v3.5. Fluorescence quantum yield (Φ_F_) measurements (absolute values) were performed on a Hamamatsu Quantaurus-QY absolute PL quantum yield spectrometer C11347. All samples were dissolved in MeCN (40 µM), transferred into 5.0 mL quartz cuvettes and excited by a xenon lamp with monochromator (selected wavelength at 400 nm with 10 nm bandwidth). The reaction quantum yields (Φ_Z→E_ and Φ_E→Z_) were determined in DMSO, at a concentration of 2.5 mM, using a setup previously published by the working group around Riedle (LMU)^46^. Irradiation occurred with 398 nm (Edison Opto, EDEV-SLC1-03) and 520 nm (OSRAM Opto Semiconductors Inc., LTCP7P-KXKZ) LEDs, respectively, at an irradiation intensity of 3.095 mW/cm^2^ at the sample for either wavelength of light. The reaction progress during irradiation was followed via HPLC.

### Cell cultures

HT-29 cells (Caucasian colon adenocarcinoma, ATCC HTB-38™, supplier: Sigma, product number 91072201-1VL) were grown in DMEM (Dulbecco’s Modified Eagle Medium) which was modified with 10% FCS (fetal calf serum) and 1% penicillin/streptomycin solution (10,000 units/mL of penicillin and 10,000 µg/mL of streptomycin) in a humid incubator at 37 °C with 5% CO_2_. Cells were washed with PBS (Phosphate-Buffered Saline) from Gibco®. Cells were detached from the surfaces with Trypsin-EDTA (0.25%) from Gibco®. The cells were from ATCC and tested negative for mycoplasma contamination.

### Viability assay

Method A: HT-29 cells were seeded in 96-well plates at 3.000 cells/well and incubated overnight to ensure cell attachment to the well bottom and cell growth. The tested compounds **1**-**6** were added in the dark and the cells were incubated for 48 h (final well volume 100 µL, 0.25% DMSO; six technical replicates). To ensure the same treatment to the control rows, the DMEM was removed from the wells and 0.25% DMSO in DMEM (100 µL) was added to the corresponding wells. The positive control was treated with 5 µL of Triton™ X-100 detergent (10% solution (w/v)) per well for at least 5 min to induce cell death before adding 15 µL of MTT dye-solution (3-(4,5-dimethylthiazol-2-yl)−2,5-diphenyltetrazoliumbromid in water / CellTiter 96®Non-Radioactive Cell Proliferation Assay from Promega or Invitrogen™ CyQUANT™ MTT Cell Viability Assay) to all sample wells and incubating for 3 h in the dark. 100 µL of Solubilization Solution/Stop Mix (CellTiter 96®Non-Radioactive Cell Proliferation Assay from Promega/Invitrogen™ CyQUANT™ MTT Cell Viability Assay) were added after incubation to stop the reduction of MTT to formazan, therefore prevent overreaction and solubilize the formazan crystals. Alternatively, a freshly prepared stop solution (10% sodium dodecyl sulfate, 0.01 M HCl in water) was used. After 24 h of solubilization in the incubator, the plate was read out with a plate reader (SpectraMax® iD3, Molecular Devices) by measuring the absorption of each well at 570 nm or 590 nm. Data points were only excluded from the analysis when errors were observed during execution of the experiment (e.g. pipetting to a wrong well). In the experiment where a sample of compound **6** was irradiated at 490 nm (Supplementary Fig. 1), a complete row of wells (one concentration) was excluded. Otherwise only single wells were excluded. Absorbance data was averaged over the technical replicates, the positive control subtracted as background, then normalized to viable cell count from negative control cells (% control) as 100% using Microsoft Excel 2019 Version 1808 (Microsoft Corporation). Three independent experiments were performed for each purified isomer. In the case of photoequilibrated mixtures, the number of replicates has been indicated at each respective experiment. Data were plotted against the log of agonist concentration (log_10_([agonist]) (M)) with mean and SD in GraphPad Prism Version 9.1.1 for Windows, GraphPad Software, San Diego, California USA, www.graphpad.com.

Method B: Following changes apply for viability assay with more detailed concentration range (Supplementary Fig. 1, compounds **5** and **6**): The tested photoisomers or irradiated mixtures were added in the dark and the cells were incubated for 48 h (final well volume 100 µL, *E*-isomer: 0.5% DMSO/*Z*-isomer and 490 nm PSS: 0% DMSO; three to six technical replicates). To ensure the same treatment to the control rows, the DMEM was removed from the wells and *E*-isomer: 0.5% DMSO/*Z*-isomer and 490 nm PSS: 0% DMSO in DMEM (100 µL) was added to the corresponding wells. The positive control was treated with 5 µL of Triton™ X-100 detergent (10% solution (w/v)) per well for at least 5 min before adding 10 µL of MTT dye-solution (3-(4,5-dimethylthiazol-2-yl)−2,5-diphenyltetrazoliumbromid in water / Cell Proliferation kit I (MTT) from Roche) to all sample wells and incubating for 4 h in the dark. 100 µL of Solubilization Solution/Stop Mix (Cell Proliferation kit I (MTT) from Roche) were added after incubation to stop the reduction of MTT to formazan, therefore prevent overreaction and solubilize the formazan crystals.

### Immunofluorescence imaging

HT-29 cells were seeded in 8 well µ-slides from Ibidi and incubated overnight. The medium was replaced by 200 µL test solution per well, diluted in DMEM medium containing 0.25% DMSO. Based on the results of the viability assays, a concentration between the two IC_50_-values of the respective photoisomers was chosen: plinabulin (**1**): 2 nM; compound **2**: 20 nM; compound **3**: 200 nM. After 6 h of incubation in the dark, the medium was removed, each well washed with 200 µL PBS and 200 µL/well of a 4% *para*-formaldehyde solution in PBS were added. After incubation for 10 min the fixing solution was removed, the cells were washed with PBS and 200 µL of a 1% Triton-X100 solution in PBS was added to each well and incubated for 4 min. Then the solution was removed, and the cells were washed again with PBS. To avoid false positive staining of the antibody, all binding sites were blocked by a CAS-Block™ histochemical reagent solution (ThermoFisher). For this purpose, PBS was removed and the CAS-Block™ solution was added (200 µL per well) and incubated for 30 min. For tubulin staining a fluorescein (FITC)-labeled monoclonal anti-α-tubulin antibody (3 mg/mL) produced in mice (Sigma; F2168-2ML) was used. 200 µL of a 1:500 dilution of the antibody in PBS was added and the cells were incubated overnight in the fridge. The next day, the solution was replaced by 1:5000 Hoechst 33342 (Promega) in PBS. After 5 min of incubation, the cells were washed with PBS and imaged using a TCS SPE microscope (Leica) microscope. The FITC-fluorescence was detected at 525 nm after irradiation at 470 nm, the Hoechst dye was irradiated at 361 nm and its emission was detected at 497 nm. The results were processed and visualized with Leica Application Suite X 3.5.7.23225 from Leica Microsystems CMS GmbH.

### Crystal structure determinations

The single-crystal X-ray diffraction study were carried out on a Bruker D8 Venture diffractometer with a PhotonII detector at 123(2) K, 173(2) K, or 298(2) K using Cu-K_α_ radiation (*γ* = 1.54178 Å). Dual space methods (SHELXT)^47^ were used for structure solution and refinement was carried out using SHELXL-2014 (full-matrix least-squares on *F*^*2*^)^48^. Hydrogen atoms were localized by difference electron density determination and refined using a riding model (H(N, O) free). Semi-empirical absorption corrections were applied. The absolute configuration of *Z*-**3** (CCDC 2076714) was determined by refinement of Parsons’ x-parameter^49^. For *Z*-**4** (CCDC 2076716) and *Z*-**6** (CCDC 2076718) an extinction correction was applied. In *Z*-**5** (CCDC 2076717), the 3-methoxybenzylidene moiety is disordered. Crystals of *Z***−7** (CCDC 2177720) and of *Z-***15** (CCDC 2177725) are refined as twins. For *Z-***7** (CCDC 2177720) the additional refinement as racemic twin failed and the absolute structure cannot be determined reliably (see Supplementary Data 2 for details). Due to the bad quality of the data, the structures of *Z-***7** (CCDC 2177720) and of *Z-***13** (CCDC 2177721) could be only used as a proof of the structure (see Supplementary Figs. 71-90, Supplementary Data 1 and Supplementary Data 2 - for details).

### Determination of isomerization quantum yields

The maximum reaction quantum yields Φ_max_ for either isomerization direction (*Z*-isomer to *E*-isomer Φ_*Z*→*E*_ and the reverse process Φ_*E*→*Z*_) were determined, using the experimental setup previously published by the working group around Riedle (LMU)^46^:

A 2.5 mM solution of **12** in DMSO was irradiated with 398 nm (*Edison Opto*, *EDEV-SLC1-03*) and 520 nm (*OSRAM Opto Semiconductors Inc*., *LTCP7P-KXKZ*) in succession. Several samples were taken from the solution during irradiation and the isomer-ratio was analyzed via HPLC. After reaching the PSS at 398 nm (55.9% *E*-isomer), the same solution was irradiated with 520 nm for a maximum of 120 min. The LEDs were driven at 4.0 V and 100 mA (398 nm) as well as 3.7 V and 59 mA (520 nm), to ensure the same irradiation intensity at the sample of 3.095 mW/cm^2^ for both wavelengths of light. The following formula, taken from^46^, was used to calculate Φ_max_:

1 $$\Phi=\frac{{N}_{{prod}}}{{N}_{{ph},{abs}}}={N}_{A}{hc}\frac{{c}_{{prod}}V}{{P}_{{abs}}\Delta t{\lambda }_{{LED}}}$$

with ${N}_{A}$ representing Avogrado’s constant, $h$ Planck’s constant, ${c}$ the speed of light, ${c}_{{prod}}$ the product concentration, $V$ the sample volume, ${P}_{{abs}}$ the absorbed radiant power, $\Delta t$ the irradiation time, ${\lambda }_{{LED}}$ the maximum emission wavelength of the LED used.

Since continuous irradiation well after the PSS is reached will eventually lead to a measured reaction quantum yield of Φ_t→∞_ = 0, only samples were taken into account, which still lie in the linear regime of the reaction progress. This ensures that Φ_max_ can be determined accurately, which more closely represents the switching efficiency of each individual molecule, rather than the efficiency of reaching the PSS of the whole mixture.

### Reporting summary

Further information on research design is available in the Nature Research Reporting Summary linked to this article.

## Supplementary information

Supplementary Information

Description of Additional Supplementary Files

Supplementary Data 1

Supplementary Data 2

Reporting Summary

## Data Availability

The data relating to the materials and methods, experimental procedures, NMR, MS and UV-Vis spectra, as well as calculations are available in the main text or in the Supplementary Information files, including the raw data from cell viability assays (MTT assays, Source Data) and crystallographic data (Supplementary Data 1 for plinabulin derivatives **2-6**, and Supplementary Data 2 for compounds **7**, **8**, **11**, **13**, **15**, and **19**), provided as the Supplementary Data files. The structure of plinabulin bound to beta-tubulin in alpha/beta-tubulin heterodimers is accessible in the Protein Data Bank (www.rcsb.org) under the accession code 5c8y Crystal structures have been deposited at the Cambridge Structural Database (https://www.ccdc.cam.ac.uk/structures/) under the accession codes: 2076713 (*Z*-**2**), 2076714 (*Z*-**3**), 2076715 (*E*-**3**), 2076716 (*Z*-**4**), 2076717 (*Z*-**5**), 2076718 (*Z*-**6**), 2177720 (*Z*-**7**), 2177723 (*Z*-**8**), 2177722 (*Z*-**11**), 2177721 (*Z*-**13**), 2177725 (*Z*-**15**), and 2177724 (**19**). CheckCIF files are available for the crystallographic structures reported with this article. Crystal structures generated during this study are also accessible in the Chemotion repository https://www.chemotion-repository.net under the following links: *Z*-**2** 10.14272/JORKNWYVOQPNQP-QOOFZUOPSA-N/CHMO0000156*Z*-**3** 10.14272/LMVUWTHIXFBXBY-UOUVAZQYSA-N.1*E*-**3** 10.14272/LMVUWTHIXFBXBY-GOBHWCIESA-N.2*Z*-**4** 10.14272/PIABYVZMMACTIP-APGQMXJTSA-N.1*Z*-**5** 10.14272/AIJQSTUPFONWLS-VULZFCBJSA-N.1*Z*-**6** 10.14272/OTVHUVBFCLOWCA-KPJFGDCZSA-N.1*Z*-**7** 10.14272/MJCOATFQVUUHFN-XFFZJAGNSA-N.1*Z*-**8** 10.14272/DHTWSXQNUMZDAD-GHXNOFRVSA-N.1*Z*-**11** 10.14272/SYFVNJGPALDWRA-JYRVWZFOSA-N.1*Z*-**13** 10.14272/DDZMJSZYMNIGEY-SDQBBNPISA-N.1*Z*-**15** 10.14272/HEBNVDMSJJEXNA-LCYFTJDESA-N/CHMO0000156**19** 10.14272/XEFPBGSACIWODS-UHFFFAOYSA-N.1 Data is available from the corresponding author upon request. Source data are provided with this paper.

## Source data

Source Data
