# Supplementary material for: Hemipiperazines as peptide-derived molecular photoswitches with low-nanomolar cytotoxicity
Source: Nat Commun. 2022 Oct 14;13:6066. doi: 10.1038/s41467-022-33750-7 (PMC9568564; doi:10.1038/s41467-022-33750-7)
Supplement: Supplementary file 1 — Supplementary Information [file 41467_2022_33750_MOESM1_ESM.pdf]

Supplementary Information

Hemipiperazines as peptide-derived molecular photoswitches with low-nanomolar cytotoxicity

Susanne Kirchner, Anna-Lena Leistner, Peter Gödtel, Angelika Seliwjorstow, Sven Weber, Johannes Karcher, Martin Nieger, Zbigniew Pianowski

Table of Contents

Supplementary Methods 2

- Synthetic Procedures and Characterization 2
  - Synthesis of (Z)-1-acetyl-3-((5-(tert-butyl)-1H-imidazol-4-yl)methylene)piperazine-2,5-dione (**14**) 2
  - Synthesis of Plinabulin-Derivatives 3
  - Synthesis of Hemipiperazines 10
  - Synthesis of “Locked” Plinabulin 16
- Cell viability assays (MTT) 20
- Irradiation Intensities of LEDs 25
- Absorption Spectra 26
  - Photostationary States: Plinabulin Derivatives 26
  - Photostationary States: Hemipiperazines in CH_2Cl_2 30
  - Photostationary States: Hemipiperazines in DMSO and PBS 33
  - Photostationary States: “Locked” Plinabulin 36
  - Solvatochromism and Acidochromism 37
- Fluorescence Spectra 44
- Determination of PSS Composition *via* NMR Spectroscopy 46
  - Plinabulin Derivatives 47
  - Hemipiperazines 51
- Determination of PSS Composition *via* Analytical HPLC 57
- Determination of Isomerization Quantum Yields 58
- Thermal Relaxation 59
  - Plinabulin Derivatives and Synthetic Intermediates **7** and **14** 59
  - Hemipiperazines 60
  - “Locked” Plinabulin 63
- Switching Stability: Plinabulin 64
- Switching Stability: Hemipiperazines 67
- Switching Stability: “Locked” Plinabulin 70
- Stability in Presence of Glutathione 71
- Singlet oxygen emission from irradiated samples of plinabulin 1 71
- Crystal Structure Determinations 73
- NMR spectra 89
- Theoretically Obtained Geometries 129
- Excitation Energies and Calculated Absorption Spectra 156

Supplementary References 175

Supplementary Methods

Synthetic Procedures and Characterization

*Synthesis of (Z)-1-acetyl-3-((5-(tert-butyl)-1H-imidazol-4-yl)methylene)piperazine-2,5-dione (**14**)*

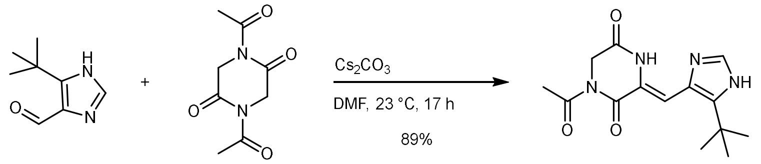

(5-(*tert*-Butyl)-1*H*-imidazol-4-carbaldehyde (1.50 g, 9.86 mmol, 1.00 equiv) was dissolved in dry DMF (17.0 mL) under an argon atmosphere. 1,4-Diacetyl-piperazine-2,5-dione (3.91 g, 19.7 mmol, 2.00 equiv) was dissolved in the mixture and Cs_2CO_3 (4.82 g, 14.8 mmol, 1.50 equiv) was added. Subsequently, the mixture was stirred for 17 hours under argon atmosphere at room temperature. The reaction mixture was subsequently poured on an ice water/brine mix (30 mL) and the precipitate was filtered off and dried to yield 2.54 g (8.73 mmol, 89%) of (*Z*)-1-acetyl-3-((5-(*tert*-butyl)-1*H*-imidazol-4-yl)methylene)piperazine-2,5-dione (**14**) as a beige solid.

^{1}H NMR (400 MHz, $CDCl_3$): δ = 12.36 (s, 1H, N*H*, imidazole), 12.01 (s, 1H, CON*H*), 7.85 (s, 1H, C*H*), 7.04 (s, 1H, C*H*), 4.30 (s, 2H, CH_2), 2.49 (s, 3H, CH_3), 1.39 (s, 9H, $C(CH_3)_3$) ppm.

^{13}C NMR (101 MHz, $CDCl_3$): δ = 172.2 (1C, *C*OCH_3), 162.2 (1C, *C*ONH), 160.1 (1C, *C*ON-COCH_3), 141.0 (1C, *C*-$C(CH_3)_3$), 134.5 (1C, *C*H, imidazole), 130.6 (1C, NH-*C*-CH), 123.7 (1C, *C*-CH, imidazole), 107.3 (1C, *C*H, double bond), 46.3 (1C, CH_2), 31.9 (1C, $C(CH_3)_3$), 30.6 (3C, $C(CH_3)_3$), 27.0 (1C, COCH_3) ppm.

IR (ATR): *v* = 3145 (vw), 3080 (w), 3023 (w), 3010 (w), 2978 (w), 2961 (w), 2901 (w), 2877 (w), 2850 (w), 2791 (w), 2765 (w), 2727 (w), 2684 (vw), 1706 (m), 1649 (m), 1612 (w), 1567 (w), 1509 (w), 1485 (w), 1436 (m), 1417 (w), 1397 (w), 1373 (w), 1364 (w), 1346 (w), 1258 (w), 1225 (m), 1200 (m), 1186 (m), 1162 (w), 1105 (w), 1048 (w), 1030 (w), 999 (w), 980 (w), 943 (w), 897 (vw), 871 (w), 829 (w), 778 (w), 741 (w), 730 (w), 688 (w), 664 (w), 586 (w), 578 (w), 533 (vw), 518 (w), 475 (w), 439 (w) cm^{-1}.

HRMS (FAB): *m*/*z* calcd. for $C_{14}H_{19}N_4O_3$ [M+H], 291.1457; found, 291.1458.

UV/Vis (MeCN): λ_{max} = 343 nm.

Synthesis of Plinabulin-Derivatives

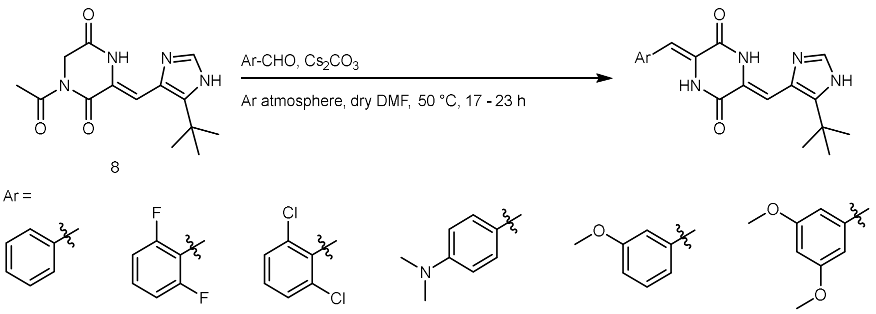

1: 65% 2: 45% 3: 58% 4: 23% 5: 49% 6: 40%

(*Z*)-1-Acetyl-3-((5-(*tert*-butyl)-1*H*-imidazol-4-yl)methylene)piperazine-2,5-dione (**14**) (100 mg, 344 µmol, 1.00 equiv) was dissolved in 1.0 mL of dry DMF under an argon atmosphere. The respective aldehyde (517 µmol, 1.50 equiv) was dissolved in the mixture and Cs_2CO_3 (168 mg, 517 µmol, 1.50 equiv) was added. The mixture was stirred for 17-23 h under an argon atmosphere at 50 °C. The reaction mixture was subsequently poured on ice water (20 mL) and the precipitate was filtered off. The crude product was purified *via* HPLC (gradient of 20-80% MeCN in H_2O within 40 min, 0.1% TFA (v/v) in the solvents) and washed with sat. aq. solution of $NaHCO_3$.

The respective *E*-isomers were isolated by irradiating a solution of the respective compound in DMSO with 407 nm for 1 hour. Subsequently, the two photoisomers were separated *via* HPLC and washed with sat. aq. solution of $NaHCO_3$.

*Plinabulin (**1**)*

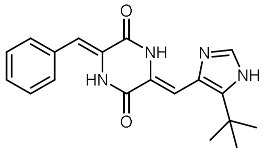

***Z*-isomer**

^{1}H NMR (400 MHz, DMSO-*d*$_6$): δ = 12.11 (bs, 1H, N*H*), 11.33 (bs, 2H, N*H*), 7.79 (s, 1H, C*H*), 7.64 (d, *J* = 7.6 Hz, 2H, H_{arom}), 7.38 (t, *J* = 7.6 Hz, 2H, H_{arom}), 7.26 (t, *J* = 7.4 Hz, 1H, H_{arom}), 6.80 (s, 1H, C*H*, double bond), 6.65 (s, 1H, C*H*, double bond), 1.38 (s, 9H, $C(CH_3)_3$) ppm.

^{13}C NMR (101 MHz, DMSO-*d*$_6$): δ = 158.0 (1C, *C*ONH), 156.7 (1C, *C*ONH), 140.2 (1C, *C*-$C(CH_3)_3$), 134.3 (1C, *C*H, imidazole), 133.8 (1C, C_{arom}-CH), 130.9 (1C, *C*-CH), 129.3 (2C, C_{arom}), 128.6 (2C, C_{arom}), 128.0 (1C, C_{arom}), 127.6 (1C, *C*-CH), 124.2 (1C, *C*-CH), 113.3 (1C, *C*H, double bond), 104.5 (1C, *C*H, double bond), 31.9 (1C, $C(CH_3)_3$), 30.7 (3C, $C(CH_3)_3$) ppm.

TLC: R_f= 0.22 (developed in 7.5% MeOH in CH_2Cl_2:toluene; 1:1).

HRMS (FAB): *m*/*z* calcd. for $C_{19}H_{20}N_4O_2$: 337.1665 [M]; found: 337.1665.

IR (ATR): *v* = 2977 (vw), 1684 (w), 1644 (w), 1400 (w), 1353 (w), 1186 (w), 1144 (w), 953 (vw), 801 (w), 765 (w), 719 (w), 689 (w), 645 (vw), 520 (vw), 459 (vw), 442 (vw) cm^{-1}.

UV/Vis (MeCN): λ_{max} = 362 nm.

Fluorescence (MeCN): λ_{em}= 512 nm (excited with λ = 370 nm).

Φ_F (MeCN): 0.045.

***E*-isomer**

^{1}H NMR (400 MHz, DMSO-*d*$_6$): δ = 12.26 (bs, 1H, N*H*), 12.07 (s, 1H, N*H*), 10.75 (bs, 1H, N*H*), 7.82 (s, 1H, C*H*), 7.54 (dd, J = 7.7, 1.6 Hz, 2H, H_{arom}), 7.28 (t, J = 7.3 Hz, 2H, H_{arom}), 7.22 (t, J = 7.3 Hz, 1H, H_{arom}), 6.79 (s, 1H, C*H*, double bond), 6.51 (s, 1H, C*H*, double bond), 1.38 (s, 9H, $C(CH_3)_3$) ppm.

^{13}C NMR (101 MHz, DMSO-*d*$_6$): δ = 157.9 (1C, *C*ONH), 155.9 (1C, *C*ONH), 140.6 (1C, *C*-$C(CH_3)_3$), 134.8 (1C, *C*H, imidazole), 131.2 (1C, C_{arom}-CH), 130.8 (2C, C_{arom}), 127.9 (2C, C_{arom}), 127.8 (1C, C_{arom}), 127.7 (1C, *C*-CH), 124.2 (1C, *C*-CH), 120.2 (1C, *C*H, double bond), 104.5 (1C, *C*H, double bond), 32.4(1C, $C(CH_3)_3$), 31.1 (3C, $C(CH_3)_3$) ppm.

UV/Vis (MeCN): λ_{max} = 360 nm.

Fluorescence (MeCN): λ_{em}= 533 nm (excited with λ = 400 nm).

Φ_F (MeCN): 0.020.

*(Z)-3-((5-(tert-butyl)-1H-imidazol-4-yl)methylene)-6-((Z)-2,6-difluorobenzylidene)piperazine-2,5-dione (**2**)*

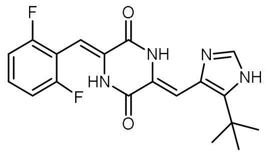

***Z*-isomer**

^{1}H NMR (400 MHz, DMSO-*d*$_6$): δ = 12.36 (s, 2H, N*H*), 10.42 (s, 1H, N*H*), 7.87 (s, 1H, C*H*), 7.43 (tt, *J* = 8.3, 6.5 Hz, 1H, H_{arom}), 7.13 (t, *J* = 8.2 Hz, 2H, H_{arom}), 6.90 (s, 1H, C*H*, double bond), 6.49 (s, 1H, C*H*, double bond), 1.39 (s, 9H, $C(CH_3)_3$) ppm.

^{13}C NMR (101 MHz, DMSO-*d*$_6$): δ = 161.4 (d, *J* = 8.2 Hz, 1C, C_{arom}-F), 158.9 (d, *J* = 7.7 Hz, 1C, C_{arom}-F), 157.3 (1C, *C*ONH), 155.4 (1C, *C*ONH), 140.7 (1C, *C*-$C(CH_3)_3$), 134.5 (1C, *C*H, imidazole), 130.5 (d, *J* = 15.7 Hz, 1C, C_{arom}), 123.5 (2C, *C*-CH), 111.7 (d, *J* = 24.3 Hz, 2C, C_{arom}), 110.6 (d, *J* = 19.9, 1C, C_{arom}-CH), 105.8 (1C, *C*H, double bond), 99.7 (1C, *C*H, double bond), 31.9 (1C, $C(CH_3)_3$), 30.6 (3C, $C(CH_3)_3$) ppm.

^{19}F NMR (376 MHz, DMSO-*d*$_6$): δ = -108.5 ppm.

IR (ATR): v = 3109 (w), 3050 (w), 2973 (w), 2876 (w), 1693 (vs), 1647 (vs), 1601 (m), 1469 (s), 1402 (vs), 1349 (m), 1273 (w), 1238 (w), 1204 (s), 1184 (vs), 1142 (vs), 1001 (vs), 952 (w), 904 (w), 837 (m), 813 (s), 803 (s), 783 (vs), 768 (vs), 717 (vs), 697 (w), 662 (w), 643 (w), 581 (w), 517 (w), 486 (m), 460 (s), 442 (m), 411 (w) cm^{-1}.

HRMS (FAB): *m/z* calcd. for $C_{19}H_{18}O_2N_4F_2$: 373.1476 [M+H]; found: 373.1477.

UV/Vis (MeCN): λ_{max} = 362 nm.

Fluorescence (MeCN): λ_{em}=525 nm (excited with λ = 370 nm).

Φ_F (MeCN): 0.055.

***E*-isomer**

^{1}H NMR (400 MHz, DMSO-*d*$_6$): δ = 12.34 (bs, 1H, N*H*), 12.11 (s, 1H, N*H*), 11.05 (s, 1H, N*H*), 7.83 (s, 1H, C*H*), 7.33 (tt, J = 8.3, 6.6 Hz, 1H, H_{arom}), 7.02 (t, J = 7.8 Hz, 2H, H_{arom}), 6.83 (s, 1H, *C*H, double bond), 6.13 (s, 1H, *C*H, double bond), 1.38 (s, 9H, C(C*H*$_3$)$_3$) ppm.

^{13}C NMR (101 MHz, DMSO-*d*$_6$): δ = 161.1 (d, *J* = 7.0 Hz, 1C, C_{arom}-F), 158.6 (d, *J* = 7.2 Hz, 1C, C_{arom}-F), 157.2 (1C, *C*ONH), 154.6 (1C, *C*ONH), 140.4 (1C, *C*-C(CH$_3$)$_3$), 134.4 (1C, *C*H, imidazole), 131.1 (1C, *C*-CH), 130.5 (1C, m, C_{arom}-CH), 129.1 (t, *J* = 10.8 Hz, 1C, C_{arom}-H), 123.5 (1C, *C*-CH), 113.30 – 112.67 (1C, C_{arom}-CH), 110.9 (d, *J* = 25.5 Hz, 2C, C_{arom}-H), 105.0 (1C, *C*H, double bond), 102.3 (1C, *C*H, double bond), 31.9 (1C, *C*(CH$_3$)$_3$), 30.6 (3C, C(*C*H$_3$)$_3$) ppm.

^{19}F NMR (376 MHz, DMSO-*d*$_6$): δ = -110.0 ppm.

UV/Vis (MeCN): λ_{max} = 363 nm.

Fluorescence (MeCN): λ_{em}= 532 nm (excited with λ = 400 nm).

Φ_F (MeCN): 0.025.

*(Z)-3-((5-(tert-butyl)-1H-imidazol-4-yl)methylene)-6-((Z)-2,6-dichlorobenzylidene)piperazine-2,5-dione (**3**)*

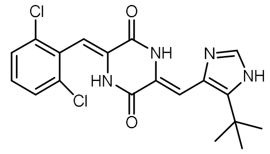

***Z*-isomer**

^{1}H NMR (400 MHz, DMSO-*d*$_6$): δ = 12.37 (s, 2H, N*H*), 10.42 (s, 1H, N*H*), 7.87 (s, 1H, C*H*, imidazole), 7.50 (d, *J* = 8.1 Hz, 2H, H_{arom}), 7.37 (t, *J* = 8.1 Hz, 1H, H_{arom}), 6.89 (s, 1H, C*H*, double bond), 6.55 (s, 1H, C*H*, double bond), 1.38 (s, 9H, C(C*H*$_3$)$_3$) ppm.

^{13}C NMR (101 MHz, DMSO-*d*$_6$): δ = 157.8 (1C, *C*ONH), 155.8 (1C, *C*ONH), 141.2 (1C, *C*-C(CH$_3$)$_3$), 135.0 (1C, *C*H, imidazole), 134.9 (1C, *C*-CH), 131.6 (1C, *C*-CH, imidazole), 131.1

(1C, C_{arom}-H), 130.7 (1C, C_{arom}-CH), 128.7 (2C, C_{arom}-H), 124.0 (1C, *C*-CH), 108.6 (1C, *C*H, double bond), 106.4 (1C, *C*H, double bond), 32.4 (1C, *C*(CH_3)$_3$), 31.1 (3C, C(*C*H_3)$_3$) ppm.

IR (ATR): *v* = 3111 (w), 3046 (w), 2978 (w), 2911 (w), 2873 (w), 1691 (s), 1645 (vs), 1599 (m), 1555 (w), 1490 (w), 1473 (w), 1405 (vs), 1347 (w), 1205 (s), 1183 (vs), 1143 (vs), 1096 (w), 953 (w), 904 (w), 817 (s), 802 (vs), 778 (vs), 765 (vs), 717 (vs), 700 (w), 656 (w), 642 (w), 601 (w), 558 (w), 537 (w), 518 (w), 469 (w), 446 (m), 426 (w), 402 (w) cm^{-1}.

HRMS (FAB): *m/z* calcd. for $C_{19}H_{18}Cl_2N_4O_2$: 405.0885 [M+H]; found: 405.0886.

UV/Vis (MeCN): λ_{max} = 358 nm.

Fluorescence (MeCN): λ_{em}= 527 nm (excited with λ = 400 nm).

Φ_F (MeCN): 0.056.

E-isomer

^{1}H NMR (400 MHz, DMSO-*d*$_6$): δ = 12.32 (bs, 1H, N*H*), 12.10 (s, 1H, N*H*), 11.05 (s, 1H, N*H*), 7.82 (s, 1H, C*H*, imidazole), 7.42 (d, J = 8.0 Hz, 2H, H_{arom}), 7.27 (t, J = 8.1 Hz, 1H, H_{arom}), 6.84 (s, 1H, C*H*, double bond), 6.23 (s, 1H, C*H*, double bond), 1.37 (s, 9H, C(CH_3)$_3$ ppm.

^{13}C NMR (126 MHz, DMSO-*d*$_6$): δ = 157.2 (1C, *C*ONH), 154.6 (1C, *C*ONH), 140.5 (1C, *C*-C(CH_3)$_3$), 134.4 (1C, *C*-CH), 134.0 (1C, *C*-CH, imidazole), 133.9 (1C, *C*H, imidazole), 130.5 (1C, *C*-CH), 129.0 (1C, C_{arom}-H), 127.5 (2C, C_{arom}-H), 123.4 (1C, *C*-CH), 111.0 (1C, *C*H, double bond), 105.1 (1C, *C*H, double bond), 31.9 (1C, *C*(CH_3)$_3$), 30.6 (3C, C(*C*H_3)$_3$) ppm.

UV/Vis (MeCN): λ_{max} = 356 nm.

Fluorescence (MeCN): λ_{em}= 534 nm (excited with λ = 400 nm).

Φ_F (MeCN): 0.027.

*(Z)-3-((5-(tert-butyl)-1H-imidazol-4-yl)methylene)-6-((Z)-4-(dimethylamino)benzylidene)-piperazine-2,5-dione (**4**)*

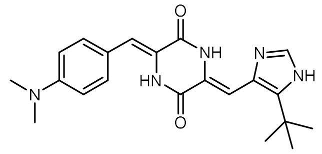

Z-isomer

^{1}H NMR (400 MHz, DMSO-*d*$_6$, ppm) δ = 12.29 (bs, 1H, N*H*), 12.11 (s, 1H, N*H*), 9.73 (s, 1H, N*H*), 7.83 (s, 1H, C*H*, imidazole), 7.42 (d, *J* = 9.0 Hz, 2H, H_{arom}), 6.82 (s, 1H, C*H*, double bond), 6.75 (d, *J* = 9.0 Hz, 2H, H_{arom}), 6.69 (s, 1H, C*H*, double bond), 2.96 (s, 6H, N-(CH_3)$_2$), 1.38 (s, 9H, C(CH_3)$_3$) ppm.

^{13}C NMR (101 MHz, DMSO-*d*$_6$, ppm) δ = 157.4 (1C, *C*ONH), 156.9 (1C, *C*ONH), 150.0 (1C, C_{arom}-N(CH_3)$_2$), 139.9 (1C, *C*-C(CH_3)$_3$), 134.2 (1C, *C*H, imidazole), 130.8 (1C, C_{arom}-CH), 130.7

(2C, C_{arom}-H), 124.1 (1C, *C*-CH), 122.8 (1C, *C*-CH), 120.5 (1C, C_{arom}-CH), 115.6 (1C, *C*H, double bond), 112.0 (2C, C_{arom}-H), 104.2 (1C, *C*H, double bond), 39.4 (2C, N(CH_3)$_2$), 31.9 (1C, *C*(CH_3)$_3$), 30.6 (3C, C(CH_3)$_3$) ppm.

IR (ATR): *v* = 3429 (w), 3391 (w), 3183 (m), 3119 (w), 3085 (w), 3055 (w), 3048 (w), 3012 (w), 2953 (m), 2921 (m), 2851 (m), 2802 (w), 2727 (w), 2681 (w), 2659 (w), 2640 (w), 2605 (w), 2601 (w), 2527 (w), 1672 (s), 1655 (s), 1635 (s), 1609 (vs), 1585 (vs), 1524 (s), 1502 (m), 1479 (w), 1453 (s), 1400 (vs), 1358 (vs), 1341 (vs), 1279 (vs), 1264 (vs), 1222 (s), 1183 (s), 1164 (vs), 1126 (vs), 1057 (vs), 1020 (s), 949 (vs), 935 (vs), 882 (s), 827 (vs), 802 (vs), 766 (vs), 747 (vs), 722 (vs), 656 (vs), 612 (s), 579 (s), 569 (s), 545 (s), 510 (vs), 499 (vs), 475 (vs), 449 (vs), 407 (vs), 375 (vs) cm^{-1}.

HRMS (FAB): *m/z* calcd. for $C_{21}H_{25}N_5O_2$: 379.2008 [M]; found: 379.2011.

UV/Vis (MeCN): λ_{max} = 400 nm.

Fluorescence (MeCN): λ_{em}= 534 nm (excited with λ = 370 nm).

Φ_F (MeCN): 0.027.

E-isomer

^{1}H NMR (400 MHz, DMSO-*d*$_6$): δ = 12.25 (bs, 1H, N*H*), 11.98 (s, 1H, N*H*), 10.51 (s, 1H, N*H*), 7.82 (s, 1H, C*H*, imidazole), 7.68 (d, J = 9.0 Hz, 2H, H_{arom}), 6.71 (s, 1H, C*H*, double bond), 6.64 (d, J = 9.0 Hz, 2H, H_{arom}), 6.44 (s, 1H, C*H*, double bond), 2.94 (s, 6H, N-(CH_3)$_2$), 1.37 (s, 9H, C(CH_3)$_3$) ppm.

^{13}C NMR (126 MHz, DMSO-*d*$_6$): δ = 157.1 (1C, *C*ONH), 156.1 (1C, *C*ONH), 149.9 (1C, C_{arom}-N(CH_3)$_2$), 139.4 (1C, *C*-C(CH_3)$_3$), 134.1 (1C, *C*H, imidazole), 132.6 (2C, C_{arom}-H), 130.9 (1C, C_{arom}-CH), 124.3 (1C, *C*-CH), 123.1 (1C, *C*-CH), 122.5 (1C, *C*H, double bond), 121.2 (1C, C_{arom}-CH), 111.0 (2C, C_{arom}-H), 102.7 (1C, *C*H, double bond), 39.4 (2C, N(CH_3)$_2$), 31.8 (1C, *C*(CH_3)$_3$), 30.6 (3C, C(CH_3)$_3$) ppm.

UV/Vis (MeCN): λ_{max} = 416 nm.

Fluorescence (MeCN): λ_{em}= 544 nm (excited with λ = 400 nm).

Φ_F (MeCN): 0.008.

*(Z)-3-((5-(tert-butyl)-1H-imidazol-4-yl)methylene)-6-((Z)-3-methoxybenzylidene)piperazine-2,5-dione (**5**)*

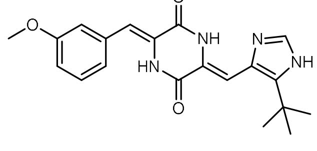

Z-isomer

^{1}H NMR (400 MHz, DMSO-d_6): δ = 12.34 (bs, 1H, N*H*), 12.25 (s, 1H, N*H*), 10.04 (s, 1H, N*H*), 7.85 (s, 1H, C*H*, imidazole), 7.33 (t, *J* = 7.9 Hz, 1H, H_{arom}), 7.12 – 7.04 (m, 2H, H_{arom}), 6.93 – 6.87 (m, 1H, H_{arom}), 6.86 (s, 1H, C*H*, double bond), 6.72 (s, 1H, C*H*, double bond), 3.79 (s, 3H, O-CH_3), 1.38 (s, 9H, C(CH_3)$_3$) ppm.

^{13}C NMR (101 MHz, DMSO-d_6): δ = 159.3 (1C, C_{arom}-OCH$_3$), 157.5 (1C, *C*ONH), 156.3 (1C, *C*ONH), 140.4 (1C, *C*-C(CH$_3$)$_3$), 134.5 (1C, *C*H, imidazole), 134.4 (1C, C-*C*H, imidazole), 130.7 (1C, C_{arom}-CH), 129.8 (1C, C_{arom}-H), 126.8 (1C, *C*-CH), 123.8 (1C, *C*-CH), 121.5 (1C, C_{arom}-H), 114.5 (1C, C_{arom}-H), 113.9 (1C, C_{arom}-H), 113.8 (1C, *C*H, double bond), 105.1 (1C, *C*H, double bond), 55.1 (1C, O-*C*H$_3$), 31.9 (1C, *C*(CH$_3$)$_3$), 30.6 (3C, C(*C*H$_3$)$_3$) ppm.

IR (ATR): v = 3159 (m), 3099 (m), 3046 (m), 2953 (m), 2921 (s), 2868 (m), 2851 (m), 1669 (vs), 1629 (vs), 1572 (s), 1507 (w), 1486 (m), 1466 (m), 1451 (m), 1412 (vs), 1375 (vs), 1343 (vs), 1316 (vs), 1293 (vs), 1279 (vs), 1262 (s), 1241 (s), 1204 (m), 1197 (m), 1184 (m), 1154 (s), 1146 (vs), 1089 (m), 1047 (vs), 1024 (m), 996 (w), 958 (vs), 898 (w), 881 (w), 871 (w), 860 (m), 840 (m), 812 (vs), 785 (vs), 775 (vs), 735 (vs), 686 (vs), 664 (vs), 615 (m), 595 (m), 582 (m), 561 (s), 551 (m), 533 (s), 500 (vs), 489 (s), 459 (s), 438 (vs), 414 (m), 394 (m), 375 (m) cm^{-1}.

HRMS (FAB): *m/z* calcd. for $C_{20}H_{23}O_3N_4$: 367.1765 [M+H]; found: 367.1763.

UV/Vis (MeCN): λ_{max} = 362 nm.

Fluorescence (MeCN): λ_{em}= 512 nm (excited with λ = 400 nm).

Φ_F (MeCN): 0.045.

***E*-isomer**

^{1}H NMR (400 MHz, DMSO-d_6): δ = 12.29 (bs, 1H, N*H*), 12.08 (s, 1H, N*H*), 10.73 (s, 1H, N*H*), 7.83 (s, 1H, C*H*, imidazole), 7.29 (s, 1H, H_{arom}), 7.19 (t, J = 7.9 Hz, 1H, H_{arom}), 7.03 (d, J = 7.7 Hz, 1H, H_{arom}), 6.81 (dd, J = 8.1, 2.6 Hz, 1H, H_{arom}), 6.78 (s, 1H, C*H*, double bond), 6.49 (s, 1H, C*H*, double bond), 3.74 (s, 3H, O-CH_3), 1.38 (s, 9H, C(CH_3)$_3$) ppm.

^{13}C NMR (101 MHz, DMSO-d_6): δ = 158.4 (1C, C_{arom}-OCH$_3$), 157.4 (1C, *C*ONH), 155.4 (1C, *C*ONH), 140.0 (1C, *C*-C(CH$_3$)$_3$), 135.6 (1C, C-*C*H, imidazole), 134.3 (1C, *C*H, imidazole), 130.7 (1C, C_{arom}-CH), 128.4 (1C, C_{arom}-H), 127.4 (1C, *C*-CH), 123.8 (1C, *C*-CH), 123.0 (1C, C_{arom}-H), 119.7 (1C, *C*H, double bond), 115.5 (1C, C_{arom}-H), 113.3 (1C, C_{arom}-H), 104.0 (1C, *C*H, double bond), 54.9 (1C, O-*C*H$_3$), 31.9 (1C, *C*(CH$_3$)$_3$), 30.6 (3C, C(*C*H$_3$)$_3$) ppm.

UV/Vis (MeCN): λ_{max} = 365 nm.

Fluorescence (MeCN): λ_{em}= 533 nm (excited with λ = 400 nm).

Φ_F (MeCN): 0.022.

*(Z)-3-((5-(tert-butyl)-1H-imidazol-4-yl)methylene)-6-((Z)-3,5-dimethoxybenzylidene)-piperazine-2,5-dione (**6**)*

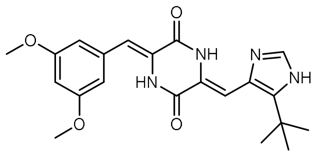

***Z*-isomer**

^{1}H NMR (400 MHz, DMSO-*d*$_6$): δ = 12.34 (bs, 1H, N*H*), 12.25 (s, 1H, N*H*), 10.02 (s, 1H, N*H*), 7.85 (s, 1H, C*H*, imidazole), 6.86 (s, 1H, C*H*, double bond), 6.68 (s, 1H, C*H*, double bond), 6.65 (d, *J* = 2.3 Hz, 2H, H_{arom}), 6.46 (t, *J* = 2.3 Hz, 1H, H_{arom}), 3.77 (s, 6H, O-CH_3), 1.38 (s, 9H, C(CH_3)$_3$) ppm.

^{13}C NMR (101 MHz, DMSO-*d6*): δ = 160.5 (2C, C_{arom}-OCH$_3$), 157.5 (1C, *C*ONH), 156.3 (1C, *C*ONH), 140.4 (1C, *C*-C(CH$_3$)$_3$), 135.0 (1C, C-*C*H, imidazole), 134.4 (1C, *C*H, imidazole), 130.7 (1C, C_{arom}-CH), 127.1 (1C, *C*-CH), 123.8 (1C, *C*-CH), 113.9 (1C, *C*H, double bond), 107.1 (2C, C_{arom}-H), 105.1 (1C, *C*H, double bond), 100.3 (1C, C_{arom}-H), 55.2 (2C, O-*C*H$_3$), 31.9 (1C, *C*(CH$_3$)$_3$), 30.6 (3C, C(*C*H$_3$)$_3$) ppm.

IR (ATR): *v* = 3434 (w), 3193 (m), 3078 (w), 3038 (w), 3007 (w), 2983 (w), 2961 (w), 2952 (w), 2928 (w), 2873 (w), 2837 (w), 1670 (vs), 1632 (vs), 1589 (vs), 1502 (w), 1451 (s), 1441 (m), 1414 (vs), 1374 (s), 1341 (vs), 1319 (s), 1290 (s), 1275 (m), 1259 (m), 1205 (vs), 1186 (vs), 1139 (vs), 1069 (s), 1055 (vs), 1014 (m), 973 (m), 955 (s), 880 (m), 840 (s), 819 (vs), 800 (vs), 765 (vs), 752 (vs), 739 (vs), 724 (vs), 696 (s), 677 (vs), 659 (vs), 630 (s), 613 (s), 579 (s), 557 (vs), 534 (vs), 516 (vs), 499 (vs), 482 (vs), 466 (vs), 441 (vs), 394 (vs), 378 (vs) cm^{-1}.

HRMS (FAB): *m/z* calcd. for $C_{21}H_{25}O_4N_4$: 397.1870 [M+H]; found: 397.1870.

UV/Vis (MeCN): λ_{max} = 363 nm.

Fluorescence (MeCN): λ_{em}= 515 nm (excited with λ = 400 nm).

Φ_F (MeCN): 0.045.

***E*-isomer**

^{1}H NMR (400 MHz, DMSO-*d*$_6$): δ = 12.29 (bs, 1H, N*H*), 12.08 (s, 1H, N*H*), 10.72 (s, 1H, N*H*), 7.83 (s, 1H, C*H*, imidazole), 6.78 (s, 1H, C*H*, double bond), 6.76 (d, *J* = 2.3 Hz, 2H, H_{arom}), 6.44 (s, 1H, C*H*, double bond), 6.39 (t, *J* = 2.3 Hz, 1H, H_{arom}), 3.72 (s, 6H, O-CH_3), 1.38 (s, 9H, C(CH_3)$_3$) ppm.

^{13}C NMR (101 MHz, DMSO-*d*$_6$): δ = 159.5 (2C, C_{arom}-OCH$_3$), 157.4 (1C, *C*ONH), 155.3 (1C, *C*ONH), 140.0 (1C, *C*-C(CH$_3$)$_3$), 136.1 (1C, C-*C*H, imidazole), 134.3 (1C, *C*H, imidazole), 130.7 (1C, C_{arom}-CH), 127.6 (1C, *C*H, double bond), 123.8 (1C, *C*H, double bond), 119.7 (1C, *C*H, double bond), 108.3 (2C, C_{arom}-H), 104.0 (1C, *C*H, double bond), 99.9 (1C, C_{arom}-H), 55.1 (2C, O-*C*H$_3$), 31.9 (1C, *C*(CH$_3$)$_3$), 30.6 (3C, C(*C*H$_3$)$_3$) ppm.

UV/Vis (MeCN): λ_{max} = 364 nm.

Fluorescence (MeCN): λ_{em}= 534 nm (excited with λ = 400 nm).

Φ_F (MeCN): 0.020.

Synthesis of Hemipiperazines

General procedure A

The aldehyde (1.00 equiv) was dissolved in dry DMF (2.0 mL/1 mmol aldehyde) under argon atmosphere. 1,4-Diacetylpiperazine-2,5-dione (1.50 equiv) and Cs_2CO_3 (1.50 equiv) were added and the reaction mixture was stirred at room temperature until the complete consumption of the aldehyde was detected. The reaction mixture was then poured on ice water and the precipitate was filtered off and dried under reduced pressure.

General procedure B

The aldehyde (1.00 equiv) and 1,4-diacetylpiperazine-2,5-dione (1.50 equiv) were dissolved in dry THF (6.0 mL/1 mmol aldehyde) under argon atmosphere and cooled down to 0 °C. KO^{t}Bu (1.10 equiv) was added carefully and subsequently the reaction mixture was allowed to warm up to room temperature and stirred until the complete consumption of the aldehyde was detected. The THF was removed under reduced pressure and the reaction mixture was then poured on ice water and the precipitate was filtered off and dried under reduced pressure.

*(Z)-1-Acetyl-3-benzylidenepiperazine-2,5-dione (**7**)*

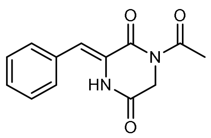

General procedure A.

The crude residue was purified *via* column chromatography (CH_2Cl_2:ethyl acetate; 10:1). (*Z*)-1-Acetyl-3-benzylidenepiperazine-2,5-dione (**7**) was isolated as a colorless solid in 57% yield (210 mg, 860 µmol).

To isolate the *E*-isomer, a solution of compound **7** in ethyl acetate was irradiated at 365 nm for 1 hour. The two photoisomers were then separated *via* column chromatography (CH_2Cl_2:ethyl acetate; 9:1).

***Z*-isomer**

^{1}H NMR (400 MHz, $CDCl_3$): δ = 7.96 (s, 1H, N*H*), 7.51 – 7.42 (m, 2H, H_{arom}), 7.39 (ddt, J = 6.1, 3.0, 1.9 Hz, 3H, H_{arom}), 7.18 (s, 1H, Ph-C*H*), 4.51 (s, 2H, CH_2), 2.66 (s, 3H, CH_3) ppm.

^{13}C NMR (101 MHz, $CDCl_3$): δ = 172.6 (1C, *C*OCH_3), 162.8 (1C, *C*ONH), 160.1 (1C, *C*ONCOCH_3), 132.7 (1C, C_{arom}-CH_2), 129.7 (2C, C_{arom}-H), 129.6 (2C, C_{arom}-H), 128.7 (1C, C_{arom}-H), 125.8 (1C, CO-*C*), 120.1 (1C, Ph-*C*H), 46.3 (1C, *C*H_2), 27.4 (1C, CO*C*H_3) ppm.

IR (ATR): v = 3269 (w), 3125 (w), 3044 (w), 3006 (w), 2939 (w), 1698 (vs), 1677 (vs), 1629 (vs), 1494 (w), 1455 (m), 1428 (m), 1404 (m), 1360 (vs), 1262 (vs), 1222 (vs), 1203 (vs), 1098 (vs), 1077 (s), 1034 (s), 1006 (s), 999 (s), 979 (vs), 960 (s), 933 (s), 873 (m), 860 (m), 853 (m),

837 (s), 790 (m), 768 (vs), 735 (s), 715 (vs), 701 (s), 680 (vs), 653 (vs), 618 (s), 596 (s), 584 (s), 555 (vs), 534 (vs), 476 (vs), 428 (vs), 401 (s) cm^{-1}.

HRMS (FAB): *m*/*z* calcd. for $C_{13}H_{13}N_2O_3$ [M+H], 245.0921; found, 245.0923.

EA ($C_{13}H_{12}N_2O_3$): calcd. C 63.93; H 4.95; N 11.47; O 19.65; found C 63.95; H 4.98; N 11.28.

UV/Vis (MeCN): λ_{max} = 316 nm.

***E*-isomer**

^{1}H NMR (400 MHz, $CDCl_3$): δ = 8.63 (s, 1H, N*H*), 7.52 – 7.45 (m, 2H, H_{arom}), 7.44 – 7.29 (m, 3H, H_{arom}), 6.61 (s, 1H, Ph-C*H*), 4.50 (s, 2H, CH_2), 2.57 (s, 3H, CH_3) ppm.

^{13}C NMR (101 MHz, $CDCl_3$): δ = 172.5 (1C, *C*OCH$_3$), 165.6 (1C, *C*ONH), 159.8 (1C, *C*ON COCH$_3$), 133.1 (1C, C_{arom}-CH$_2$), 130.0 (2C, C_{arom}-H), 129.0 (1C, C_{arom}-H), 128.2 (2C, C_{arom}-H), 127.5 (1C, Ph-*C*H), 125.3 (1C, CO-*C*), 45.8 (1C, *C*H$_2$), 27.4 (1C, CO*C*H$_3$) ppm.

*(Z)-1-Acetyl-3-(4-methoxybenzylidene)piperazine-2,5-dione (**8**)*

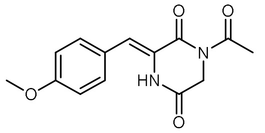

General procedure A.

The crude residue was purified *via* column chromatography (cyclohexane:ethyl acetate; 2:1 -> 3:2). The target compound was isolated as a pale-yellow solid in 55% yield.

To isolate the *E*-isomer a solution of compound **8** in ethyl acetate was irradiated at 365 nm for 1 hour. The two photoisomers were then separated *via* column chromatography (cyclohexane:ethyl acetate; 2:1 -> 3:2).

***Z*-isomer**

^{1}H NMR (400 MHz, DMSO-*d*$_6$): δ = 10.28 (s, 1H), 7.58 (d, *J* = 8.8 Hz, 2H), 7.01 (d, *J* = 8.8 Hz, 2H), 6.95 (s, 1H), 4.37 (s, 2H), 3.81 (s, 3H), 2.50 (s, 3H) ppm.

^{13}C NMR (101 MHz, DMSO-*d*$_6$): δ = 171.8, 164.2, 162.1, 159.7, 131.5, 125.4, 124.9, 119.7, 114.2, 55.3, 45.5, 26.6 ppm.

IR (ATR): ν = 387 (w), 402 (w), 419 (m), 453 (s), 470 (m), 504 (s), 538 (vs), 550 (s), 574 (m), 603 (m), 626 (m), 652 (m), 721 (s), 745 (vs), 754 (s), 779 (s), 810 (vs), 820 (vs), 882 (m), 894 (m), 933 (m), 948 (m), 982 (s), 1000 (s), 1021 (vs), 1040 (m), 1101 (s), 1119 (m), 1181 (vs), 1205 (vs), 1222 (s), 1261 (vs), 1302 (w), 1356 (vs), 1370 (vs), 1407 (vs), 1445 (m), 1455 (w), 1513 (s), 1570 (w), 1601 (vs), 1622 (vs), 1679 (vs), 1696 (s), 2840 (w), 2904 (w), 2929 (w), 2969 (w), 3000 (w), 3014 (w), 3038 (w), 3065 (w), 3075 (w), 3091 (w), 3099 (w), 3122 (w), 3173 (w), 3206 (w), 3223 (w), 3231 (w) cm^{-1}.

HRMS (FAB): *m*/*z* calcd. for $C_{14}H_{14}N_2O_4$ [M+H], 274.0948; found, 274.0947.

EA ($C_{13}H_{12}N_2O_3$): calcd. C 61.31; H 5.15; N 10.21; O 23.33. Found C 61.41; H 5.18; N 10.17.

UV/Vis (MeCN): λ_{max} = 333 nm.

***E*-isomer**

^{1}H NMR (400 MHz, DMSO-*d*$_6$): δ = 10.51 (s, 1H), 7.51 (d, J = 8.8 Hz, 2H), 6.89 (d, J = 8.8 Hz, 2H), 6.54 (s, 1H), 4.29 (s, 2H), 3.77 (s, 3H), 2.42 (s, 3H) ppm.

^{13}C NMR (101 MHz, DMSO-*d*$_6$): δ = 171.9, 164.1, 160.5, 159.1, 131.8, 126.1, 125.5, 124.0, 113.2, 55.1, 45.7, 26.9 ppm.

UV/Vis (MeCN): λ_{max} = 343 nm.

*(Z)-1-Acetyl-3-(3-methoxybenzylidene)piperazine-2,5-dione (**9**)*

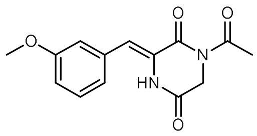

General procedure A.

The crude residue was purified *via* column chromatography (CH_2Cl_2:ethyl acetate; 9:1) followed by recrystallization from toluene. The target compound was isolated as a colorless solid in 18% yield.

^{1}H NMR (400 MHz, DMSO-*d*$_6$): δ = 10.37 (s, 1H), 7.36 (t, J = 8.2 Hz, 1H), 7.18-7.16 (brm, 1.3 Hz, 2H), 6.99 – 6.92 (brm, 2H), 4.38 (s, 2H), 3.81 (s, 3H), 2.51 (s, 3H) ppm.

^{13}C NMR (101 MHz, DMSO-*d*$_6$): δ = 171.8, 164.1, 161.6, 159.2, 134.2, 129.7, 127.0, 122.1, 118.8, 114.8, 114.5, 55.1, 45.6, 26.7 ppm.

IR (ATR): v = 3184 (w), 3160 (w), 3098 (w), 3074 (w), 3030 (w), 3014 (w), 2968 (w), 2919 (w), 2873 (w), 2851 (w), 2842 (w), 1700 (vs), 1677 (vs), 1628 (vs), 1602 (m), 1572 (s), 1487 (w), 1456 (w), 1432 (m), 1408 (s), 1378 (vs), 1366 (vs), 1315 (m), 1300 (m), 1278 (m), 1251 (vs), 1227 (vs), 1197 (vs), 1180 (s), 1162 (vs), 1105 (m), 1047 (vs), 1003 (m), 994 (m), 984 (m), 970 (w), 938 (m), 914 (w), 890 (w), 880 (w), 851 (m), 823 (m), 789 (vs), 775 (s), 747 (vs), 720 (m), 691 (vs), 657 (s), 615 (w), 605 (m), 562 (m), 551 (s), 516 (w), 477 (w), 462 (w), 442 (s), 414 (w) cm^{-1}

HRMS (FAB): *m*/*z* calcd. for $C_{14}H_{14}N_2O_4$ [M+H], 274.2022; found, 274.2023.

UV/Vis (MeCN): λ_{max} = 317 nm.

*(Z)-1-Acetyl-3-(2-methoxybenzylidene)piperazine-2,5-dione (**10**)*

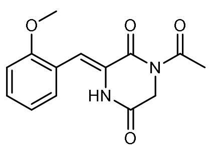

General procedure A.

The crude residue was purified *via* column chromatography (toluene:ethyl acetate; 1:0 -> 4:1) followed by recrystallization from toluene. The target compound was isolated as a colorless solid in 58% yield.

^{1}H NMR (400 MHz, DMSO-*d*$_6$): δ = 10.10 (s, 1H), 7.54 (dd, *J* = 7.8, 1.9 Hz, 1H), 7.36 (ddd, *J* = 8.8, 7.4, 1.8 Hz, 1H), 7.08 (s, 1H), 7.08 (d, *J* = 8.5 Hz, 1H), 7.02 (t, *J* = 7.5 Hz, 1H), 4.35 (s, 2H), 3.85 (s, 3H), 2.50 (s, 3H) ppm.

^{13}C NMR (101 MHz, DMSO-*d*$_6$): δ = 171.9, 163.7, 161.4, 157.2, 130.4, 129.7, 126.6, 121.6, 120.5, 114.3, 111.4, 55.6, 45.7, 26.7 ppm.

IR (ATR): *v* = 3189 (w), 3094 (vw), 3019 (vw), 2978 (vw), 2934 (w), 2836 (vw), 1697 (vs), 1676 (vs), 1630 (s), 1594 (w), 1574 (w), 1486 (w), 1459 (m), 1401 (m), 1373 (vs), 1361 (vs), 1306 (w), 1289 (m), 1259 (s), 1245 (vs), 1221 (vs), 1191 (s), 1157 (s), 1105 (m), 1047 (w), 1017 (m), 1001 (m), 983 (w), 884 (w), 861 (w), 834 (w), 807 (w), 758 (vs), 744 (m), 731 (m), 720 (m), 659 (w), 605 (w), 579 (w), 545 (s), 527 (w), 470 (w), 432 (w) cm^{-1}.

HRMS (FAB): *m*/*z* calcd. for $C_{14}H_{14}N_2O_4$ [M+H],; 274.2093; found, 274.2094.

UV/Vis (MeCN): λ_{max} = 328 nm.

*(Z)-1-Acetyl-3-(4-(dimethylamino)benzylidene)piperazine-2,5-dione (**11**)*

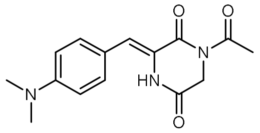

General procedure B.

The crude residue was purified *via* column chromatography (CH_2Cl_2:ethyl acetate; 9:1). The target compound was isolated as a yellow solid in 57% yield.

To isolate the *E*-isomer a solution of compound **11** in ethyl acetate was irradiated at 365 nm for 1 hour. The two photoisomers were then separated *via* column chromatography (CH_2Cl_2:ethyl acetate; 9:1 -> 9:2).

***Z*-isomer**

^{1}H NMR (400 MHz, DMSO-*d*$_6$): δ = 10.12 (s, 1H), 7.49 (d, *J* = 8.9 Hz, 2H), 6.91 (s, 1H), 6.75 (d, *J* = 9.0 Hz, 2H), 4.35 (s, 2H), 2.98 (s, 6H), 2.48 (s, 3H) ppm.

^{13}C NMR (101 MHz, DMSO-d_6): δ = 171.7, 164.1, 162.6, 150.6, 131.5, 122.3, 121.8, 120.2, 111.7, 45.3, 26.4 ppm.

IR (ATR): v = 3169 (w), 3084 (w), 3077 (w), 3067 (w), 3014 (w), 3002 (w), 2959 (w), 2891 (w), 2860 (w), 2803 (w), 1679 (vs), 1615 (m), 1589 (vs), 1550 (m), 1523 (s), 1485 (w), 1458 (m), 1441 (m), 1411 (s), 1351 (vs), 1329 (s), 1313 (s), 1262 (vs), 1224 (vs), 1198 (s), 1181 (vs), 1166 (vs), 1098 (s), 1067 (s), 1037 (s), 1003 (s), 982 (s), 963 (s), 942 (s), 899 (s), 882 (s), 854 (s), 812 (vs), 793 (vs), 752 (s), 741 (vs), 718 (s), 694 (s), 657 (s), 628 (s), 599 (vs), 578 (s), 551 (vs), 524 (vs), 506 (vs), 482 (vs), 456 (vs), 428 (s), 419 (s), 404 (s), 390 (s), 378 (s) cm^{-1}.

HRMS (FAB): m/z calcd. for $C_{15}H_{17}N_3O_3$ [M+H], 287.1264; found, 287.1264.

EA ($C_{15}H_{17}N_3O_3$): calcd. C 62.71; H 5.96; N 14.63; O 16.71. Found C 62.64; H 5.98; N 14.28.

UV/Vis (MeCN): λ_{max} = 386 nm.

Φ_F (DMSO): 0.004.

***E*-isomer**

^{1}H NMR (400 MHz, DMSO-d_6): δ = 10.35 (s, 1H), 7.56 (d, J = 9.0 Hz, 2H), 6.66 (d, J = 9.0 Hz, 2H), 6.50 (s, 1H), 4.28 (s, 2H), 2.95 (s, 6H), 2.45 (s, 3H) ppm.

^{13}C NMR (101 MHz, DMSO-d_6): δ = 171.8, 164.1, 161.0, 150.3, 132.3, 126.5, 122.7, 120.6, 111.0, 45.5, 26.8 ppm.

*(Z)-1-Acetyl-3-(4-(diphenylamino)benzylidene)piperazine-2,5-dione (**12**)*

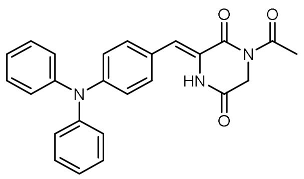

General procedure A.

The crude residue was purified *via* column chromatography (CH_2Cl_2:ethyl acetate; 1:0 -> 9:1). The target compound was isolated as a yellow solid in 65% yield.

^{1}H NMR (400 MHz, DMSO-d_6): δ = 10.27 (s, 1H), 7.50 (d, J = 8.9 Hz, 2H), 7.35 (dd, J = 8.5, 7.4 Hz, 4H), 7.12 (tt, J = 7.3, 1.3 Hz, 2H), 7.08 (d, J = 7.4 Hz, 4H), 6.93 (d, J = 8.9 Hz, 2H), 6.91 (s, 1H), 4.35 (s, 2H), 2.45 (d, J = 15.3 Hz, 3H) ppm.

^{13}C NMR (101 MHz, DMSO-d_6): δ = 171.8, 164.1, 162.0, 147.7, 146.5, 131.1, 129.7, 126.3, 125.1, 124.8, 124.0, 121.3, 119.5, 45.5, 26.6 ppm.

^{1}H NMR (400 MHz, $CDCl_3$): δ = 7.89 (s, 1H), 7.39 – 7.27 (m, 4H), 7.25 (d, J = 8.0 Hz, 3H), 7.16 – 7.09 (m, 7H), 7.06 (d, J = 8.8 Hz, 2H), 4.51 (s, 2H), 2.64 (s, 3H) ppm.

^{13}C NMR (101 MHz, $CDCl_3$): δ = 172.7, 162.9, 160.5, 149.2, 146.8, 130.0, 129.7, 125.6, 125.1, 124.4, 123.9, 122.1, 120.6, 46.2, 27.3 ppm.

IR (ATR): *v* = 3234 (w), 3196 (w), 3101 (w), 3057 (w), 3038 (w), 1687 (vs), 1677 (vs), 1618 (m), 1602 (m), 1585 (vs), 1509 (m), 1485 (vs), 1455 (w), 1442 (m), 1432 (m), 1409 (s), 1374 (s), 1366 (s), 1356 (m), 1333 (s), 1302 (m), 1288 (m), 1265 (vs), 1207 (s), 1196 (s), 1176 (s), 1150 (s), 1130 (m), 1099 (m), 1079 (s), 1044 (m), 1038 (m), 1031 (s), 1000 (m), 984 (s), 970 (m), 952 (m), 929 (m), 921 (m), 904 (m), 894 (s), 841 (s), 827 (vs), 793 (s), 778 (s), 751 (vs), 734 (vs), 722 (vs), 694 (vs), 654 (s), 630 (vs), 622 (vs), 603 (s), 595 (s), 569 (m), 560 (s), 543 (vs), 517 (s), 500 (vs), 455 (vs), 428 (vs), 415 (s), 407 (vs), 392 (m), 377 (m) cm^{-1}.

HRMS (FAB): *m*/*z* calcd. for $C_{25}H_{21}N_3O_3$ [M+H], 411.1577; found, 411.1579.

UV/Vis (MeCN): λ_{max} = 389 nm.

Φ_F (DMSO): 0.030.

*(Z)-4-((4-Acetyl-3,6-dioxopiperazin-2-ylidene)methyl)benzonitrile (**13**)*

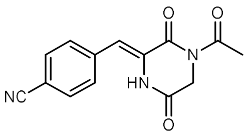

General procedure A.

The crude residue was purified *via* column chromatography (cyclohexane:ethyl acetate; 4:1 -> 1:1). The target compound was isolated as a pale-yellow solid in 52% yield.

^{1}H NMR (400 MHz, DMSO-*d*$_6$): δ = 10.61 (s, 1H), 7.88 (d, *J* = 8.4 Hz, 2H), 7.73 (d, *J* = 8.1 Hz, 2H), 6.96 (s, 1H), 4.37 (s, 2H), 2.50 (s, 3H) ppm.

^{13}C NMR (101 MHz, DMSO-*d*$_6$): δ = 171.8, 164.2, 161.1, 138.1, 132.4, 130.3, 129.2, 118.8, 116.2, 110.3, 45.8, 26.8 ppm.

IR (ATR): *v* = 3279 (w), 3268 (w), 3223 (w), 3114 (w), 3104 (w), 3072 (w), 3050 (w), 3041 (w), 3036 (w), 3013 (w), 2997 (w), 2942 (w), 2227 (w), 1686 (vs), 1621 (vs), 1604 (s), 1506 (w), 1436 (m), 1421 (w), 1397 (m), 1358 (vs), 1285 (m), 1256 (vs), 1222 (vs), 1201 (vs), 1098 (s), 1043 (s), 1021 (m), 997 (s), 976 (s), 962 (m), 891 (s), 885 (s), 839 (s), 827 (s), 813 (s), 800 (s), 764 (s), 748 (vs), 718 (s), 696 (s), 654 (m), 632 (s), 599 (m), 561 (vs), 544 (vs), 487 (s), 462 (s), 449 (vs), 429 (vs), 404 (s), 387 (m) cm^{-1}.

HRMS (FAB): *m*/*z* calcd. for $C_{14}H_{12}N_3O_3$ [M+H], 270.0873; found, 270.0870.

UV/Vis (MeCN): λ_{max} = 325 nm.

Synthesis of "Locked" Plinabulin

*Methyl 2-(1H-indole-2-carbonylamino)acetate (**16**)*

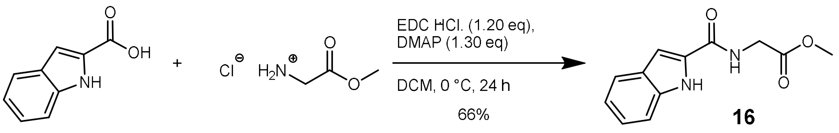

To a solution of 1*H*-indole-2-carboxylic acid (10.0 g, 62.1 mmol, 1.00 equiv) and the methyl 2-aminoacetate hydrochloride (9.35 g, 74.5 mmol, 1.20 equiv) in 620 mL DCM, EDC•HCl (14.3 g, 74.5 mmol, 1.20 equiv) and DMAP (12.1 g, 99.3 mmol, 1.60 equiv) were added at 0 °C. After stirring for 4 h at 0 °C, the solution was allowed to warm to ambient temperature and stirred for additional 20 h. The precipitate was filtered off and the mother liquor was washed with water (300 mL) and 10% HCl solution (300 mL) and evaporated *in vacuo*. The residual solid was combined with the precipitate from the first filtration step and purified by recrystallization from methanol to yield 9.05 g (40.9 mmol, 66% yield) of methyl 2-(1*H*-indole-2-carbonylamino)acetate **16** as a colorless solid.

^{1}H NMR (400 MHz, DMSO-*d*$_6$): δ = 11.62 (s, 1H), 8.95 (t, *J* = 6.0 Hz, 1H), 7.63 (d, *J* = 7.9, 1.1 Hz, 1H), 7.44 (d, *J* = 8.3, 1.0 Hz, 1H), 7.19 (ddd, *J* = 8.2, 7.0, 1.2 Hz, 1H), 7.16 (dd, *J* = 2.2, 0.9 Hz, 1H), 7.04 (t, *J* = 1.0 Hz, 1H), 4.06 (d, *J* = 5.9 Hz, 2H), 3.67 (s, 3H) ppm.

^{13}C NMR (101 MHz, DMSO-*d*$_6$): δ = 170.5, 161.5, 136.5, 131.0, 127.0, 123.5, 121.6, 119.8, 112.3, 103.0, 51.7, 40.8 ppm.

IR (ATR): *v* = 3367 (m), 3271 (vs), 3194 (w), 3153 (w), 3132 (w), 3084 (w), 3055 (w), 3043 (w), 3027 (w), 2996 (w), 2946 (w), 1737 (vs), 1639 (vs), 1621 (s), 1577 (m), 1548 (vs), 1511 (m), 1497 (w), 1449 (w), 1438 (m), 1421 (s), 1409 (s), 1370 (m), 1344 (w), 1315 (s), 1275 (s), 1215 (vs), 1184 (s), 1159 (m), 1140 (s), 1116 (m), 1077 (w), 1023 (w), 1001 (w), 983 (m), 949 (w), 932 (w), 849 (w), 822 (vs), 781 (vs), 773 (s), 747 (vs), 730 (vs), 703 (s), 628 (s), 611 (m), 585 (w), 558 (vs), 538 (s), 469 (s), 441 (vs), 375 (s) cm^{-1}.

HRMS (FAB): *m*/*z* calcd. for $C_{12}H_{12}N_2O_3$ [M+H], 232.0922 g/mol; found, 232.0921.

*2-(1H-Indole-2-carbonylamino)acetic acid (**17**)*

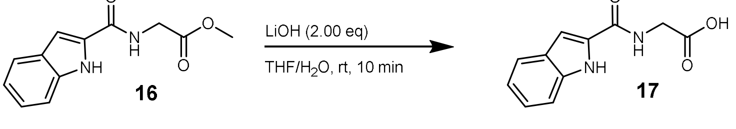

To a solution of methyl 2-(1*H*-indole-2-carbonylamino)acetate **16** (150 mg, 646 µmol, 1.00 equiv) in THF (3.00 mL) LiOH (30.9 mg, 1.29 mmol, 2.00 equiv) in water (3.00 mL) was added and the reaction mixture was stirred at room temperature for 10 min. The reaction was quenched by adding aq. solution of HCl (2 M, 1 mL). Subsequently, THF was removed *in vacuo* and the precipitate was filtererd off and dried. The resulting slightly yellow solid (**17**) was used for the next reaction step without further purification.

^{1}H NMR (400 MHz, DMSO-d_6): δ = 12.63 (s, 1H), 11.60 (s, 1H), 8.83 (t, J = 6.0 Hz, 1H), 7.63 (d, J = 8.0 Hz, 1H), 7.43 (dd, J = 8.2, 1.0 Hz, 1H), 7.18 (ddd, J = 8.2, 6.9, 1.2 Hz, 1H), 7.15 – 7.14 (m, 2H), 7.04 (ddd, J = 8.0, 6.9, 1.0 Hz, 1H), 3.96 (d, J = 6.0 Hz, 2H) ppm.

*2,3-Dihydropyrazino[1,2-a]indole-1,4-dione (**18**)*

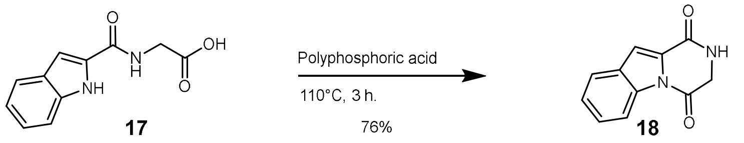

2-(1*H*-Indole-2-carbonylamino)acetic acid **17** (6.40 g, 29.3 mmol, 1.00 equiv) in polyphosphoric acid (265 g, 1.49 mol, 50.8 equiv) was stirred at 110 °C for 3 hours. The reaction was quenched carefully by adding an ice/water mixture and subsequently extracted with ethyl acetate. The combined organic layers were dried over Na_2SO_4 and concentrated *in vacuo* to yield 2,3-dihydropyrazino[1,2-a]indole-1,4-dione **18** (4.47 g, 22.4 mmol, 76%) as a brown solid.

^{1}H NMR (400 MHz, DMSO-d_6): δ = 8.49 (s, 1H), 8.37 (dd, J = 8.3, 1.0 Hz, 1H), 7.80 (d, J = 7.9 Hz, 1H), 7.52 (ddd, J = 8.5, 7.3, 1.4 Hz, 1H), 7.41 (td, J = 7.6, 1.2 Hz, 1H), 7.36 (s, 1H), 4.42 (d, J = 2.1 Hz, 2H) ppm.

^{13}C NMR (101 MHz, DMSO-d_6): δ = 163.5, 156.4, 134.2, 129.9, 128.5, 127.2, 124.8, 122.5, 115.7, 111.8, 46.7 ppm.

IR (ATR): ν = 3390 (vw), 3367 (vw), 3350 (vw), 3322 (vw), 3308 (vw), 3291 (vw), 3284 (vw), 3275 (vw), 3267 (vw), 3254 (vw), 3245 (vw), 3170 (w), 3129 (w), 3102 (w), 3041 (w), 3034 (w), 2952 (w), 2911 (w), 2873 (w), 2825 (w), 2751 (vw), 2738 (vw), 1703 (vs), 1672 (vs), 1605 (m), 1592 (vs), 1574 (s), 1499 (w), 1477 (w), 1434 (vs), 1417 (s), 1380 (vs), 1356 (s), 1332 (vs), 1309 (m), 1298 (m), 1259 (w), 1241 (w), 1203 (m), 1187 (w), 1153 (m), 1132 (s), 1109 (m), 1079 (s), 1037 (m), 1010 (w), 986 (w), 953 (w), 919 (w), 898 (w), 867 (w), 841 (s), 823 (s), 752 (vs), 731 (vs), 693 (m), 653 (m), 623 (w), 609 (w), 595 (w), 571 (m), 538 (vs), 484 (s), 469 (vs), 433 (s), 412 (m), 398 (m) cm^{-1}.

HRMS (EI): *m*/*z* calcd. for $C_{11}H_8N_2O_2$ [M+H], 200.0580 g/mol; found, 200.0580.

*2-Acetyl-2,3-dihydropyrazino[1,2-a]indole-1,4-dione (**19**)*

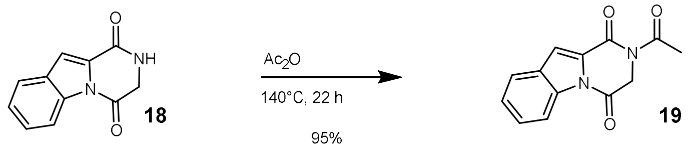

2,3-Dihydropyrazino[1,2-a]indole-1,4-dione **18** (2.00 g, 9.99 mmol, 1.00 equiv) was dissolved in acetic anhydride (229 g, 212 mL, 2.24 mol, 224 equiv) and stirred under reflux at 140 °C for 22 hours. The solvent was removed under reduced pressure and the product was washed with diethyl ether and filtered off to yield 2.30 g (9.50 mmol, 95% yield) of 2-acetyl-2,3-dihydropyrazino[1,2-*a*]indole-1,4-dione **19** as a beige solid.

^{1}H NMR (400 MHz, DMSO-d_6): δ = 8.35 (d, J = 8.3 Hz, 1H), 7.87 (dt, J = 7.9, 1.0 Hz, 1H), 7.70 (s, 1H), 7.60 (ddd, J = 8.4, 7.2, 1.3 Hz, 1H), 7.46 (td, J = 7.6, 7.2, 1.1 Hz, 1H), 4.69 (s, 3H), 2.58 (s, 3H) ppm.

^{13}C NMR (101 MHz, DMSO-d_6): δ = 171.7, 162.1, 156.9, 134.2, 129.2, 128.7, 128.4, 125.2, 123.1, 115.7, 115.6, 48.9, 27.2 ppm.

IR (ATR): ν = 1720 (vs), 1696 (vs), 1679 (vs), 1606 (w), 1557 (s), 1436 (s), 1404 (vs), 1370 (s), 1360 (vs), 1336 (vs), 1266 (vs), 1234 (s), 1210 (vs), 1160 (s), 1139 (s), 1115 (m), 1099 (s), 1069 (m), 1044 (m), 1010 (m), 979 (m), 967 (s), 948 (m), 878 (m), 853 (s), 839 (m), 800 (w), 755 (vs), 739 (vs), 694 (m), 670 (m), 642 (w), 618 (m), 602 (m), 585 (m), 575 (m), 550 (m), 537 (s), 499 (w), 484 (w), 448 (w), 432 (s), 407 (w), 388 (w), 375 (m) cm^{-1}.

HRMS (EI): m/z calcd. for $C_{13}H_{10}N_2O_3$ [M+H], 242.0686 g/mol; found, 242.0687.

UV/Vis (MeCN): λ_{max} = 211, 235, 299 nm.

(Z)-3-((5-(tert-Butyl)-1H-imidazol-4-yl)methylene)-2,3-dihydropyrazino[1,2-a]indole-1,4-dione ***15***

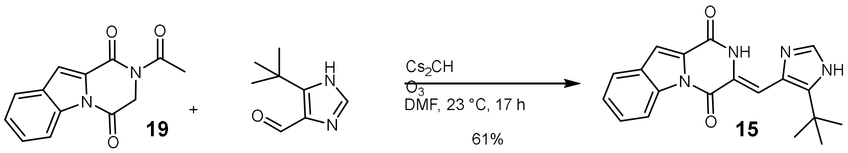

5-*tert*-Butyl-1*H*-imidazole-4-carbaldehyde (251 mg, 1.65 mmol, 1.00 equiv) was dissolved in dry DMF (5.3 mL) under argon atmosphere. The diketopiperazine **19** (400 mg, 1.65 mmol, 1.00 equiv) and Cs_2CO_3 (637 mg, 3.30 mmol, 2.00 equiv) were added and the mixture was stirred for 17 hours under argon atmosphere at room temperature. The reaction mixture was poured on an ice water/brine mix (30 mL) and the precipitate was filtered off and dried. The crude product was purified *via* HPLC (gradient of 40-80% MeCN in H_2O within 40 min, 0.1% TFA (v/v) in the solvents) and washed with sat. aq. solution of $NaHCO_3$. (*Z*)-3-((5-(*tert*-butyl)-1*H*-imidazol-4-yl)methylene)-2,3-dihydropyrazino[1,2-a]indole-1,4-dione **15** (210 mg, 628 µmol, 61% yield) was obtained as a yellow solid.

^{1}H NMR (400 MHz, DMSO-d_6): δ = 12.49 (s, 1H), 12.40 (s, 1H), 8.51 (dd, J = 8.3, 0.9 Hz, 1H), 7.94 (d, J = 0.8 Hz, 1H), 7.85 (dt, J = 7.9, 1.1 Hz, 1H), 7.56 (ddd, J = 8.4, 7.3, 1.3 Hz, 1H), 7.51 (d, J = 0.9 Hz, 1H), 7.43 (ddd, J = 8.1, 7.1, 1.1 Hz, 1H), 7.27 (d, J = 0.8 Hz, 1H), 1.44 (s, 9H) ppm.

^{13}C NMR (101 MHz, DMSO-d_6): δ = 156.3, 153.5, 142.3, 135.1, 135.0, 130.8, 128.9, 128.5, 127.4, 124.9, 124.0, 122.9, 116.2, 112.2, 109.2, 32.2, 30.7 ppm.

IR (ATR): ν = 3207 (s), 3084 (m), 3053 (m), 3029 (m), 2955 (m), 2925 (m), 2904 (m), 2868 (m), 2803 (w), 1693 (vs), 1657 (vs), 1606 (vs), 1582 (s), 1568 (s), 1502 (s), 1475 (w), 1453 (s), 1442 (vs), 1404 (vs), 1395 (vs), 1373 (vs), 1366 (vs), 1349 (vs), 1336 (vs), 1295 (s), 1245 (s), 1197 (vs), 1183 (vs), 1135 (vs), 1099 (vs), 1055 (vs), 1030 (vs), 1004 (s), 949 (vs), 933 (s), 849 (vs), 834 (vs), 829 (vs), 815 (vs), 802 (vs), 744 (vs), 732 (vs), 725 (vs), 704 (vs), 666 (s), 652 (vs), 612 (s), 589 (vs), 582 (vs), 537 (vs), 513 (vs), 477 (vs), 458 (vs), 446 (vs), 429 (vs), 405 (s), 388 (s) cm^{-1}.

HRMS (EI): *m*/*z* calcd. for $C_{19}H_{18}N_4O_2$ [M], 334.1424 g/mol; found, 334.1424.

UV/Vis (MeCN): λ_{max} = 209, 263, 271, 389 nm.

Fluorescence (MeCN): λ_{em}= 470 nm (excited with λ = 380 nm).

Φ_F (MeCN): 0.242.

Φ_F (DMSO): 0.233.

Φ_F (DMSO/ascorbic acid): 0.388.

Cell viability assays (MTT)

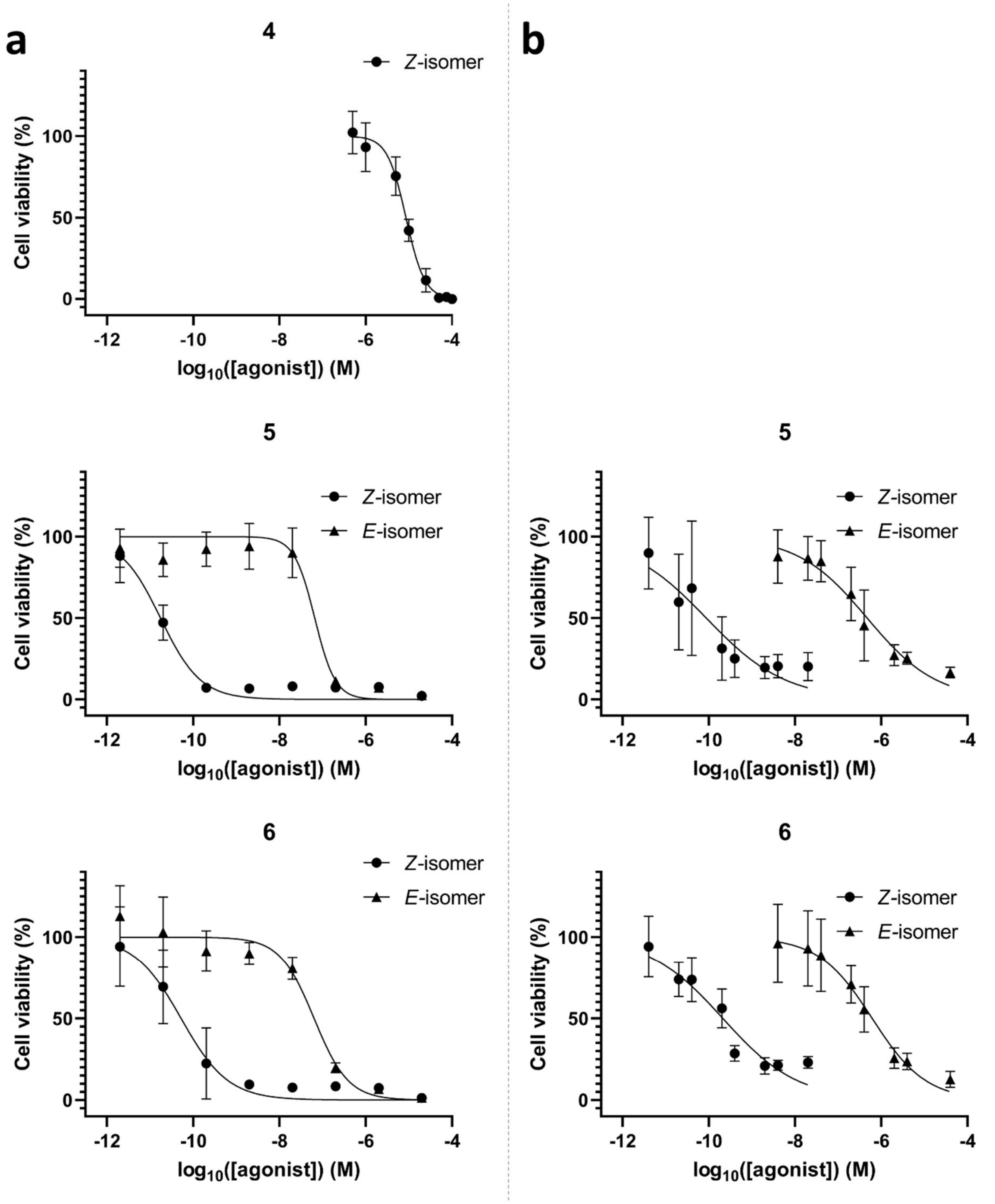

Supplementary Figure 1: ***a)*** *Viability of HT-29 cells after treatment with the HPLC-purified Z- and E-isomers of* ***4-6*** *for 48 hours. For compound* ***4*** *the E-isomer was not tested. Data were plotted against the log of agonist concentration (log*$_{10}$*([agonist]) (M)) with mean and SD values in GraphPad Prism. Number of independent experiments (six technical replicates per test):* ***4-Z*** *N = 3;* ***5*** *N = 3,* ***6;*** *N = 3.* ***b)*** *Viability of HT-29 cells after treatment with the respective photoisomers of* ***5*** *and* ***6*** *with concentrations in close range of the respective IC*$_{50}$ *values. Data were plotted against the log of agonist concentration (log*$_{10}$*([agonist]) (M)) with mean and SD values in GraphPad Prism. Number of independent experiments (six technical replicates per test):* ***5*** *N = 2,* ***6-Z;*** *N = 2;* ***6-E*** *N = 2.*

*Supplementary Table 1: Viability assays of isolated photoisomers. The IC_{50} values were determined via viability assays with HT-29 cells after treatment with the respective photoisomers of **1**-**6** for 48 hours. Due to the limited solubility, the IC_{50} value of the E-**4** was not determined. Also, for **2** the less active E-**2** was irradiated with cyan light (490 nm) and the resulting mixture was tested. Data were processed using GraphPad Prism.*

Compound	IC_{50}: *Z*-isomer (nM) [95% profile likelihood]	IC_{50}: *E*-isomer (nM) [95% profile likelihood]
**1**	0.47 [0.35 to 0.62]	92 [80 to 100]
**2**	0.27 [0.18 to 0.40]	420 [347 to 510]
**3**	38 [31 to 46]	> 1000
**4**	> 1000	-
**5**	0.09 [0.05 to 0.15]	471 [352 to 636]
**6**	0.21 [0.16 to 0.28]	618 [487 to 788]

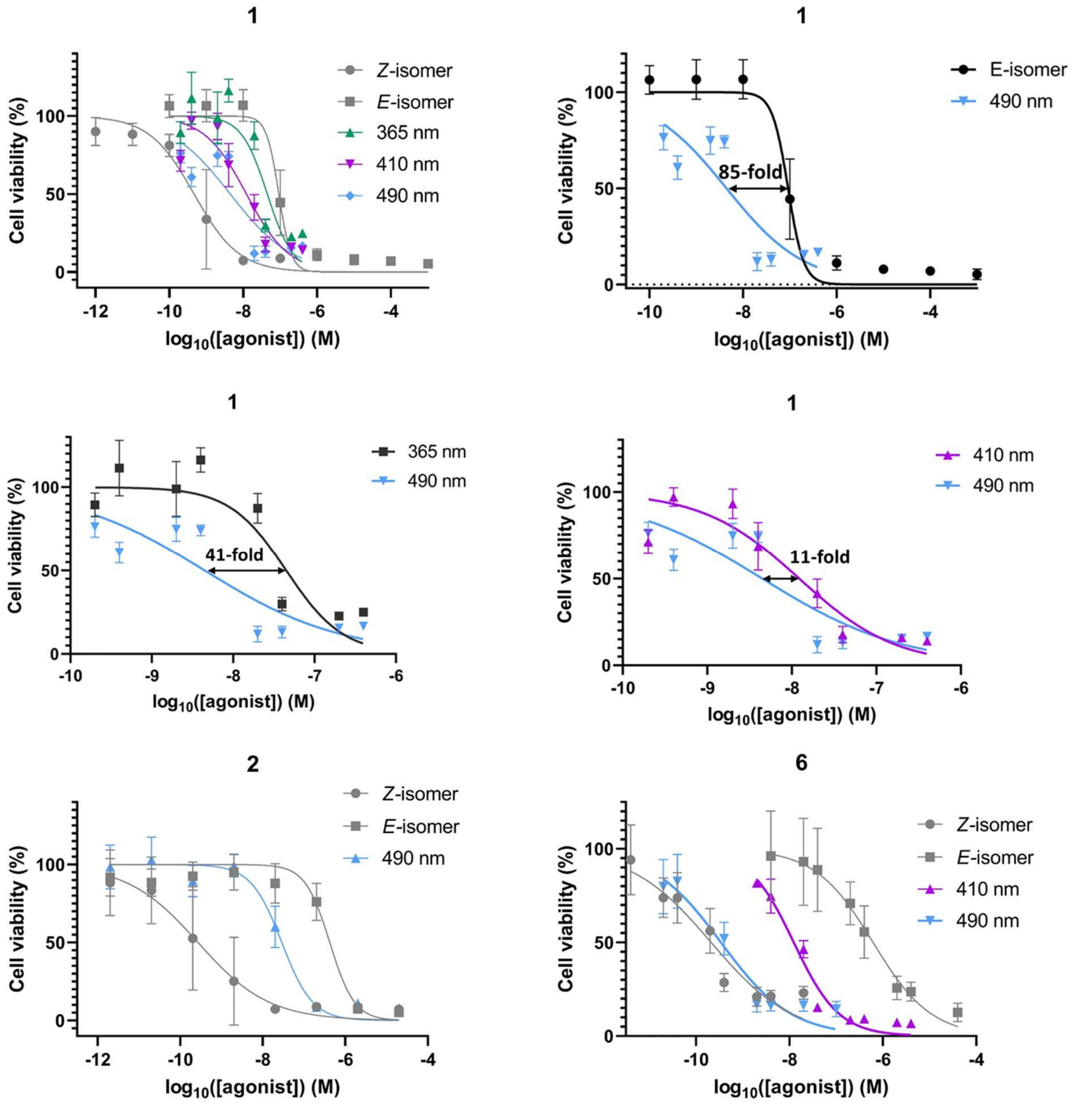

*Supplementary Figure 2: Viability of HT-29 cells after treatment with the mixture of the E- and Z-isomers generated upon equilibration under selected light frequencies (490 nm, 410 nm, 365 nm) of the compounds **1**, **2** and **6**, compared with the same parameter determined for HPLC-purified isomers. For the compound **1**, three situations of potential applicability have been additionally visualized with indicated difference in the respective activity – irreversible photouncage-like activation, reversible switching with 490/365 nm light, and reversible switching with 490/410 nm light. Data were plotted against the log of agonist concentration (log_{10}([agonist]) (M)) with mean and SD values in GraphPad Prism. Number of independent experiments (six technical replicates per test): Non-irradiated see Supplementary Figure 1. Irradiated N = 1.*

Supplementary Table 2: Viability assays of photoisomer mixtures. Data were processed using GraphPad Prism.

Compound	IC_{50}: PSS 490 nm (nM) [95% profile likelihood]	IC_{50}: PSS 410 nm (nM) [95% profile likelihood]	IC_{50}: PSS 365 nm (nM) [95% profile likelihood]
**1**	1.08<br>[0.66 to 1.8]<br>(15% *E*-**1**)	12.2<br>[8.72 to 16.0]<br>(56% *E*-**1**)	44.9<br>[25.5 to 73.3]<br>(62% *E*-**1**)
**2**	29<br>[22 to 40]<br>(50% *E*-**2**)	-	-
**6**	0.34<br>[0.24 to 0.49]<br>(14% *E*-**6**)	12.4<br>[10.6 to 14.6]<br>(52% *E*-**6**)	19.3<br>[16.1 to 23.1]<br>(63% *E*-**6**)

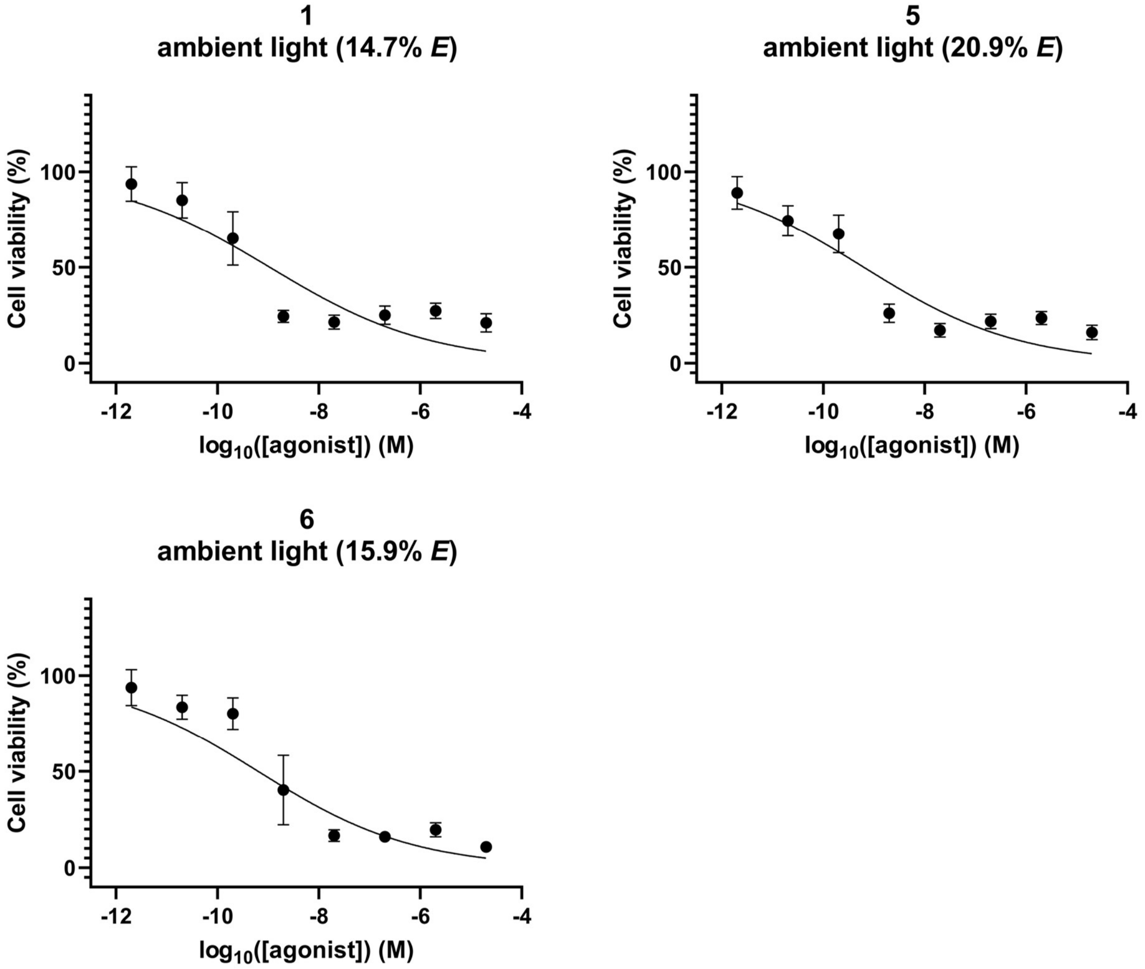

*Supplementary Figure 3: Viability of HT-29 cells after treatment with the mixtures of the respective photoisomers of **1**, **5** and **6** for 48 hours. The mixtures were obtained after incubation of DMSO solutions under ambient light for 24 hours. Data were plotted against the log of agonist concentration (log_{10}([agonist]) (M)) with mean and SD values in GraphPad Prism. Number of independent experiments (six technical replicates per test): **1** N = 3, **5** N = 3, **6** N = 2.*

*Supplementary Table 3: Viability assays of samples exposed to ambient light. The IC_{50} values determined via viability assays with HT-29 cells after treatment with the isolated photoisomers of **1**, **5** and **6** for 48 hours, as well as the results of the same procedure for samples of HPLC-purified Z-isomers exhibited on daylight for one day before the assay (where the content of E-isomer has been determined by analytical HPLC). However, incubation for further two days at daylight almost doubled the amount of E-isomer (IC_{50} values were not determined for these samples). These data are compared with the values reported in literature (with the unknown final Z/E-isomer ratio).[1] Data were processed using GraphPad Prism.*

Compound	IC_{50}: *Z*-isomer (nM) [95% profile likelihood]	IC_{50}: *E*-isomer (nM) [95% profile likelihood]	IC_{50}: 1 day daylight (nM) [95% profile likelihood]	IC_{50}: 3 days daylight (nM) [95% profile likelihood]	IC_{50}: literature report (nM)[1]
**1**	0.47 [0.35 to 0.62]	92 [80 to 100]	1.08 [0.66 to 1.8] (15% *E*-**1**)	n.d. (32% *E*-**1**)	14.9 ± 3.8
**5**	0.09 [0.05 to 0.15]	471 [352 to 636]	1.46 [0.96 to 2.26] (21% *E*-**5**)	n.d. (34% *E*-**5**)	26.1 ± 8.5
**6**	0.21 [0.16 to 0.28]	618 [487 to 788]	0.63 [0.43 to 0.94] (16% *E*-**6**)	n.d. (30% *E*-**6**)	45.4 ± 12

Irradiation Intensities of LEDs

Supplementary Table 4: The LEDs applied to irradiate the photoswitchable compounds were characterized using the PowerMax USB (type PS19Q) sensor device (Coherent®).

λ_{max} of the LED diode [nm]	Max Power [mW/cm^2]	Max mean power measured [W]	Min Power [mW/cm^2]	Min mean power measured [W]
365 nm	-	-	5,61E-01	1,59E-03
380 nm	8,67E-02	2,46E-04	-	-
407 nm	9,39E+01	2,66E-01	8,59E+01	2,43E-01
430 nm	1,85E+01	5,23E-02	1,19E+01	3,38E-02
450 nm	1,13E+00	3,21E-03	8,86E-01	2,51E-03
470 nm	1,69E+01	4,80E-02	1,59E+01	4,52E-02
490 nm	7,64E+00	2,17E-02	4,98E+00	1,41E-02
523 nm	7,08E+00	2,01E-02	6,56E+00	1,86E-02

Absorption Spectra

Photostationary States: Plinabulin Derivatives

Compounds **1-6** (respectively) were dissolved in a saturated solution of sodium ascorbate in DMSO (80 µM). Absorption spectra (d=2 mm) were first measured in the dark. The samples were then first irradiated with 407 nm (2 min), then with 490 nm (15 min) and an absorption spectrum was measured after each irradiation step. Additional spectra of **1** were depicted in Fig. 1. The spectrum of **4** with an inset was depicted in Fig. 3. The spectra of compounds **1** - **6** are depicted below.

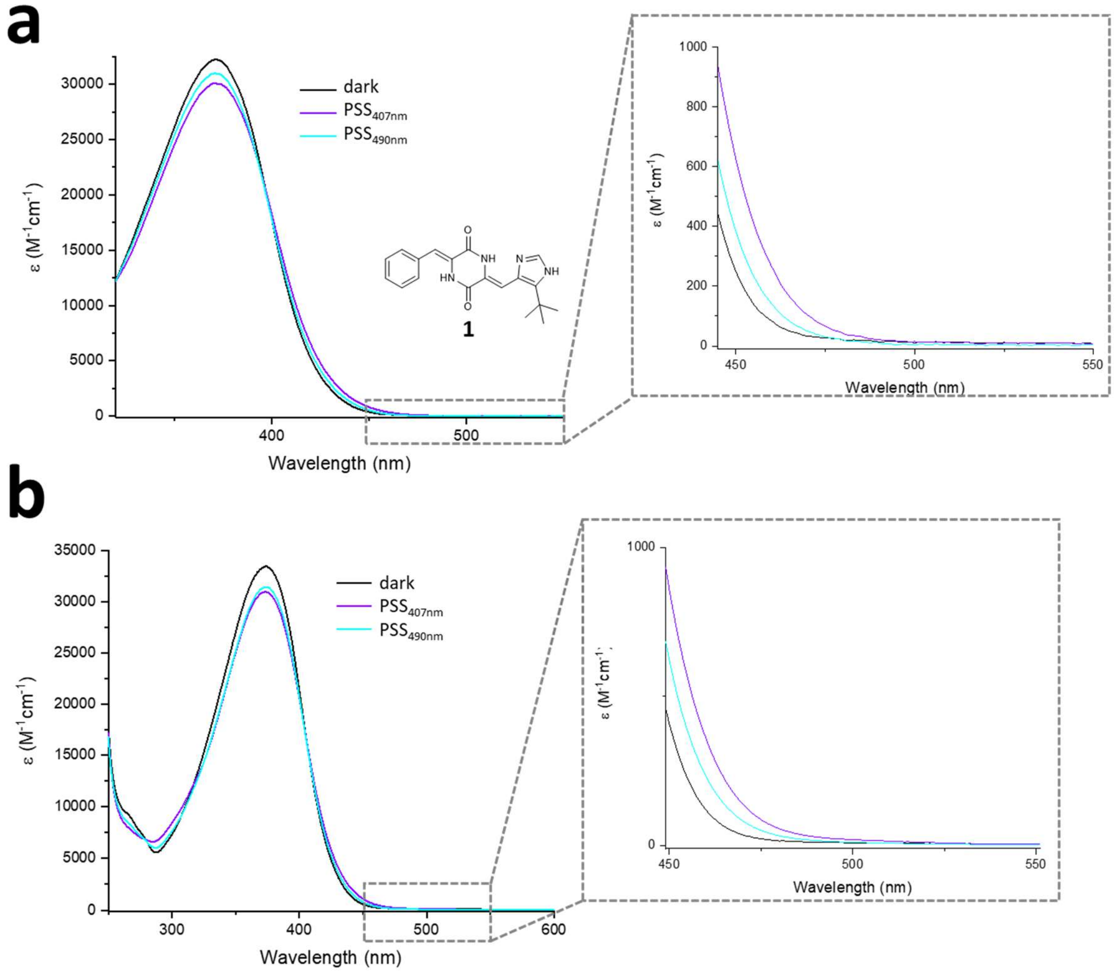

*Supplementary Figure 4: Plinabulin (**1**) – photochromism in organic and aqueous solutions.(**a**) UV-Vis spectra of Plinabulin 1 in a saturated solution of ascorbic acid in DMSO (80 µM). (**b**) In an additional experiment, the UV-Vis spectra (d=2 mm) of a solution of 2.0 mM (cutout) and 400 µM (full spectrum) compound 1 with 10 mM glutathione and 5.0 mM tris(2-carboxyethyl)phosphine in 25% PBS in DMSO were first measured in the dark. The samples were then first irradiated with 407 nm (2 min), then with 490 nm (15 min) and an UV-Vis spectrum was measured after each irradiation step.*

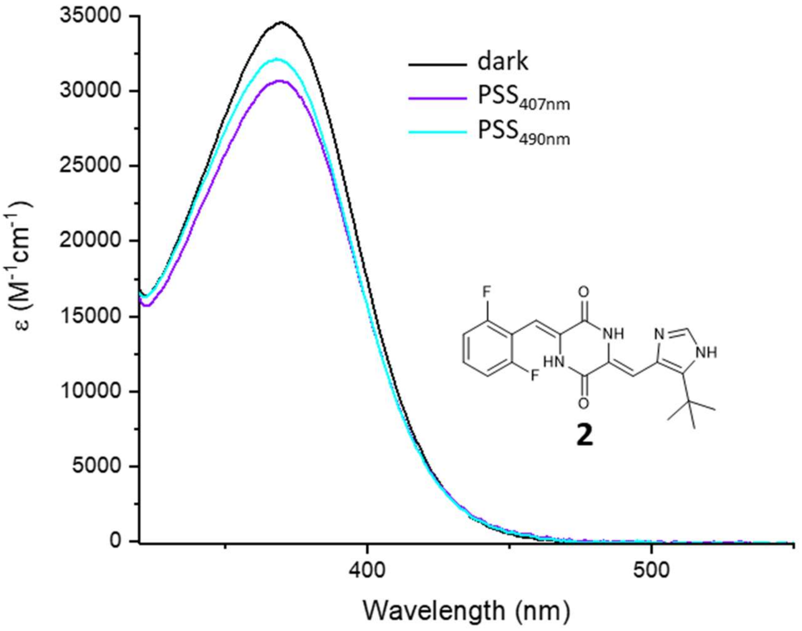

*Supplementary Figure 5: Absorption spectra of compound **2** (80 µM) in saturated solution of sodium ascorbate in DMSO before and after irradiation.*

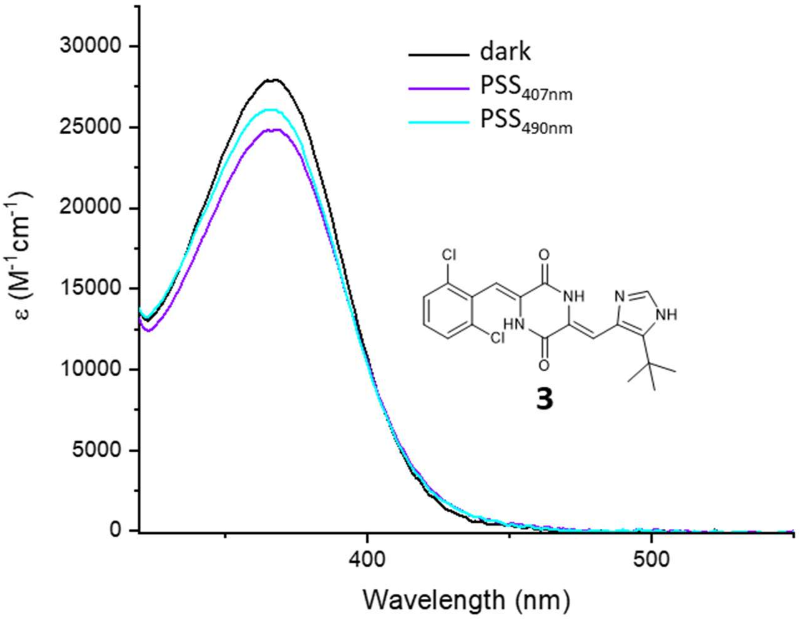

*Supplementary Figure 6: Absorption spectra of compound **3** (80 µM) in saturated solution of sodium ascorbate in DMSO before and after irradiation.*

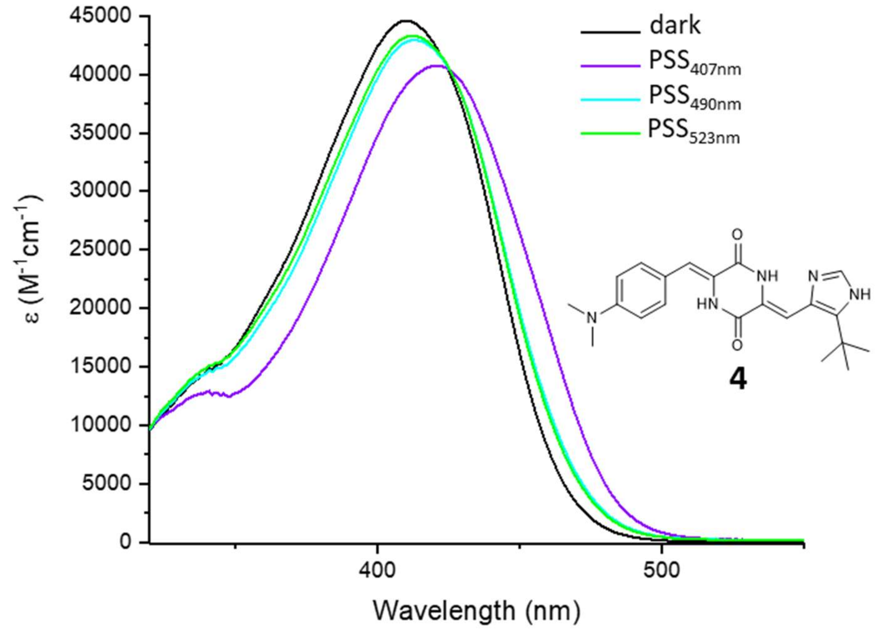

Supplementary Figure 7: Absorption spectra of compound ***4*** *(80 µM) in saturated solution of sodium ascorbate in DMSO before and after irradiation.*

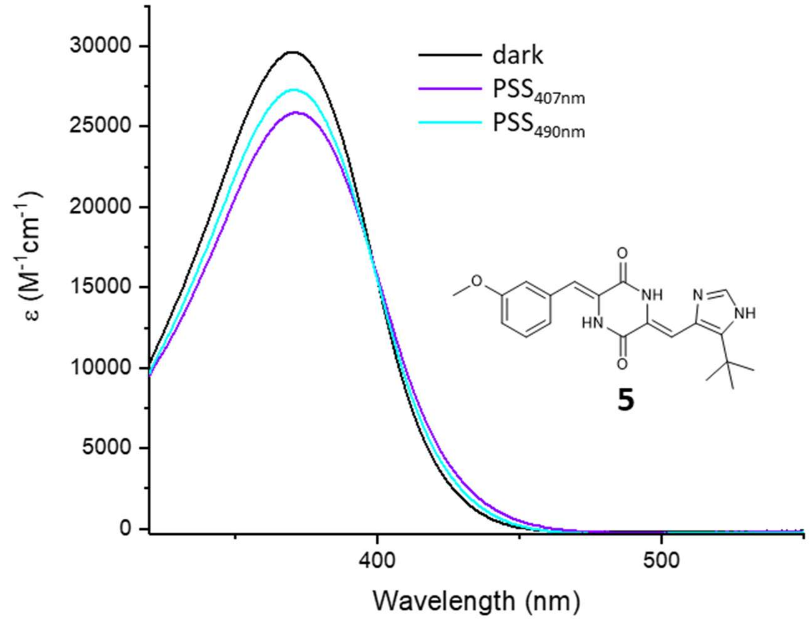

Supplementary Figure 8: Absorption spectra of compound ***5*** *(80 µM) in saturated solution of sodium ascorbate in DMSO before and after irradiation.*

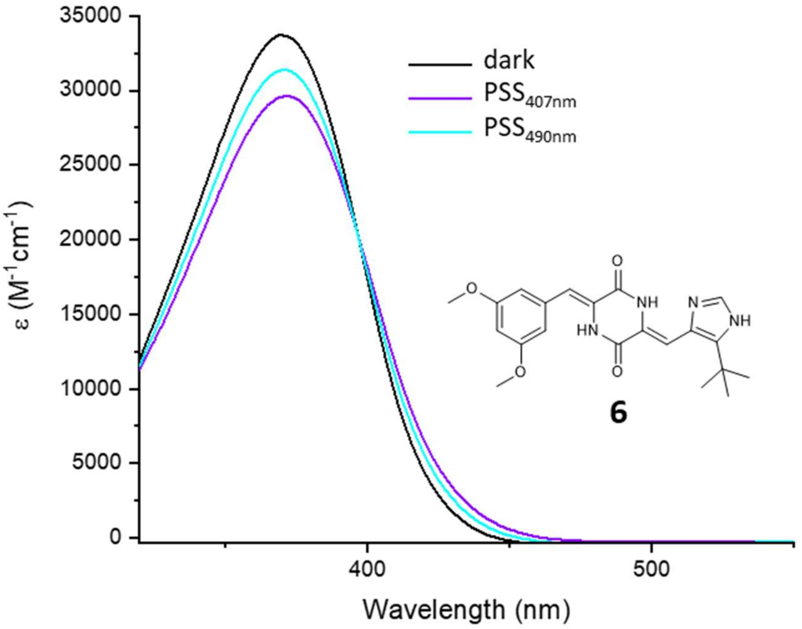

*Supplementary Figure 9: Absorption spectra of compound **6** (80 µM) in saturated solution of sodium ascorbate in DMSO before and after irradiation.*

Photostationary States: Hemipiperazines in CH_2Cl_2

Compounds **7-10** and **13** (respectively) were dissolved in CH_2Cl_2 (80 µM). Absorption spectra (d=10 mm) were first measured in the dark. The samples were then irradiated with 365 nm and with 407 nm. An absorption spectrum was measured after each irradiation step until the PSS was reached, respectively. The samples of compounds **8** und **10** were additionally irradiated with 430 nm.

It was observed that irradiation of solutions of compounds **11** and **12** in CH_2Cl_2 lead not only to photoisomerization but also degradation. Therefore, the corresponding spectra were measured in DMSO (see following subchapter).

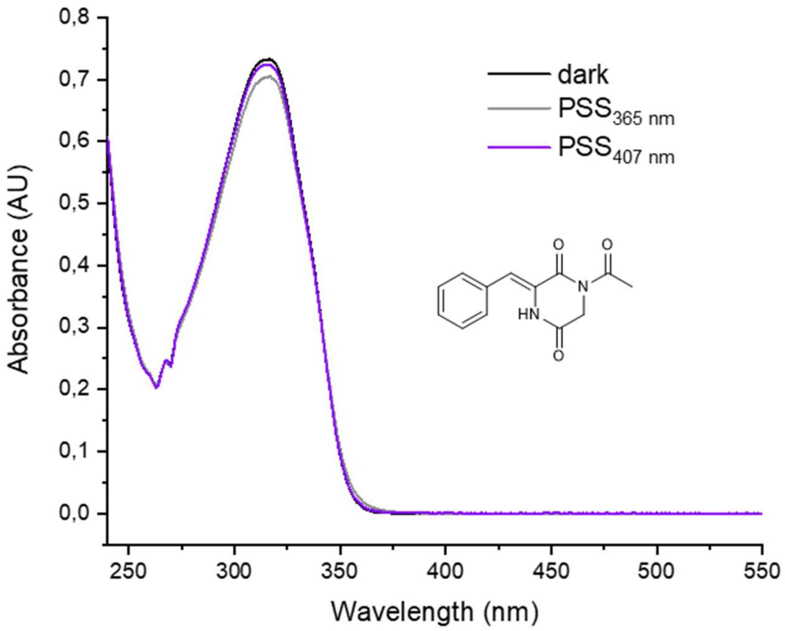

*Supplementary Figure 10: Absorption spectra of compound **7** (80 µM) in CH_2Cl_2 before and after irradiation.*

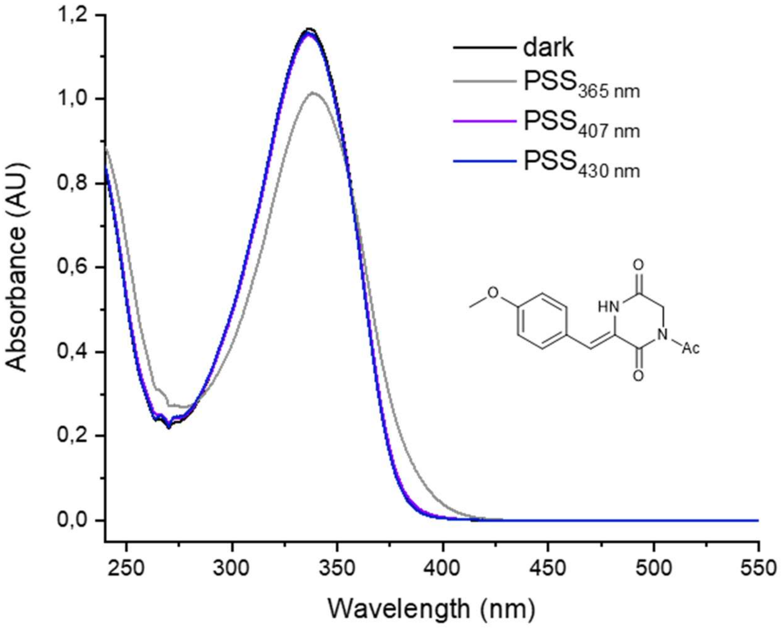

*Supplementary Figure 11: Absorption spectra of compound **8** (80 µM) in CH_2Cl_2 before and after irradiation.*

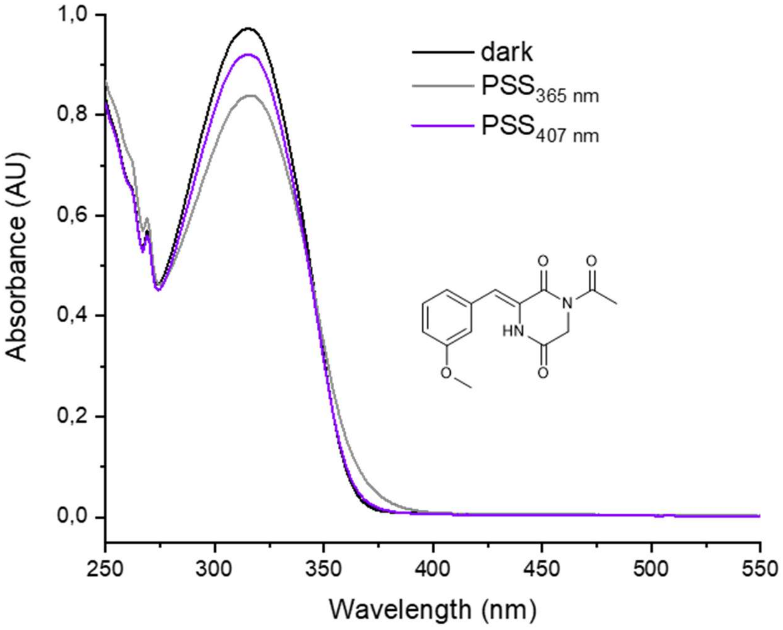

*Supplementary Figure 12: Absorption spectra of compound **9** (80 µM) in CH_2Cl_2 before and after irradiation.*

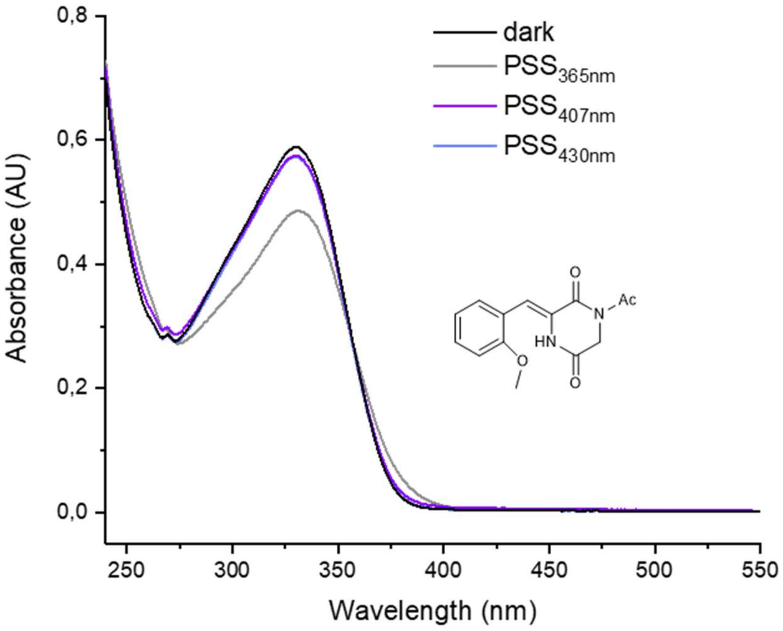

*Supplementary Figure 13: Absorption spectra of compound **10** (80 µM) in CH_2Cl_2 before and after irradiation.*

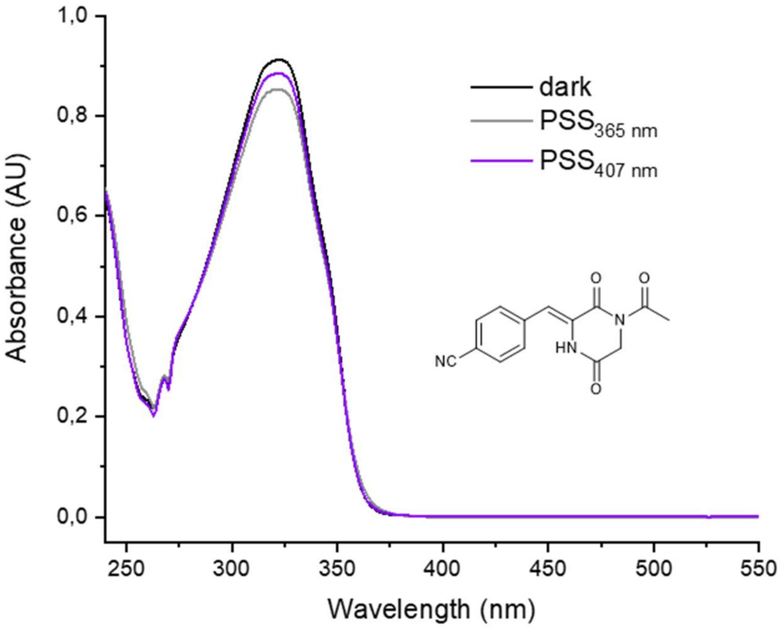

*Supplementary Figure 14: Absorption spectra of compound **13** (80 µM) in CH_2Cl_2 before and after irradiation.*

Photostationary States: Hemipiperazines in DMSO and PBS

Compounds **7**, **11** and **12** (respectively) were dissolved in DMSO (80 µM) or mixtures of DMSO and PBS buffer. Absorption spectra (d=10 mm) were first measured in the dark. The samples were then irradiated with selected wavelengths and an absorption spectrum was measured after each irradiation step until the PSS was reached, respectively.

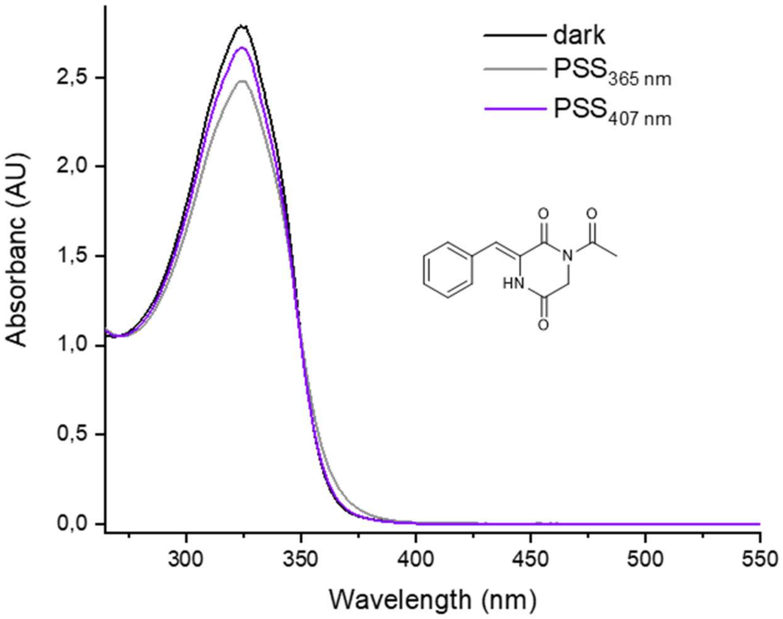

*Supplementary Figure 15: Absorption spectra of compound **7** (80 µM) in DMSO before and after irradiation.*

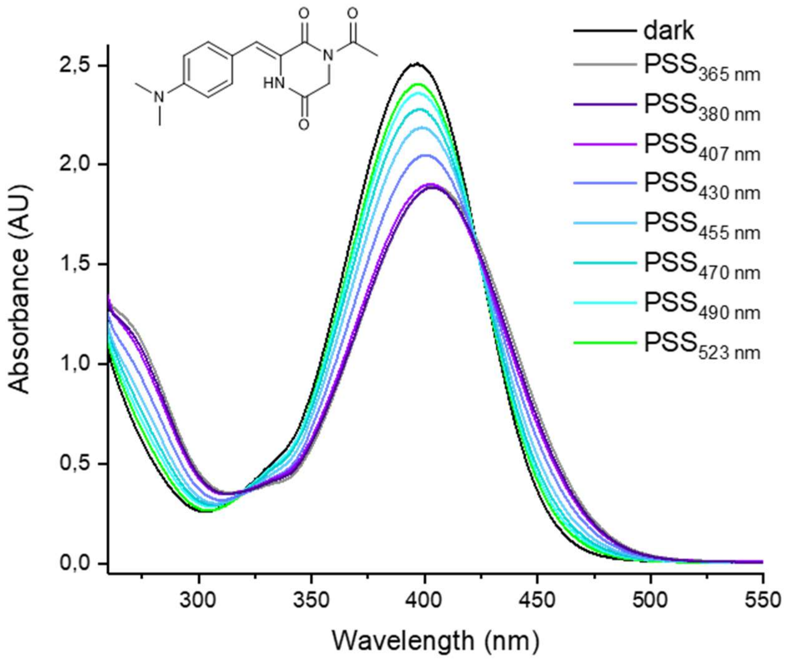

*Supplementary Figure 16: Absorption spectra of compound **11** (80 µM) in DMSO before and after irradiation.*

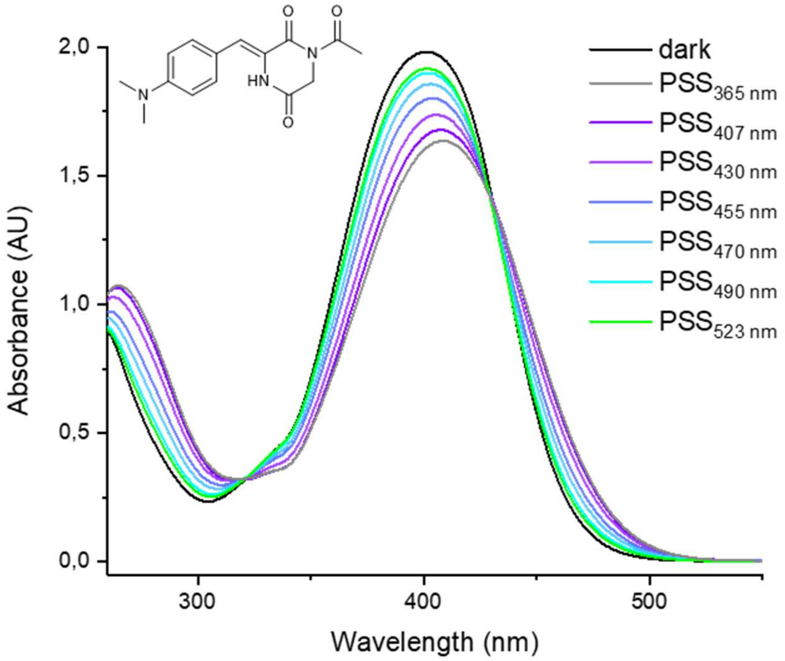

*Supplementary Figure 17: Absorption spectra of compound **11** (80 µM) in 25% PBS in DMSO (v/v) before and after irradiation.*

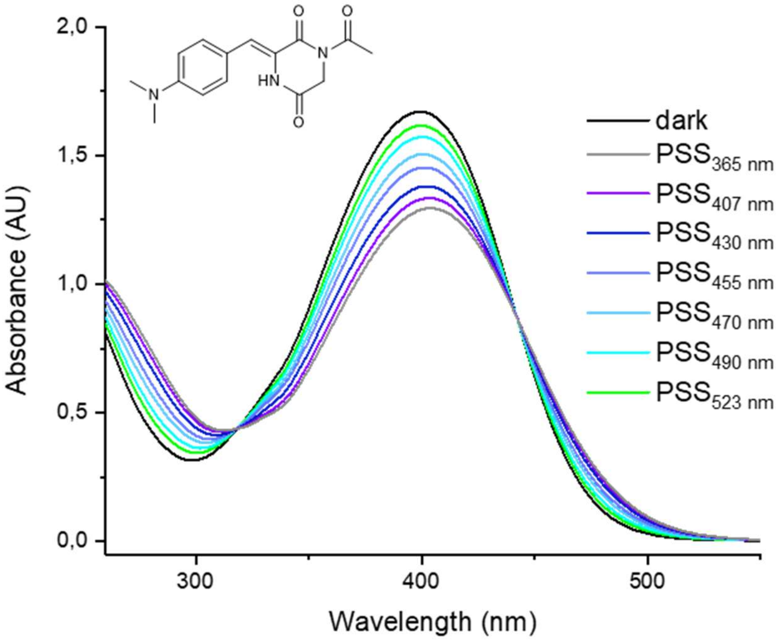

*Supplementary Figure 18: Absorption spectra of compound **11** (80 µM) in 75% PBS in DMSO (v/v) before and after irradiation.*

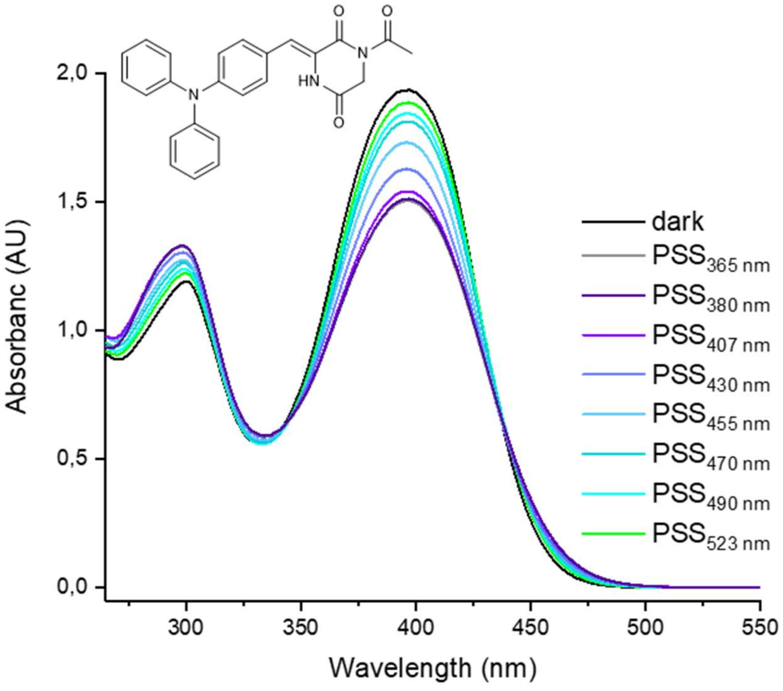

*Supplementary Figure 19: Absorption spectra of compound **12** (80 µM) in DMSO (v/v) before and after irradiation.*

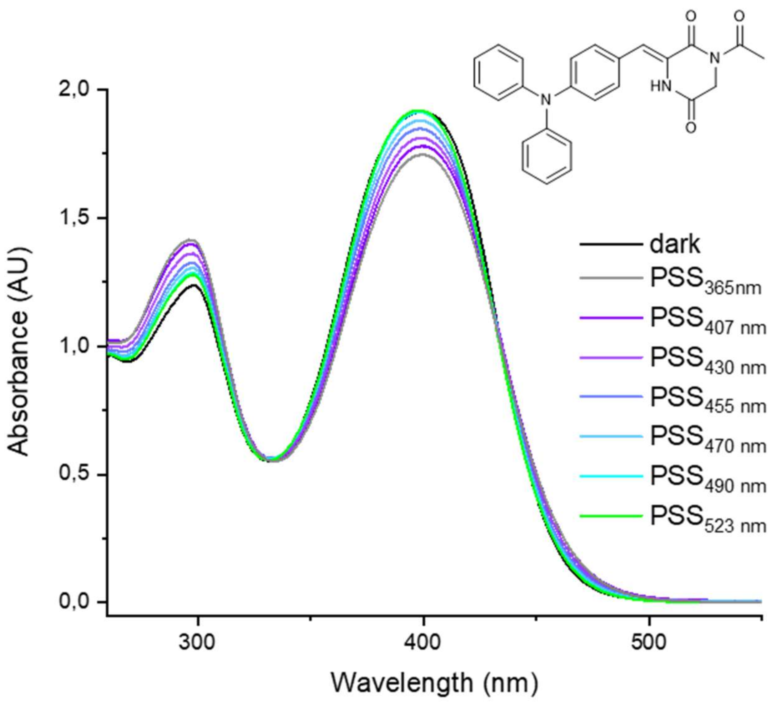

*Supplementary Figure 20: Absorption spectra of compound **12** (80 µM) in 25% PBS in DMSO (v/v) before and after irradiation.*

Photostationary States: "Locked" Plinabulin

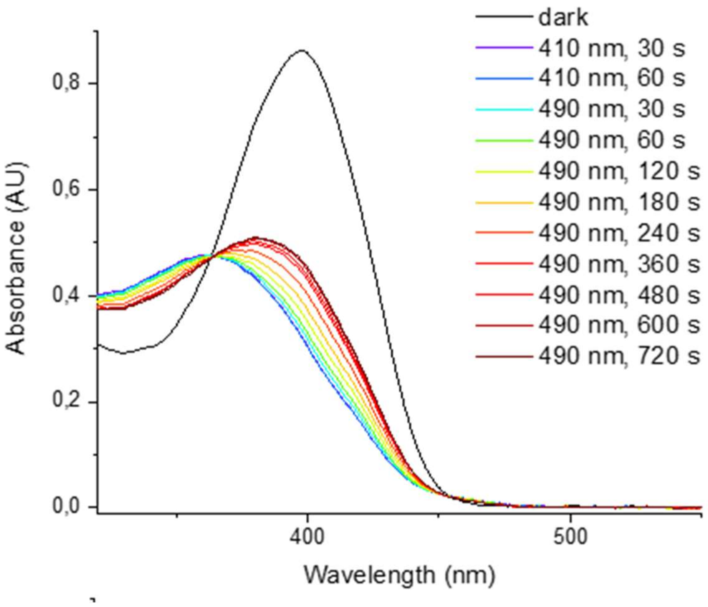

*Supplementary Figure 21: Compound **15** in saturated solution of ascorbic acid in DMSO (200 µM) was irradiated and an absorption spectrum (d = 2 mm) was measured after each irradiation step until the PSS was reached, respectively.*

Solvatochromism and Acidochromism

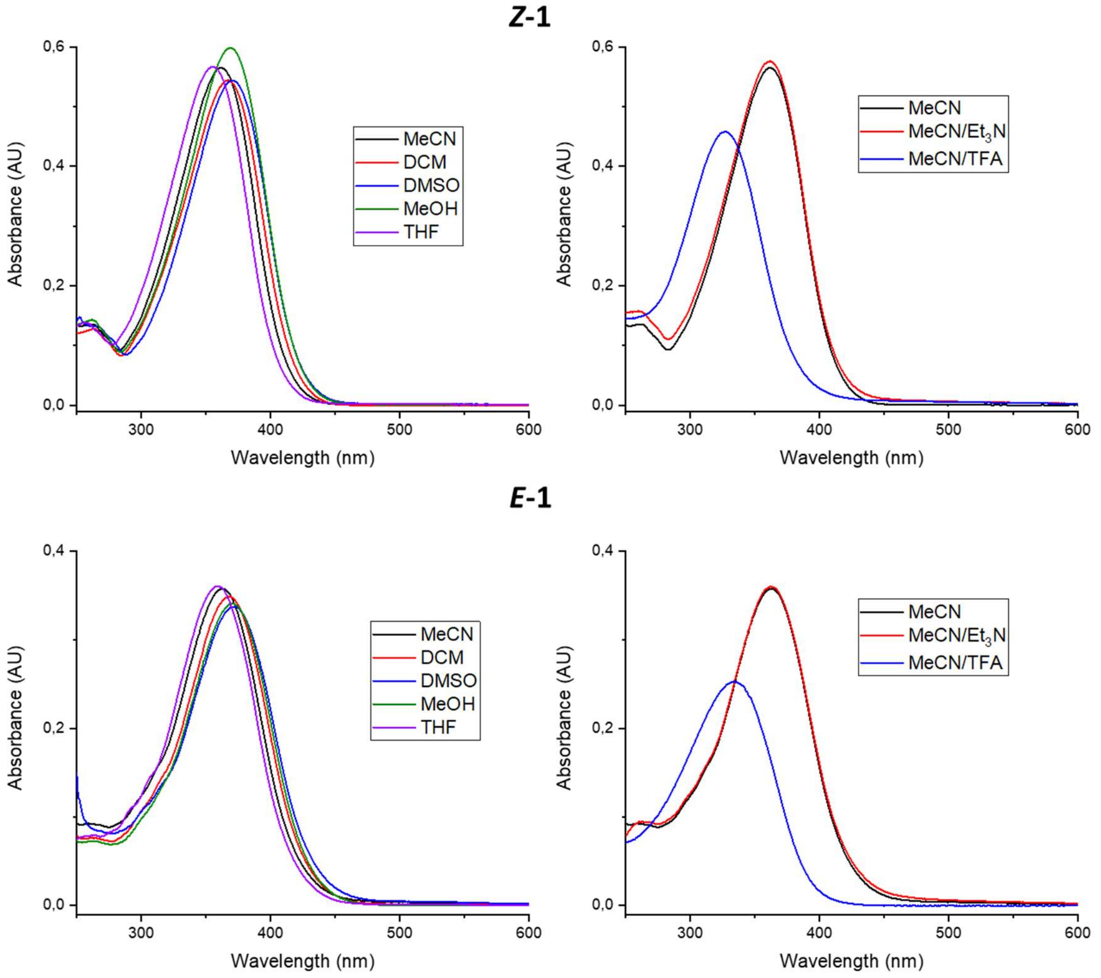

*Supplementary Figure 22: The photoisomers Z-**1** and E-**1** were dissolved in different solvents (80 µM) and in mixtures of 1% (v/v) TFA in MeCN and 1% (v/v) Et_3N in MeCN, respectively. Absorption spectra were measured in the dark (d = 2 mm). The spectra are assigned to the respective solvents via color code.*

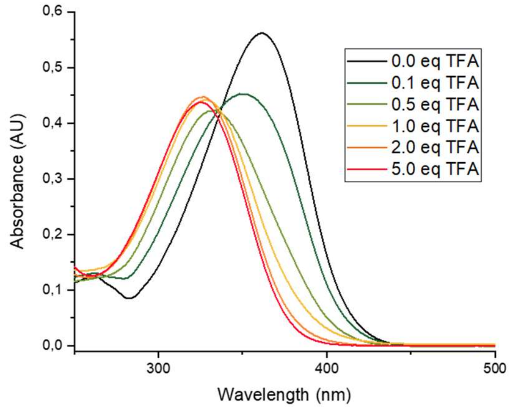

*Supplementary Figure 23: Absorption spectra of 80 µM solutions (d=2 mm) of Z-**1** in MeCN with different amounts of TFA.*

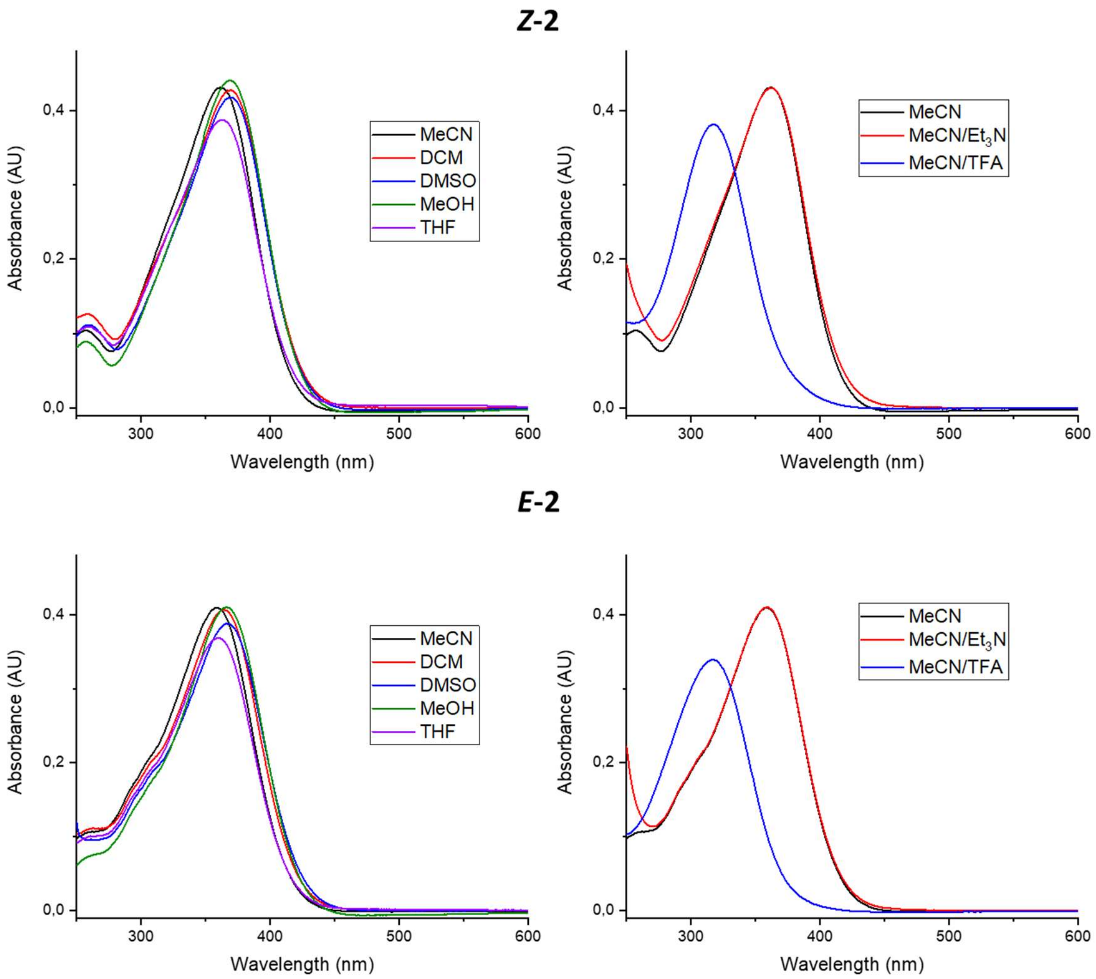

*Supplementary Figure 24: The photoisomers Z-**2** and E-**2** were dissolved in different solvents (80 µM) and in mixtures of 1% (v/v) TFA in MeCN and 1% (v/v) Et_3N in MeCN, respectively. Absorption spectra were measured in the dark (d = 2 mm). The spectra are assigned to the respective solvents via color code.*

***Z*-3**

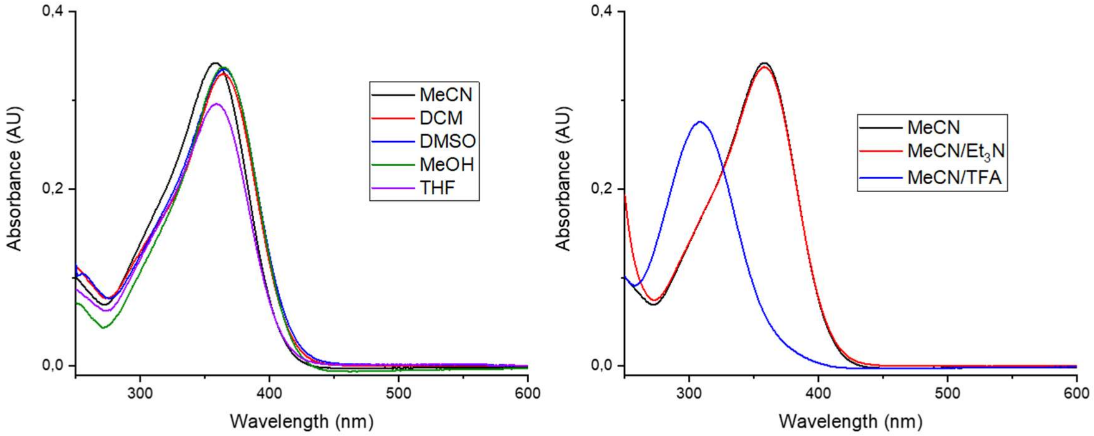

***E*-3**

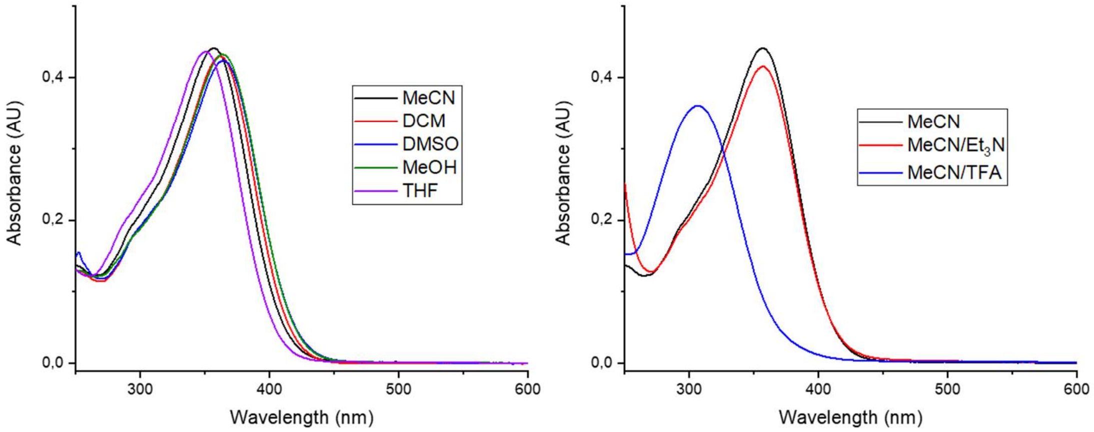

*Supplementary Figure 25: The photoisomers Z-**3** and E-**3** were dissolved in different solvents (80 µM) and in mixtures of 1% (v/v) TFA in MeCN and 1% (v/v) Et_3N in MeCN, respectively. Absorption spectra were measured in the dark (d = 2 mm). The spectra are assigned to the respective solvents via color code.*

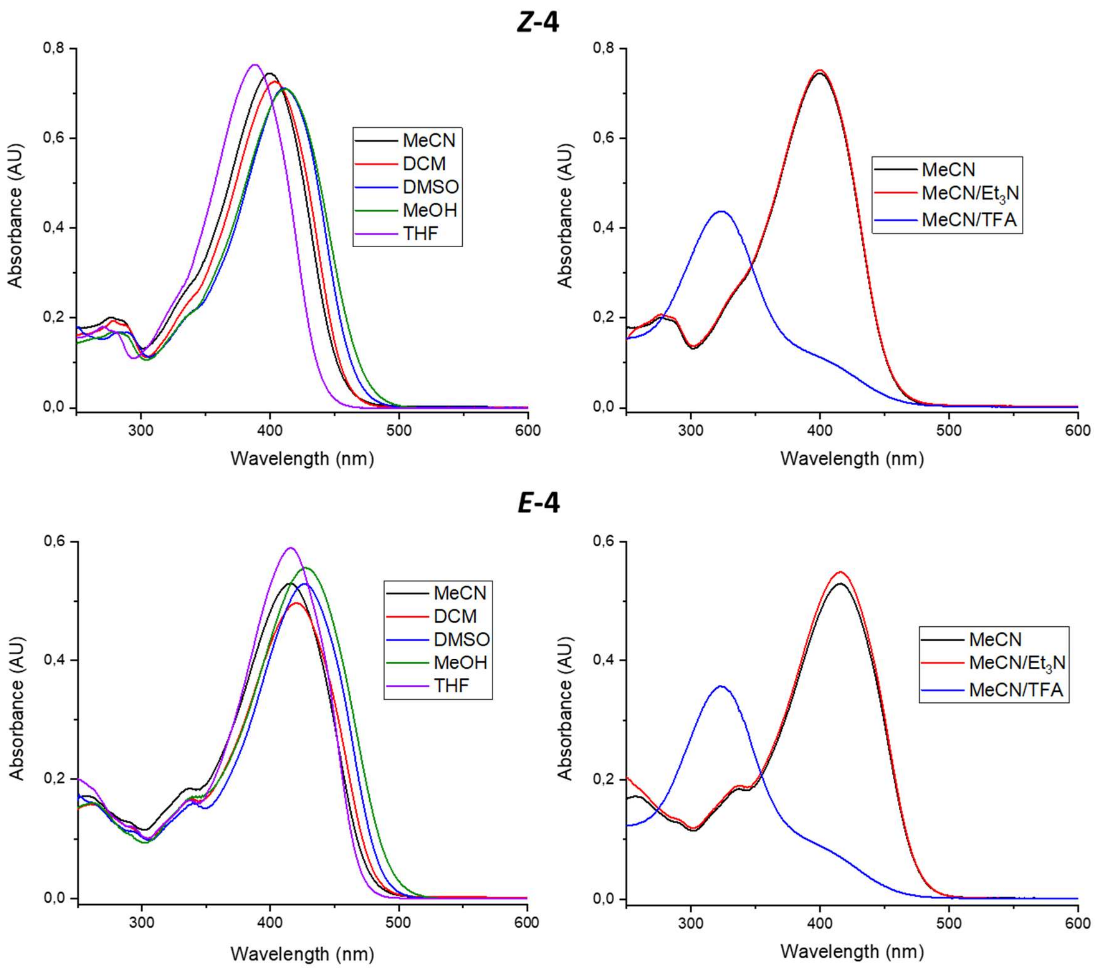

*Supplementary Figure 26: The photoisomers Z-**4** and E-**4** were dissolved in different solvents (80 µM) and in mixtures of 1% (v/v) TFA in MeCN and 1% (v/v) Et_3N in MeCN, respectively. Absorption spectra were measured in the dark (d = 2 mm). The spectra are assigned to the respective solvents via color code.*

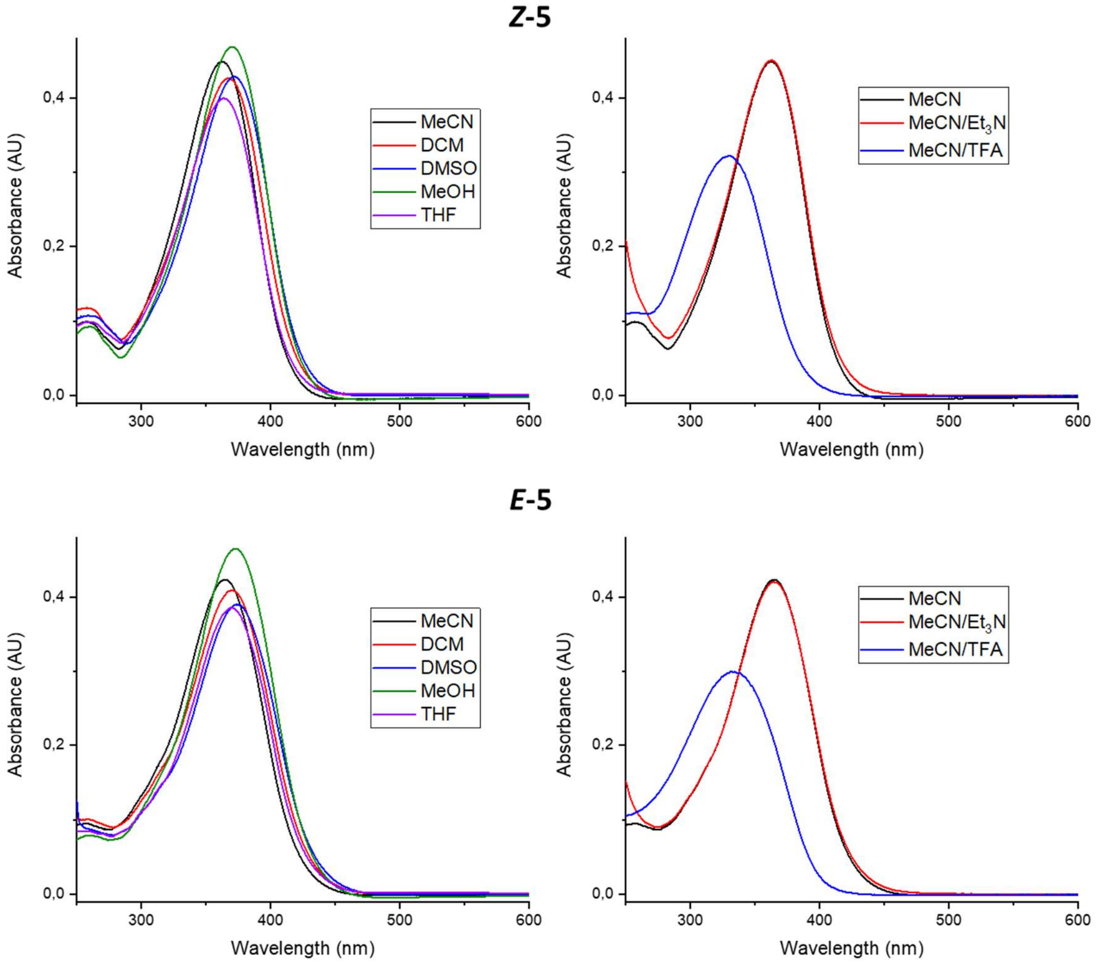

*Supplementary Figure 27: The photoisomers Z-**5** and E-**5** were dissolved in different solvents (80 µM) and in mixtures of 1% (v/v) TFA in MeCN and 1% (v/v) Et_3N in MeCN, respectively. Absorption spectra were measured in the dark (d = 2 mm). The spectra are assigned to the respective solvents via color code.*

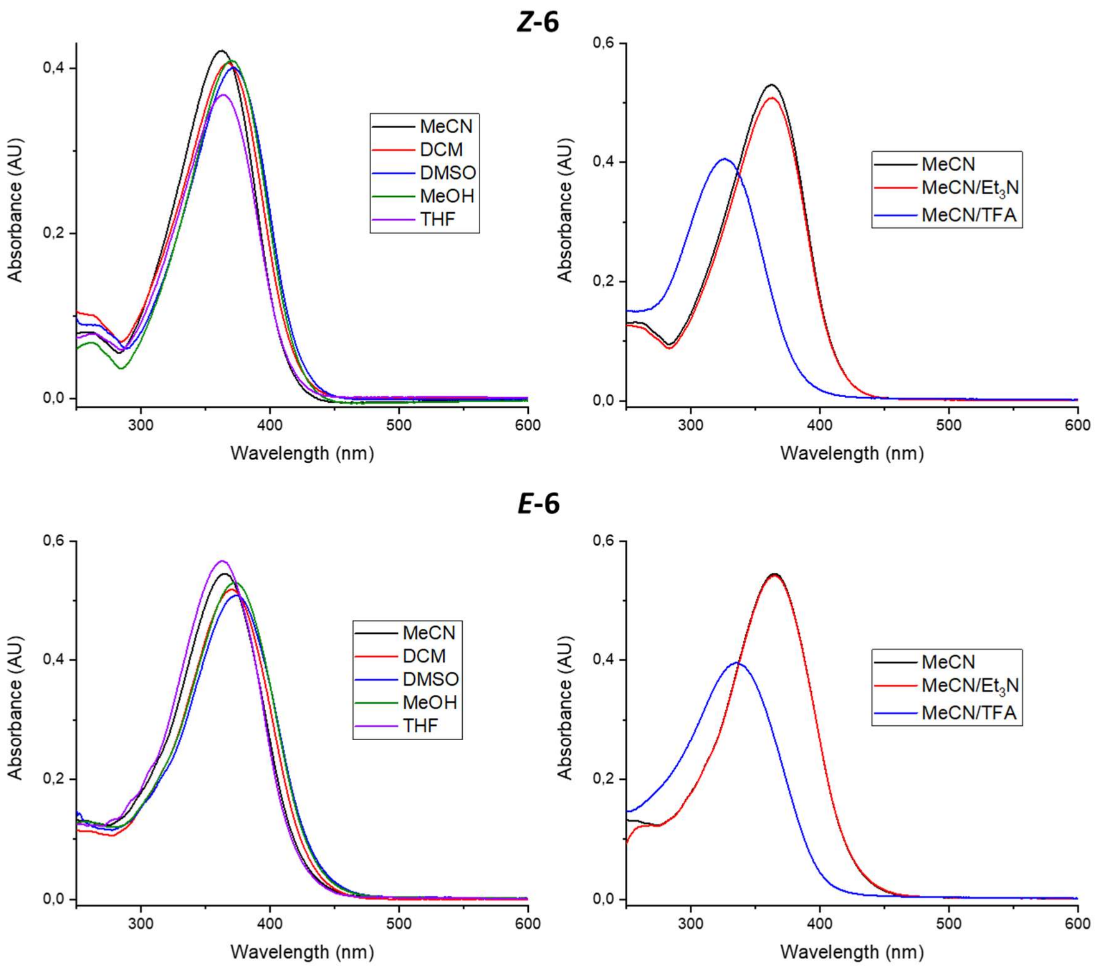

*Supplementary Figure 28: The photoisomers Z-**6** and E-**6** were dissolved in different solvents (80 µM) and in mixtures of 1% (v/v) TFA in MeCN and 1% (v/v) Et_3N in MeCN, respectively. Absorption spectra were measured in the dark (d = 2 mm). The spectra are assigned to the respective solvents via color code.*

***Z*-7**

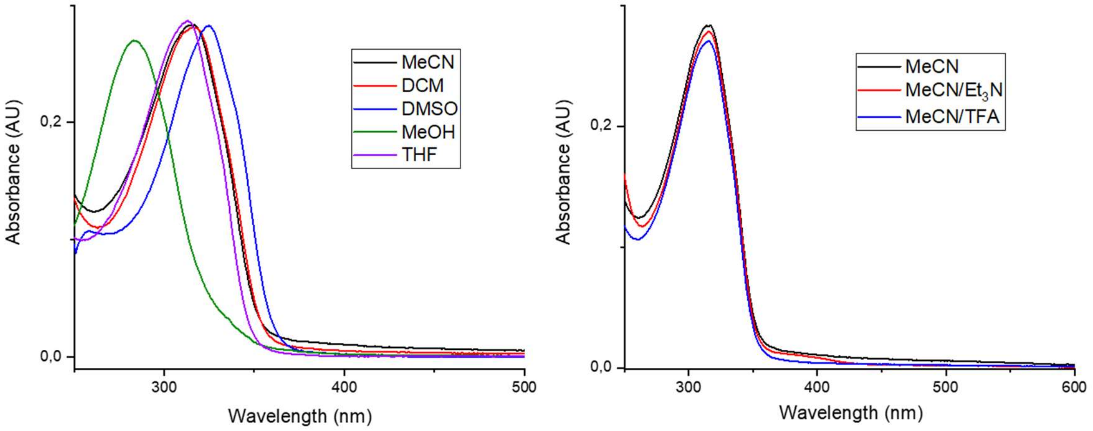

Supplementary Figure 29: The Z-isomer of ***7*** *was dissolved in different solvents (80 µM) and in mixtures of 1% (v/v) TFA in MeCN and 1% (v/v) Et_3N in MeCN, respectively. Absorption spectra were measured in the dark (d = 2 mm). The spectra are assigned to the respective solvents via color code.*

***Z*-14**

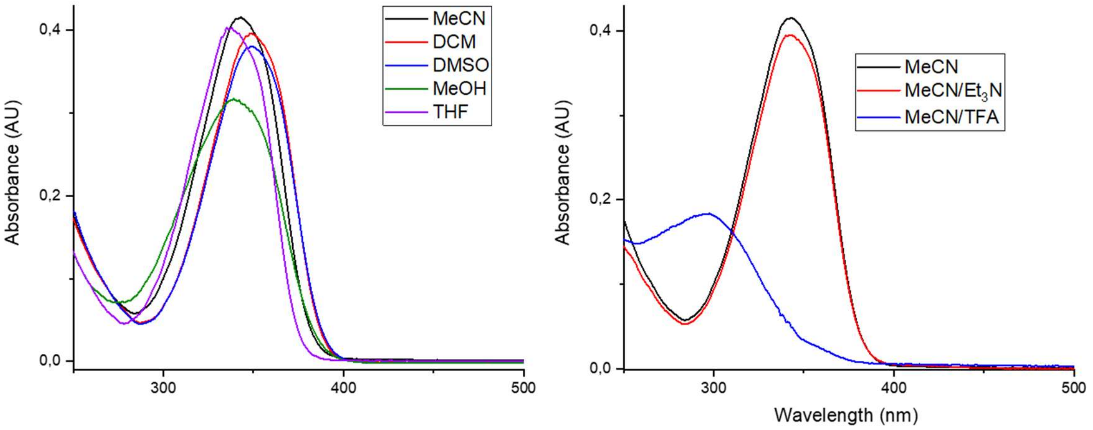

Supplementary Figure 30: The Z-isomer of ***14*** *was dissolved in different solvents (80 µM) and in mixtures of 1% (v/v) TFA in MeCN and 1% (v/v) Et_3N in MeCN, respectively. Absorption spectra were measured in the dark (d = 2 mm). The spectra are assigned to the respective solvents via color code.*

Fluorescence Spectra

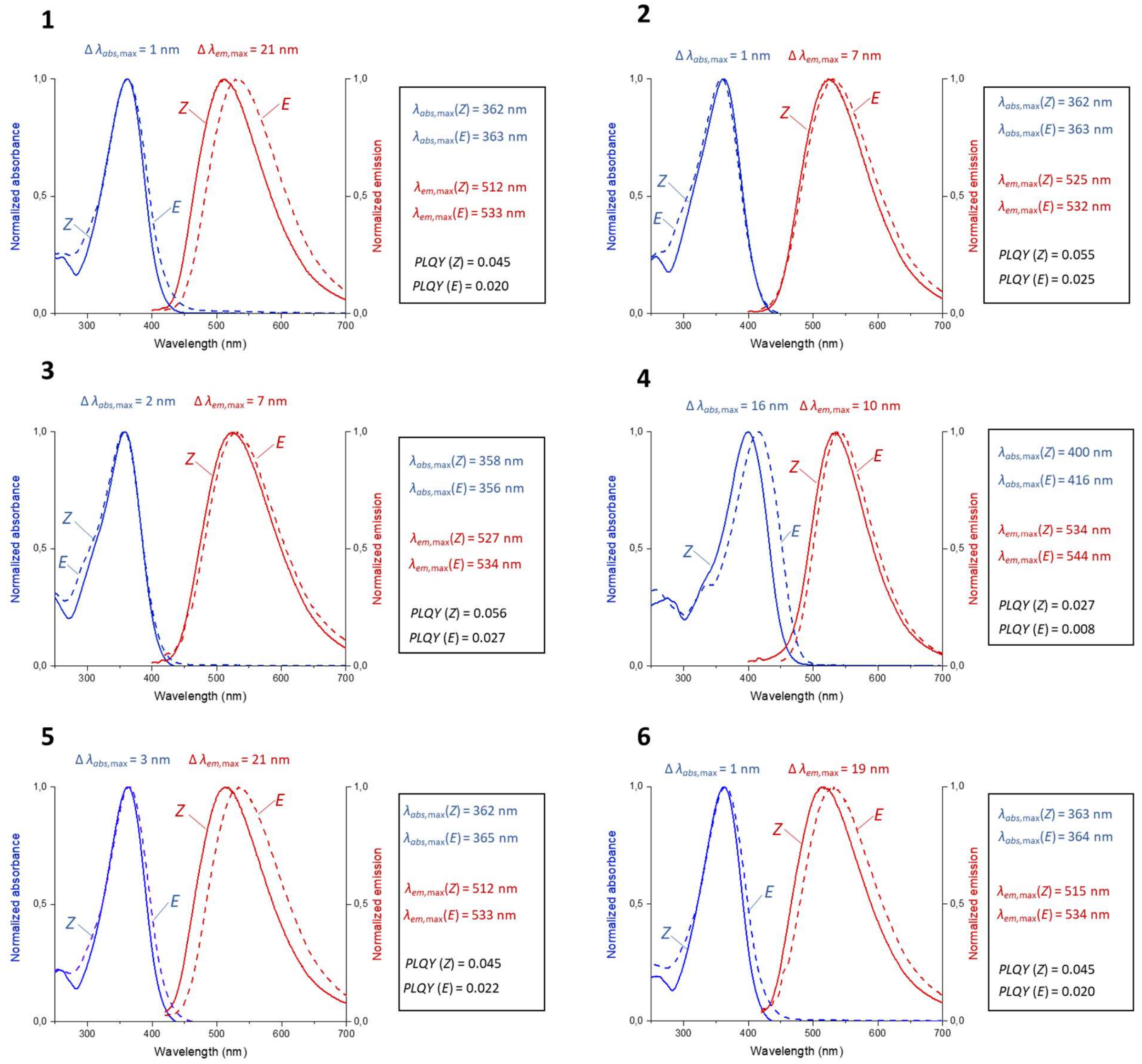

*Supplementary Figure 31: Normalized absorption (blue) and fluorescence (red) spectra of 80 µM solutions of the respective Z-isomers (solid line) and E-isomers (dashed line) of **1**-**6** in MeCN.*

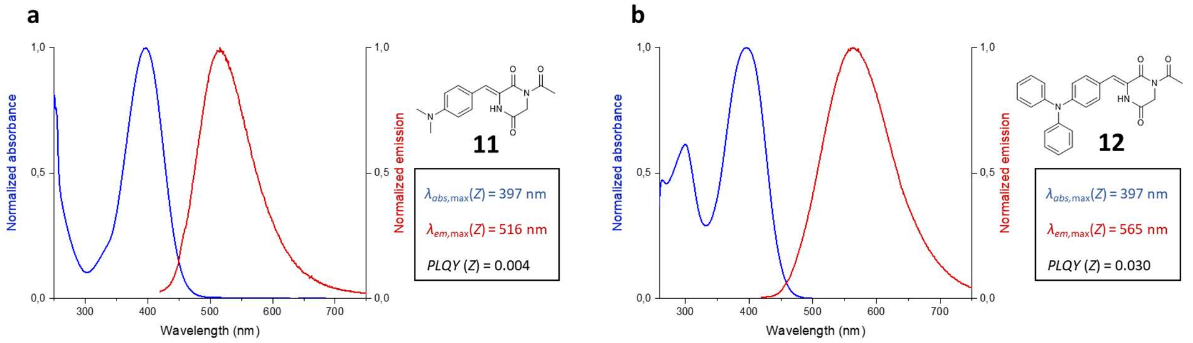

*Supplementary Figure 32: Normalized absorption (blue) and fluorescence (red) spectra of 80 µM solutions of the respective Z-isomers of **11** (a) and **12** (b) in DMSO.*

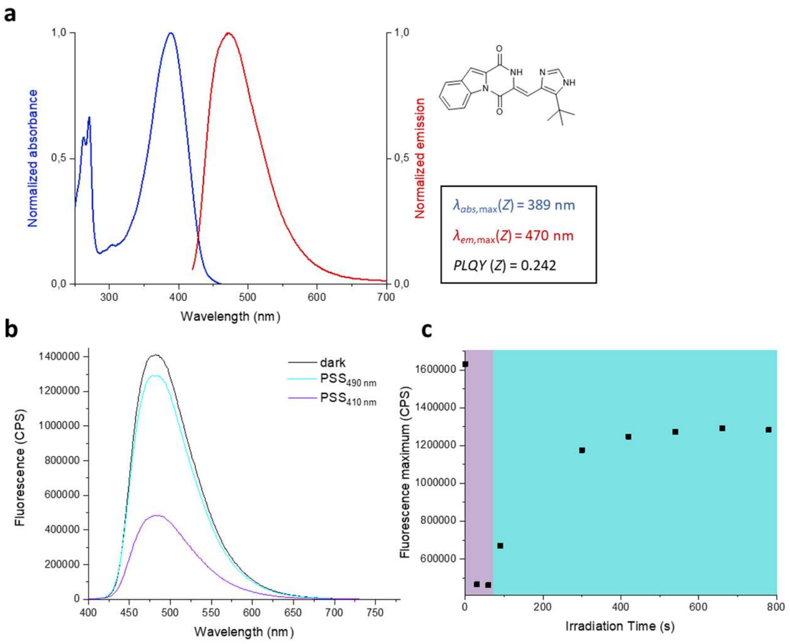

*Supplementary Figure 33: (a) Normalized absorption (blue) and fluorescence (red) spectra of 80 µM solutions of Z-**15** in DMSO. (b) Fluorescence spectra of compound **15** (200 µM) in saturated solution of ascorbic acid in DMSO before and after irradiation (excitation at 380 nm) The spectra are assigned to the respective mixture via color code. (c) Compound **15** (200 µM) in saturated solution of ascorbic acid was analyzed via fluorescence spectroscopy before and after irradiation with first 410 nm and subsequently 490 nm (indicated by the color of the background). The maximal fluorescence was plotted against the irradiation time.*

Determination of PSS Composition *via* NMR Spectroscopy

The PSS composition was determined by measuring ^1H NMR spectra of the respective compounds after irradiation. The PSS values in the NMR tubes (0.5 mL of the 30-60 mM solutions in DMSO-d_6 or CD_2Cl_2) have been achieved within 1 h (365, 407, 523 nm), 2 h (430, 450, 470 nm) or 4 h (380, 490 nm). Signal pairs were determined for each compound: two signals assigned to the same proton for the respective photoisomers were integrated and the % of *E*-isomer = $I_E/(I_E+I_Z)*100\%$ was determined. The final *E*/*Z* ratios result from the average value determined from three signal pairs.

Supplementary Table 5: Collected data from the ^1H NMR determination of the E/Z-isomer ratio of the respective compounds upon irradiation with the indicated wavelengths of light, based on the individually collected data for each of the compounds, depicted on the Supplementary Figures 34-48.

Compound	365 nm (% of *E*-Isomer)	407 nm (% of *E*-Isomer)	430 nm (% of *E*-Isomer)	450 nm (% of *E*-Isomer)	470 nm (% of *E*-Isomer)	490 nm (% of *E*-Isomer)	523 nm (% of *E*-Isomer)
**1**	62	56	42	38	19	13	-
**2**	59	64	62	61	58	56	-
**3**	48	44	41	39	34	29	-
**4**	61	55	46	19	17	12	10
**5**	63	54	42	24	19	16	-
**6**	63	52	39	31	17	11	-
**7**	19	6	-	-	-	-	-
**7***	10	2	-	-	-	-	-
**8***	29	3	<1	-	-	-	-
**9***	31	6	-	-	-	-	-
**10***	34	5	<1	-	-	-	-
**11**	71	62	53	20	13	7	3
**12**	58	54	47	26	19	15	14
**13***	12	4	-	-	-	-	-
**14**	67	<5	-	-	-	-	-

* in CD_2Cl_2, otherwise in DMSO-d_6.

Plinabulin Derivatives

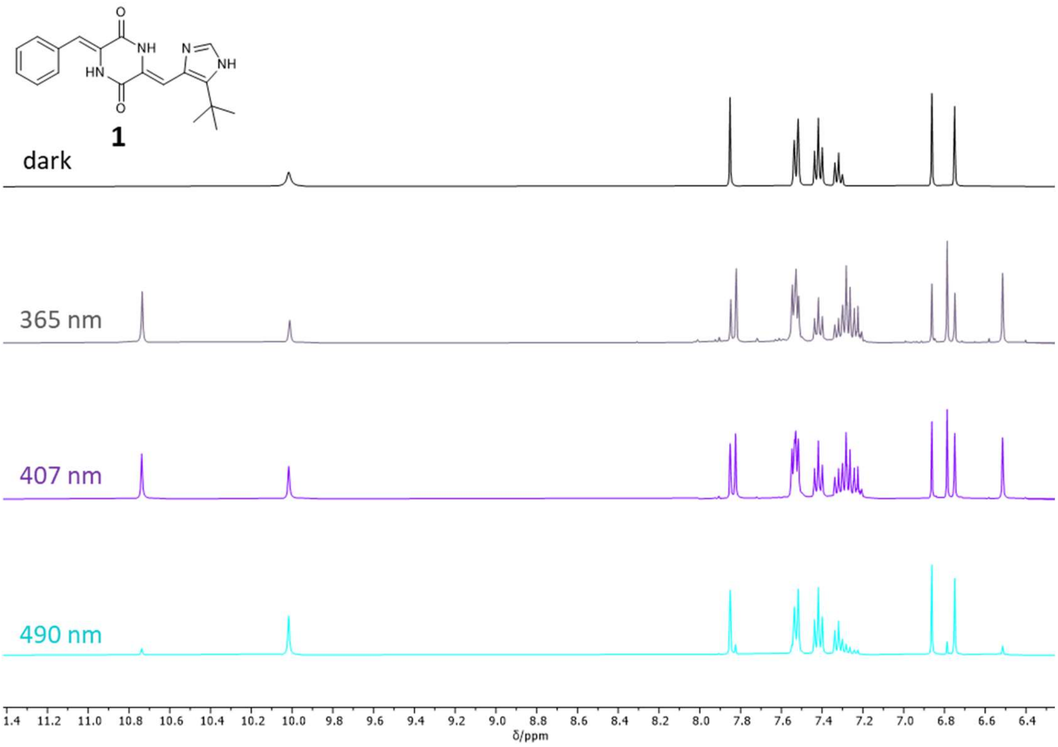

*Supplementary Figure 34: Sections of ^{1}H NMR spectra (400 MHz, DMSO-d_6) of compound **1** in the dark (top) and after reaching the PSS upon irradiation with 365 nm, 407 nm and 490 nm.*

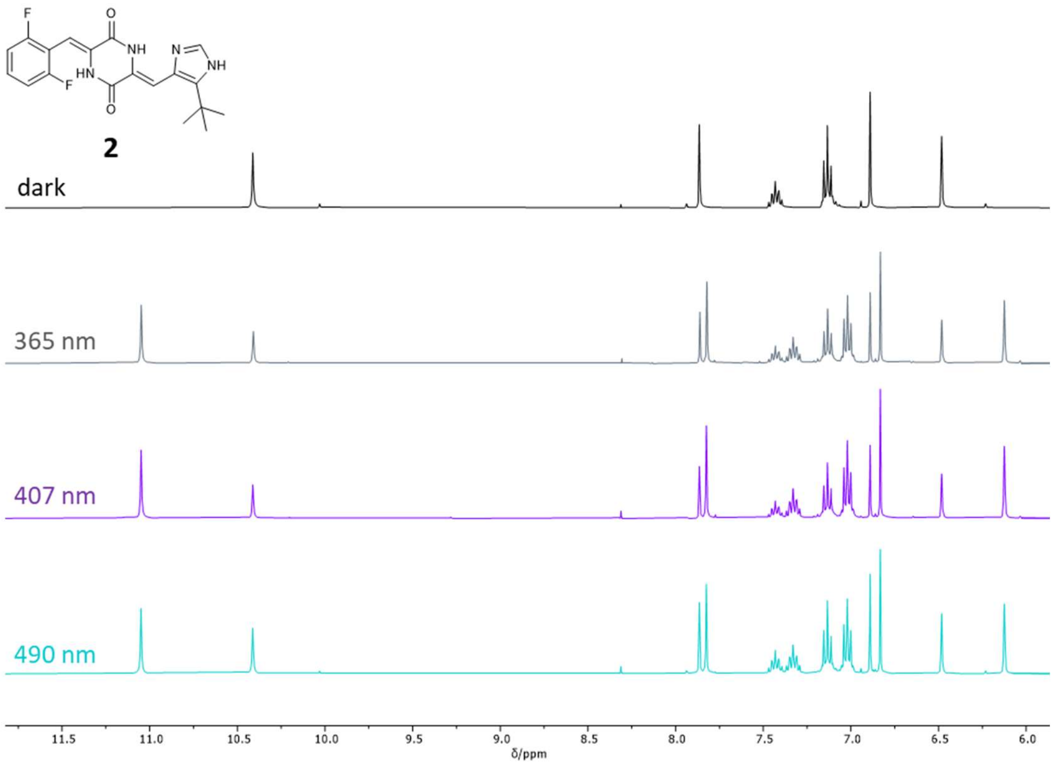

*Supplementary Figure 35: Sections of ^{1}H NMR spectra (400 MHz, DMSO-d$_6$) of compound **2** in the dark (top) and after reaching the PSS upon irradiation with 365 nm, 407 nm and 490 nm.*

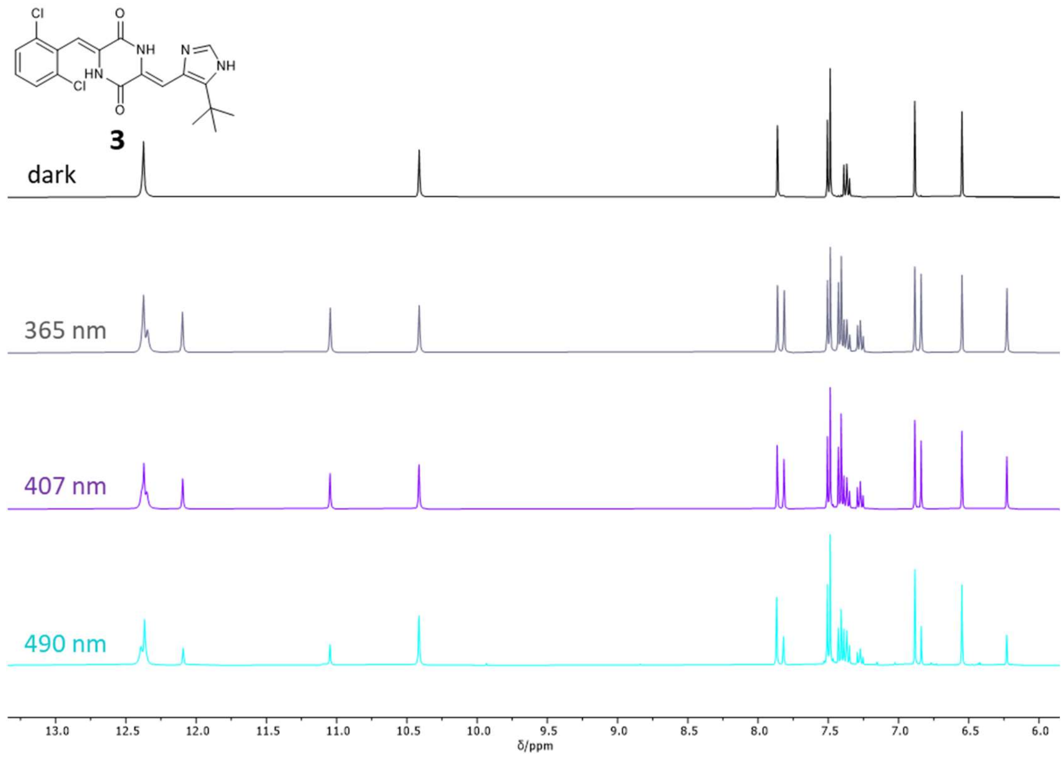

*Supplementary Figure 36: Sections of ^{1}H NMR spectra (400 MHz, DMSO-d$_6$) of compound **3** in the dark (top) and after reaching the PSS upon irradiation with 365 nm, 407 nm and 490 nm.*

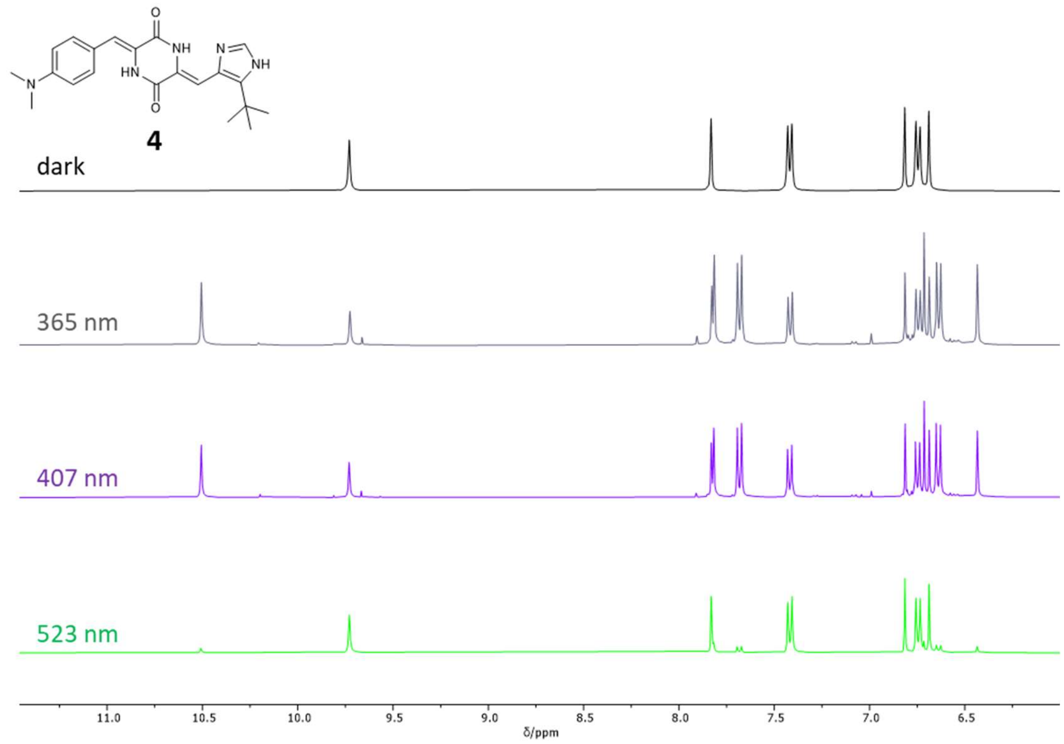

*Supplementary Figure 37: Sections of ^{1}H NMR spectra (400 MHz, DMSO-d$_6$) of compound **4** in the dark (top) and after reaching the PSS upon irradiation with 365 nm, 407 nm and 523 nm.*

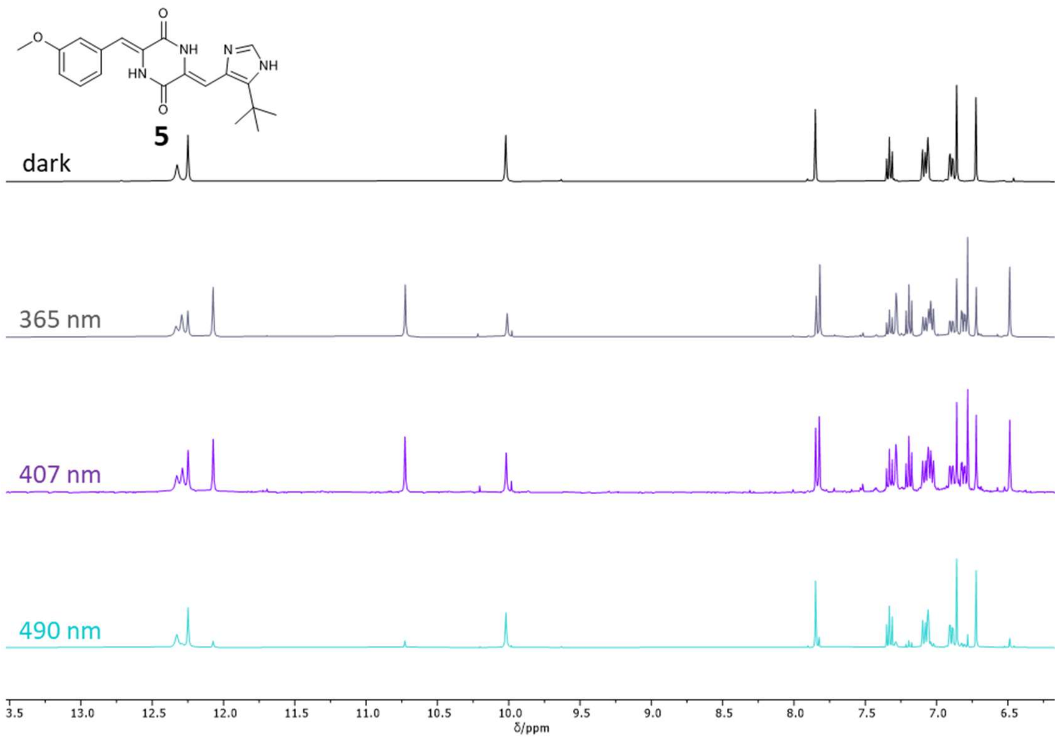

*Supplementary Figure 38: Sections of ^{1}H NMR spectra (400 MHz, DMSO-d_6) of compound **5** in the dark (top) and after reaching the PSS upon irradiation with 365 nm, 407 nm and 490 nm.*

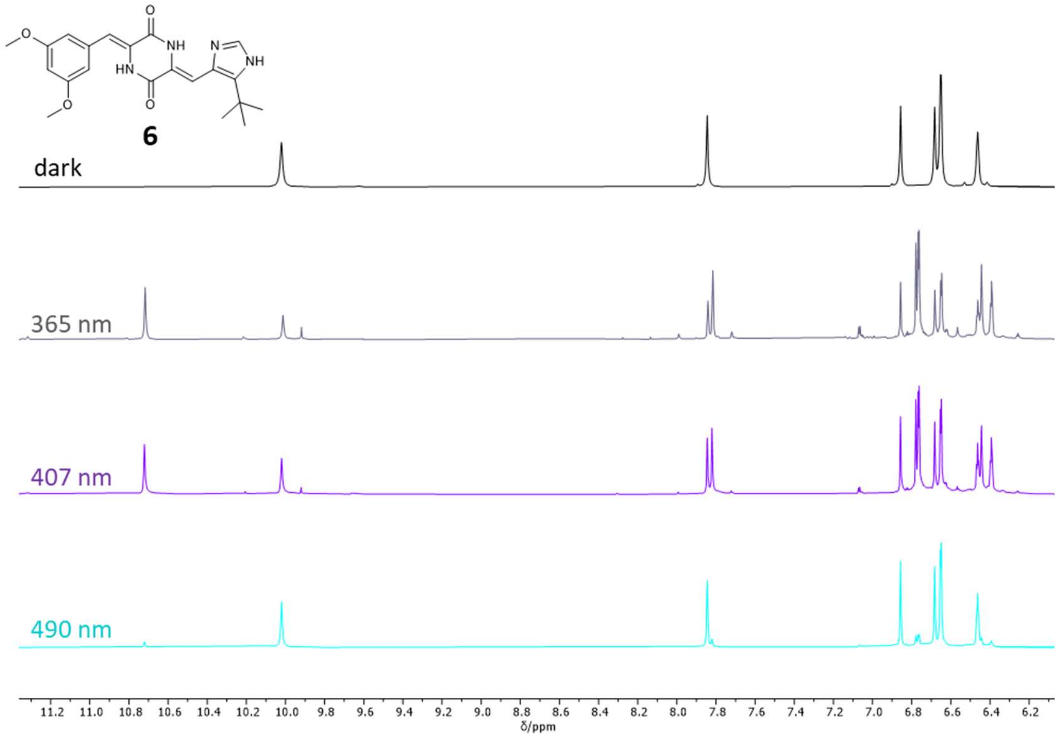

*Supplementary Figure 39: Sections of ^{1}H NMR spectra (400 MHz, DMSO-d_6) of compound **6** in the dark (top) and after reaching the PSS upon irradiation with 365 nm, 407 nm and 490 nm.*

Hemipiperazines

The PSS composition was determined by measuring ^{1}H NMR spectra of the respective compounds (0.5 mL of the 30-60 mM solutions in DMSO-d_6 or CD_2Cl_2) after irradiation. It was observed that irradiation of solutions of compounds **11** and **12** in CH_2Cl_2 lead not only to photoisomerization but also degradation. Therefore, the corresponding PSS were determined using solutions in DMSO-d_6.

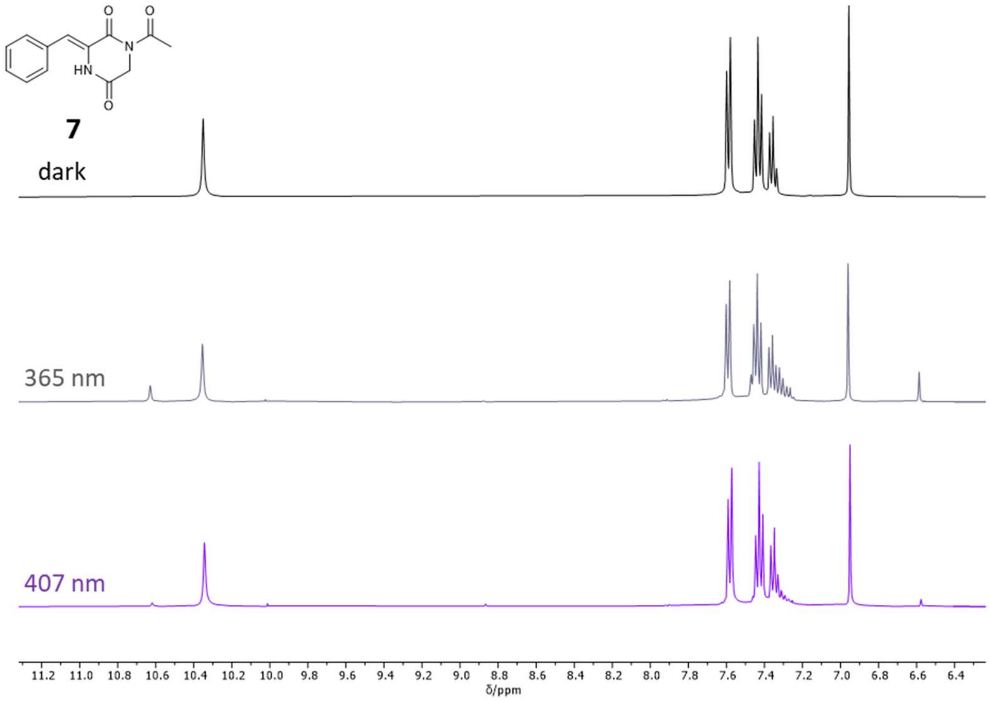

*Supplementary Figure 40: Sections of ^{1}H NMR spectra (400 MHz, DMSO-d_6) of **7** in the dark (top) and after reaching the PSS upon irradiation with 365 nm (middle) and 407 nm (bottom).*

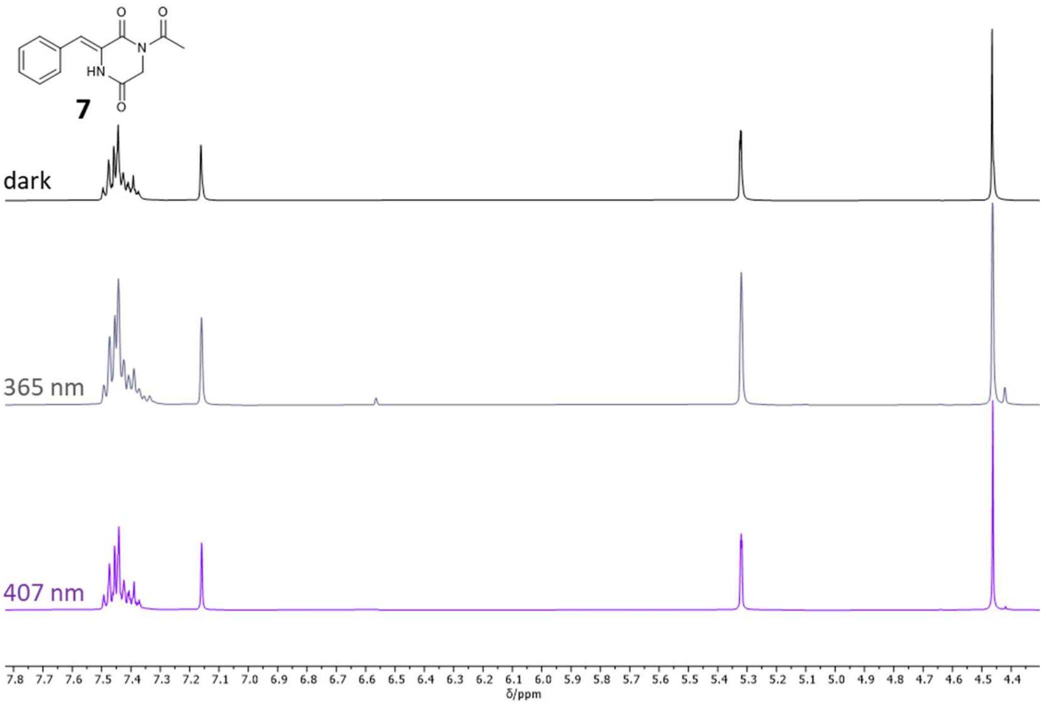

*Supplementary Figure 41: Sections of ¹H NMR spectra (400 MHz, CD_2Cl_2) of **7** in the dark (top) and after reaching the PSS upon irradiation with 365 nm (middle) and 407 nm (bottom).*

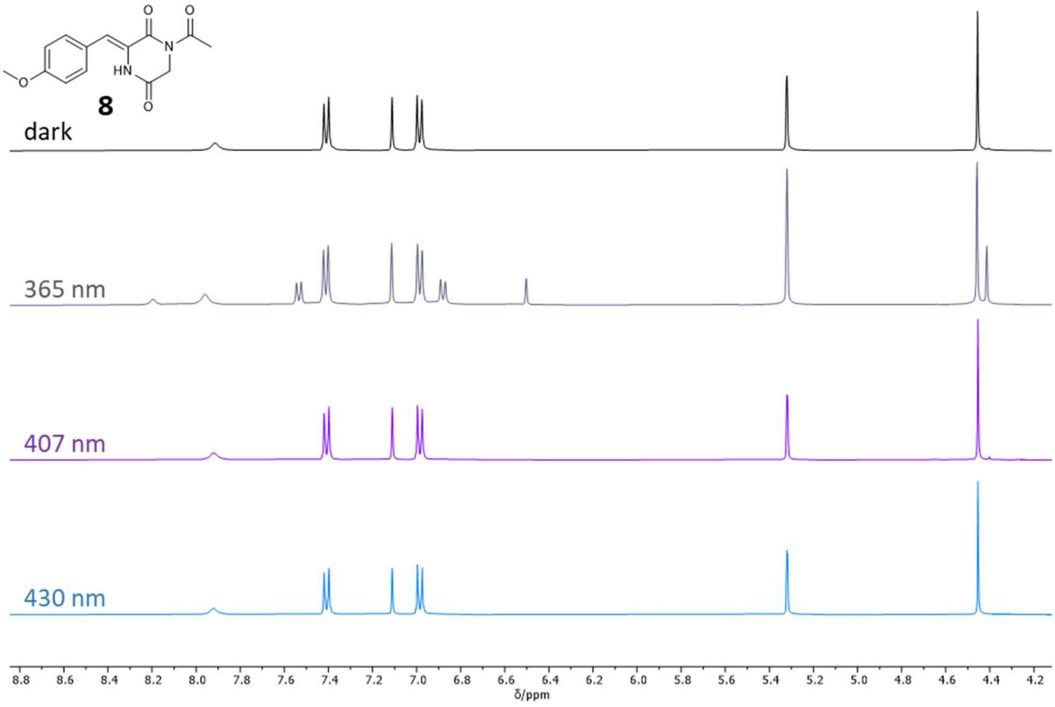

*Supplementary Figure 42: Sections of ¹H NMR spectra (400 MHz, CD_2Cl_2) of **8** in the dark (top) and after reaching the PSS upon irradiation with 365 nm, 407 nm and 430nm.*

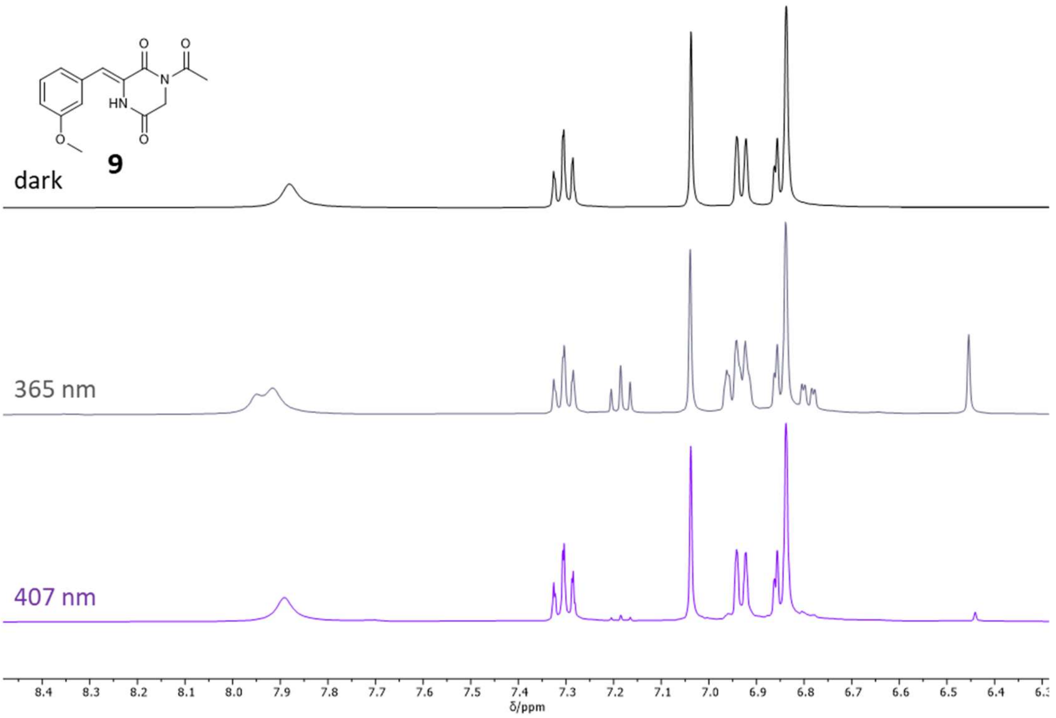

*Supplementary Figure 43: Sections of ^{1}H NMR spectra (400 MHz, CD_2Cl_2) of **9** in the dark (top) and after reaching the PSS upon irradiation with 365 nm (middle) and 407 nm (bottom).*

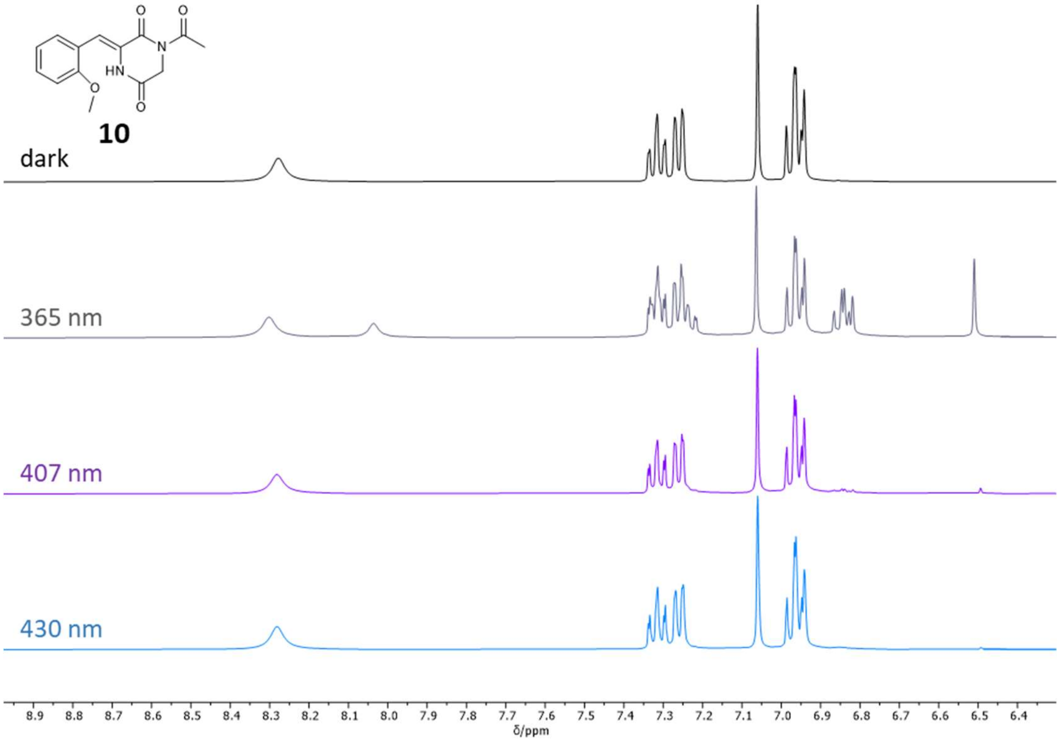

*Supplementary Figure 44: Sections of ^{1}H NMR spectra (400 MHz, CD_2Cl_2) of **10** in the dark (top) and after reaching the PSS upon irradiation with 365 nm, 407 nm and 430nm.*

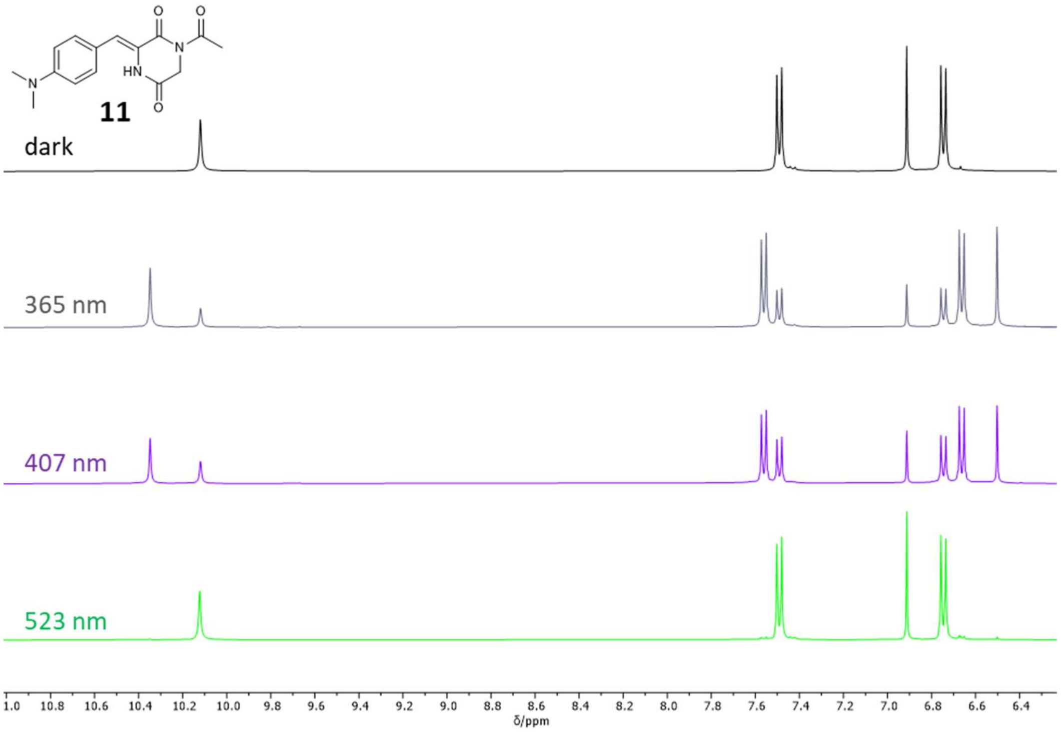

*Supplementary Figure 45: Sections of ^{1}H NMR spectra (400 MHz, DMSO-d_6) of compound **11** in the dark (top) and after reaching the PSS upon irradiation with 365 nm, 407 nm and 523 nm.*

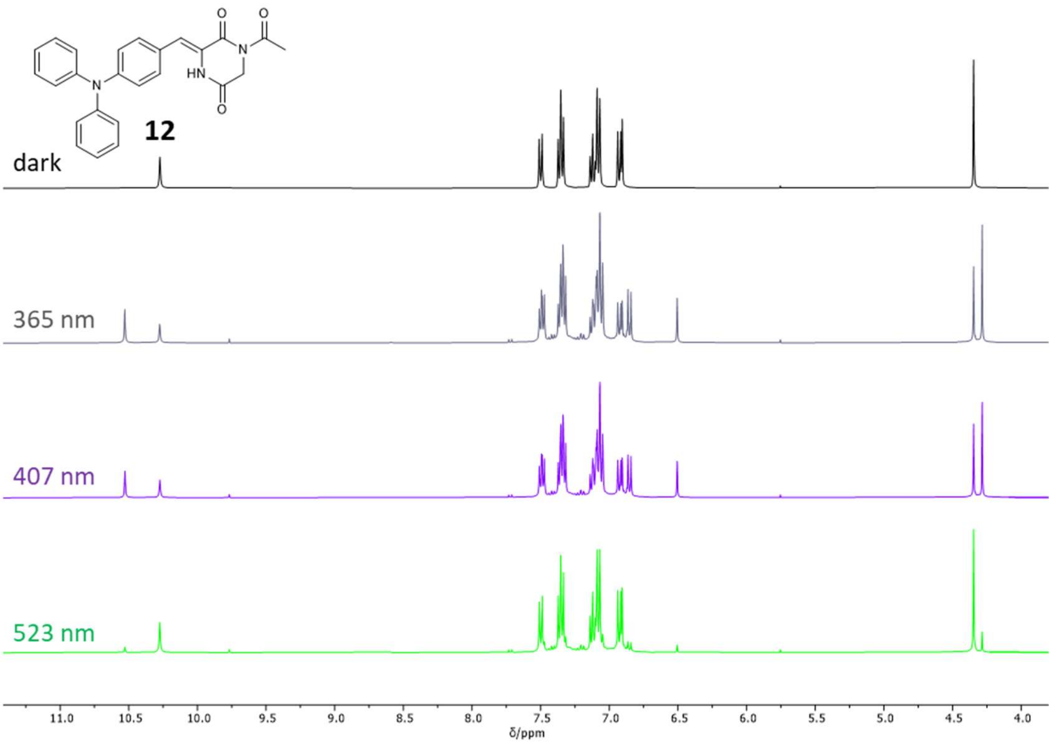

*Supplementary Figure 46: Sections of ^{1}H NMR spectra (400 MHz, DMSO-d_6) of compound **12** in the dark (top) and after reaching the PSS upon irradiation with 365 nm, 407 nm and 523 nm.*

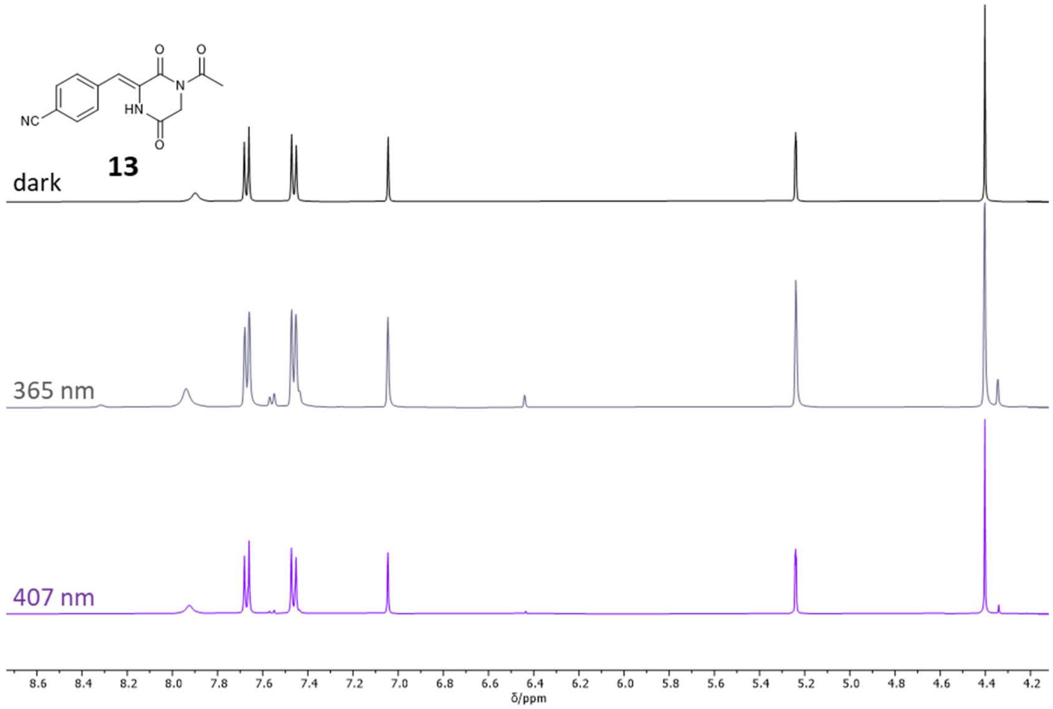

*Supplementary Figure 47: Sections of ^{1}H NMR spectra (400 MHz, CD_2Cl_2) of **13** in the dark (top) and after reaching the PSS upon irradiation with 365 nm (middle) and 407 nm (bottom).*

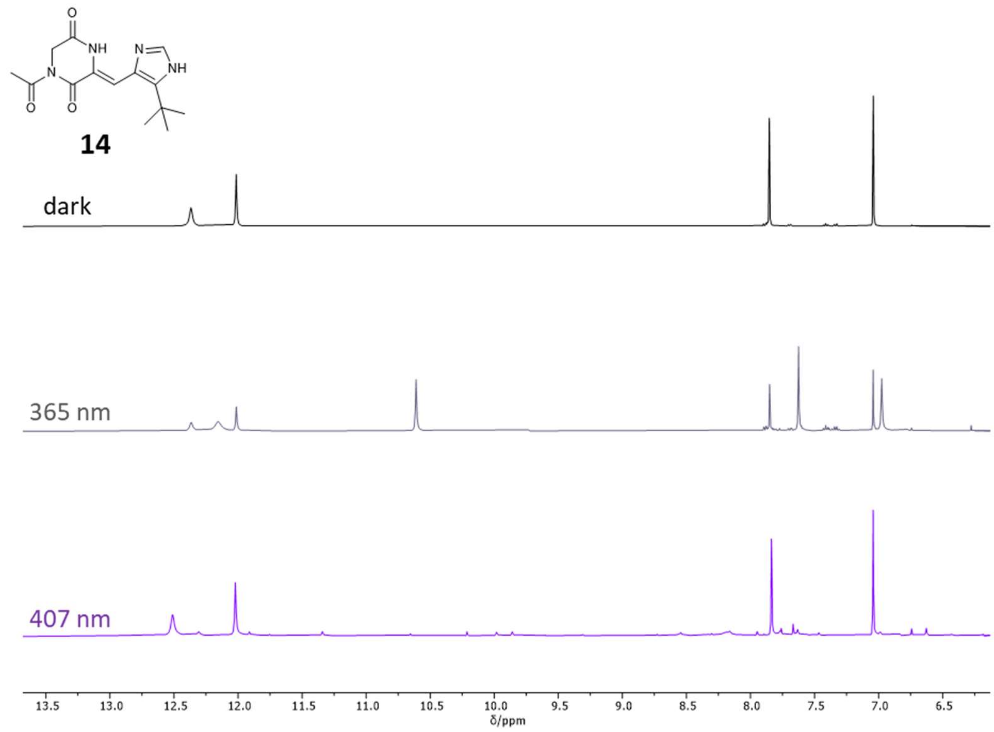

*Supplementary Figure 48: Sections of ^{1}H NMR spectra (400 MHz, DMSO-d_6) of **14** in the dark (top) and after reaching the PSS upon irradiation with 365 nm (middle) and 407 nm (bottom).*

Determination of PSS Composition *via* Analytical HPLC

The PSS composition was determined by analyzing 1 mM solutions of compound **15** in a 5:1 (v/v) mixture of a saturated solution of ascorbic acid in DMSO and DMSO after irradiation *via* analytical HPLC. The PSS values in have been achieved within 10 min (365, 410, 450 nm) or 20 min (490 nm). Triplicates were obtained by including two samples that were irradiated further after the PSS was reached. The signals assigned to the *Z*-isomer were integrated and divided by the signal area determined before irradiation:

% *Z*-isomer = $I_{after\ irradiation}$ /I_{dark}*100% (% *E*-isomer = 100% - % of *Z*-isomer).

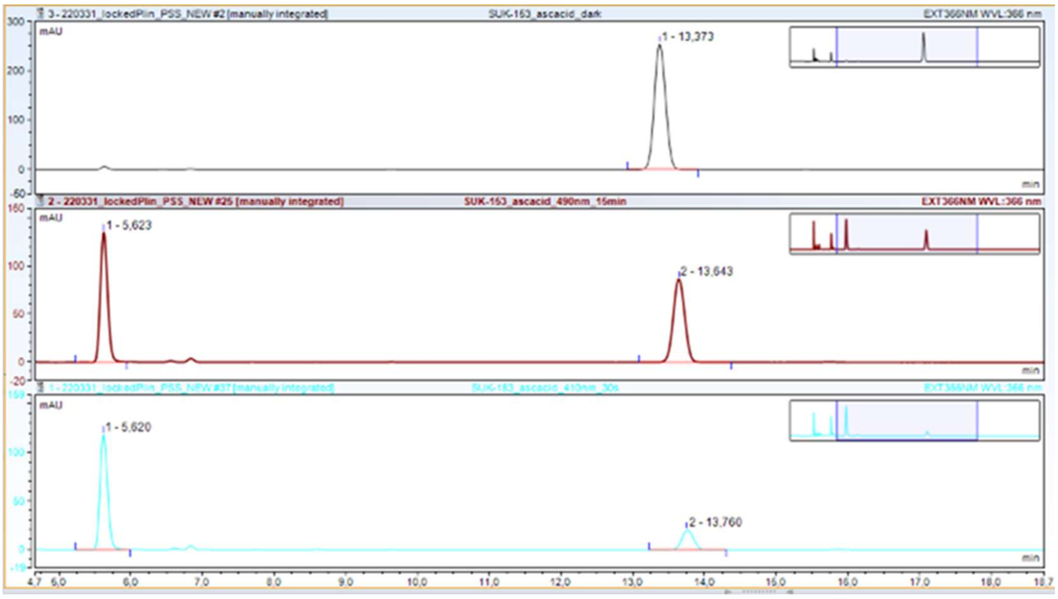

*Supplementary Figure 49: HPLC trace of 1 mM solutions of **15** in a 5:1 (v/v) mixture of a saturated solution of ascorbic acid in DMSO and DMSO before irradiation (top) and after reaching the PSS upon irradiation with 490 nm (middle) and 410 nm (bottom).*

Determination of Isomerization Quantum Yields

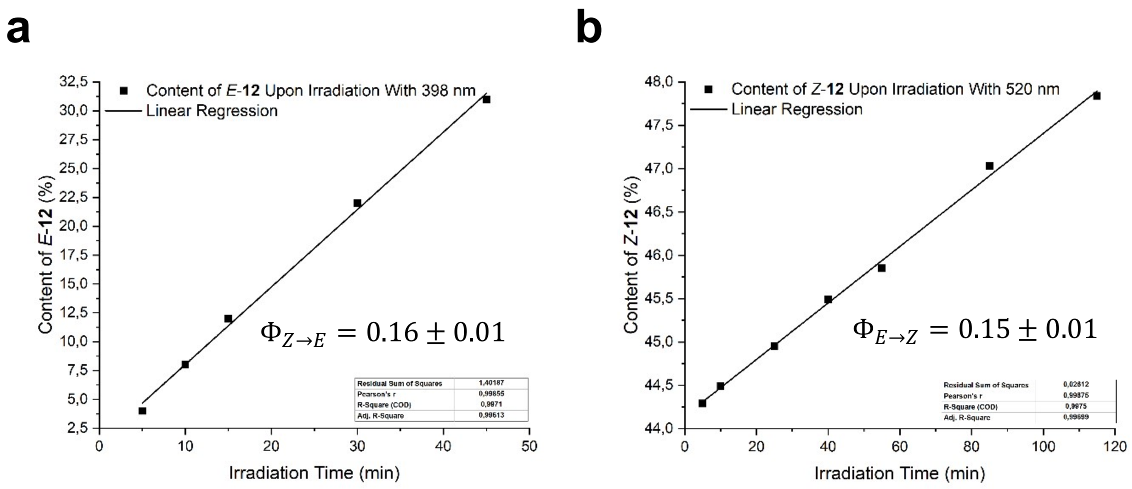

*Supplementary Figure 50: Content of E-**12** upon irradiation with 398 nm over time (a) and content of Z-**12** upon irradiation with 520 nm over time (b) in the isomer mixture, determined via HPLC.*

Thermal Relaxation

The thermal relaxation of the respective *E*-isomers of **1-15** was monitored by first irradiating solutions in MeCN, in DMSO or in mixtures of PBS and DMSO, then heating up and analyzing samples after selected time periods *via* analytical HPLC. Heating solutions of compounds **2** and **8-13** in DMSO led to degradation. Consequently, these compounds were monitored in MeCN at 60 °C.

<u>*Plinabulin Derivatives and Synthetic Intermediates **7** and **14***</u>

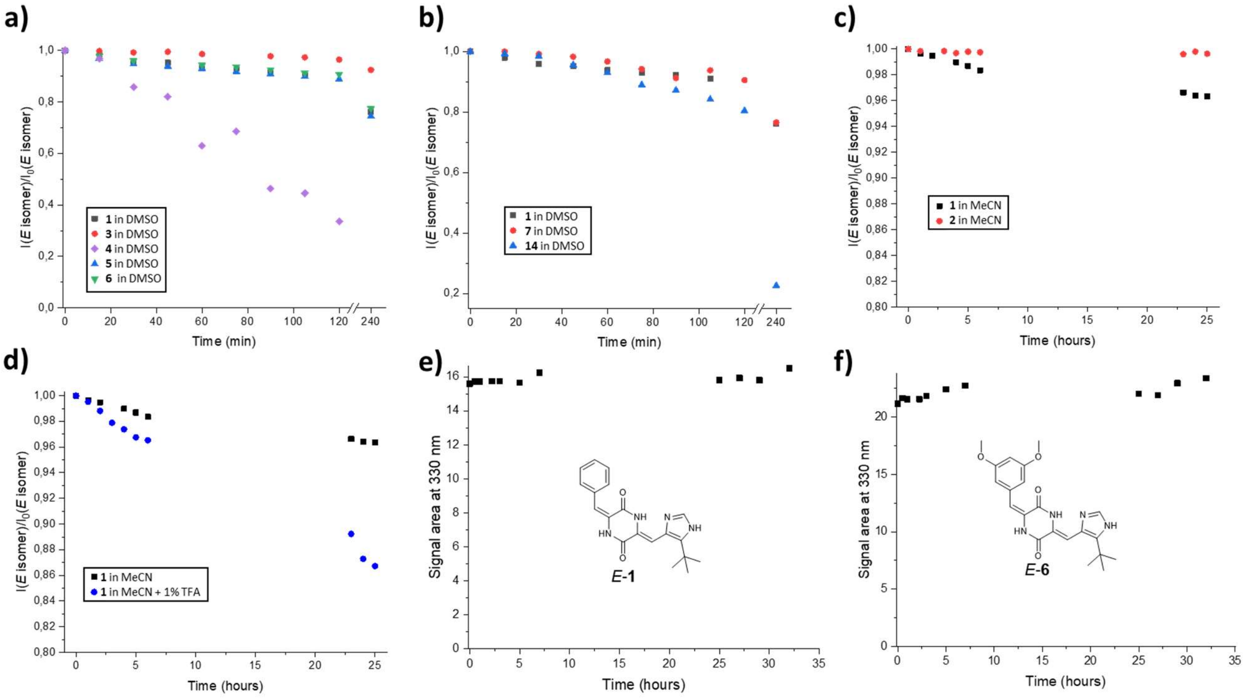

*Supplementary Figure 51: The thermal relaxation of the respective E-isomers of **1**-**7**, **14** and **15** was monitored via analytical HPLC. The integrals of the signals assigned to the respective E-isomer were determined at 330 nm: I_0 = initial integral, I = integral at given time point. (**a**) Thermal relaxation of the respective E-isomers of **1** and **3**-**6** in DMSO (1.0 mM) at 150 °C. (**b**) Thermal relaxation of E-**1**, E-**7**, and E-**14** in DMSO (1.0 mM) at 150 °C. (**c**) Thermal relaxation of E-**1** and E-**2** in MeCN (1.0 mM) at 60 °C. (**d**) Thermal relaxation of E-**1** in MeCN (1.0 mM) at 60 °C in absence and presence of TFA. (**e**) Thermal relaxation of E-**1** in PBS/DMSO (1:1) (33 µM) at 37°C. (**f**) Thermal relaxation of E-**6** in PBS/DMSO (1:1) (33 µM) at 37°C.*

Hemipiperazines

*Supplementary Table 6: The thermal relaxation of the respective E-isomers of **7**-**10**, **12** and **13** in MeCN (0.1 mM) at 60 °C after one week was monitored via analytical HPLC. Due to the decomposition upon measurements under these conditions, thermal stability of the compound E-**11** was not quantified, but estimated to be between 8% and 12% within the same time period and conditions.*

**Compound**	**7**	**8**	**9**	**10**	**12**	**13**
Decrease of *E*-isomer after 7 days	4.0%	0.8%	2.2%	1.3%	12.1%	0.8%

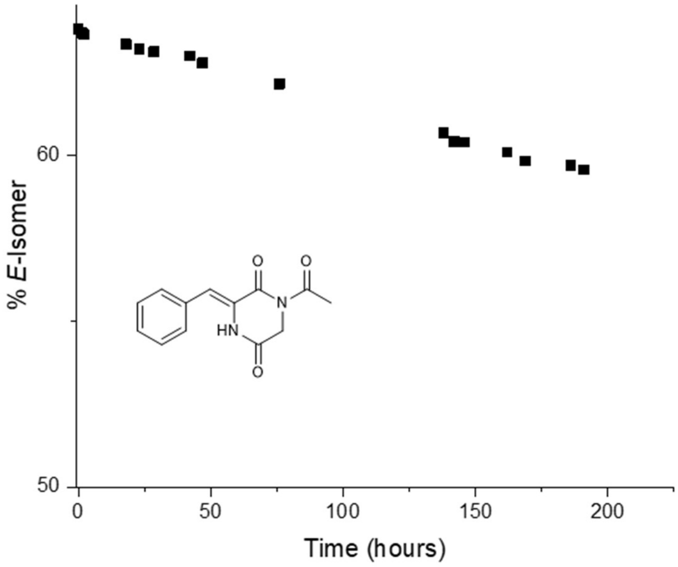

*Supplementary Figure 52: Thermal relaxation of E-**7** in MeCN (0.1 mM) at 60 °C was monitored via analytical HPLC. The percentage of E-isomer was determined with the integrals of the signals assigned to the respective isomer at 342 nm.*

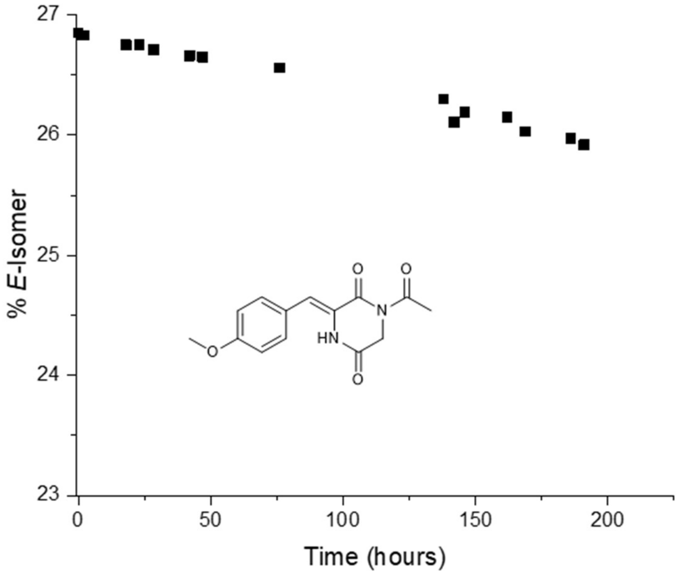

*Supplementary Figure 53: Thermal relaxation of E-**8** in MeCN (0.1 mM) at 60 °C was monitored via analytical HPLC. The percentage of E-isomer was determined with the integrals of the signals assigned to the respective isomer at 450 nm.*

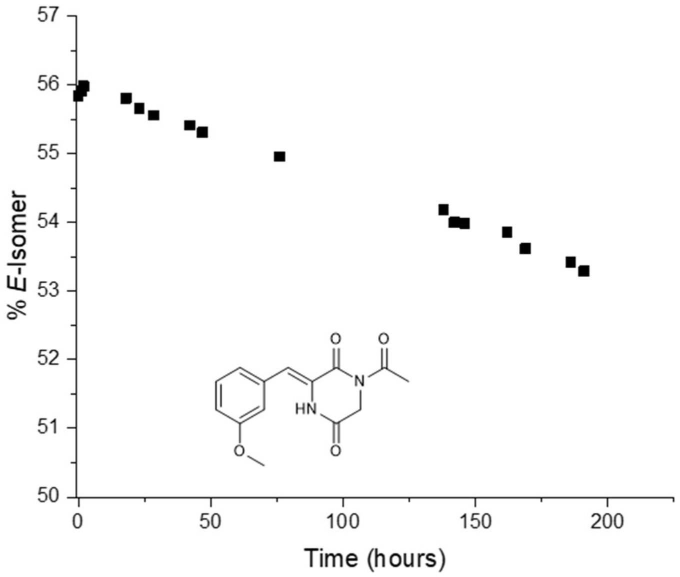

*Supplementary Figure 54: Thermal relaxation of E-**9** in MeCN (0.1 mM) at 60 °C was monitored via analytical HPLC. The percentage of E-isomer was determined with the integrals of the signals assigned to the respective isomer at 349 nm.*

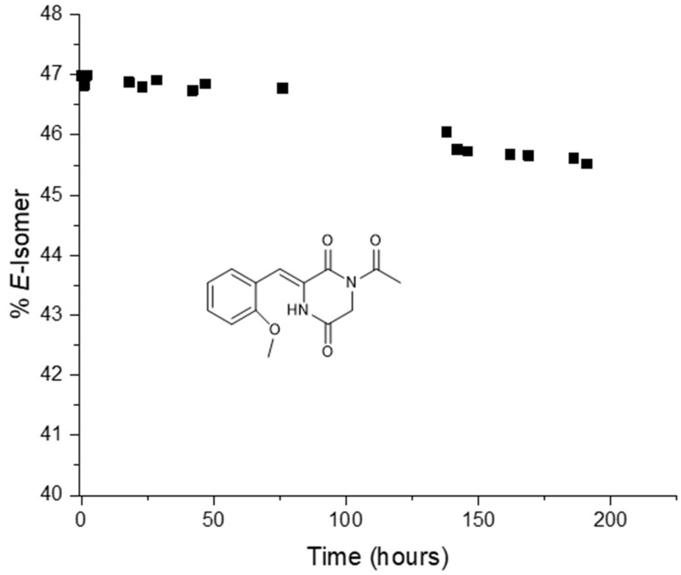

*Supplementary Figure 55: Thermal relaxation of E-**10** in MeCN (0.1 mM) at 60 °C was monitored via analytical HPLC. The percentage of E-isomer was determined with the integrals of the signals assigned to the respective isomer at 368 nm.*

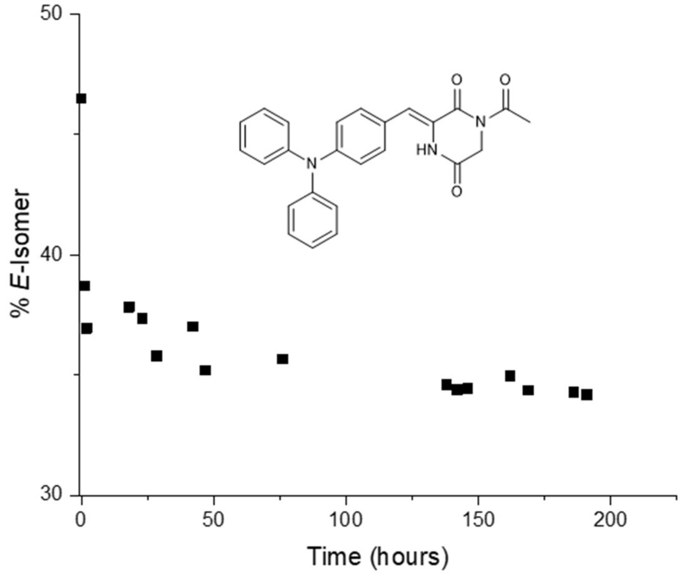

*Supplementary Figure 56: Thermal relaxation of E-**12** in MeCN (0.1 mM) at 60 °C was monitored via analytical HPLC. The percentage of E-isomer was determined with the integrals of the signals assigned to the respective isomer at 325 nm.*

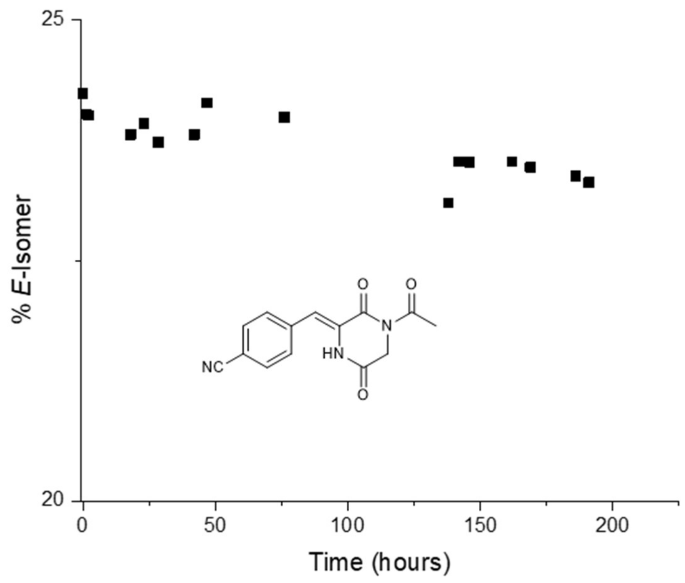

*Supplementary Figure 57: Thermal relaxation of E-**13** in MeCN (0.1 mM) at 60 °C was monitored via analytical HPLC. The percentage of E-isomer was determined with the integrals of the signals assigned to the respective isomer at 355nm.*

"Locked" Plinabulin

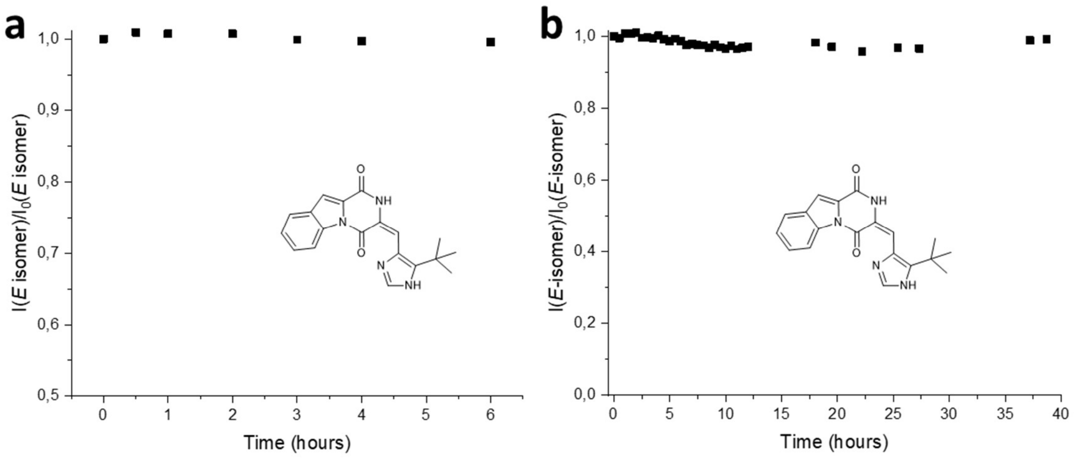

*Supplementary Figure 58 The thermal relaxation of E-**15** in (a) DMSO (0.1 mM) at 25 °C and (b) in PBS/DMSO (1:1) (0.1 mM) at 37 °C was monitored via analytical HPLC. The integrals of the signals assigned to the respective E-isomer were determined at 330 nm: I0 = initial integral, I = integral at given time point.*

Switching Stability: Plinabulin

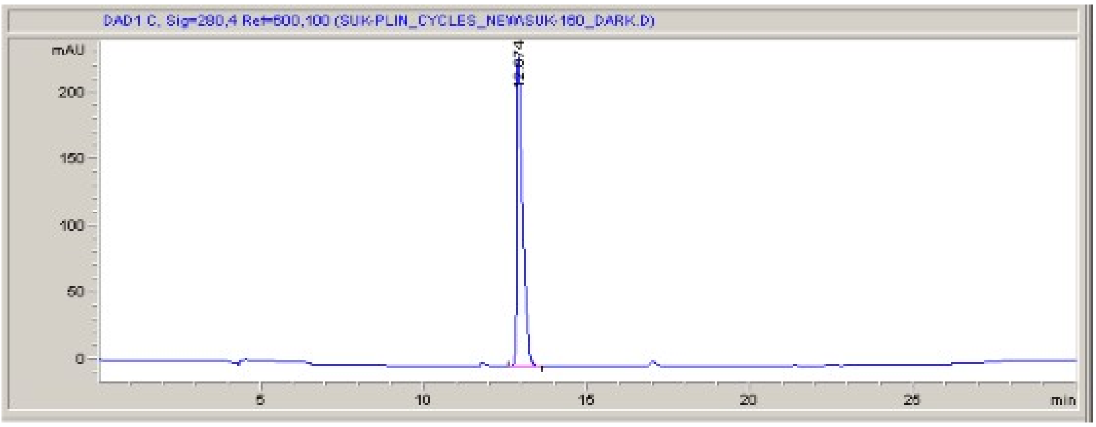

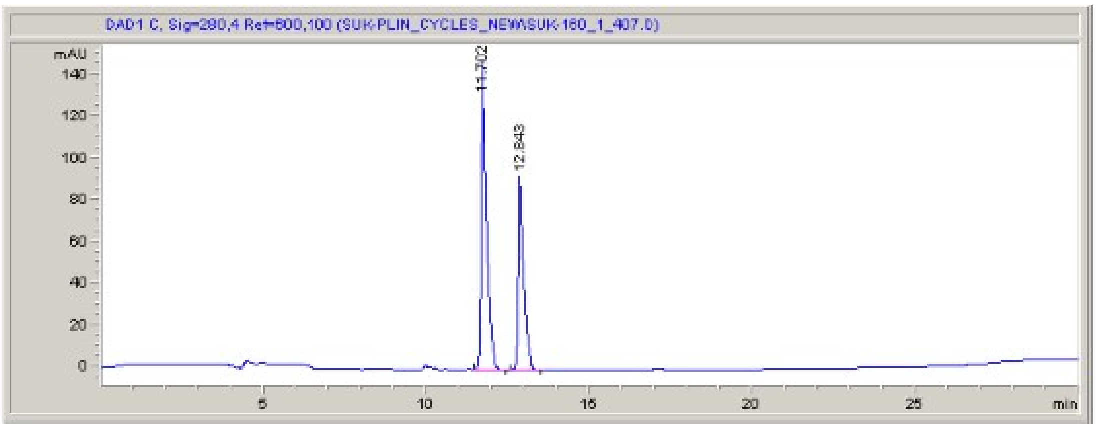

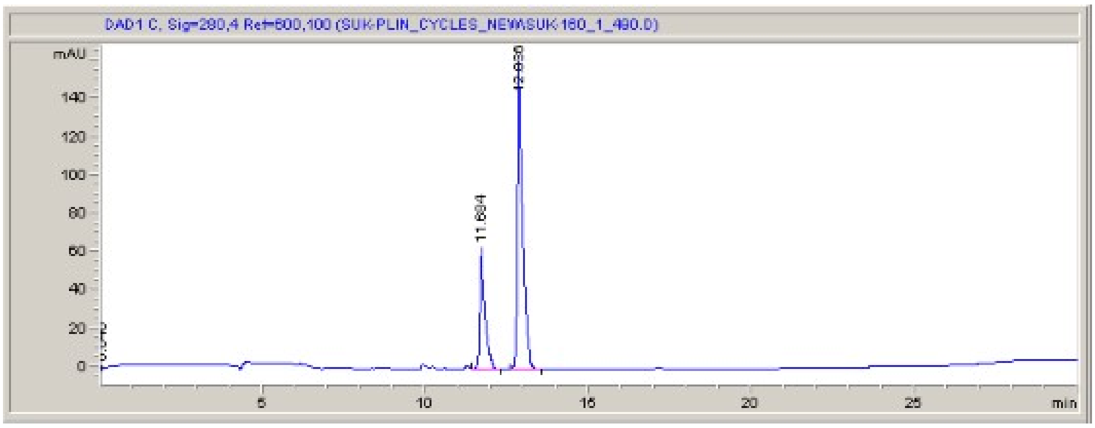

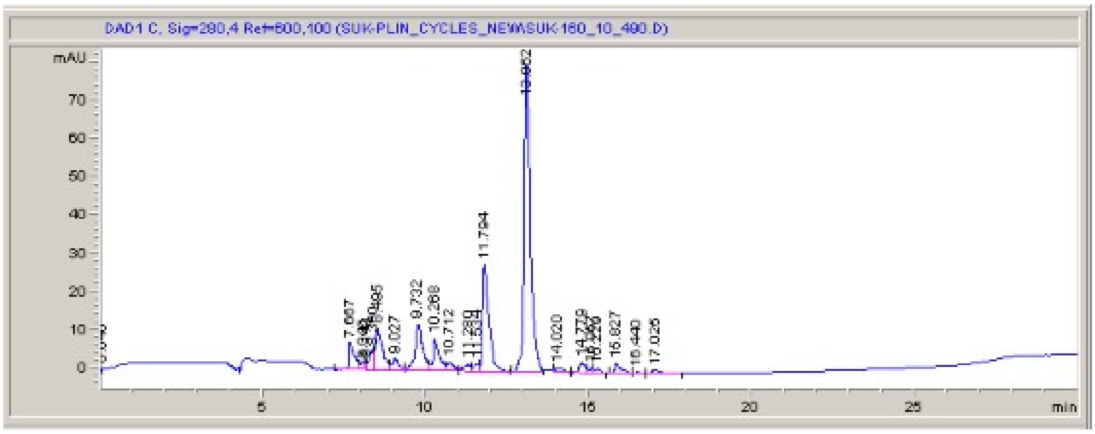

*Supplementary Figure 59: Photodegradation of **1** in the presence of atmospheric oxygen (air) - A 40 µM solution of **1** in DMSO was irradiated under ambient atmosphere with alternating 407 nm and 490 nm light. The HPLC chromatograms of the non-irradiated Z-**1**, first irradiation with 407 nm, first irradiation with 490 nm, and upon 10 cycles of 407/490 nm irradiation (from top to bottom). The last chromatogram clearly demonstrates multiple degradation (photooxidation) products, which could not be purified nor unequivocally identified.*

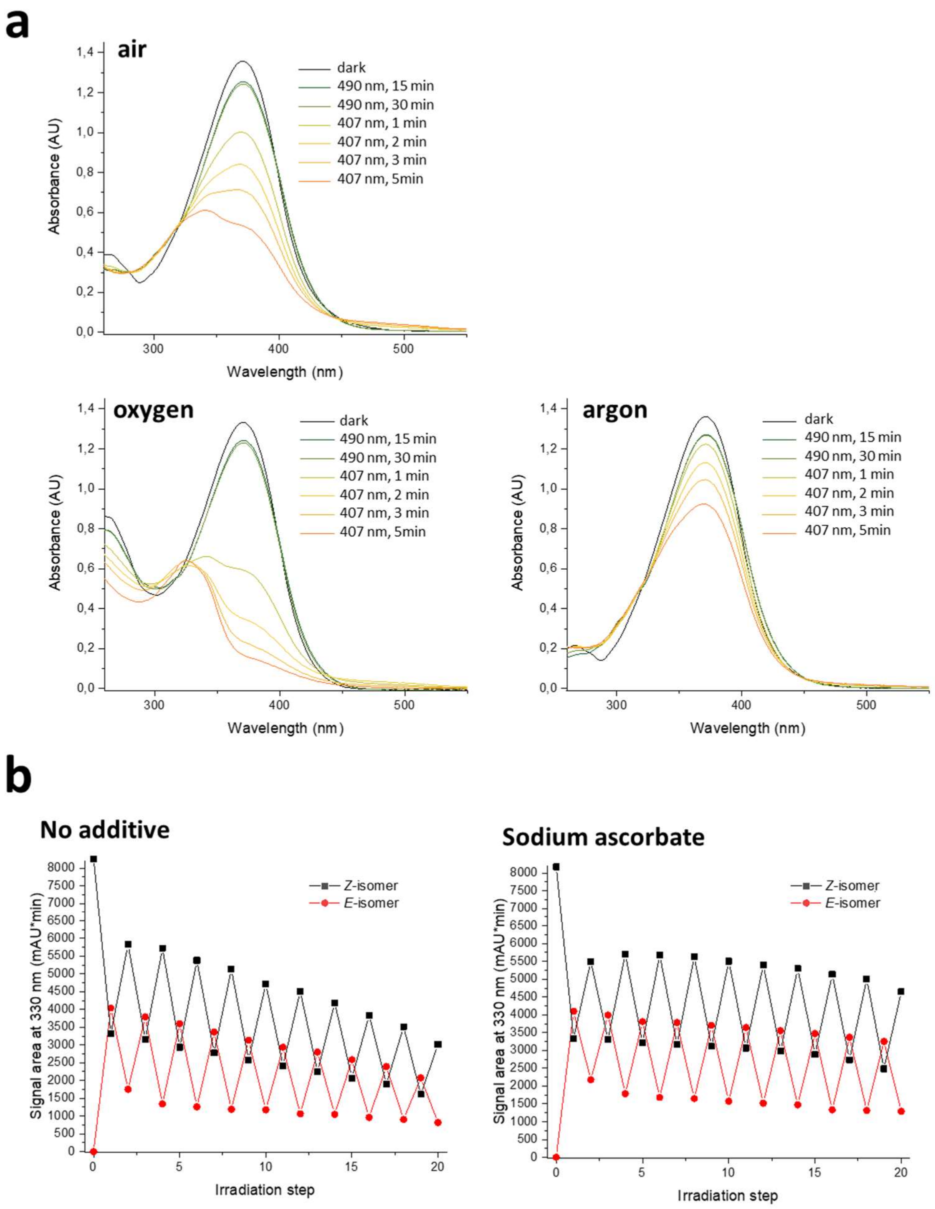

Supplementary Figure 60: Stability of plinabulin (1) upon photoisomerization

*(**a**) A 40 µM solution of 1 in DMSO was prepared and split up into three samples. One sample was kept under ambient atmosphere, the other samples were purged for 5 min with oxygen or argon, respectively. UV-Vis absorption spectra were first recorded in the dark and then after each irradiation step. Afterwards, the samples were first irradiated twice for 15 min with 490 nm and subsequently five times for 1 min with 407 nm. (**b**) Solutions of pure Z-1 in DMSO (1.0 mM) in DMSO and in a saturated solution of ascorbic acid in DMSO were analyzed via analytical HPLC. The samples were irradiated with 407 nm for 2 min and with 490 nm for 35 min, respectively. The irradiation cycles were repeated ten times and after every irradiation step the solutions were analyzed via analytical HPLC.*

Switching Stability: Hemipiperazines

Solutions of pure *Z*-isomers in DMSO (1.0 mM) were analyzed *via* analytical HPLC. The samples were irradiated consecutively with two wavelengths, respectively. The irradiation cycles were repeated ten times and after every irradiation step the solutions were analyzed *via* analytical HPLC. The area of the signals assigned to the respective photoisomers at 330 nm were determined and plotted.

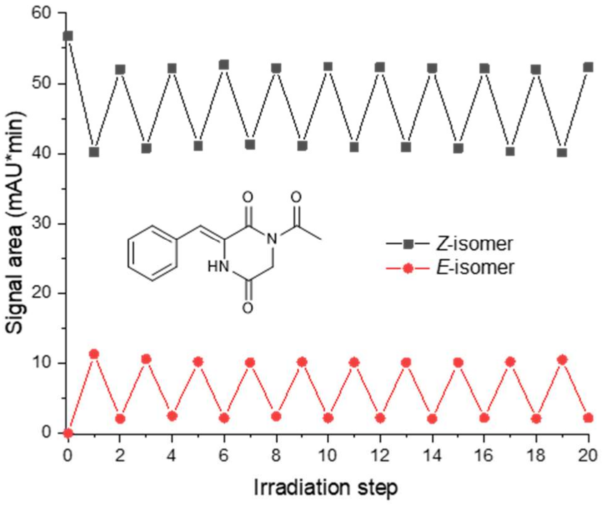

*Supplementary Figure 61: Solutions of pure Z-**7** in DMSO (1.0 mM) were irradiated with 365 nm for 2 min and with 407 nm for 10 min, respectively. The irradiation cycles were repeated ten times and after every irradiation step the solutions were analyzed via analytical HPLC.*

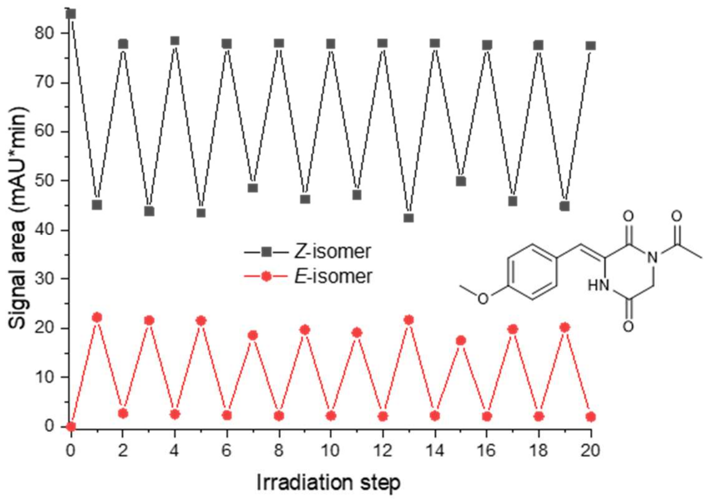

*Supplementary Figure 62: Solutions of pure Z-**8** in DMSO (1.0 mM) were irradiated with 365 nm for 2 min and with 430 nm for 20 min, respectively. The irradiation cycles were repeated ten times and after every irradiation step the solutions were analyzed via analytical HPLC.*

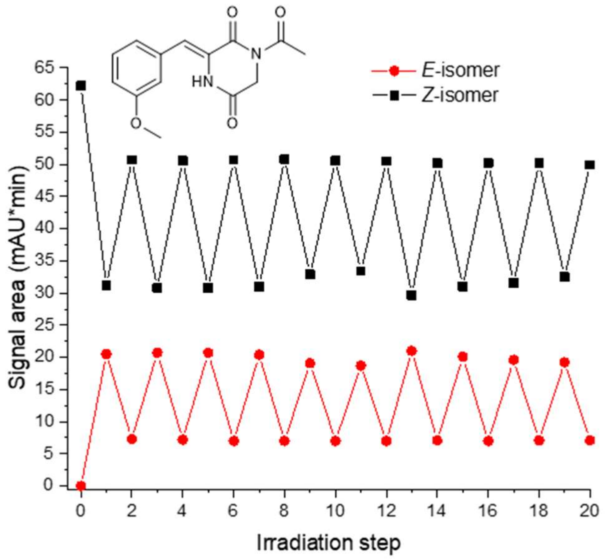

*Supplementary Figure 63: Solutions of pure Z-**9** in DMSO (1.0 mM) were irradiated with 365 nm for 2 min and with 407 nm for 5 min, respectively. The irradiation cycles were repeated ten times and after every irradiation step the solutions were analyzed via analytical HPLC.*

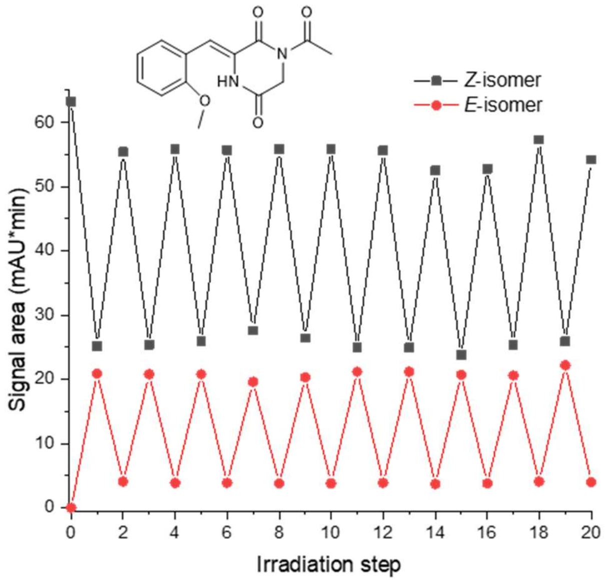

*Supplementary Figure 64: Solutions of pure Z-**10** in DMSO (1.0 mM) were irradiated with 365 nm for 4 min and with 430 nm for 45 min, respectively. The irradiation cycles were repeated ten times and after every irradiation step the solutions were analyzed via analytical HPLC.*

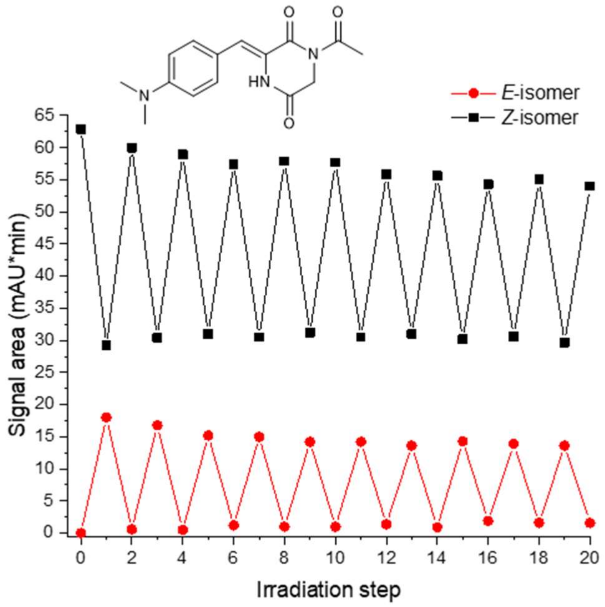

*Supplementary Figure 65: Solutions of pure Z-**11** in DMSO (1.0 mM) were irradiated with 407 nm for 4 min and with 523 nm for 10 min, respectively. The irradiation cycles were repeated ten times and after every irradiation step the solutions were analyzed via analytical HPLC.*

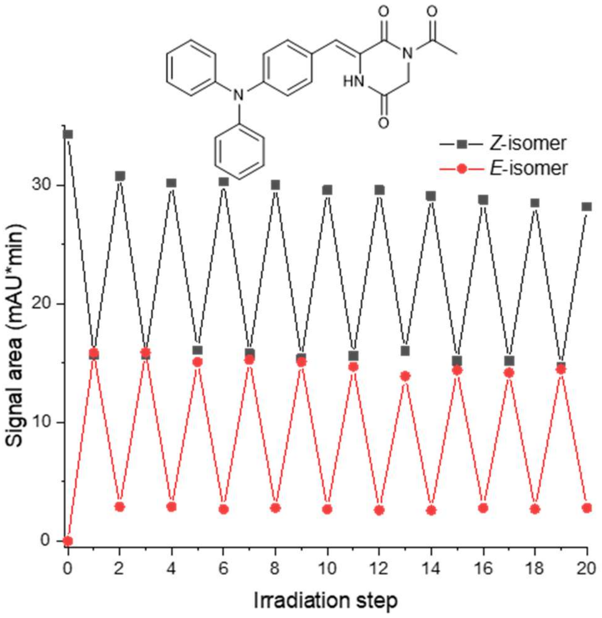

*Supplementary Figure 66: Solutions of pure Z-**12** in DMSO (1.0 mM) were irradiated with 407 nm for 5 min and with 523 nm for 35 min, respectively. The irradiation cycles were repeated ten times and after every irradiation step the solutions were analyzed via analytical HPLC.*

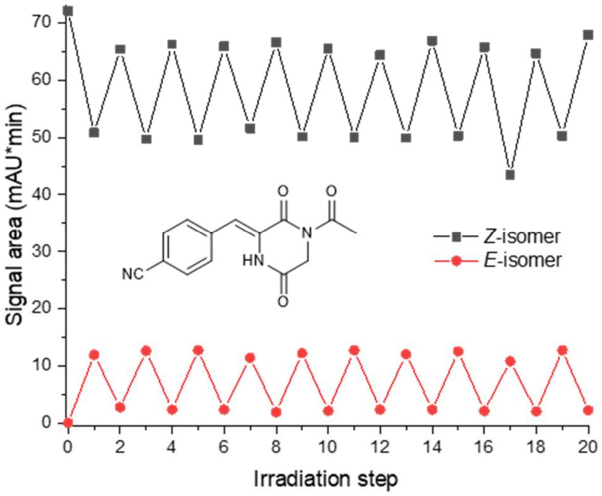

*Supplementary Figure 67: Solutions of pure Z-**13** in DMSO (1.0 mM) were irradiated with 365 nm for 4 min and with 430 nm for 45 min, respectively. The irradiation cycles were repeated ten times and after every irradiation step the solutions were analyzed via analytical HPLC.*

Switching Stability: “Locked” Plinabulin

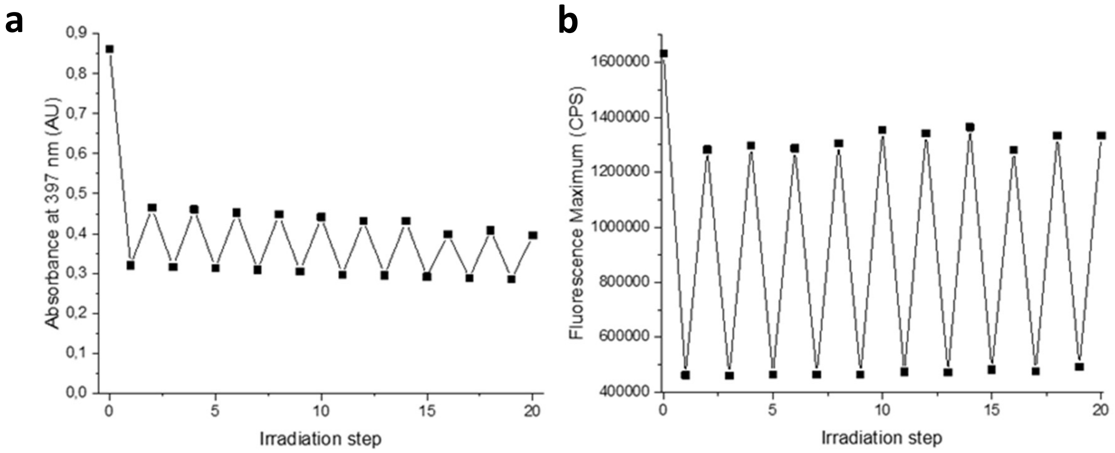

*Supplementary Figure 68: A solution of pure Z-**15** in a saturated solution of ascorbic acid in DMSO (200 µM) was irradiated with 410 nm for 30 s and with 490 nm for 12 min, respectively. The irradiation cycle was repeated ten times and after every irradiation step the solution was analyzed via absorbance (a) and fluorescence (b) spectroscopy.*

Stability in Presence of Glutathione

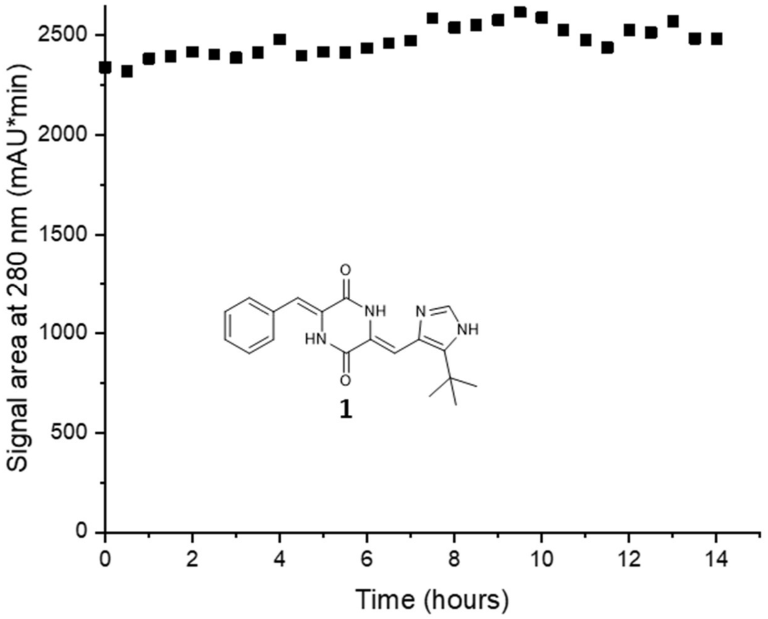

*Supplementary Figure 69: A 1.0 mM solution of **1** with 10.0 mM glutathione and 5.0 mM tris(2-carboxyethyl)phosphine in 25% PBS in DMSO (v/v) was monitored for 14 hours via analytical HPLC. The area of the signal assigned to **1** at 280 nm was determined after each HPLC run.*

Singlet oxygen emission from irradiated samples of plinabulin 1

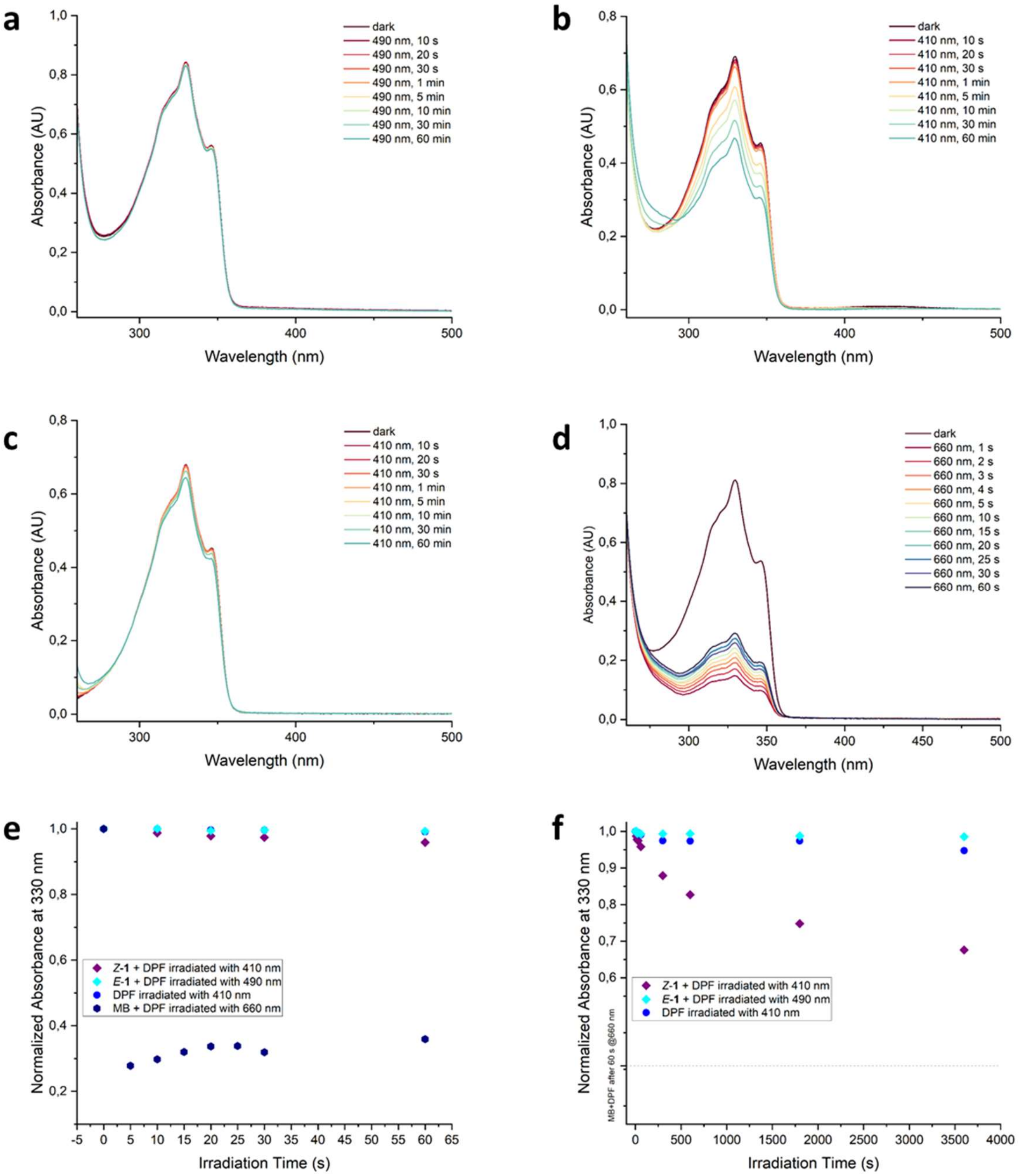

*Supplementary Figure 70: (**a**) A 60µM solution of equal parts E-**1** and DPF in DMSO was blanked to a pure 60 µM solution of E-**1**, both of which were irradiated with 490 nm over the course of 1 hour. (**b**) A 50µM solution of equal parts Z-**1** and DPF in DMSO was blanked to a pure 50 µM solution of Z-**1**, both of which were irradiated with 410 nm over the course of 1 hour. (**c**) A 50 µM solution of pure DPF in DMSO (negative control), was irradiated with 410 nm over the course of one hour. (**d**) A 60µM solution of equal parts MB and DPF in DMSO (positive control), was blanked to a pure 60 µM solution of MB, both of which were irradiated with 660 nm over the course of 1 min. (**e**) Normalized absorbance maximum of DPF in the four samples over the course of 1 min. (**f**) Normalized absorbance maximum of DPF in the four samples over the course of 1 hour; absorbance of positive control after 60 s depicted as a dashed line.*

Crystal Structure Determinations

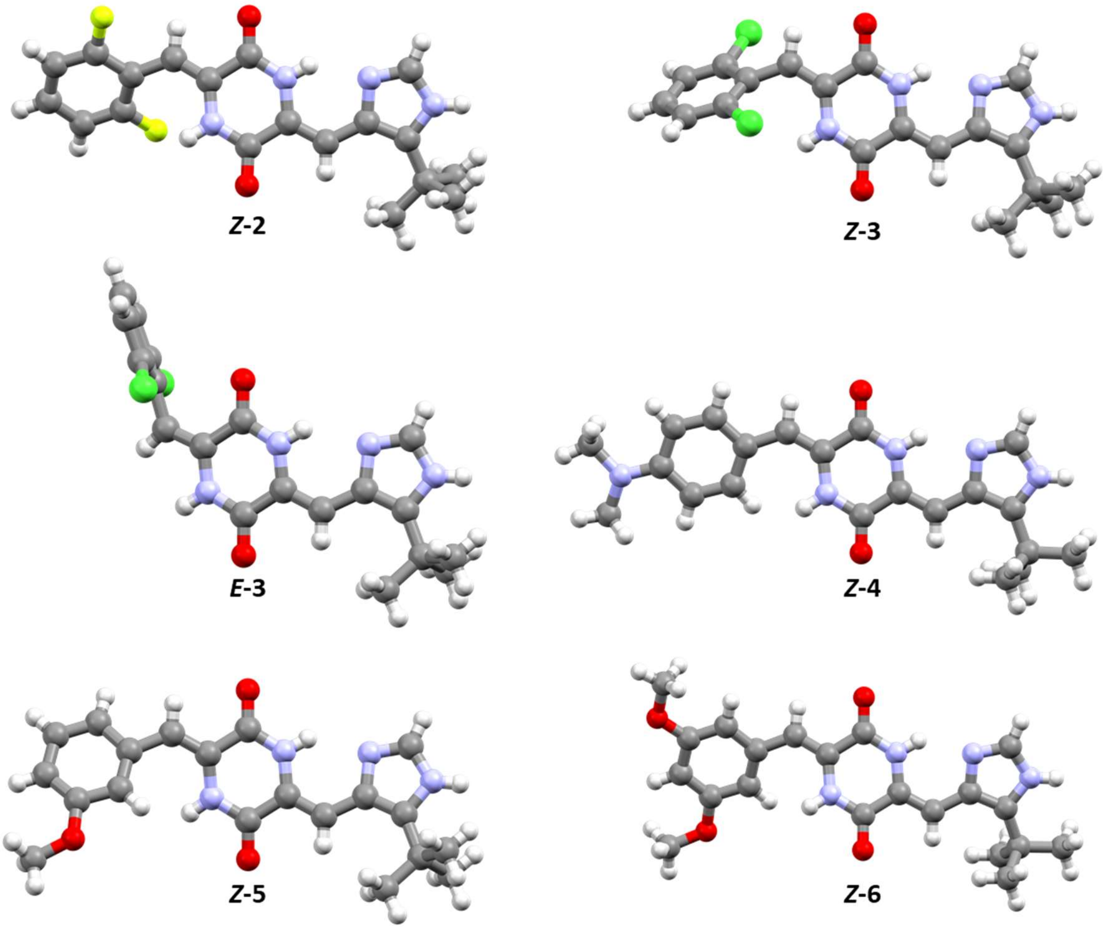

*Supplementary Figure 71: Single crystals of Z-**2**, Z-**3**, E-**3**, Z-**4**, Z-**5** and Z-**6** were analyzed in single-crystal X-ray diffraction studies. The results were visualized with Mercury 2020.1 (CCDC). Additional atoms in the results of Z-**2** (H_2O) and Z-**5** (disordered 3-methoxybenzylidene moiety) were removed in this visual presentation. Atoms of carbon are depicted in grey, hydrogen in white, oxygen in red, nitrogen in blue, fluorine in yellow and chlorine in green.*

***Z*-2:**

SB1177_HY: yellow crystals, $C_{19}H_{18}F_2N_4O_2 \cdot H_2O$, M_r = 390.39, crystal size 0.18 × 0.14 × 0.02 mm, monoclinic, space group $P2_1/n$ (No. 14), a = 7.0552(2) Å, b = 9.6541(3) Å, c = 26.1751(8) Å, β = 94.157(2)°, V = 1778.14(9) Å^3, Z = 4, ρ = 1.458 Mg/m^{-3}, μ(Cu-K$_\alpha$) = 0.97 mm^{-1}, F(000) = 816, $2\theta_{max}$ = 144.4°, T = 123(2) K, 15347 reflections, of which 3502 were independent (R_{int} = 0.037), 268 parameters, 6 restraints, R_1 = 0.041 (for 3133 I > 2σ(I)), wR_2 = 0.102 (all data), S = 1.09, largest diff. peak / hole = 0.32 / -0.20 e Å^{-3}.

***Z*-3:**

SB1192_HY: yellow crystals, $C_{19}H_{18}Cl_2N_4O_2$, M_r = 405.27, crystal size 0.16 × 0.06 × 0.02 mm, orthorhombic, space group $P2_12_12_1$ (No. 19), a = 7.7969(3) Å, b = 14.3794(6) Å, c =

16.8804(7) Å, V = 1892.54(13) Å^3, Z = 4, ρ = 1.422 Mg/m^{-3}, μ(Cu-K$_\alpha$) = 3.28 mm^{-1}, F(000) = 840, $2\theta_{max}$ = 144.4°, T = 123(2) K, 15438 reflections, of which 3736 were independent (R_{int} = 0.066), 253 parameters, 3 restraints, R_1 = 0.044 (for 3264 I > 2σ(I)), wR_2 = 0.102 (all data), S = 1.06, largest diff. peak / hole = 0.27 / -0.20 e Å^{-3}, x = 0.013(13).

***E*-3**

SB1354_HY: yellow crystals $C_{19}H_{18}Cl_2N_4O_2$, M_r = 405.27, crystal size 0.15 × 0.105 × 0.03 mm, monoclinic, space group C2/c (No. 15), a = 27.8992(10) Å, b = 10.2709(4) Å, c = 14.6321(5) Å, β = 116.603(2)°, V = 3748.9(2) Å^3, Z = 8, ρ = 1.436 Mg/m^{-3}, μ(Cu-K$_\alpha$) = 3.31 mm^{-1}, F(000) = 1680, $2\theta_{max}$ = 144.8°, T = 298(2) K, 21109 reflections, of which 3705 were independent (R_{int} = 0.039), 253 parameters, 219 restraints (general RIGU restraints, see cif-file for details), R_1 = 0.041 (for 3057 I > 2σ(I)), wR_2 = 0.103 (all data), S = 1.03, largest diff. peak / hole = 0.28 / -0.39 e Å^{-3}.

***Z*-4**

SB1373_HY: orange crystals, $C_{21}H_{25}N_5O_2$, M_r = 379.46, crystal size 0.15 × 0.09 × 0.03 mm, monoclinic, space group $P2_1$/c (No. 14), a = 7.2824(2) Å, b = 14.1512(4) Å, c = 18.4592(5) Å, β = 94.740(1)°, V = 1895.80(9) Å^3, Z = 4, ρ = 1.329 Mg/m^{-3}, μ(Cu-K$_\alpha$) = 0.71 mm^{-1}, F(000) = 808, $2\theta_{max}$ = 144.2°, T = 173(2) K, 22924 reflections, of which 3722 were independent (R_{int} = 0.026), 265 parameters, 3 restraints, R_1 = 0.036 (for 3420 I > 2σ(I)), wR_2 = 0.093 (all data), S = 1.03, largest diff. peak / hole = 0.23 / -0.17 e Å^{-3}.

***Z*-5**

SB1342_HY: yellow crystals, $C_{20}H_{22}N_5O_3$, M_r = 366.41, crystal size 0.36 × 0.24 × 0.12 mm, monoclinic, space group $P2_1$/c (No. 14), a = 11.5196(2) Å, b = 14.6170(2) Å, c = 11.8196(2) Å, β = 101.496(1)°, V = 1950.28(5) Å^3, Z = 4, ρ = 1.248 Mg/m^{-3}, μ(Cu-K$_\alpha$) = 0.70 mm^{-1}, F(000) = 776, $2\theta_{max}$ = 144.4°, T = 298(2) K, 25765 reflections, of which 3836 were independent (R_{int} = 0.024), 318 parameters, 646 restraints (see cif-file for details), R_1 = 0.044 (for 3513 I > 2σ(I)), wR_2 = 0.130 (all data), S = 1.03, largest diff. peak / hole = 0.21 / -0.18 e Å^{-3}.

***Z*-6**

SB1336_HY: yellow crystals, $C_{21}H_{24}N_4O_4$, M_r = 396.44, crystal size 0.14 × 0.10 × 0.06 mm, monoclinic, space group $P2_1$/c (No. 14), a = 11.2851(4) Å, b = 15.2971(5) Å, c = 11.6703(4) Å, β = 99.474(1)°, V = 1987.16(12) Å^3, Z = 4, ρ = 1.325 Mg/m^{-3}, μ(Cu-K$_\alpha$) = 0.77 mm^{-1}, F(000) = 840, $2\theta_{max}$ = 144.6°, T = 123(2) K, 29490 reflections, of which 3924 were independent (R_{int} = 0.027), 274 parameters, 3 restraints, R_1 = 0.032 (for 3736 I > 2σ(I)), wR_2 = 0.085 (all data), S = 1.03, largest diff. peak / hole = 0.30 / -0.18 e Å^{-3}.

***Z*-7**

SB1440_HY_SUK170: colourless crystals, $C_{13}H_{12}N_2O_3$, M_r = 244.25, crystal size 0.36 × 0.18 × 0.06 mm, monoclinic, space group $P2_1$ (No. 4), a = 7.2931(5) Å, b = 23.1044(16) Å, c = 13.5985(9) Å, β = 90.424(4)°, V = 2291.3(3) Å^3, Z = 8, ρ = 1.416 Mg/m^{-3}, μ(Cu-K$_\alpha$) = 0.85 mm^{-1}, F(000) = 1024, $2\theta_{max}$ = 146.2°, T = 298(2) K, 46761 reflections, of which 8936 were independent (R_{int} = 0.061), 654 parameters, 541 restraints (general RIGU restraint), R_1 = 0.060 (for 8174 I > 2σ(I)), wR_2 = 0.165 (all data), S = 1.06, largest diff. peak / hole = 0.29 / -0.21 e Å^{-3}, twin,

absolute structure cannot be determined reliably (x = 0.23(9)). Due to the bad quality of the data the structure could be only used as a proof.

***Z*-8**

SB1440_HY: colourless crystals, $C_{14}H_{14}N_2O_4$, M_r = 274.27, crystal size 0.16 × 0.08 × 0.04 mm, triclinic, space group *P*-1 (No. 2), *a* = 6.6984(2) Å, *b* = 7.3720(2) Å, *c* = 13.9541(3) Å, *α* = 82.063(1)°, *β* = 89.009(1)°, *γ* = 74.886(1)°, *V* = 658.73(3) Å^3, *Z* = 2, *ρ* = 1.383 Mg/m^{-3}, μ(Cu-K$_\alpha$) = 0.86 mm^{-1}, *F*(000) = 288, $2\theta_{max}$ = 144.6°, *T* = 298(2) K, 9756 reflections, of which 2584 were independent (R_{int} = 0.023), 186 parameters, 1 restraint, R_1 = 0.038 (for 2336 I > 2σ(I)), wR_2 = 0.110 (all data), *S* = 1.08, largest diff. peak / hole = 0.16 / -0.16 e Å^{-3}.

***Z*-11**

SB1393_HY: yellow crystals, $C_{15}H_{17}N_3O_3$, M_r = 287.32, crystal size 0.24 × 0.24 × 0.06 mm, monoclinic, space group *P*2$_1$/n (No. 14), *a* = 11.9836(3) Å, *b* = 7.9926(2) Å, *c* = 15.3272(4) Å, *β* = 104.010(1)°, *V* = 1424.37(6) Å^3, *Z* = 4, *ρ* = 1.340 Mg/m^{-3}, μ(Cu-K$_\alpha$) = 0.78 mm^{-1}, *F*(000) = 608, $2\theta_{max}$ = 144.6°, *T* = 173(2) K, 13451 reflections, of which 2789 were independent (R_{int} = 0.022), 196 parameters, 1 restraint, R_1 = 0.037 (for 2644 I > 2σ(I)), wR_2 = 0.102 (all data), *S* = 1.05, largest diff. peak / hole = 0.22 / -0.23 e Å^{-3}.

***Z*-13**

SB1440_HY_SUK130: colourless crystals, $C_{14}H_{11}N_3O_3 \cdot 2\ H_2O$, M_r = 305.29, crystal size 0.16 × 0.08 × 0.04 mm, triclinic, space group *P*-1 (No. 2), *a* = 7.4710(17) Å, *b* = 8.0460(17) Å, *c* = 12.747(3) Å, *α* = 80.540(15)°, *β* = 74.438(15)°, *γ* = 86.632(15)°, *V* = 728.0(3) Å^3, *Z* = 2, *ρ* = 1.393 Mg/m^{-3}, μ(Cu-K$_\alpha$) = 0.91 mm^{-1}, *F*(000) = 320, $2\theta_{max}$ = 144.4°, *T* = 298(2) K, 4516 reflections, of which 2736 were independent (R_{int} = 0.047), 212 parameters, 6 restraints, R_1 = 0.077 (for 2100 I > 2σ(I)), wR_2 = 0.221 (all data), *S* = 1.08, largest diff. peak / hole = 0.28 / -0.28 e Å^{-3}. Due to the bad quality of the crystal, incomplete data and possible twinning only the structure could be confirmed.

***Z*-15**

SB1368_HY: yellow crystals, $C_{19}H_{18}N_4O_2$, M_r = 334.37, crystal size 0.18 × 0.04 × 0.02 mm, monoclinic, space group *P*2$_1$/c (No. 14), *a* = 7.1588(3) Å, *b* = 15.0290(6) Å, *c* = 14.9109(6) Å, *β* = 95.951(2)°, *V* = 1595.61(11) Å^3, *Z* = 4, *ρ* = 1.392 Mg/m^{-3}, μ(Cu-K$_\alpha$) = 0.76 mm^{-1}, *F*(000) = 704, $2\theta_{max}$ = 144.6°, *T* = 173(2) K, 11533 collected reflections, which were merged to 3119 independent reflection (HKLF 5 file, R_{int} = 0.000, see cif-file for details. Module TwinRotMat of the program package PLATON: A. L. Spek,. *Acta Crystallogr. 2009*, **D65**, 148-155. A. L. Spek,. *Acta Crystallogr. 2015*, **C71**, 9-18.), 233 parameters, 2 restraints, R_1 = 0.068 (for 2743 I > 2σ(I)), wR_2 = 0.163 (all data), *S* = 1.18, largest diff. peak / hole = 0.28 / -0.35 e Å^{-3}. The structure is refined a twin with 2 domains.

19

SB1376_HY: colourless crystals, $C_{13}H_{10}N_2O_3$, M_r = 242.23, crystal size 0.18 × 0.12 × 0.02 mm, orthorhombic, space group *P*bca (No. 61), *a* = 15.4147(2) Å, *b* = 6.1889(1) Å, *c* = 23.7010(3) Å, *V* = 2261.08(5) Å^3, *Z* = 8, *ρ* = 1.423 Mg/m^{-3}, μ(Cu-K$_\alpha$) = 0.86 mm^{-1}, *F*(000) = 1008, $2\theta_{max}$ = 144.2°, *T* = 298(2) K, 36061 reflections, of which 2223 were independent (R_{int} = 0.039), 164

parameters, R_1 = 0.038 (for 1986 I > 2σ(I)), wR_2 = 0.103 (all data), S = 1.05, largest diff. peak / hole = 0.15 / -0.17 e Å^{-3}.

The Cambridge Crystallographic Data Centre (CCDC, https://www.ccdc.cam.ac.uk/structures/) entries CCDC 2076713 (*Z*-**2**, SB1177_HY), 2076714 (*Z*-**3**, SB1192_HY), 2076715 (*E*-**3**, SB1354_HY), 2076716 (*Z*-**4**, SB1373_HY), 2076717 (*Z*-**5**, SB1342_HY), 2076718 (*Z*-**6**, SB1336_HY), 2177720 (*Z*-**7**, SB1440_HY_SUK170), 2177721 (*Z*-**13**, SB1440_HY_SUK130), 2177722 (*Z*-**11**, SB1393_HY), 2177723 (*Z*-**8**, SBSB1440_HY), 2177724 (**19**, SB1376_HY), and 2177725 (*Z*-**15**, SB1368_HY) contain the supplementary crystallographic data for this paper. These data can be also obtained free of charge from the Chemotion repository https://www.chemotion-repository.net under the following links:

Z-**2** https://dx.doi.org/10.14272/JORKNWYVOQPNQP-QOOFZUOPSA-N/CHMO0000156
Z-**3** https://dx.doi.org/10.14272/LMVUWTHIXFBXBY-UOUVAZQYSA-N.1
E-**3** https://dx.doi.org/10.14272/LMVUWTHIXFBXBY-GOBHWCIESA-N.2
Z-**4** https://dx.doi.org/10.14272/PIABYVZMMACTIP-APGQMXJTSA-N.1
Z-**5** https://dx.doi.org/10.14272/AIJQSTUPFONWLS-VULZFCBJSA-N.1
Z-**6** https://dx.doi.org/10.14272/OTVHUVBFCLOWCA-KPJFGDCZSA-N.1
Z-**7** https://dx.doi.org/10.14272/MJCOATFQVUUHFN-XFFZJAGNSA-N.1
Z-**8** https://dx.doi.org/10.14272/DHTWSXQNUMZDAD-GHXNOFRVSA-N.1
Z-**11** https://dx.doi.org/10.14272/SYFVNJGPALDWRA-JYRVWZFOSA-N.1
Z-**13** https://dx.doi.org/10.14272/DDZMJSZYMNIGEY-SDQBBNPISA-N.1
Z-**15** https://dx.doi.org/10.14272/HEBNVDMSJJEXNA-LCYFTJDESA-N/CHMO0000156
19 https://dx.doi.org/10.14272/XEFPBGSACIWODS-UHFFFAOYSA-N.1

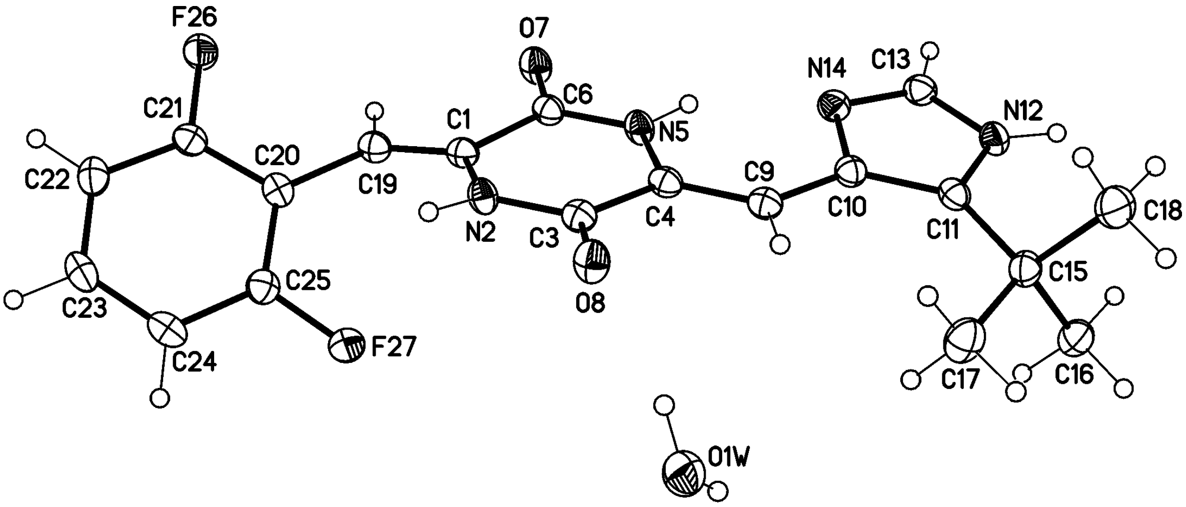

*Supplementary Figure 72: Molecular structure of Z-**2** (CCDC 2076713) (displacement parameters are drawn at 50 % probability level).*

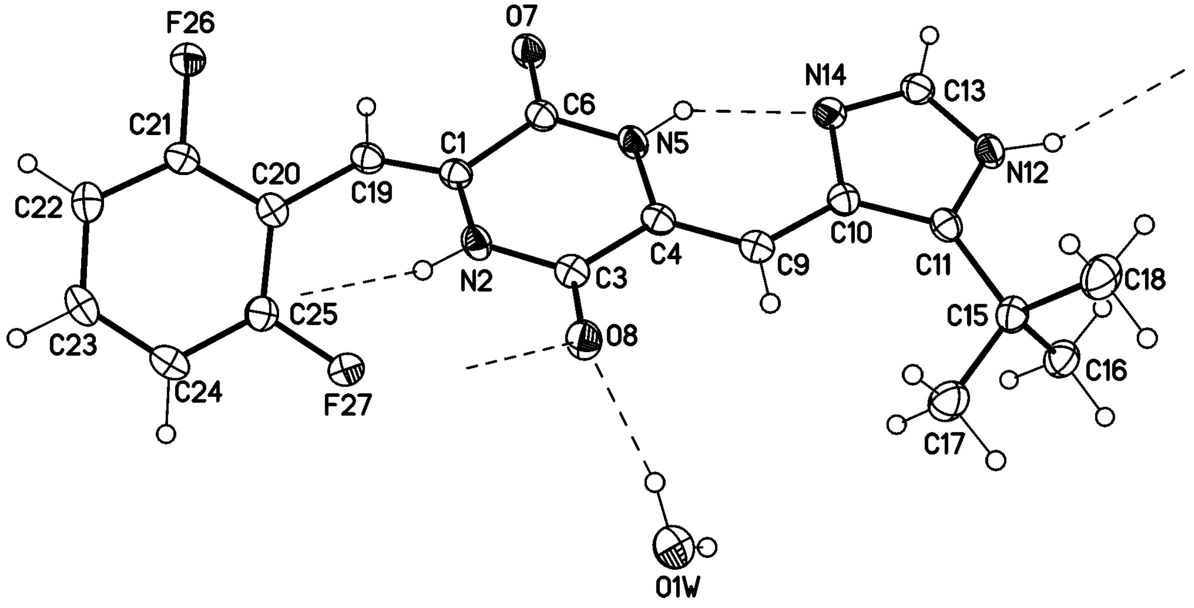

*Supplementary Figure 73: Molecular structure of Z-**2** (CCDC 2076713) showing hydrogen bond pattern (displacement parameters are drawn at 50 % probability level).*

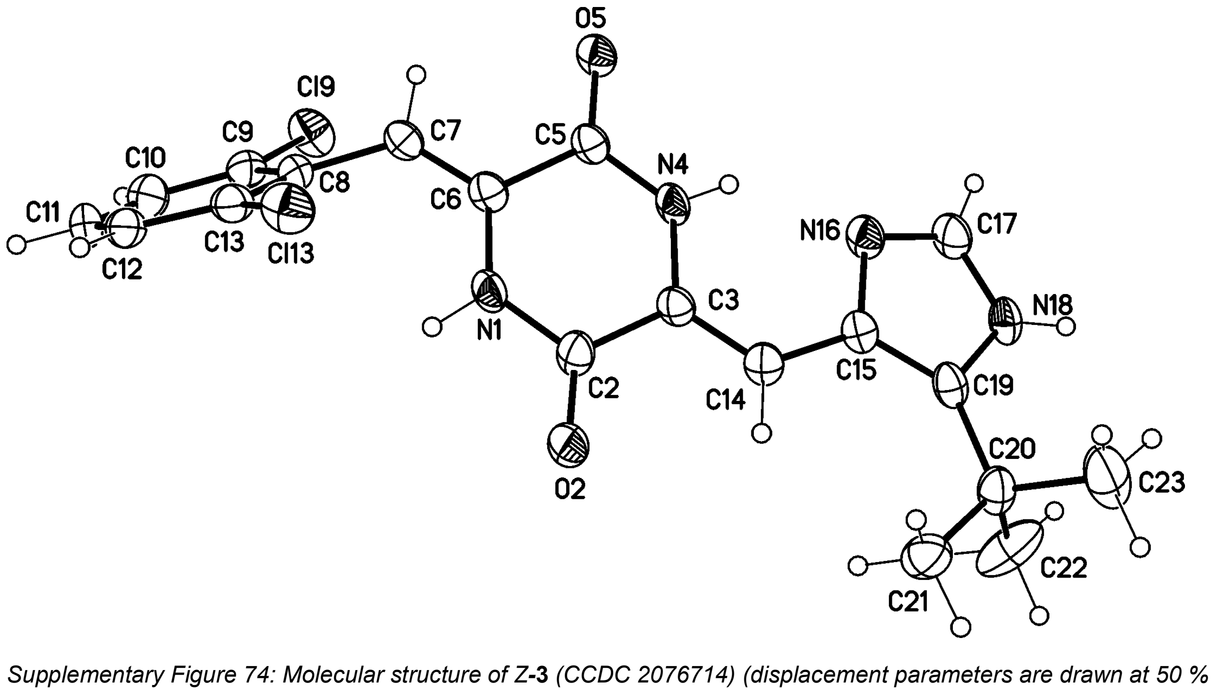

*Supplementary Figure 74: Molecular structure of Z-**3** (CCDC 2076714) (displacement parameters are drawn at 50 % probability level).*

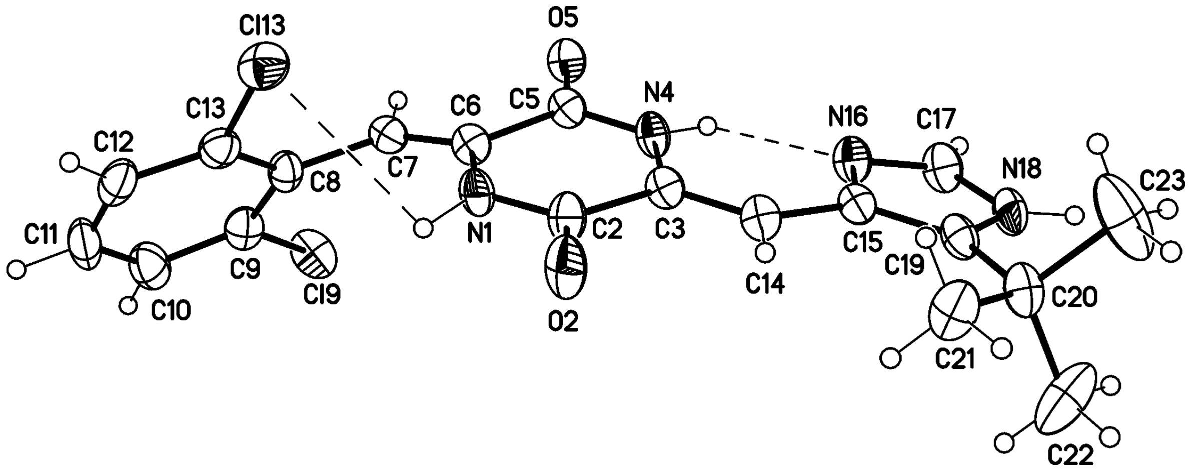

*Supplementary Figure 75: Molecular structure of Z-**3** (CCDC 2076714) showing intramolecular hydrogen bonds (displacement parameters are drawn at 50 % probability level).*

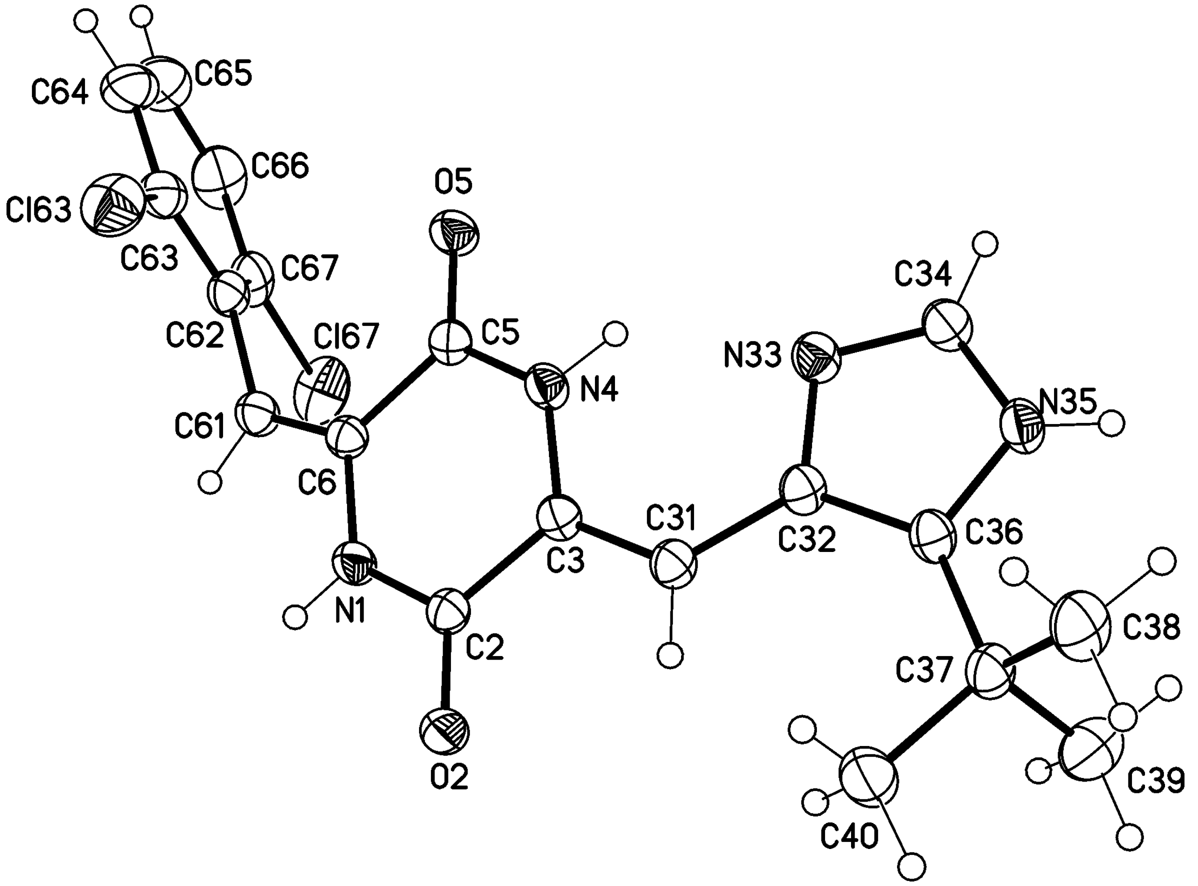

*Supplementary Figure 76: Molecular structure of E-**3** (CCDC 2076715) (displacement parameters are drawn at 30 % probability level).*

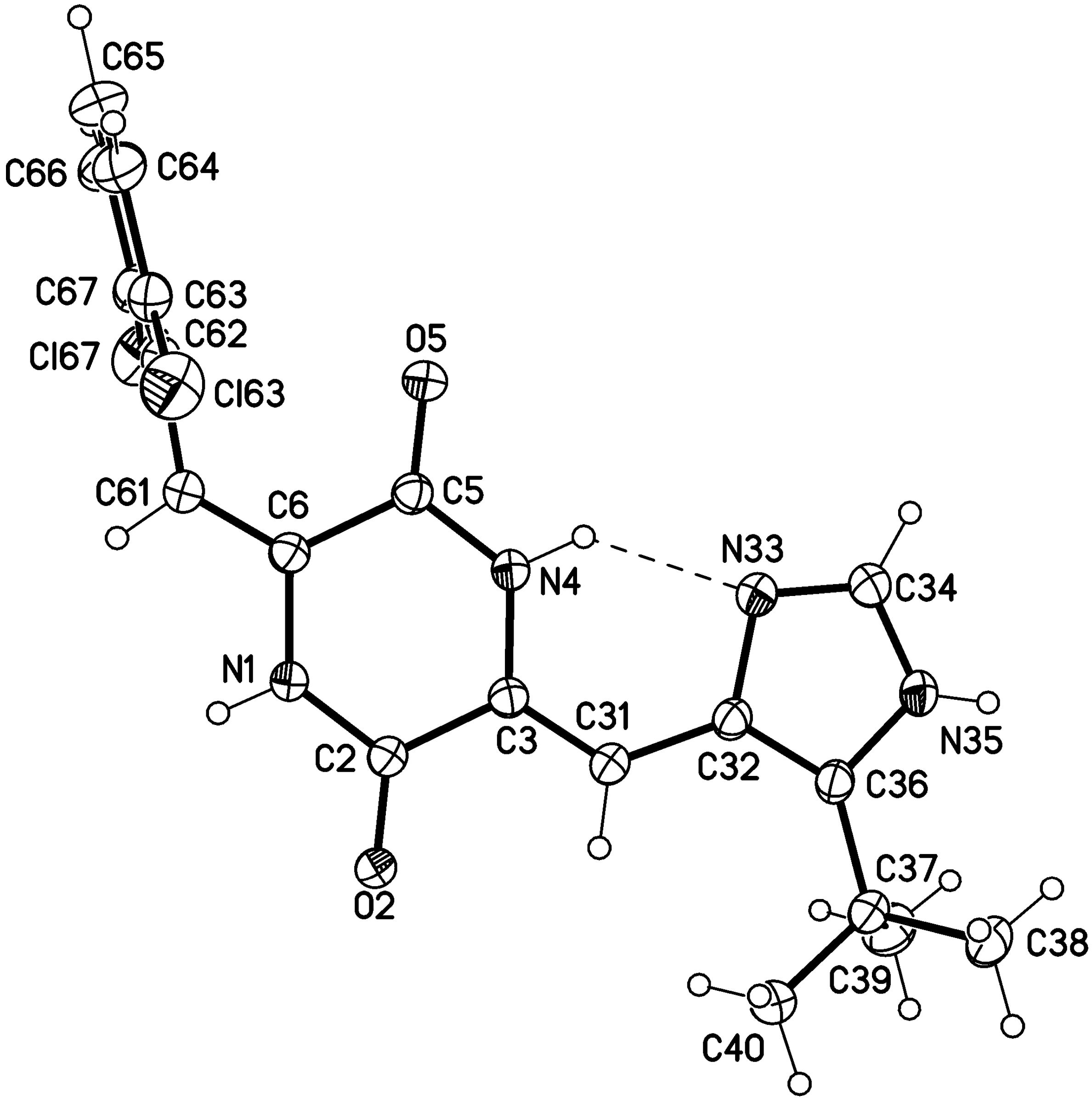

*Supplementary Figure 77: Molecular structure of E-**3** (CCDC 2076715) showing intramolecular hydrogen bond (displacement parameters are drawn at 30 % probability level).*

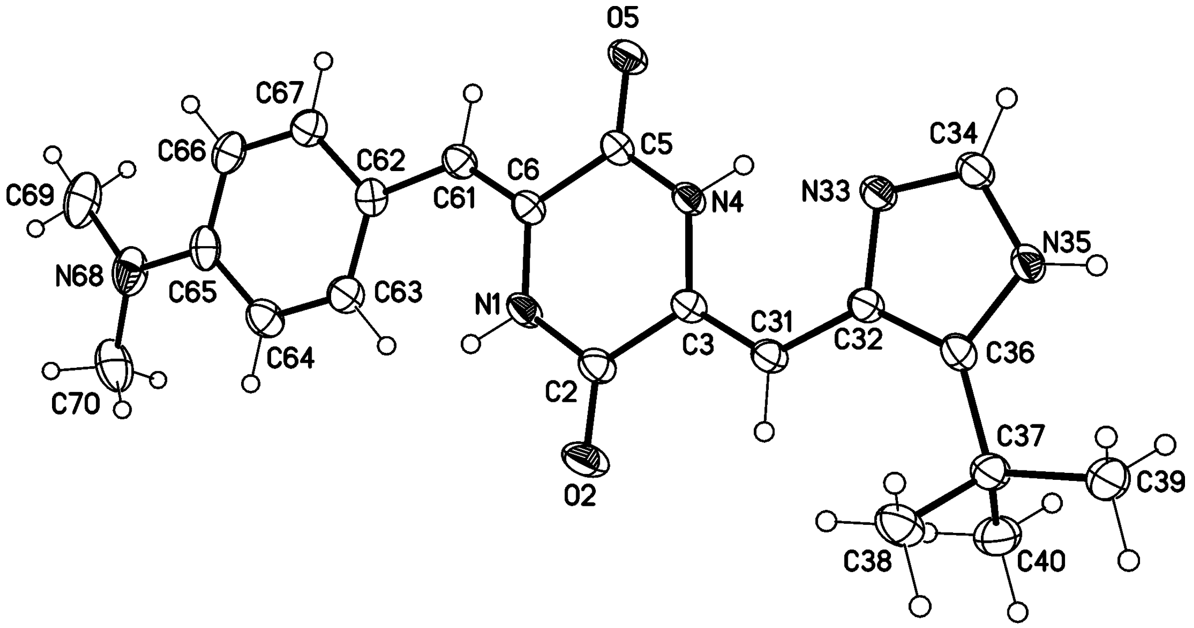

*Supplementary Figure 78: Molecular structure of Z-**4** (CCDC 2076716) (displacement parameters are drawn at 50 % probability level).*

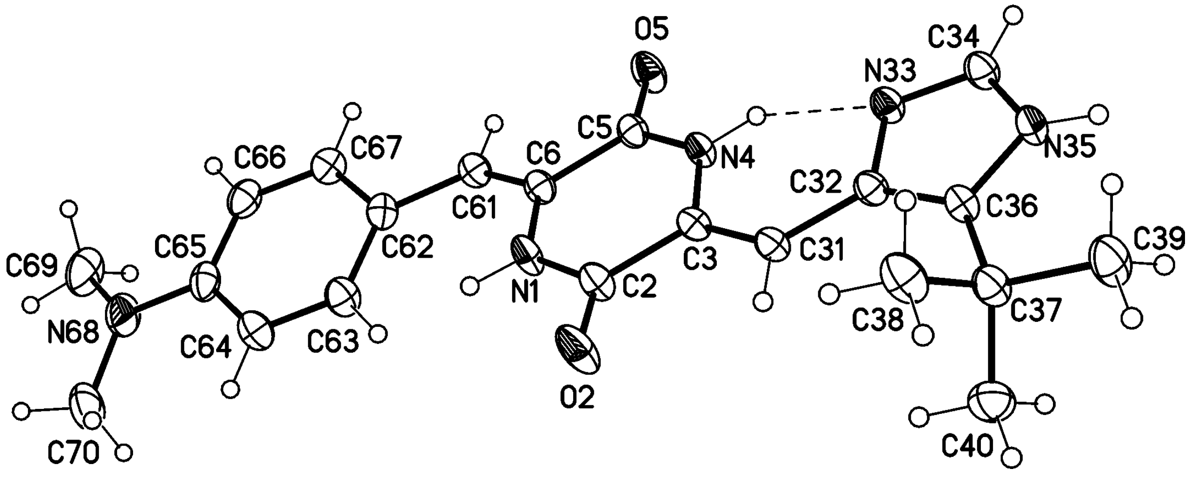

*Supplementary Figure 79: Molecular structure of Z-**4** (CCDC 2076716) showing intramolecular hydrogen bond (displacement parameters are drawn at 50 % probability level).*

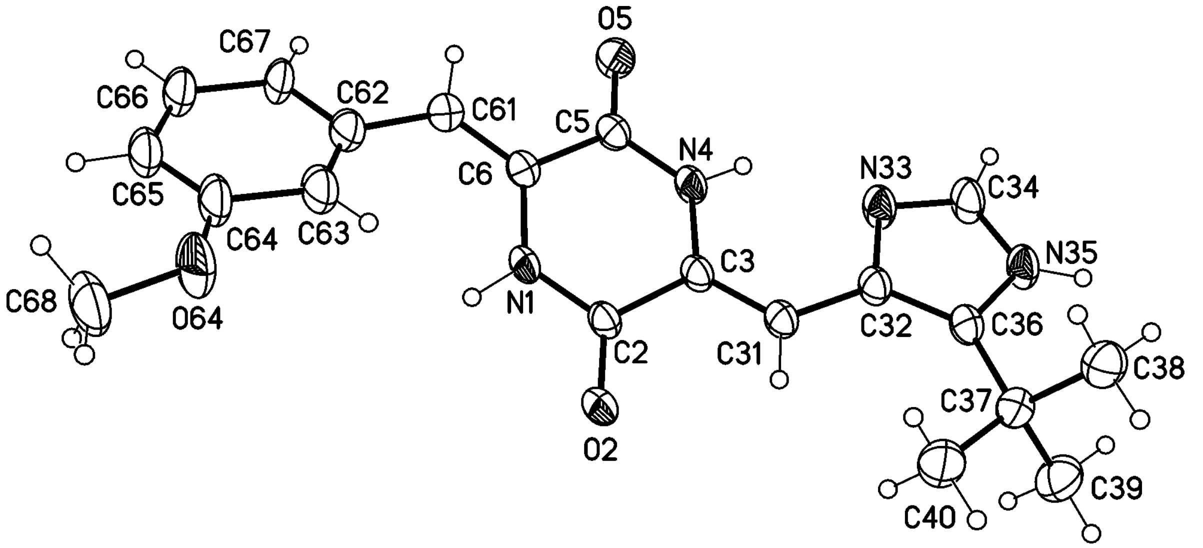

*Supplementary Figure 80: Molecular structure of Z-**5** (CCDC 2076717) (one disordered 3-methoxybenzylidene moiety omitted for clarity, displacement parameters are drawn at 30 % probability level).*

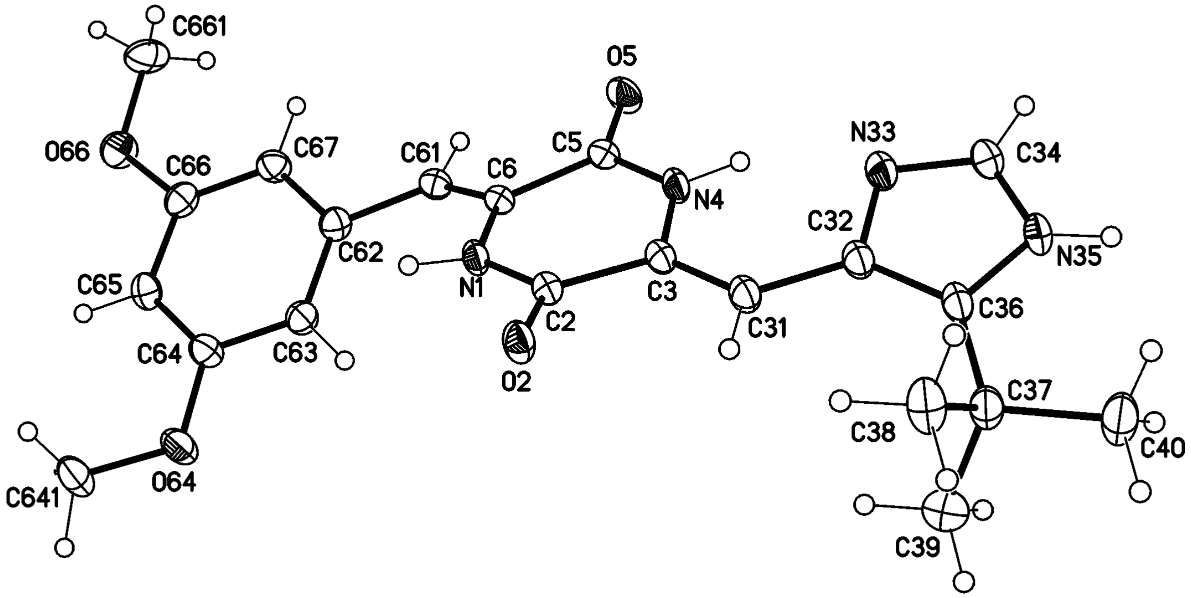

*Supplementary Figure 81: Molecular structure of Z-**6** (CCDC 2076718) (displacement parameters are drawn at 50 % probability level).*

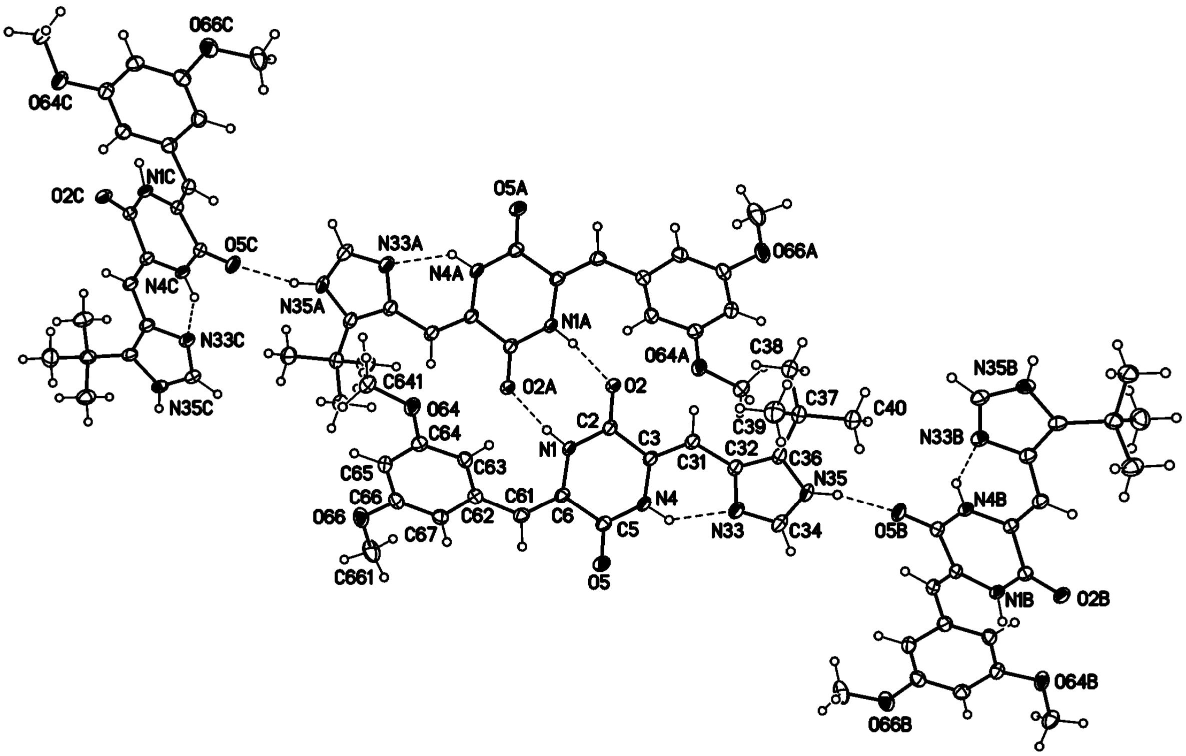

*Supplementary Figure 82: Structure of Z-**6** (CCDC 2076718) showing intra- and intermolecular hydrogen bonds (displacement parameters are drawn at 50 % probability level).*

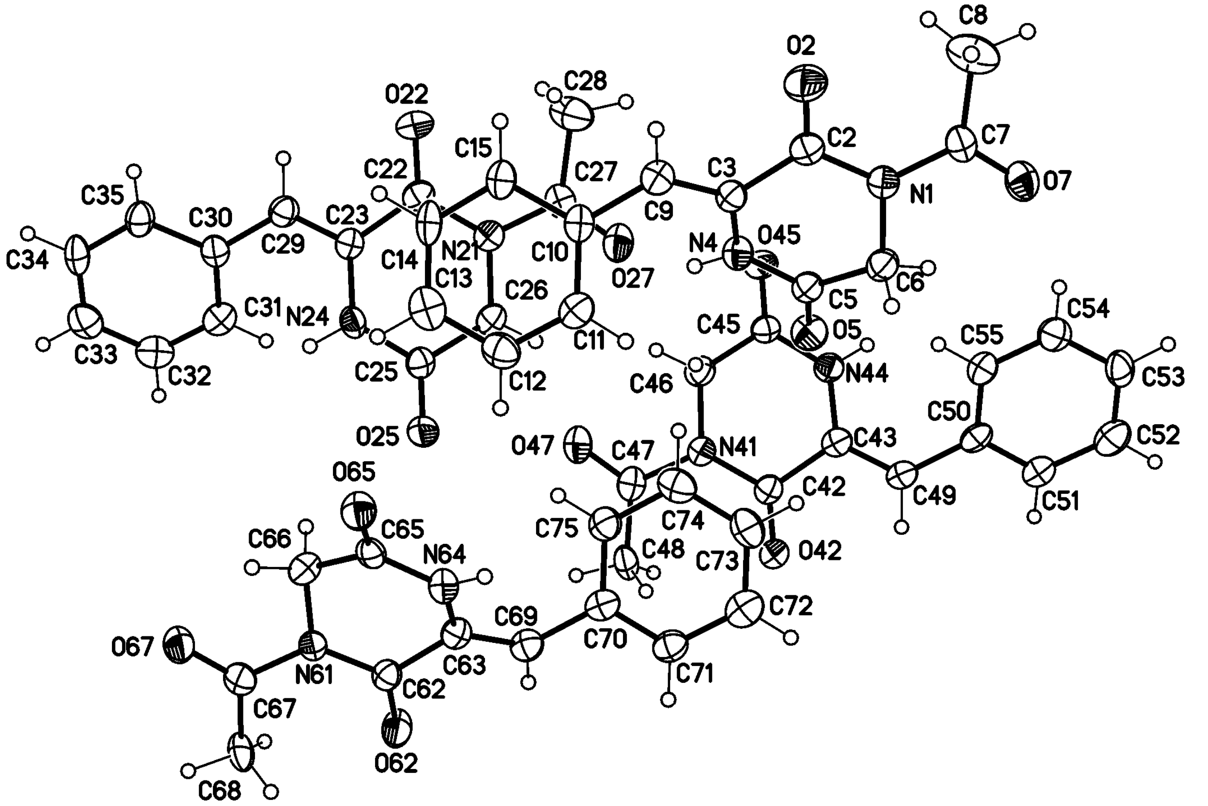

*Supplementary Figure 83: Structure of Z-**7** (CCDC 2177720) (displacement parameters are drawn at 30 % probability level).*

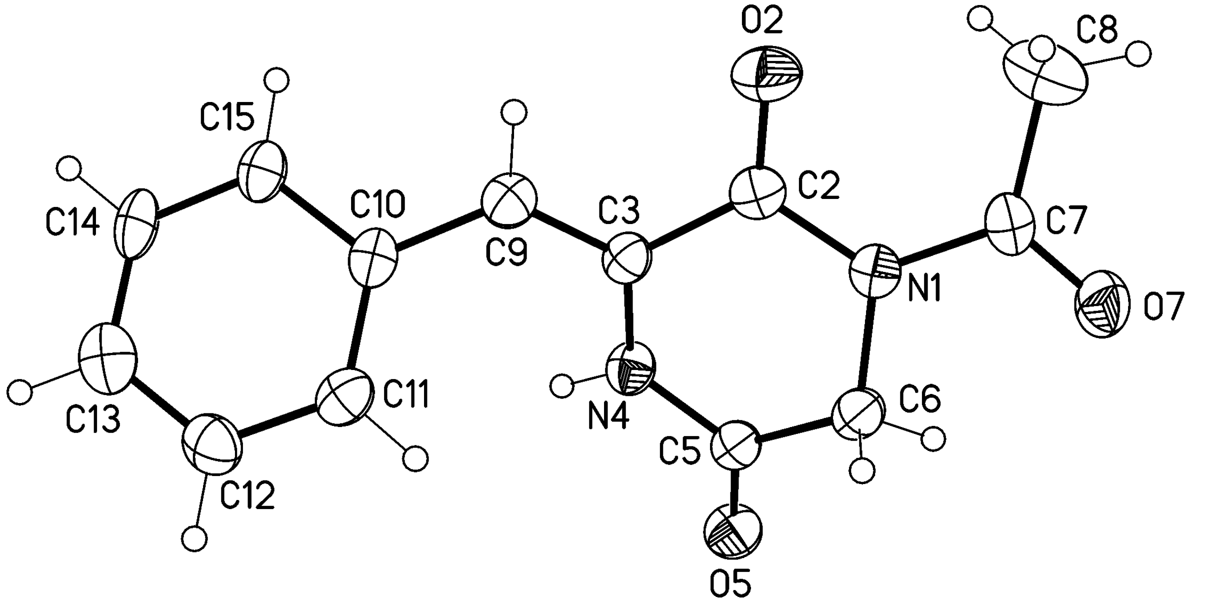

*Supplementary Figure 84: Structure of one crystallographic independent molecule of Z-**7** (CCDC 2177720) (displacement parameters are drawn at 30 % probability level).*

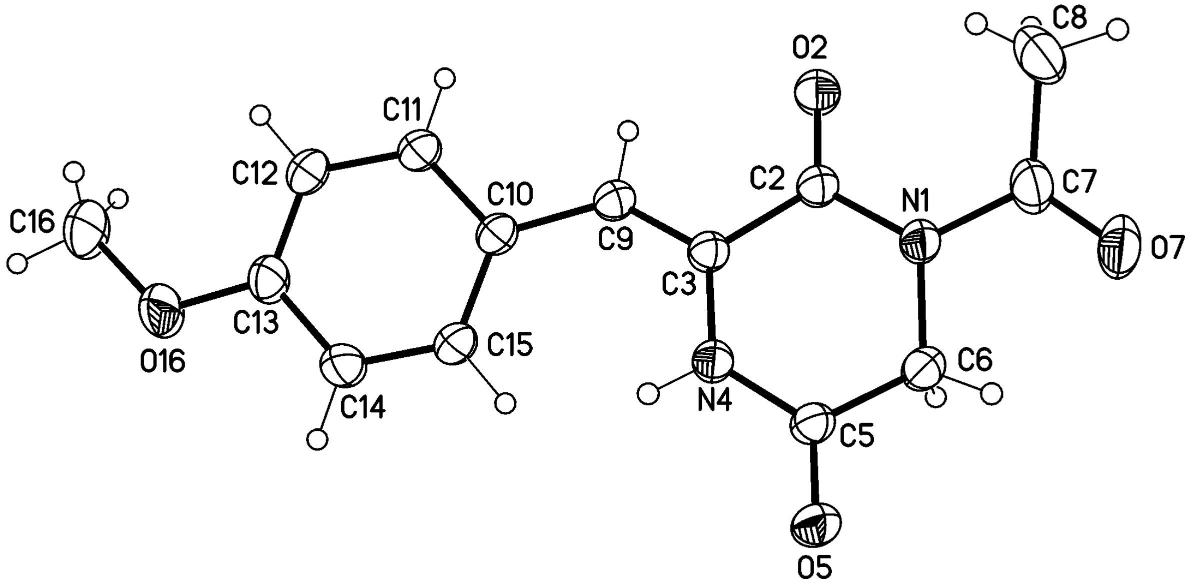

*Supplementary Figure 85: Molecular structure of Z-***8** *(CCDC 2177723) (displacement parameters are drawn at 30 % probability level).*

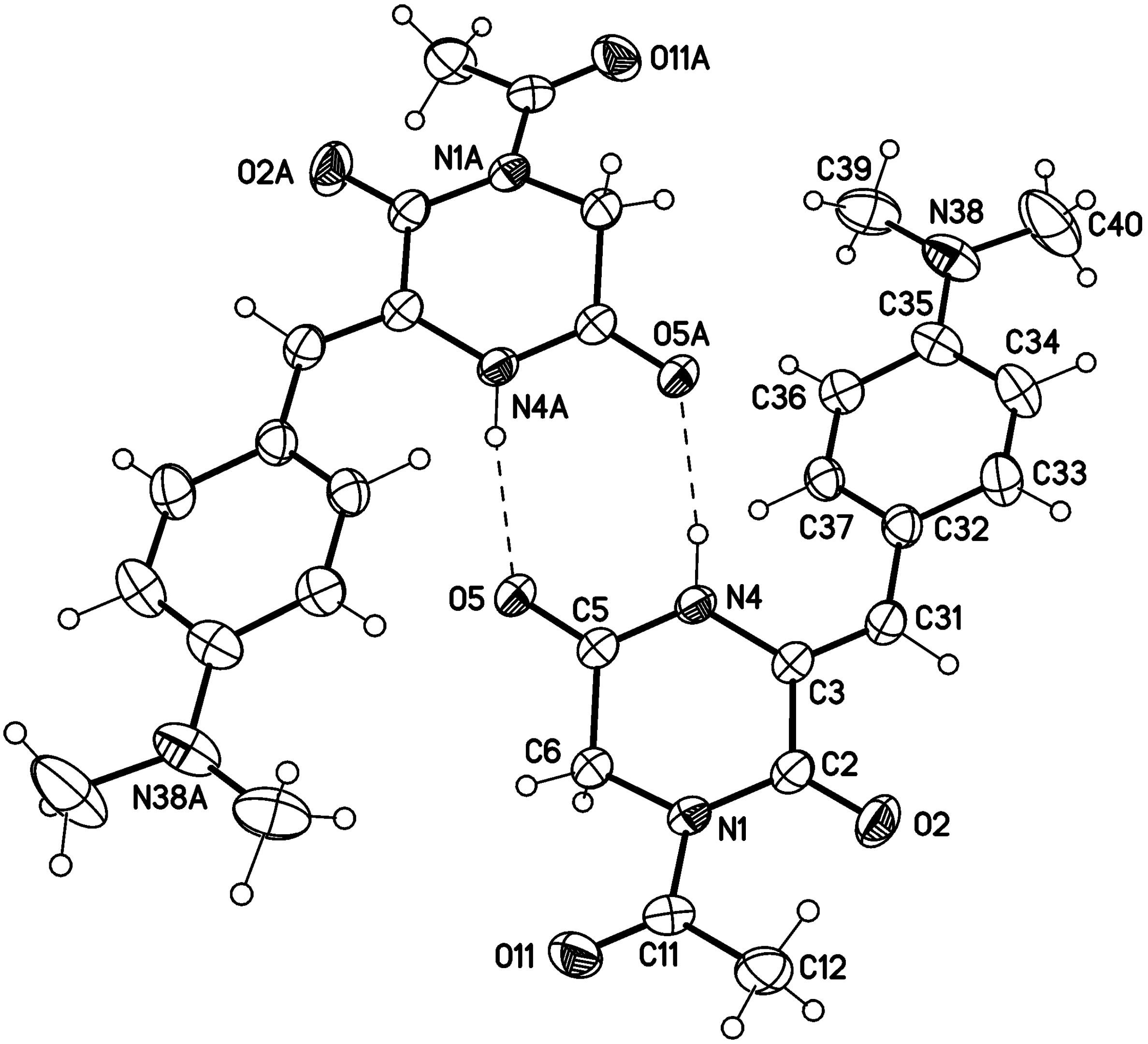

*Supplementary Figure 86: Dimeric structure of Z-***11** *(CCDC 2177722) showing intermolecular hydrogen bonds (displacement parameters are drawn at 50 % probability level).*

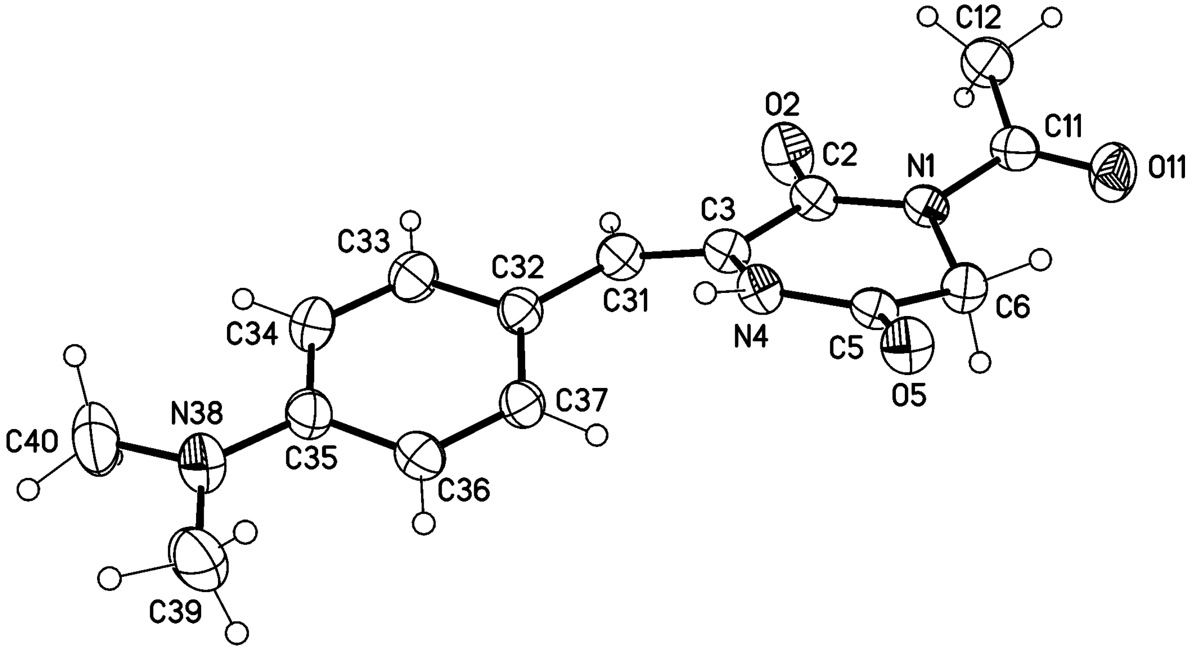

*Supplementary Figure 87: Molecular structure of Z-***11** *(CCDC 2177722) (displacement parameters are drawn at 50 % probability level).*

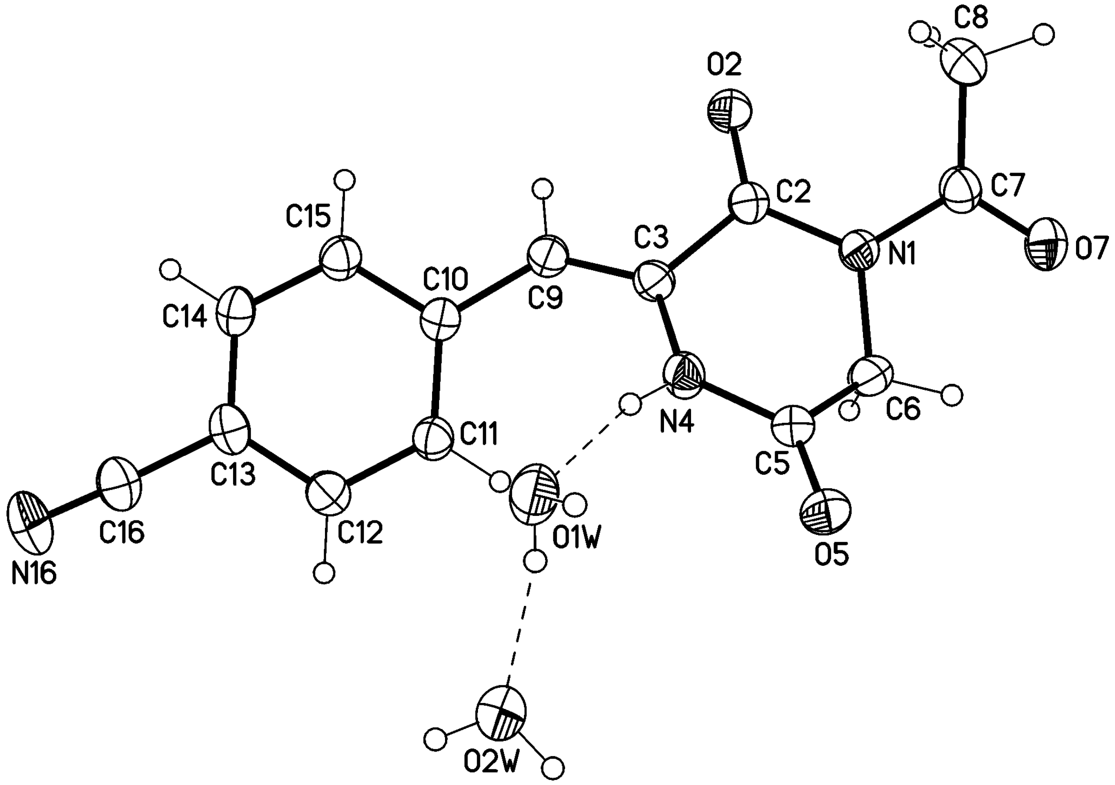

*Supplementary Figure 88: Molecular structure of Z-***13** *(CCDC 2177721) showing intermolecular hydrogen bonds (displacement parameters are drawn at 30 % probability level).*

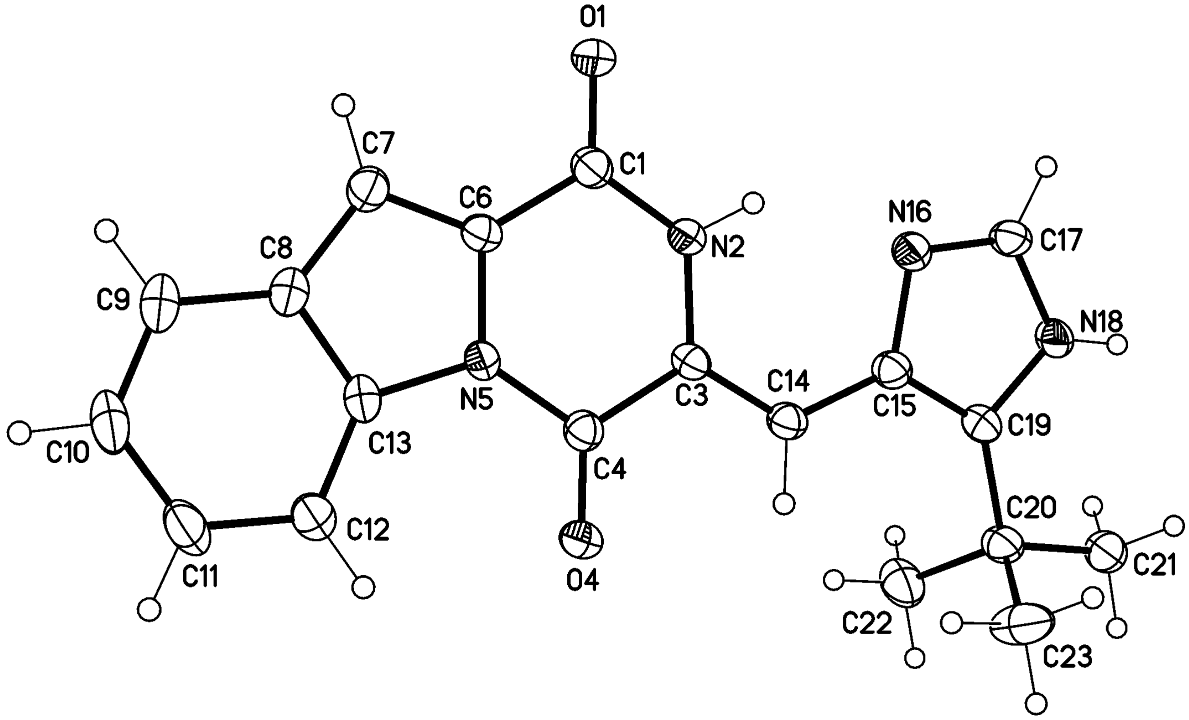

*Supplementary Figure 89: Molecular structure of Z-**15** (CCDC 2177725) (displacement parameters are drawn at 50 % probability level).*

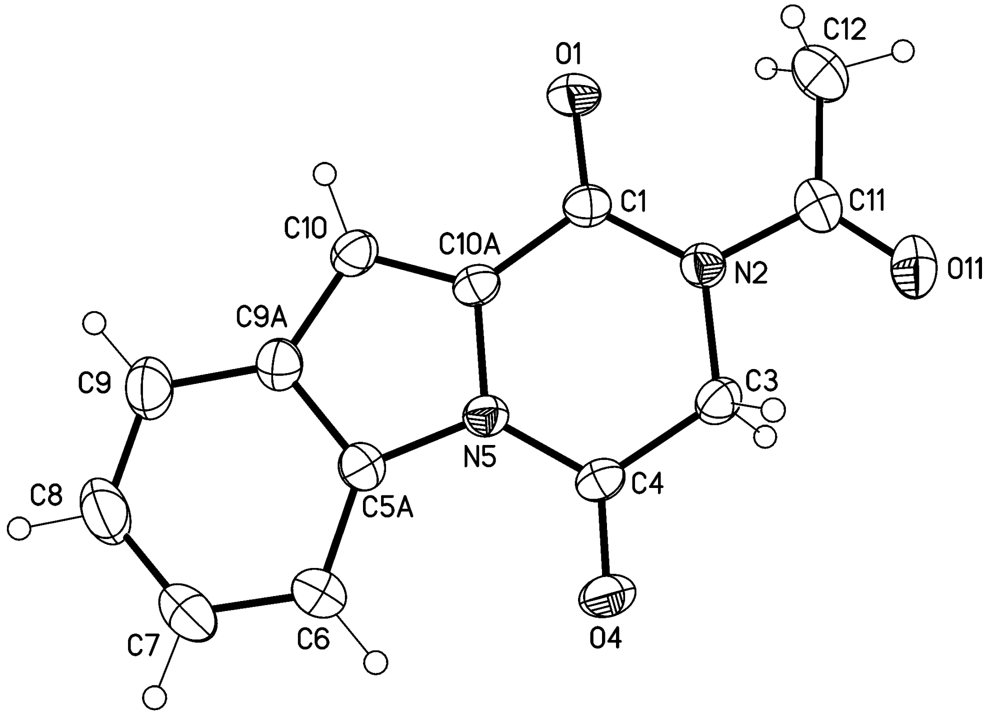

*Supplementary Figure 90: Molecular structure of **19** (CCDC 2177724) (displacement parameters are drawn at 30 % probability level).*

NMR spectra

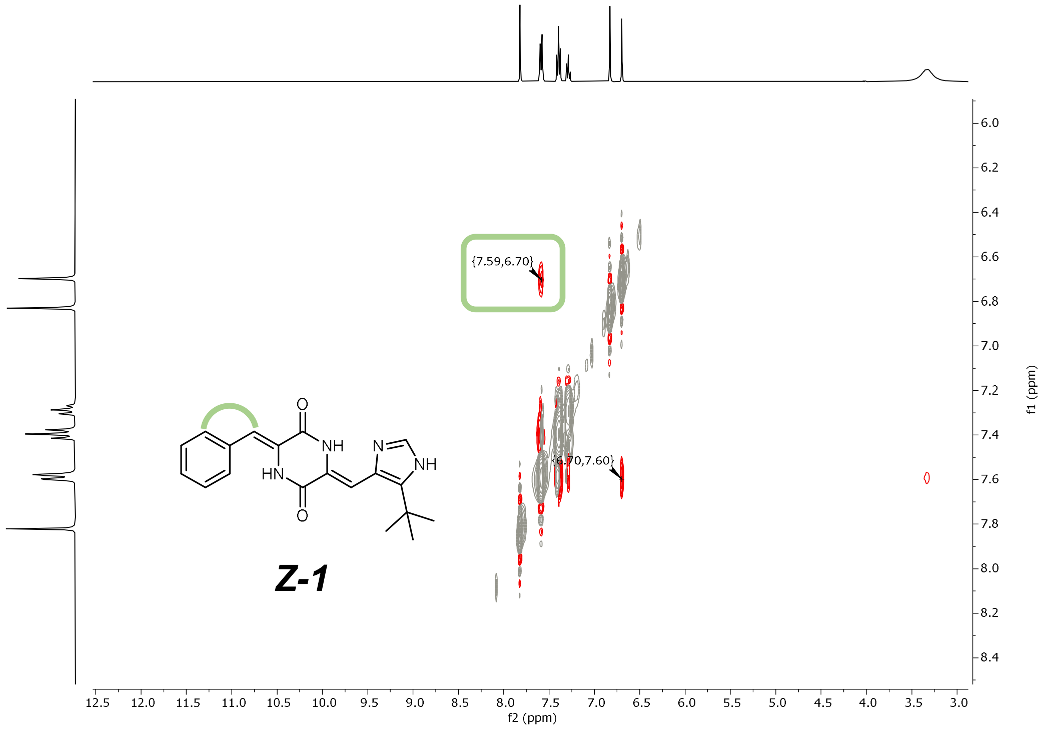

*Supplementary Figure 91: Extract of NOESY NMR spectra (400 MHz, DMSO-d_6) of the photoisomer **Z-1**. The relevant signal was assigned to the corresponding proton via color code.*

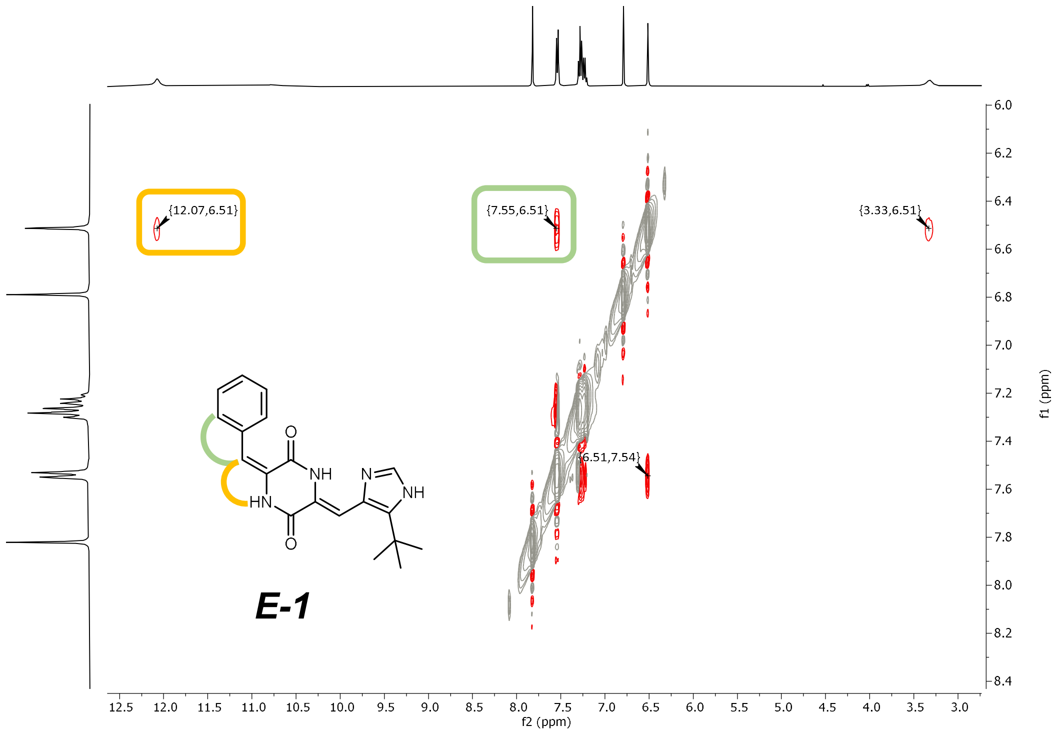

*Supplementary Figure 92: Extract of NOESY NMR spectra (400 MHz, DMSO-d_6) of the photoisomer **E-1**. Several relevant signals were assigned to the corresponding protons via color code.*

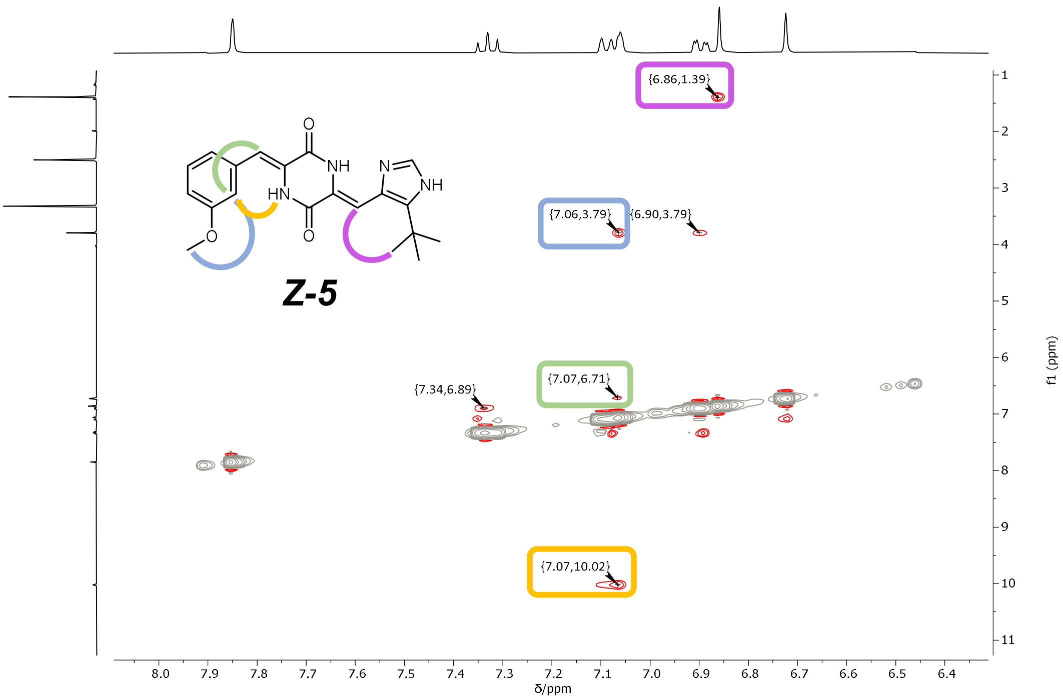

Supplementary Figure 93: Extract of NOESY NMR spectra (400 MHz, DMSO-d_6) of the photoisomer ***Z*-5***. Several relevant signals were assigned to the corresponding protons via color code.*

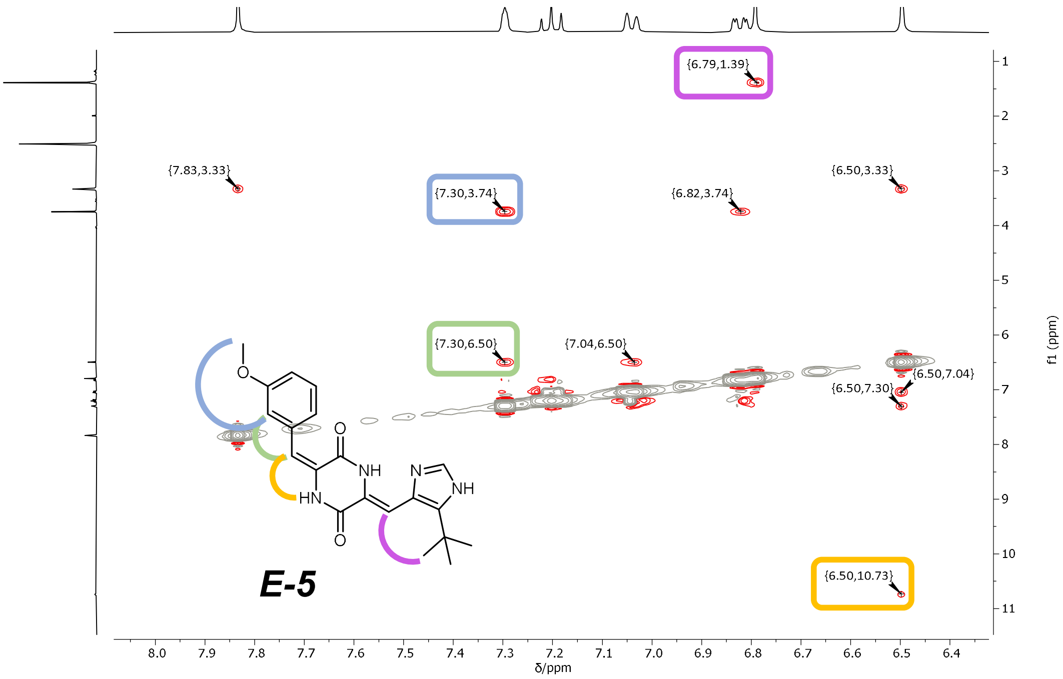

Supplementary Figure 94: Extract of NOESY NMR spectra (400 MHz, DMSO-d_6) of the photoisomer ***E*-5***. Several relevant signals were assigned to the corresponding protons via color code.*

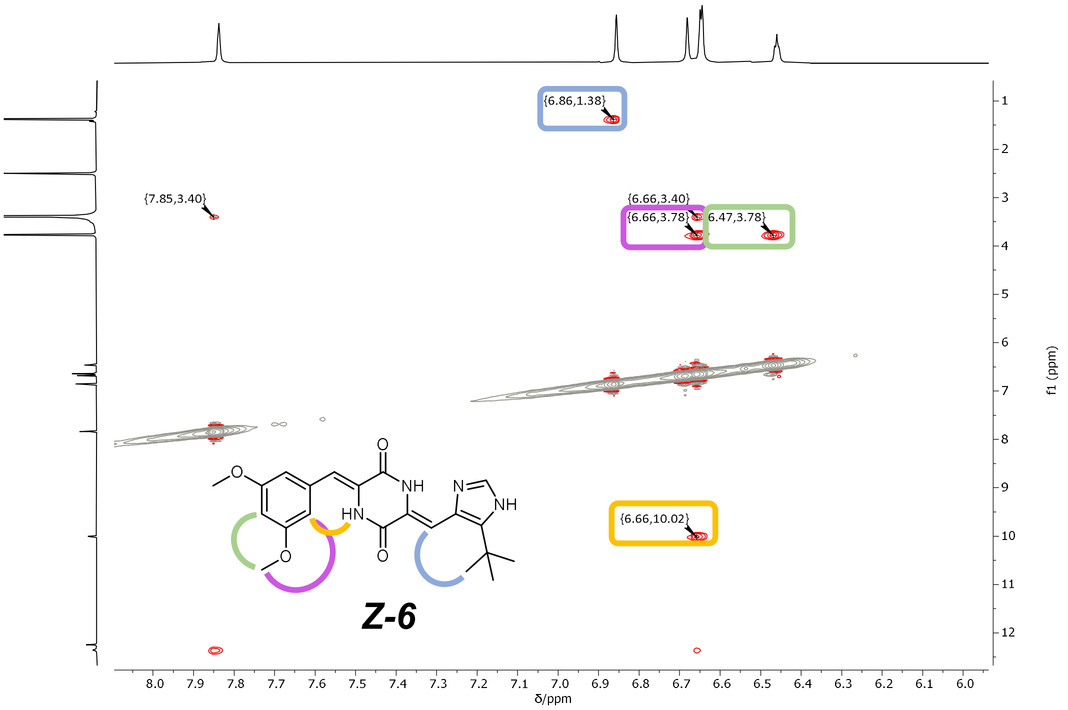

Supplementary Figure 95: *Extract of NOESY NMR spectra (400 MHz, DMSO-d6) of the photoisomer **Z-6**. Several relevant signals were assigned to the corresponding protons via color code.*

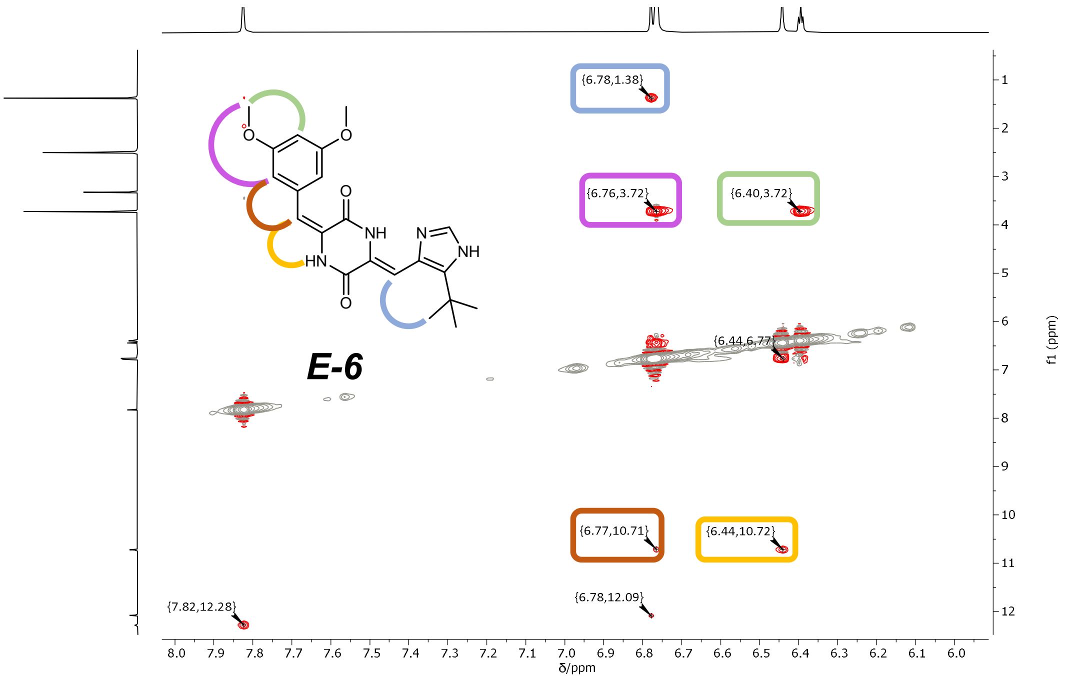

Supplementary Figure 96: *Extract of NOESY NMR spectra (400 MHz, DMSO-d6) of the photoisomer **E-6**. Several relevant signals were assigned to the corresponding protons via color code.*

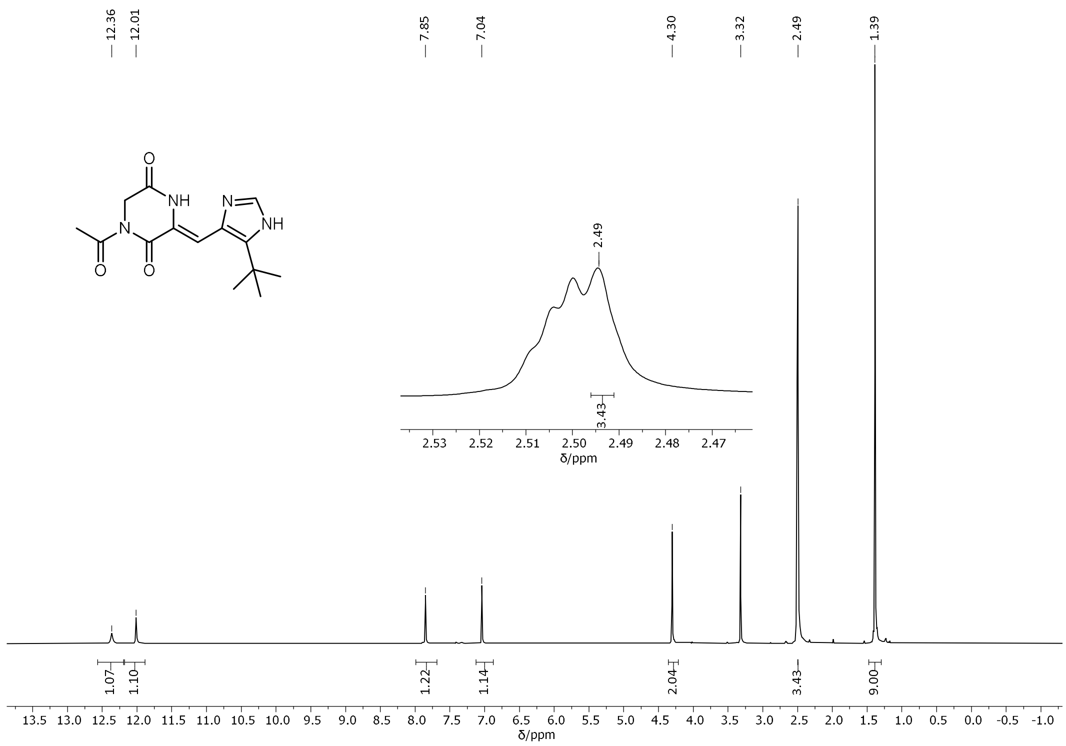

*Supplementary Figure 97: ^{1}H NMR spectrum (400 MHz, DMSO-d$_6$) of compound Z-**14**.*

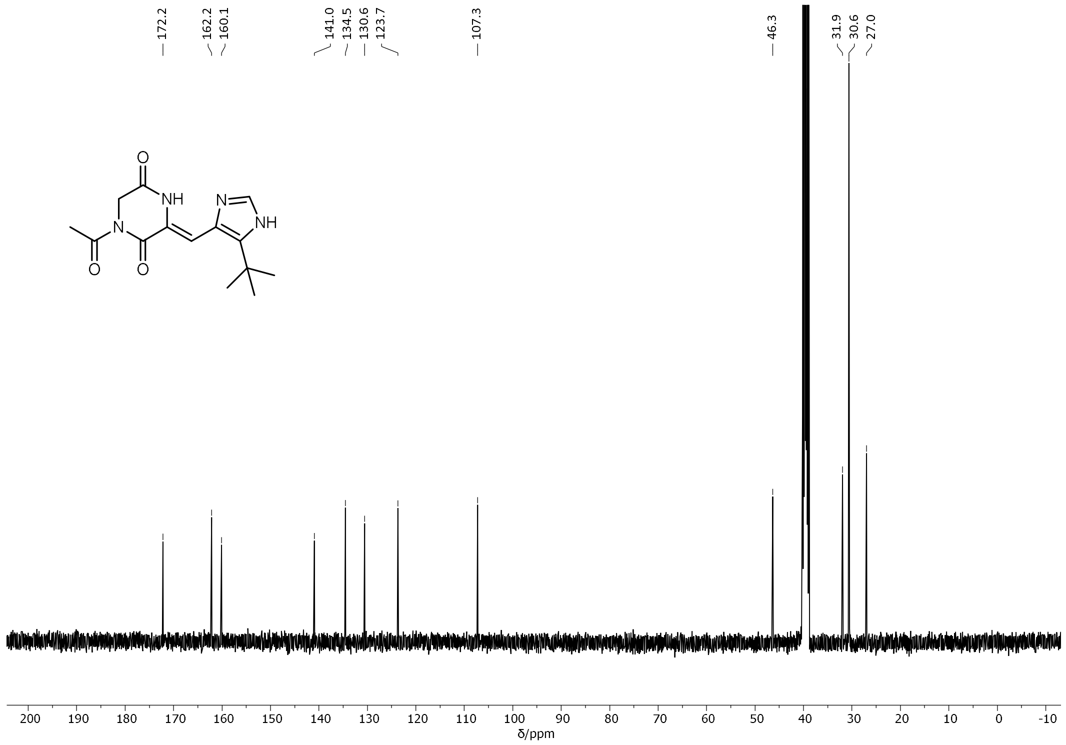

*Supplementary Figure 98: ^{13}C NMR spectrum (101 MHz, DMSO-d$_6$) of compound Z-**14**.*

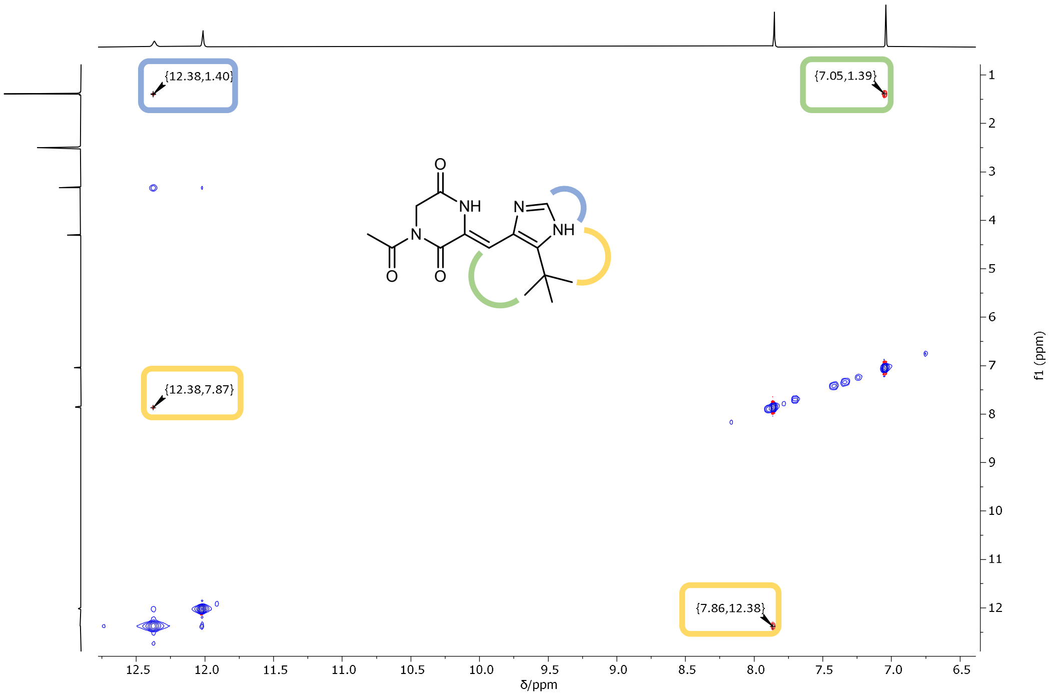

*Supplementary Figure 99: NOESY NMR spectrum (400 MHz, DMSO-d6) of compound Z-**14**.*

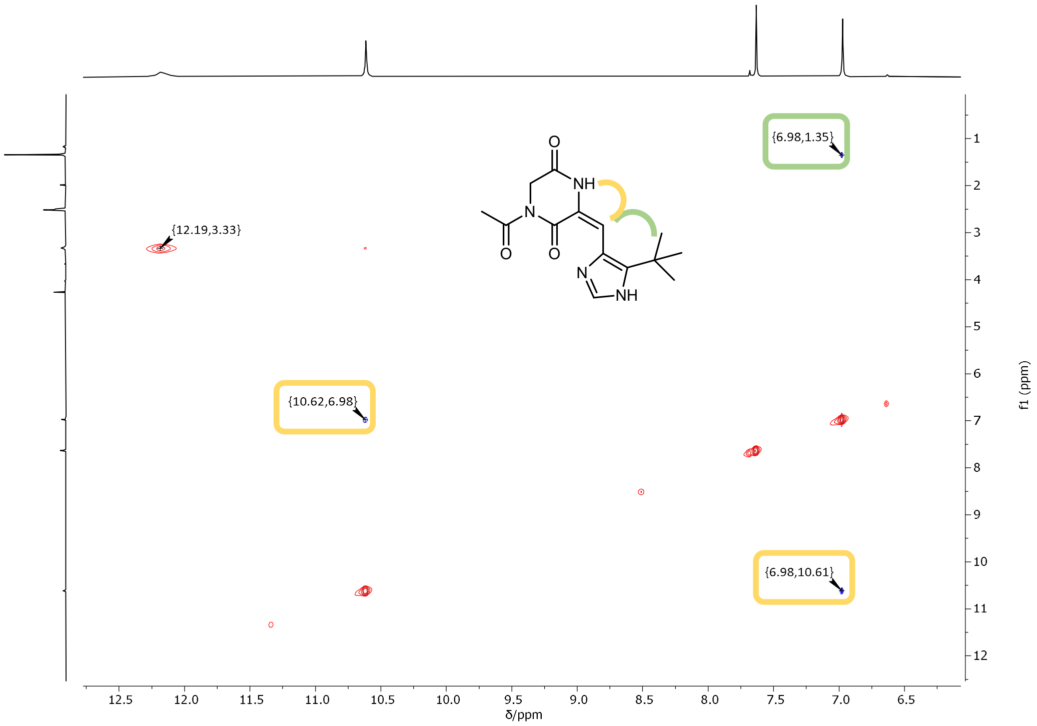

*Supplementary Figure 100: NOESY NMR spectrum (400 MHz, DMSO-d6) of compound E-**14**.*

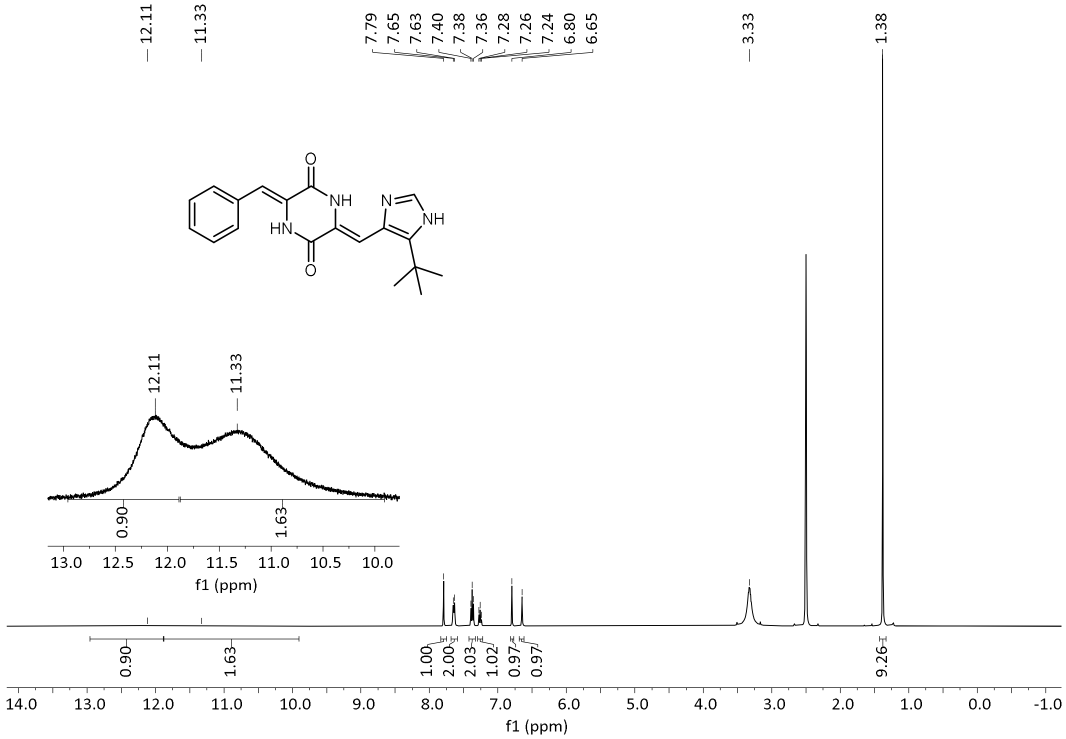

Supplementary Figure 101: ^{1}H NMR spectrum (400 MHz, DMSO-d_6) of compound Z-**1**.

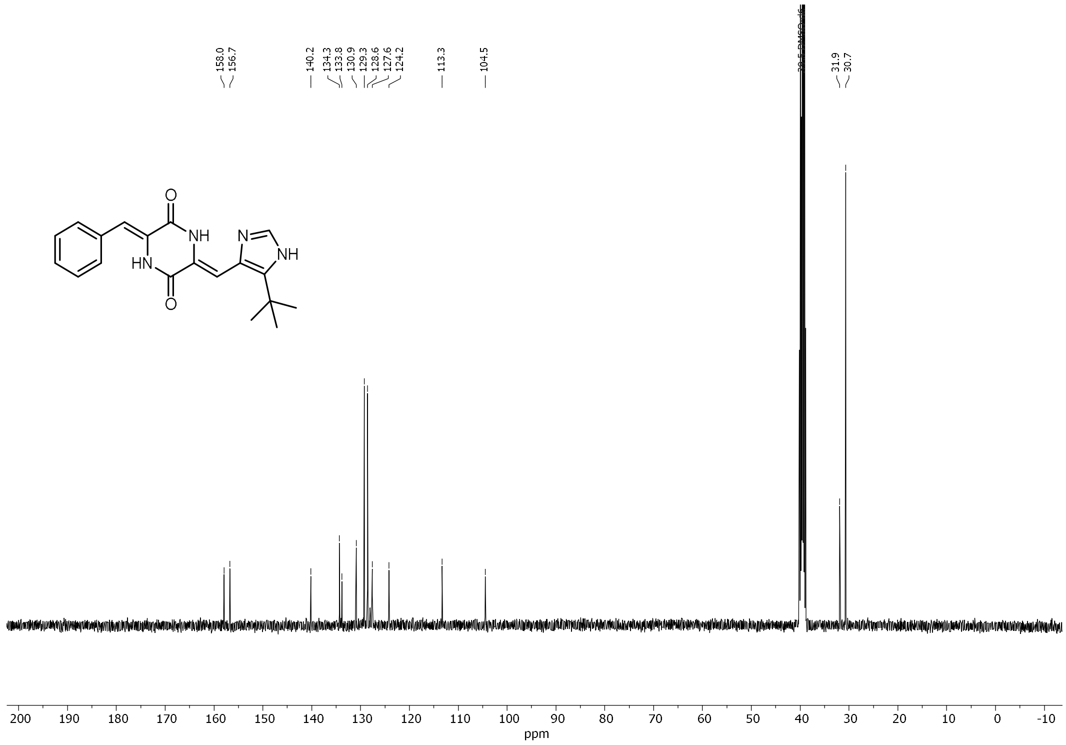

Supplementary Figure 102: ^{13}C NMR spectrum (101 MHz, DMSO-d_6) of compound Z-**1**.

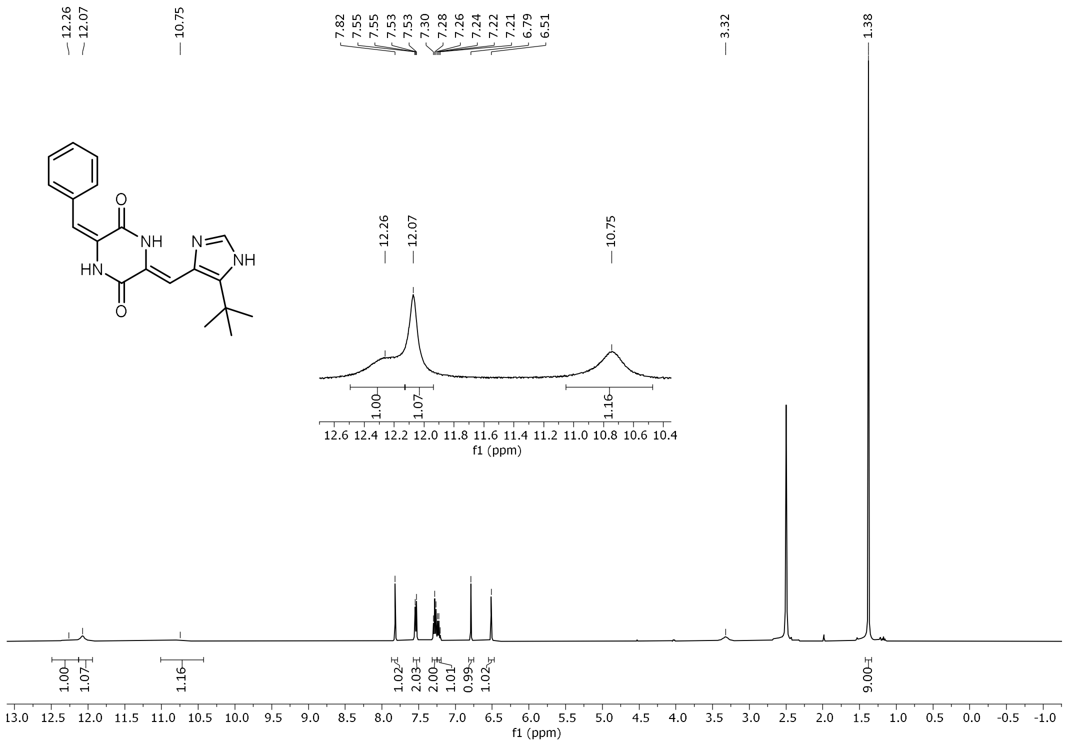

Supplementary Figure 103: ^{1}H NMR spectrum (400 MHz, DMSO-d_6) of compound E-**1**.

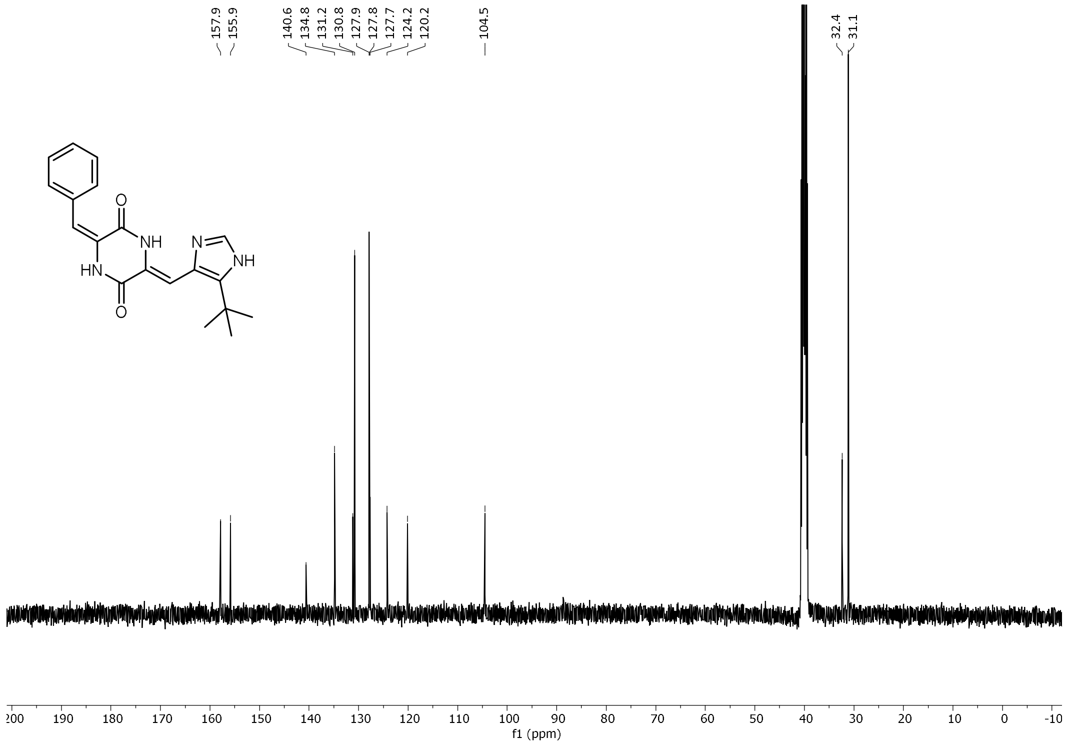

Supplementary Figure 104: ^{13}C NMR spectrum (101 MHz, DMSO-d_6) of compound E-**1**.

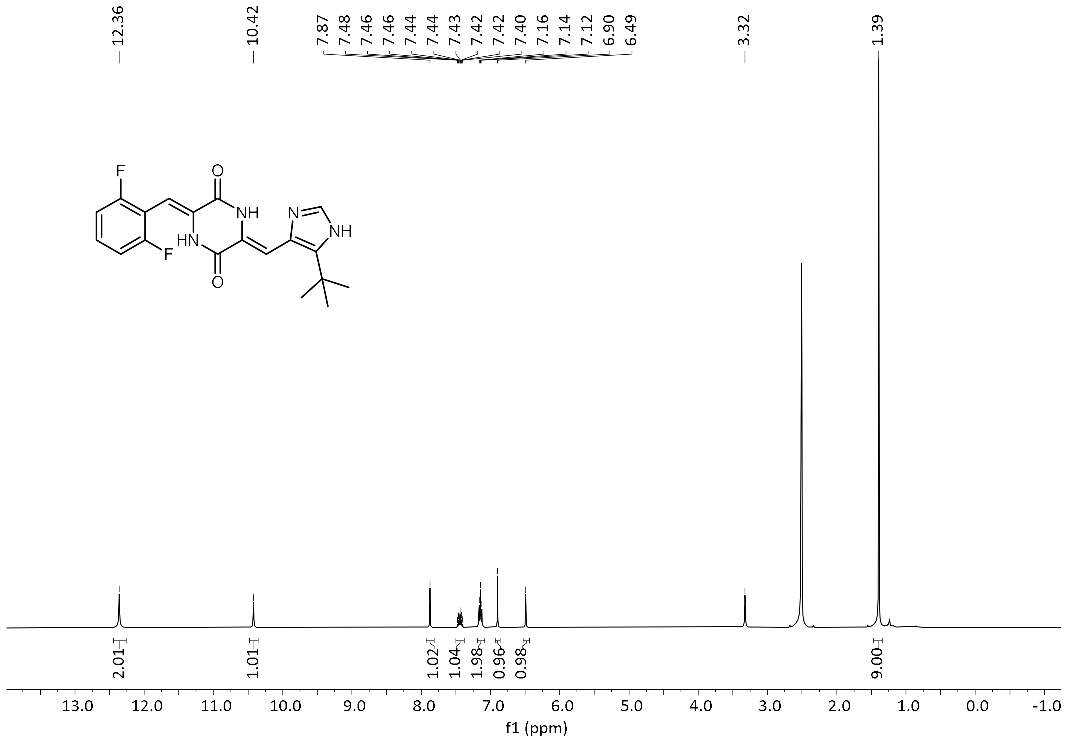

*Supplementary Figure 105: ^{1}H NMR spectrum (400 MHz, DMSO-d$_6$) of compound Z-**2**.*

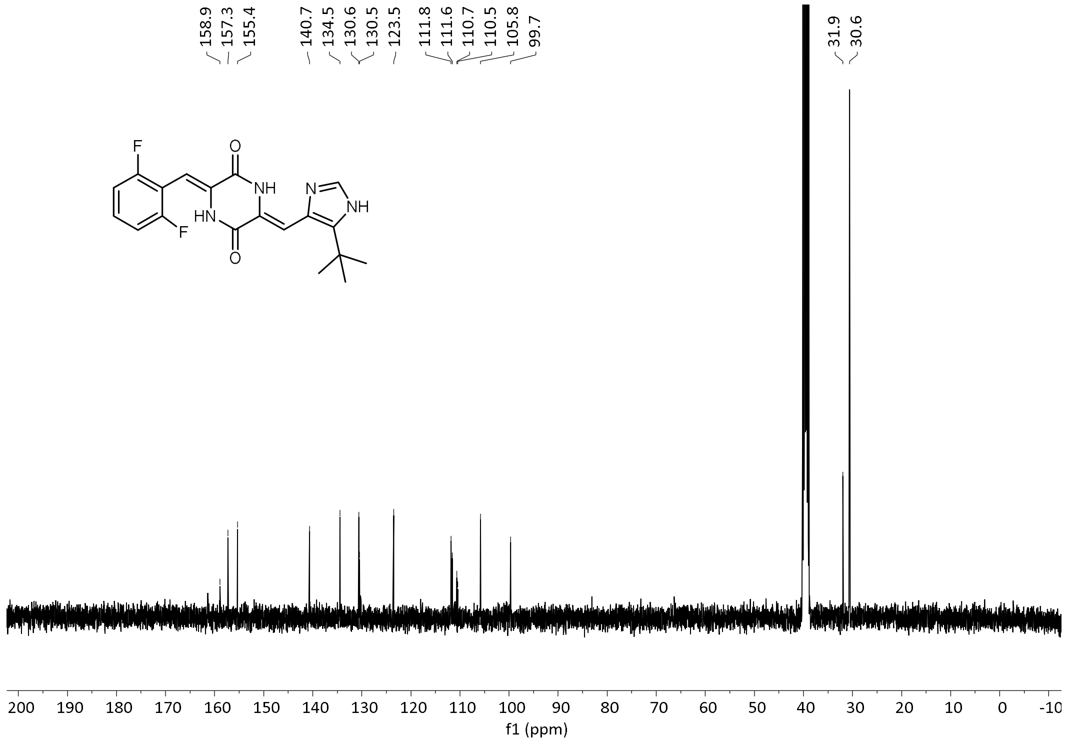

*Supplementary Figure 106: ^{13}C NMR spectrum (101 MHz, DMSO-d$_6$) of compound Z-**2**.*

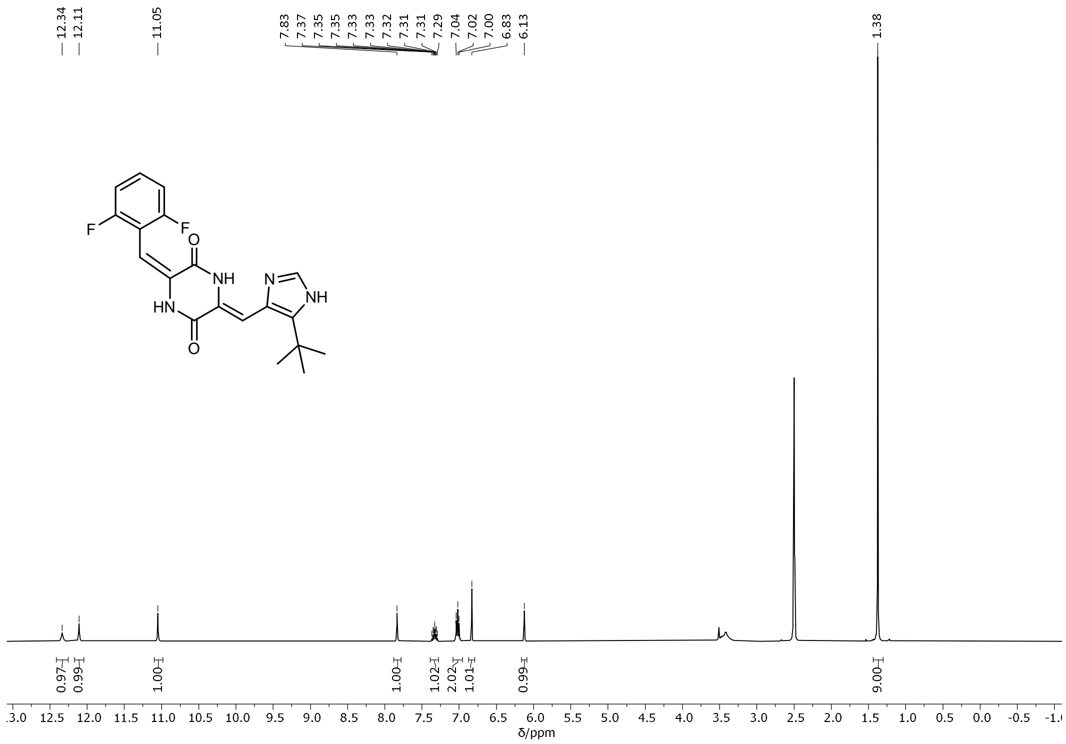

*Supplementary Figure 107: ¹H NMR spectrum (400 MHz, DMSO-d6) of compound E-**2**.*

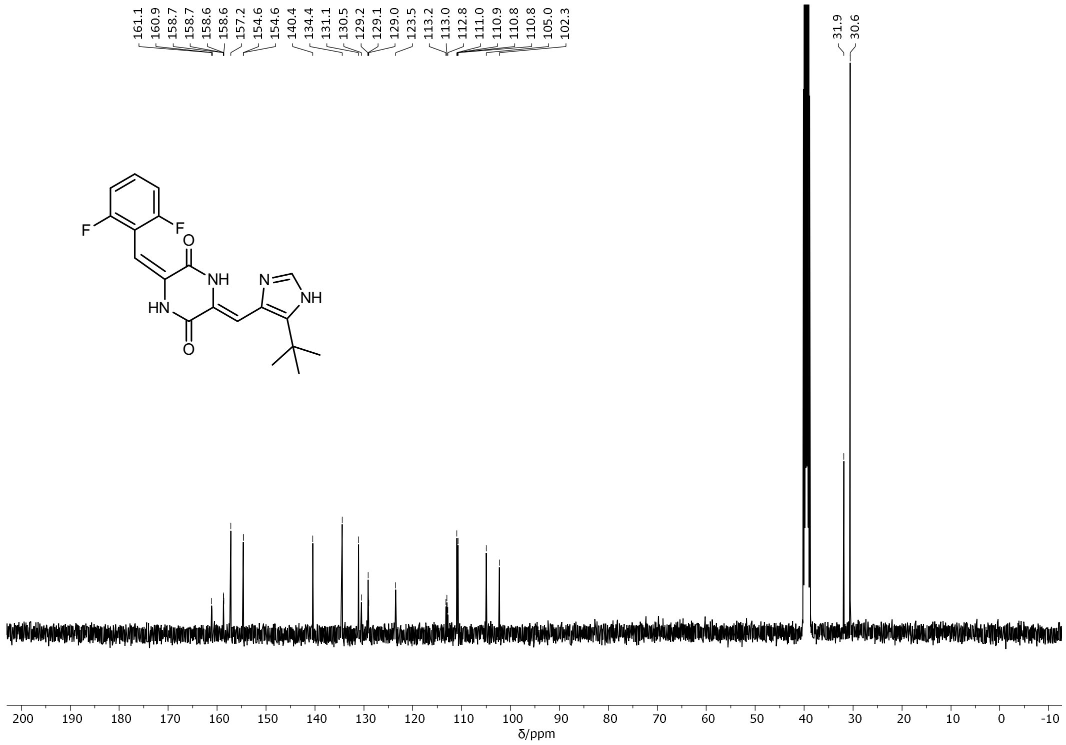

*Supplementary Figure 108: ¹³C NMR spectrum (101 MHz, DMSO-d6) of compound E-**2**.*

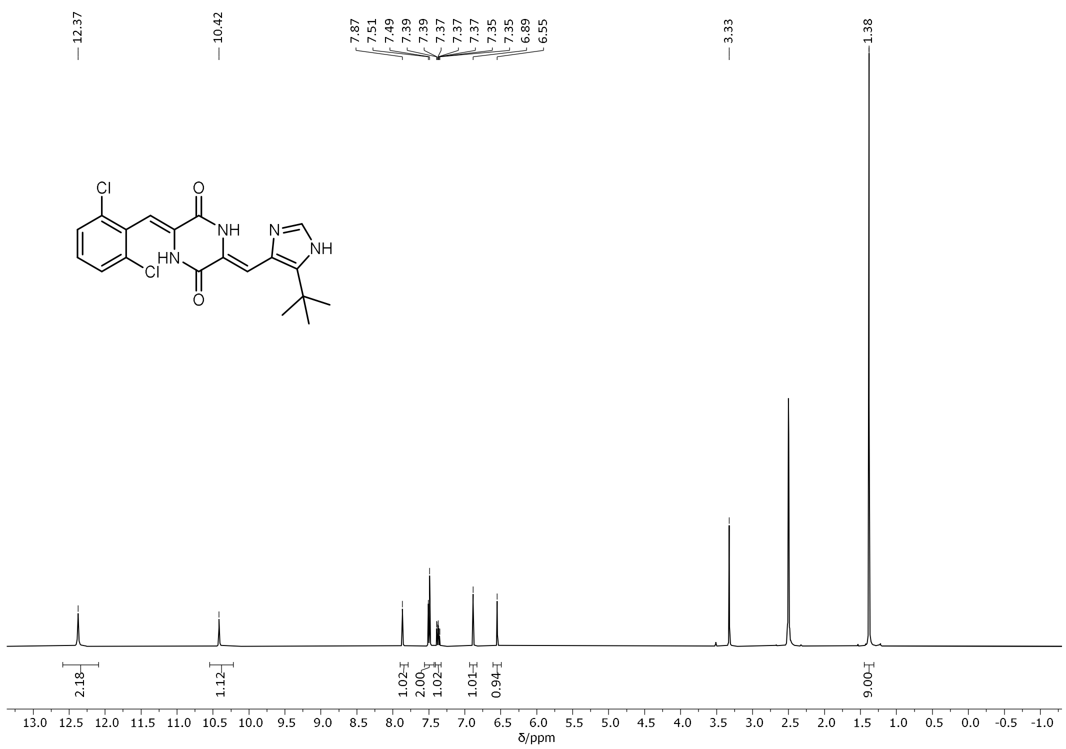

Supplementary Figure 109: ^{1}H NMR spectrum (400 MHz, DMSO-d_6) of compound Z-**3**.

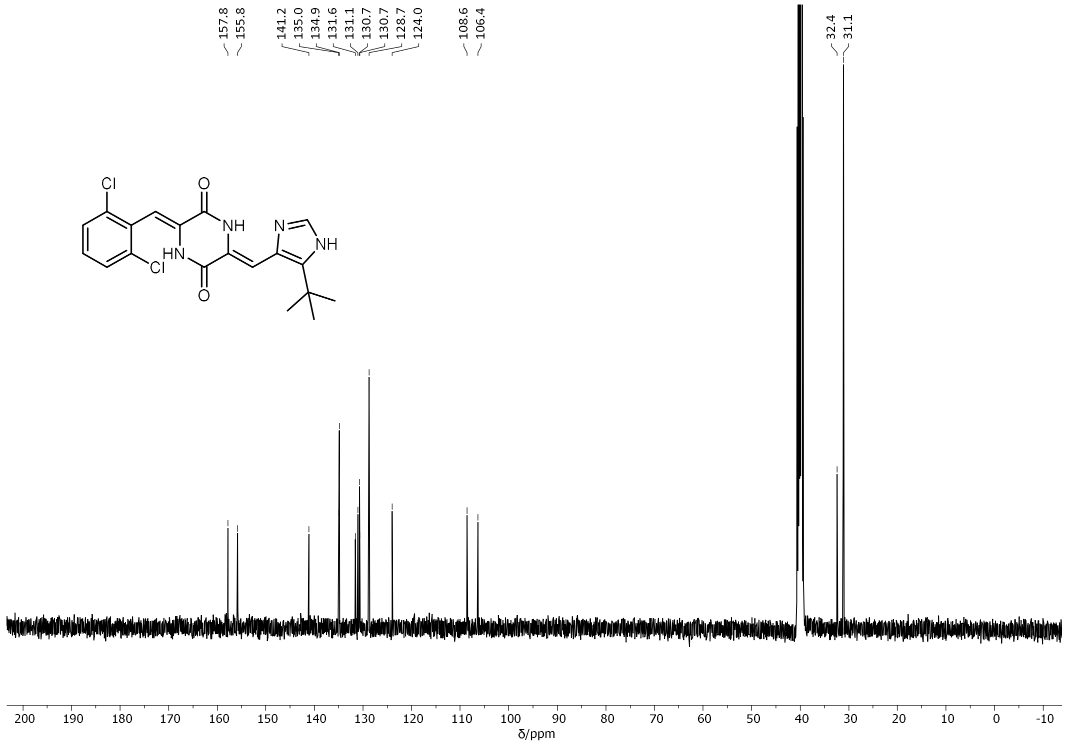

Supplementary Figure 110: ^{13}C NMR spectrum (101 MHz, DMSO-d_6) of compound Z-**3**.

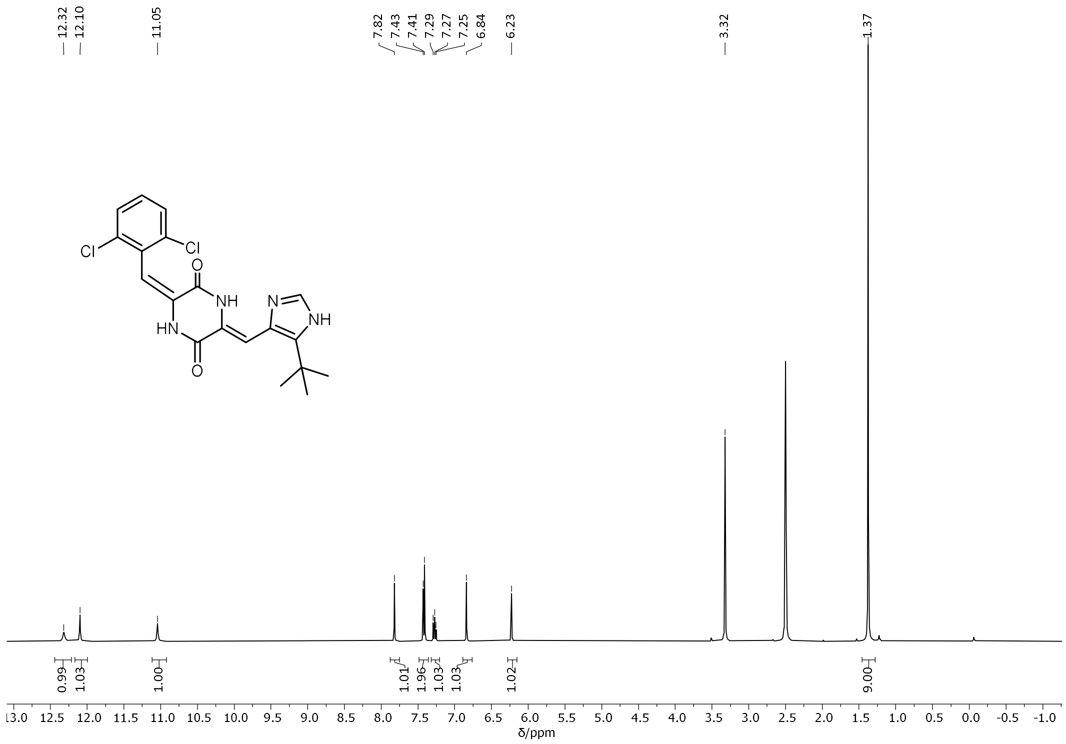

*Supplementary Figure 111: ¹H NMR spectrum (400 MHz, DMSO-d₆) of compound E-**3**.*

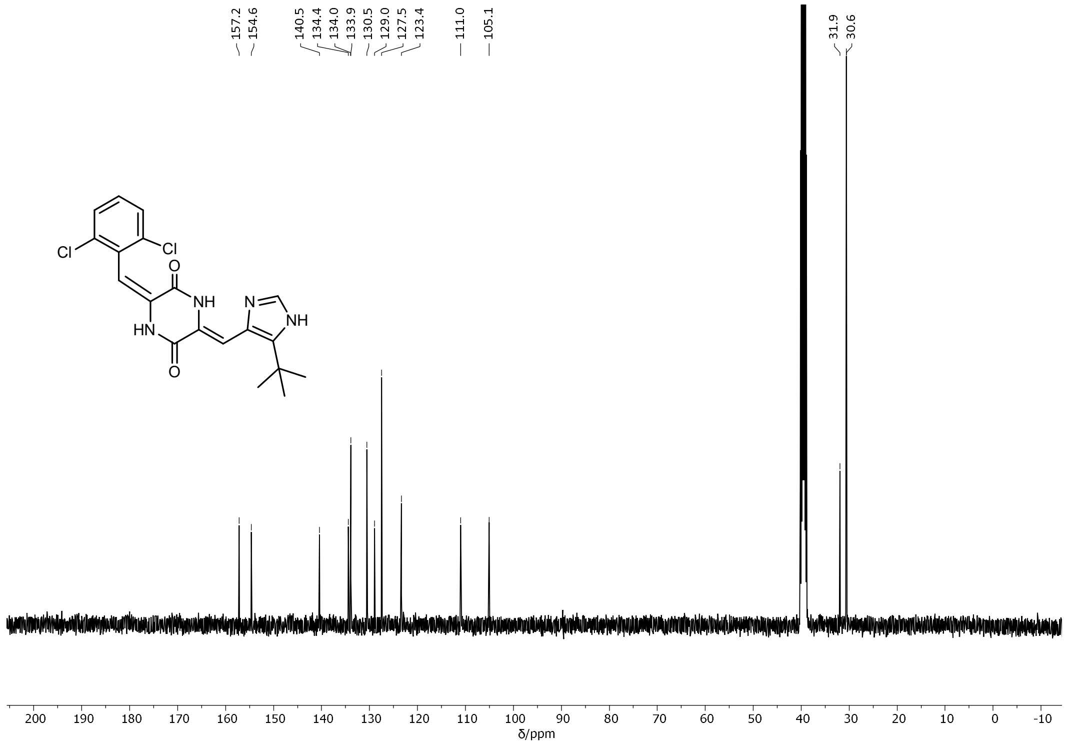

*Supplementary Figure 112: ¹³C NMR spectrum (101 MHz, DMSO-d₆) of compound E-**3**.*

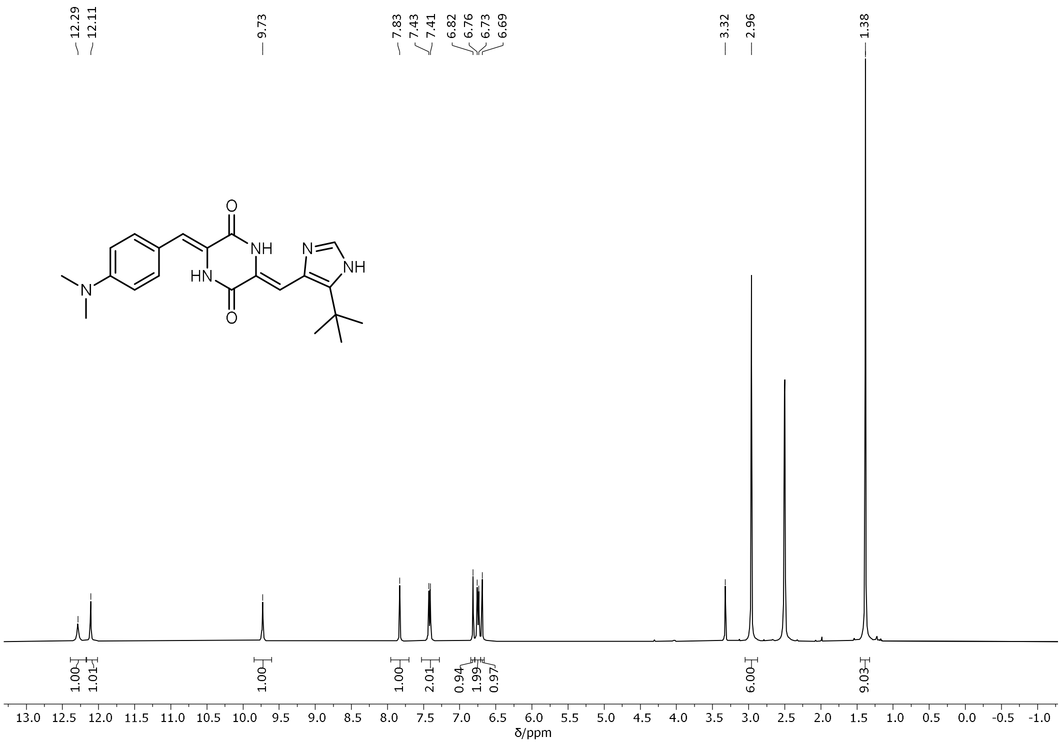

Supplementary Figure 113: ^{1}H NMR spectrum (400 MHz, DMSO-d_6) of compound Z-**4**.

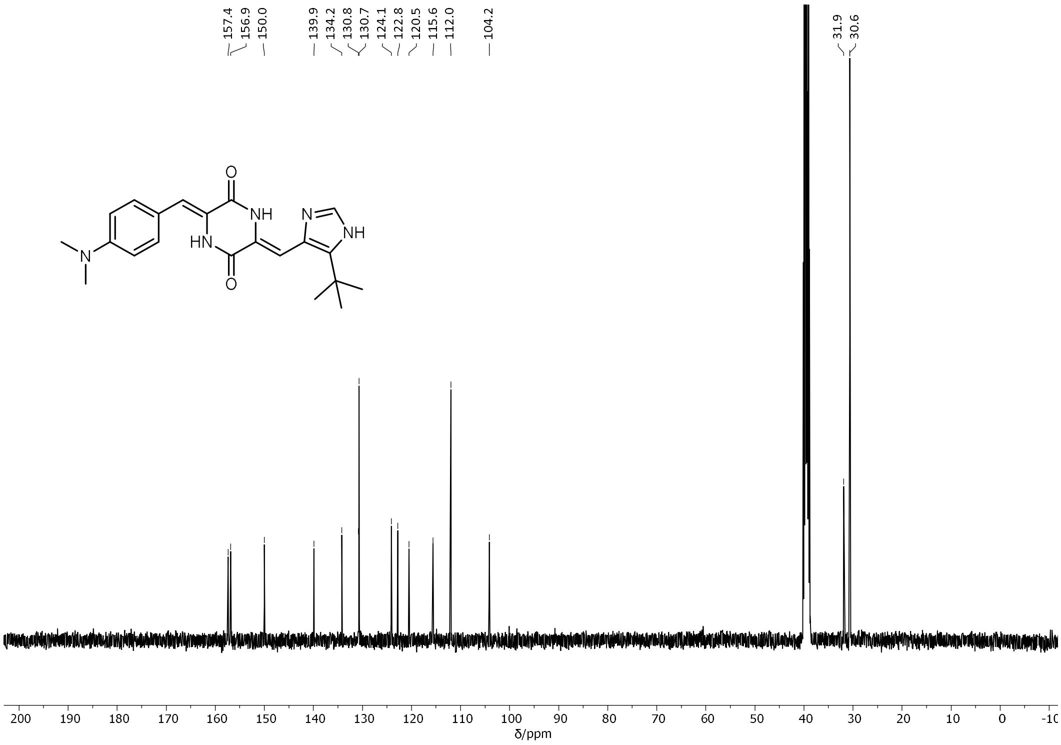

Supplementary Figure 114: ^{13}C NMR spectrum (101 MHz, DMSO-d_6) of compound Z-**4**.

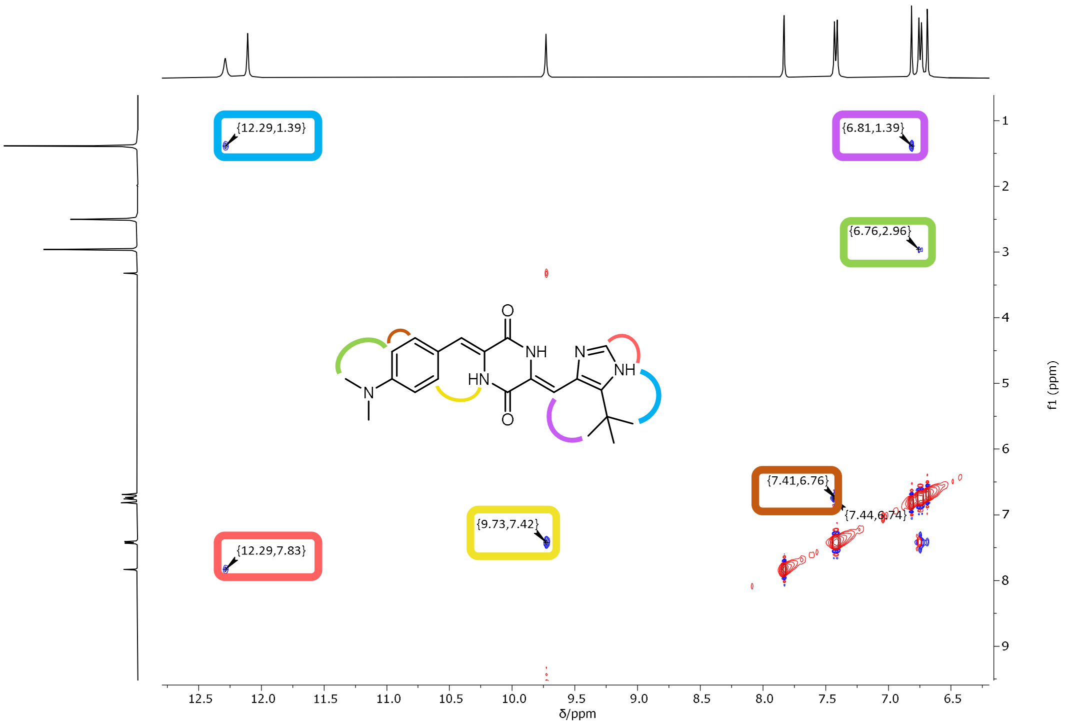

*Supplementary Figure 115: NOESY NMR spectrum (400 MHz, DMSO-d6) of compound Z-**4**.*

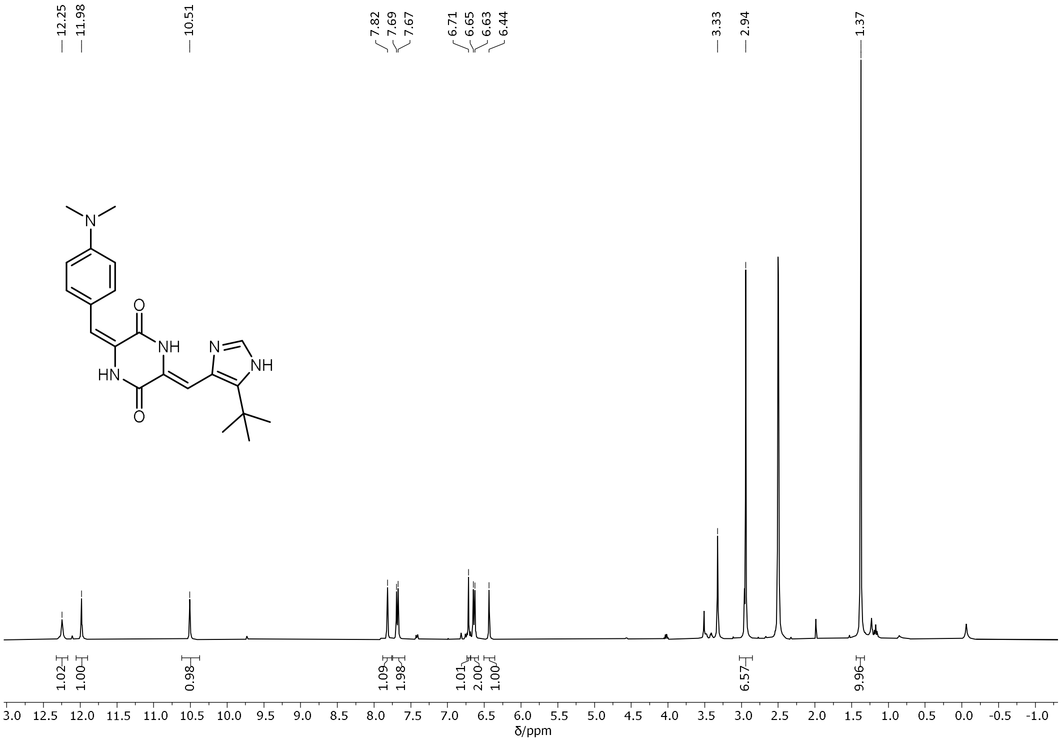

*Supplementary Figure 116: ^{1}H NMR spectrum (400 MHz, DMSO-d6) of compound E-**4**.*

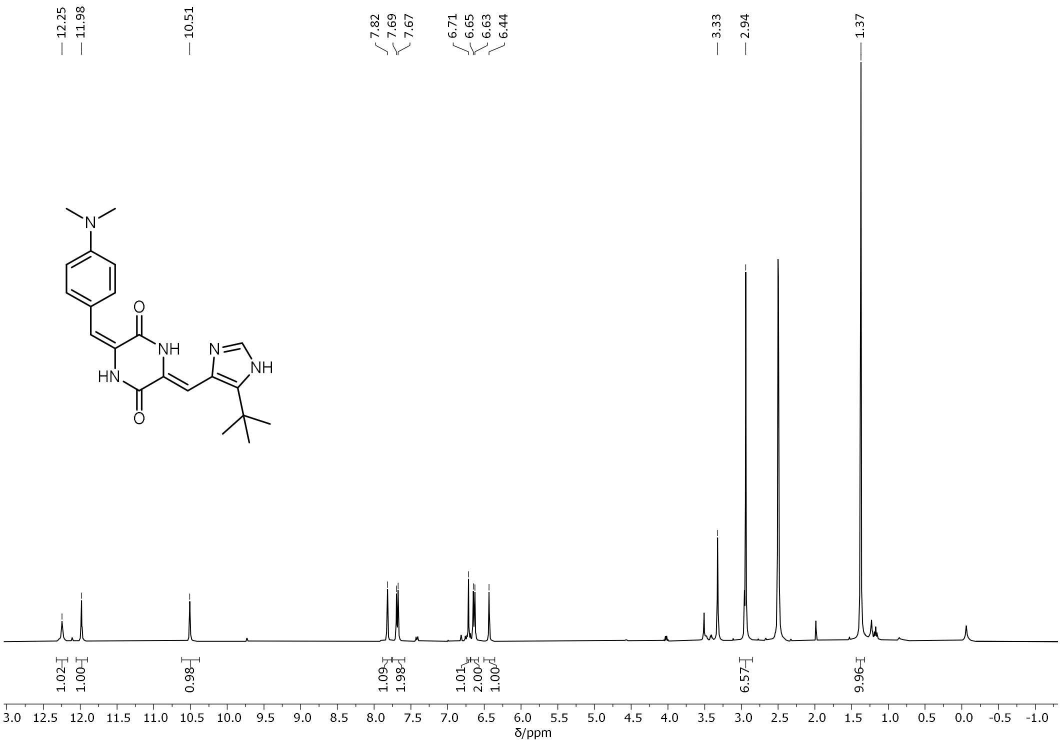

Supplementary Figure 117: ^{13}C NMR spectrum (101 MHz, DMSO-d_6) of compound E-**4**.

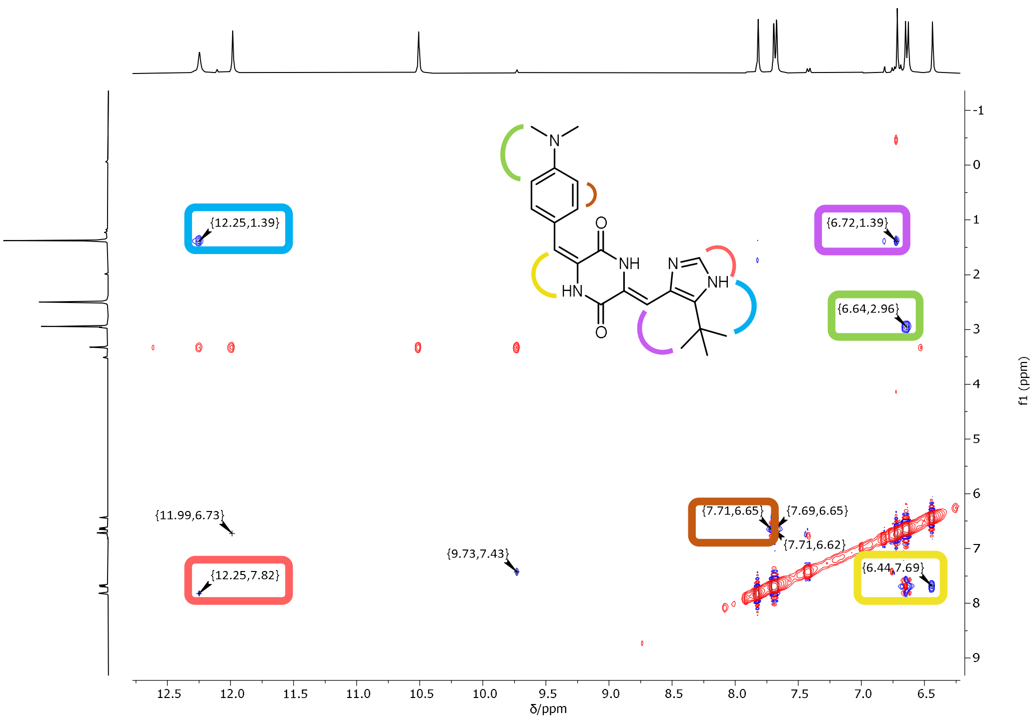

Supplementary Figure 118: NOESY NMR spectrum (400 MHz, DMSO-d_6) of compound E-**4**.

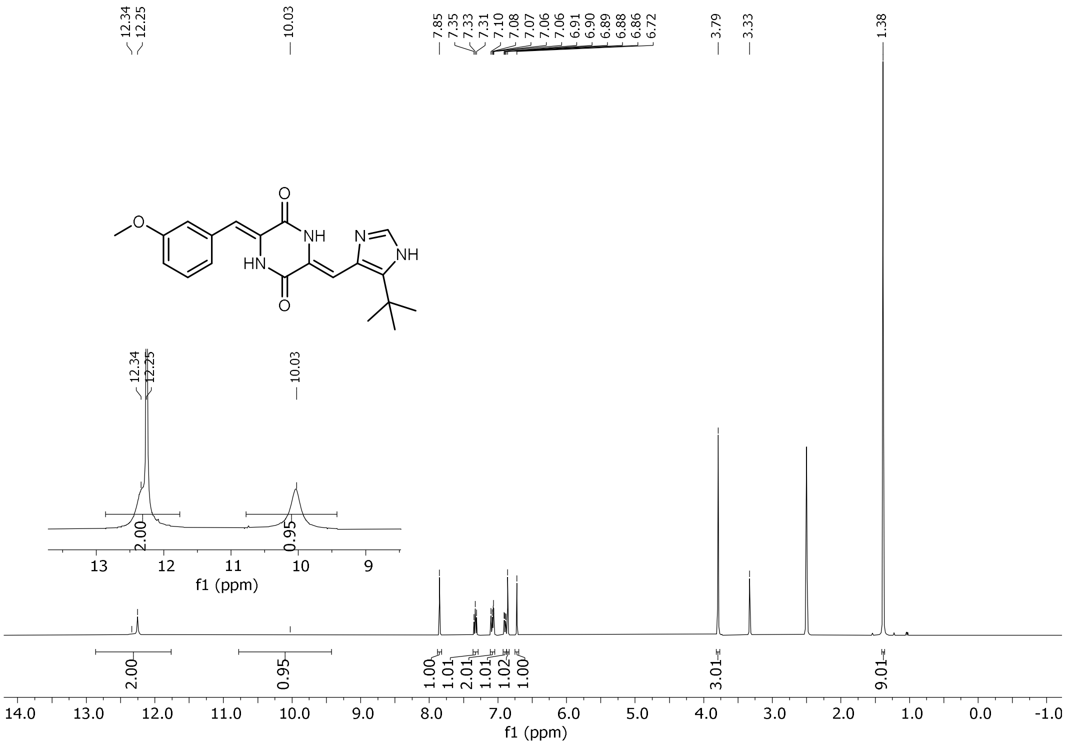

Supplementary Figure 119: ^{1}H NMR spectrum (400 MHz, DMSO-d_6) of compound Z-**5**.

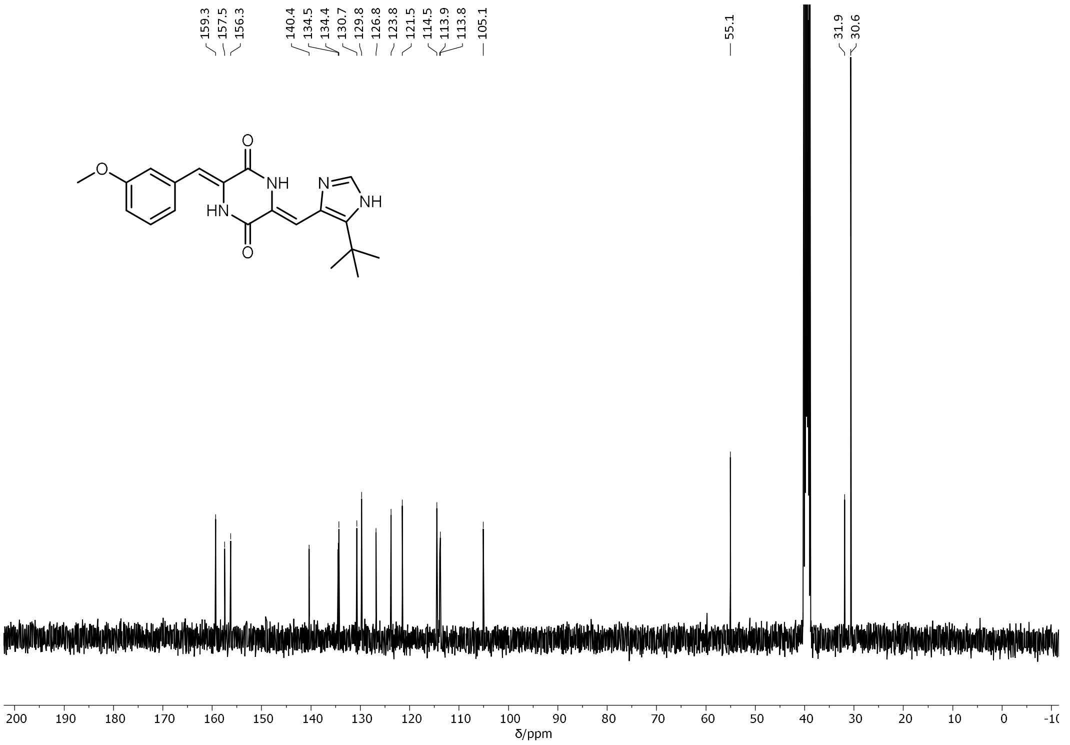

Supplementary Figure 120: ^{13}C NMR spectrum (101 MHz, DMSO-d_6) of compound Z-**5**.

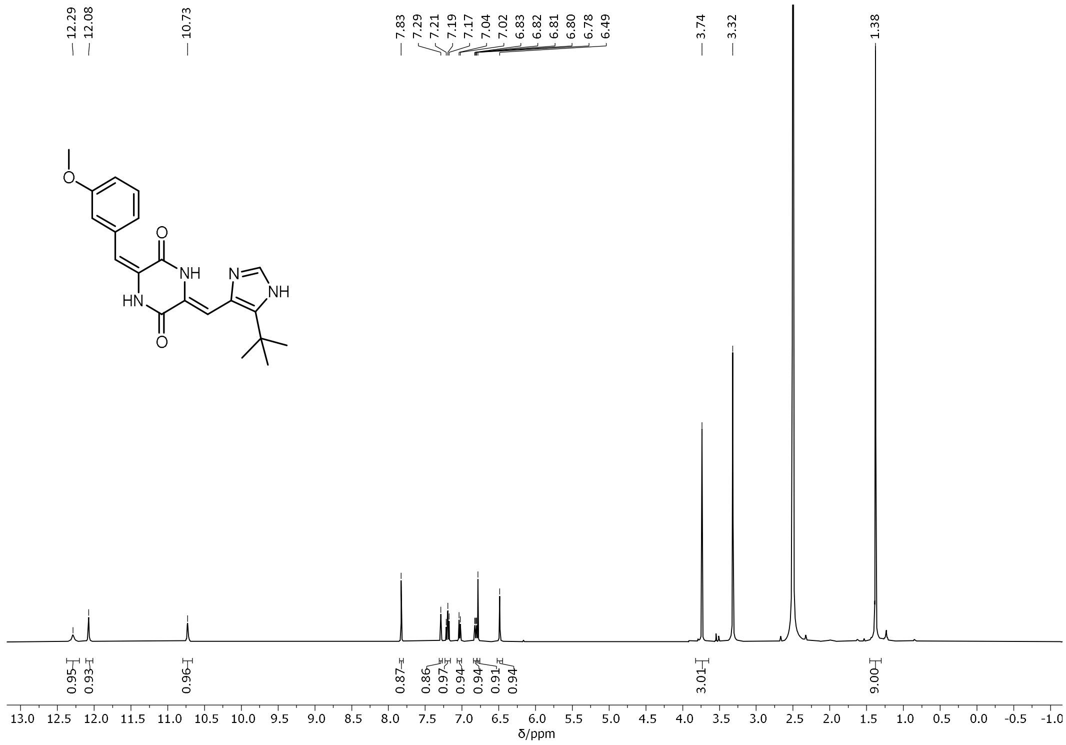

*Supplementary Figure 121: ^{1}H NMR spectrum (400 MHz, DMSO-d_6) of compound E-**5**.*

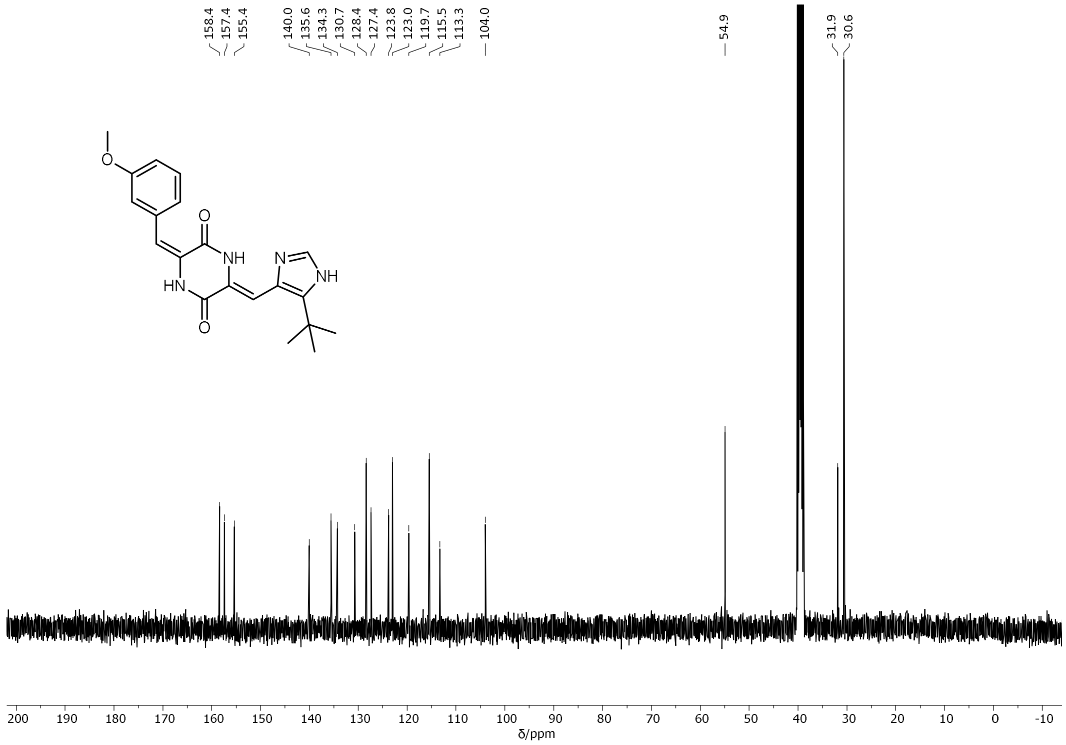

*Supplementary Figure 122: ^{13}C NMR spectrum (101 MHz, DMSO-d_6) of compound E-**5**.*

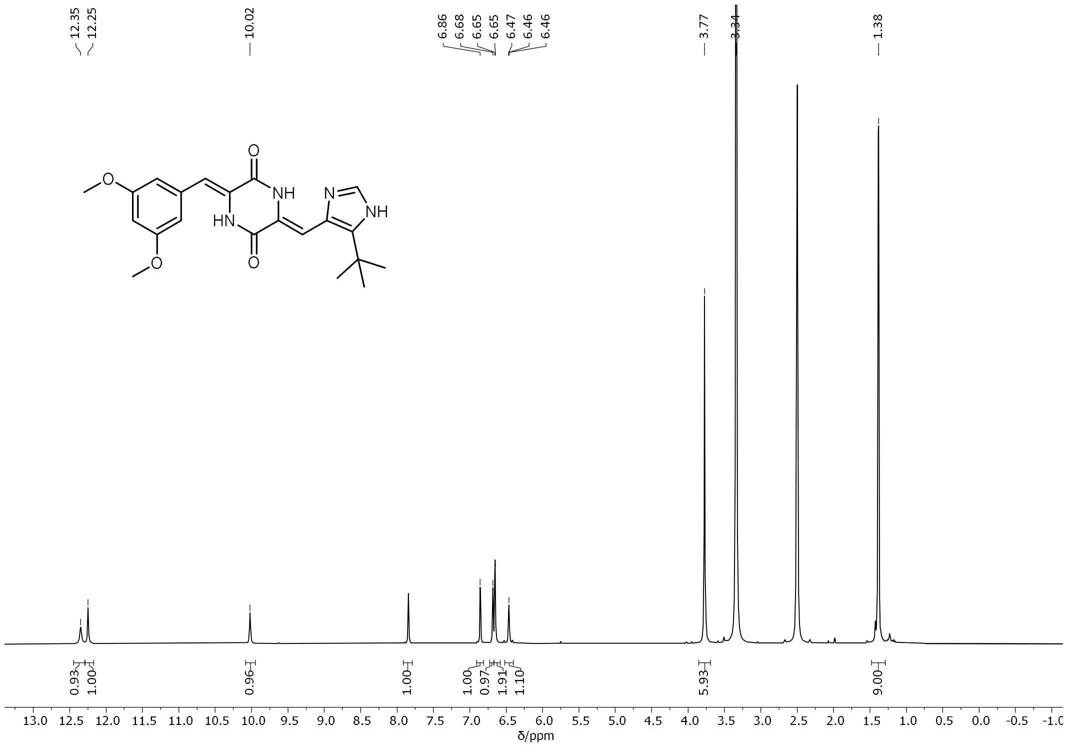

*Supplementary Figure 123: ^{1}H NMR spectrum (400 MHz, DMSO-d_6) of compound Z-**6**.*

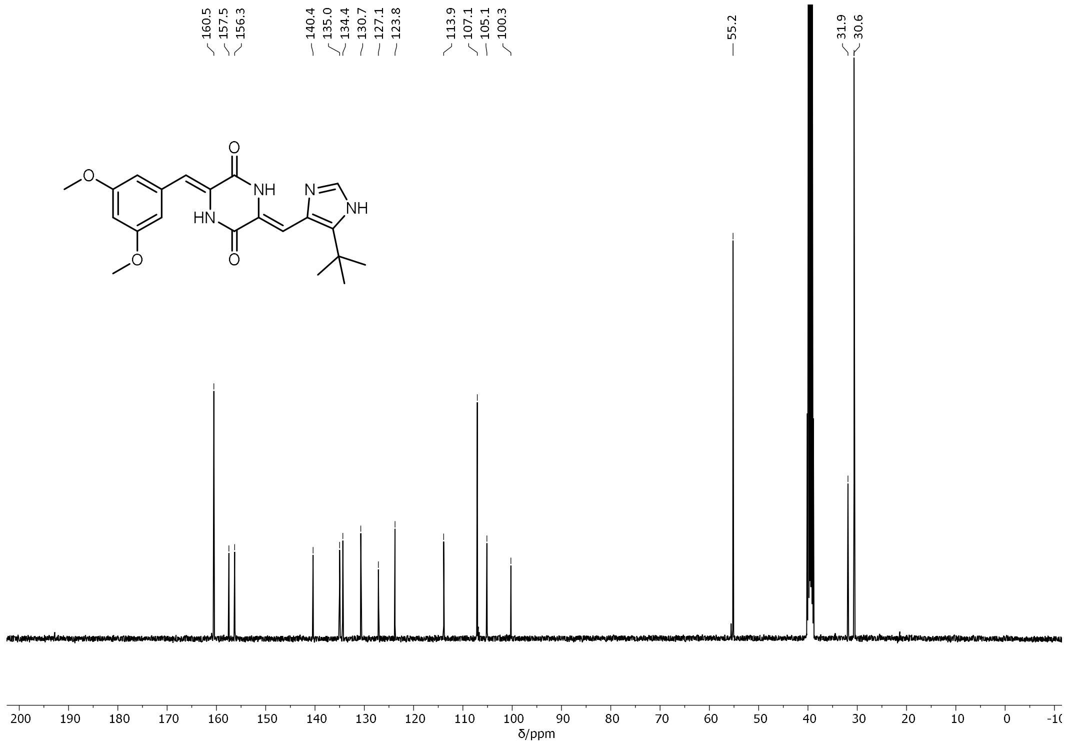

*Supplementary Figure 124: ^{13}C NMR spectrum (101 MHz, DMSO-d_6) of compound Z-**6**.*

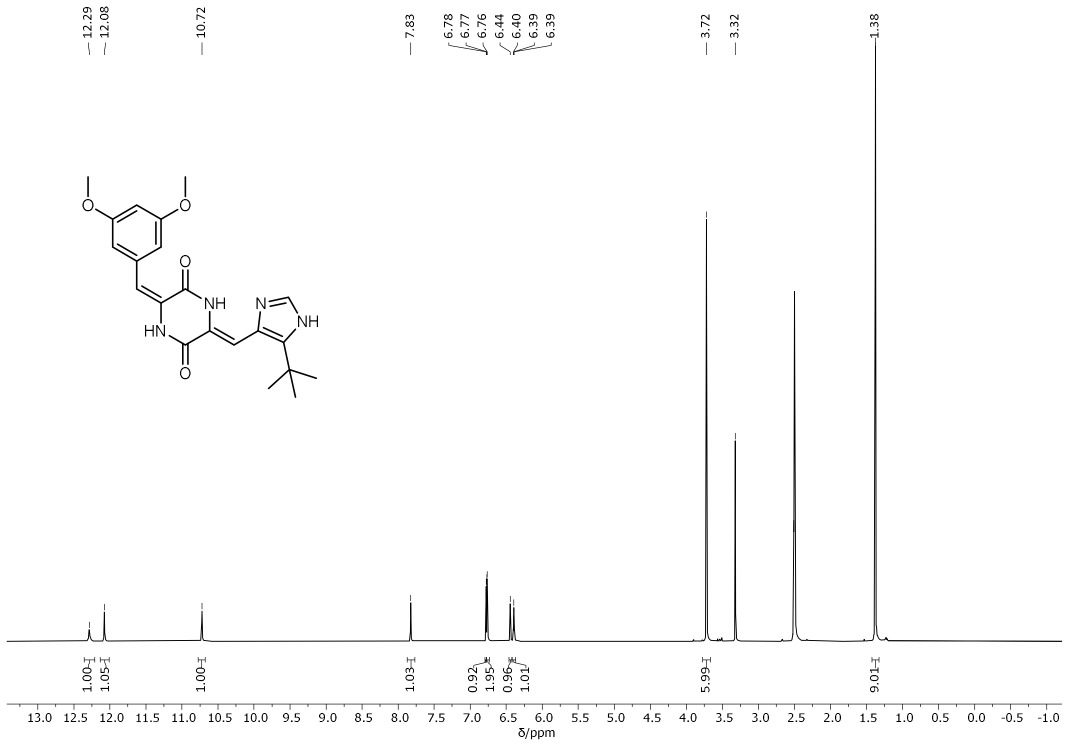

*Supplementary Figure 125: ^{1}H NMR spectrum (400 MHz, DMSO-d_6) of compound E-**6**.*

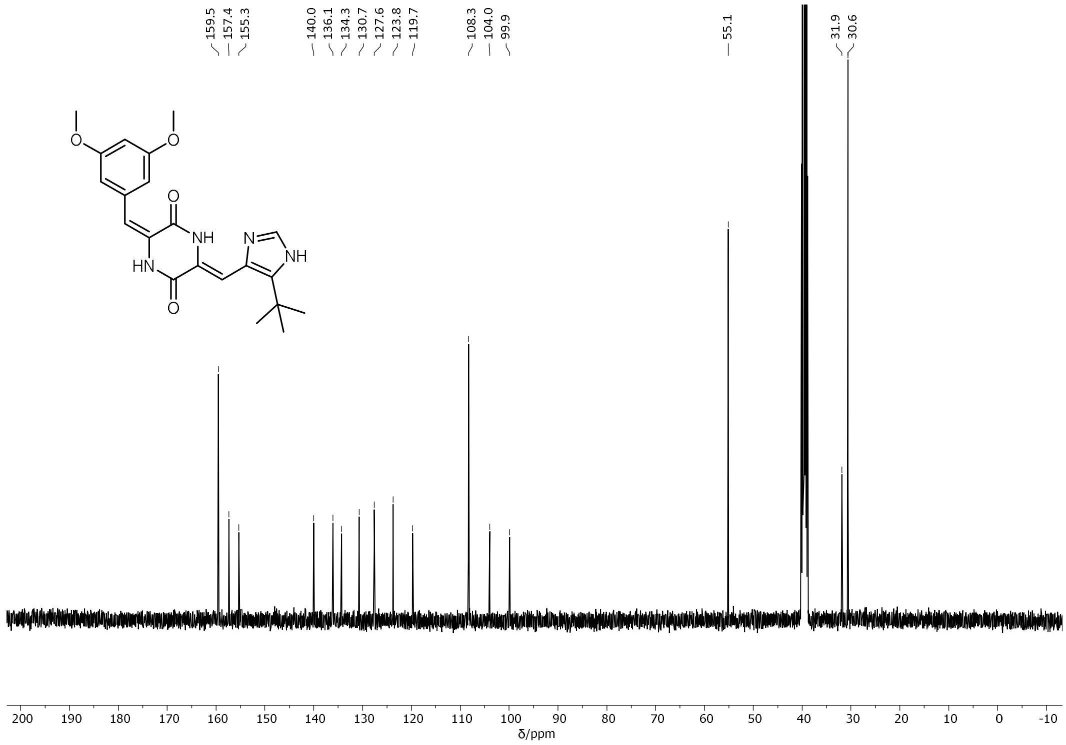

*Supplementary Figure 126: ^{13}C NMR spectrum (101 MHz, DMSO-d_6) of compound E-**6**.*

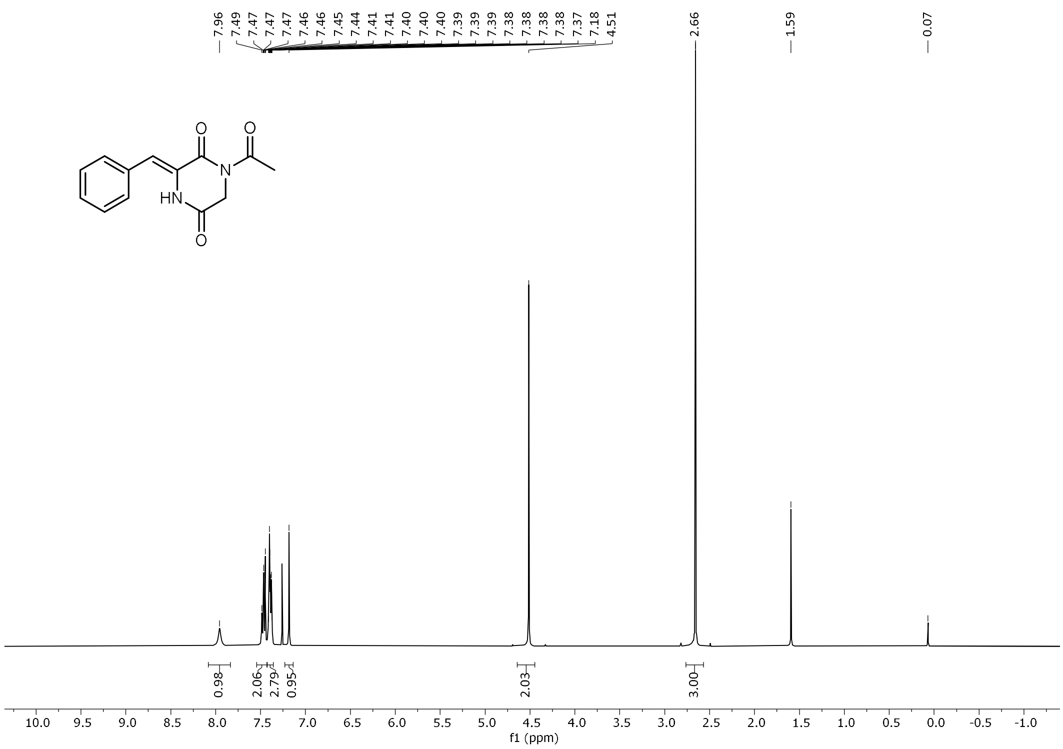

*Supplementary Figure 127: ^{1}H NMR spectrum (400 MHz, $CDCl_3$) of compound Z-**7**.*silicon grease.*

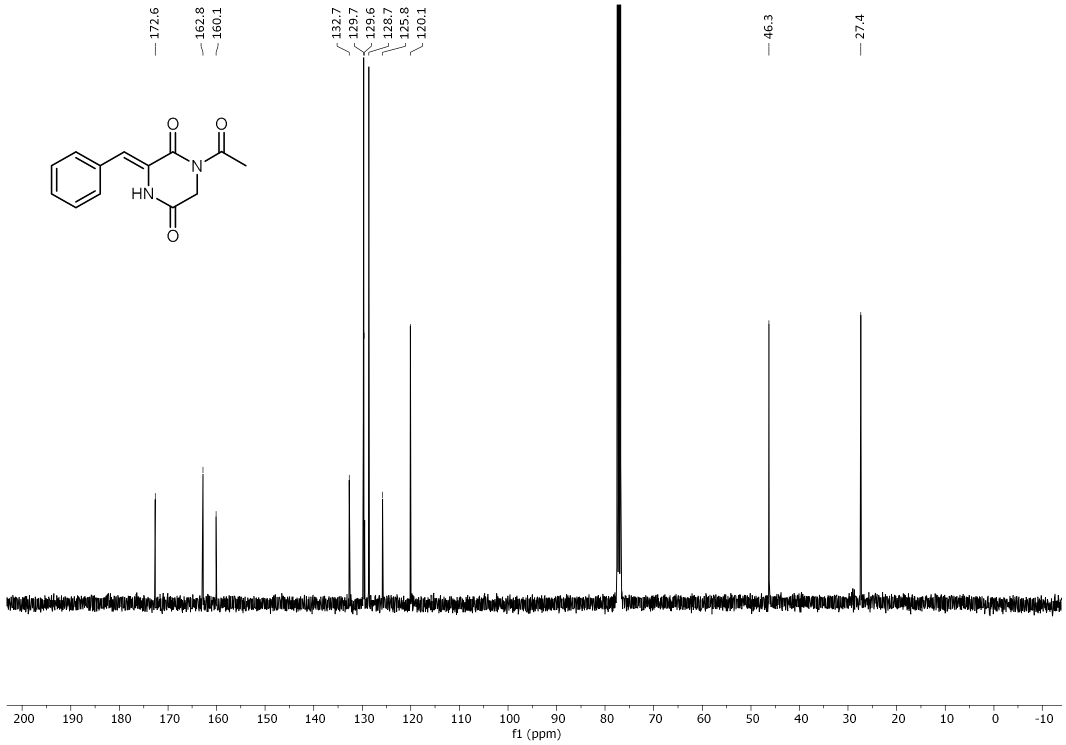

*Supplementary Figure 128: ^{13}C NMR spectrum (101 MHz, $CDCl_3$) of compound Z-**7**.*

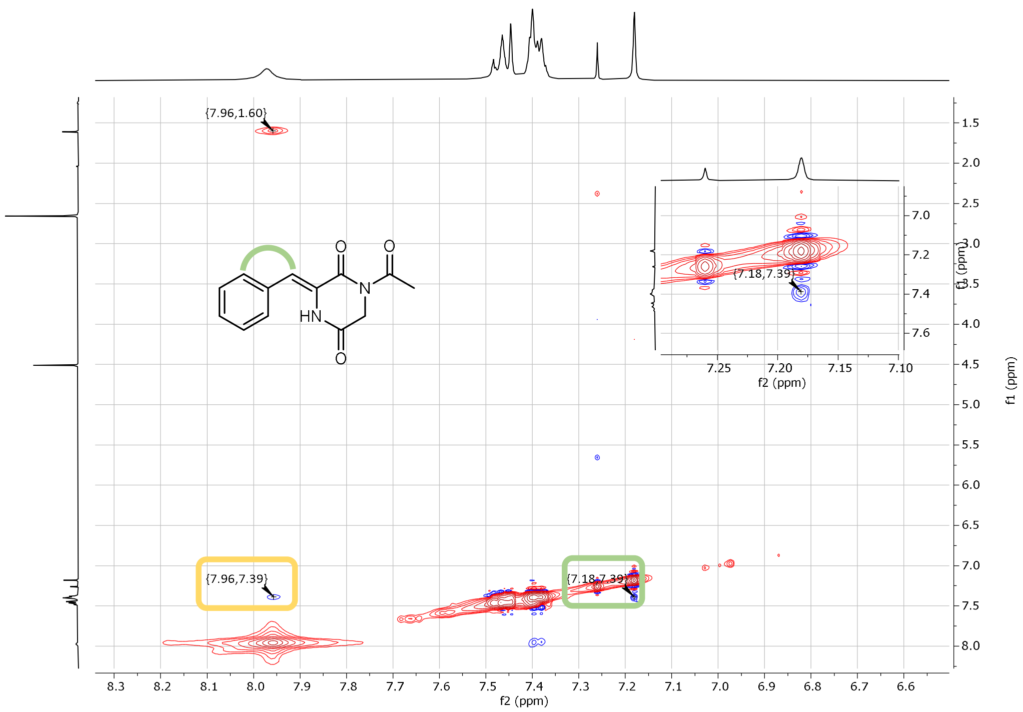

Supplementary Figure 129: NOESY NMR spectrum (400 MHz, $CDCl_3$) of compound Z-**7**.

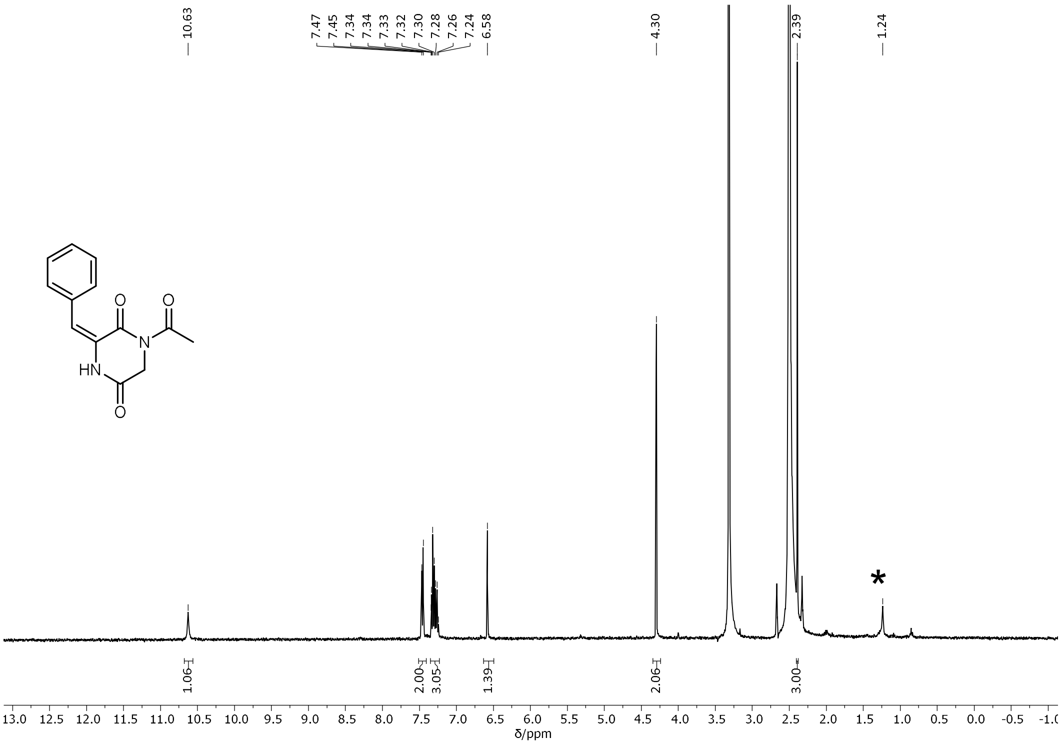

Supplementary Figure 130: ^1H NMR spectrum (400 MHz, DMSO-d_6) of compound E-**7**. *undefined impurity

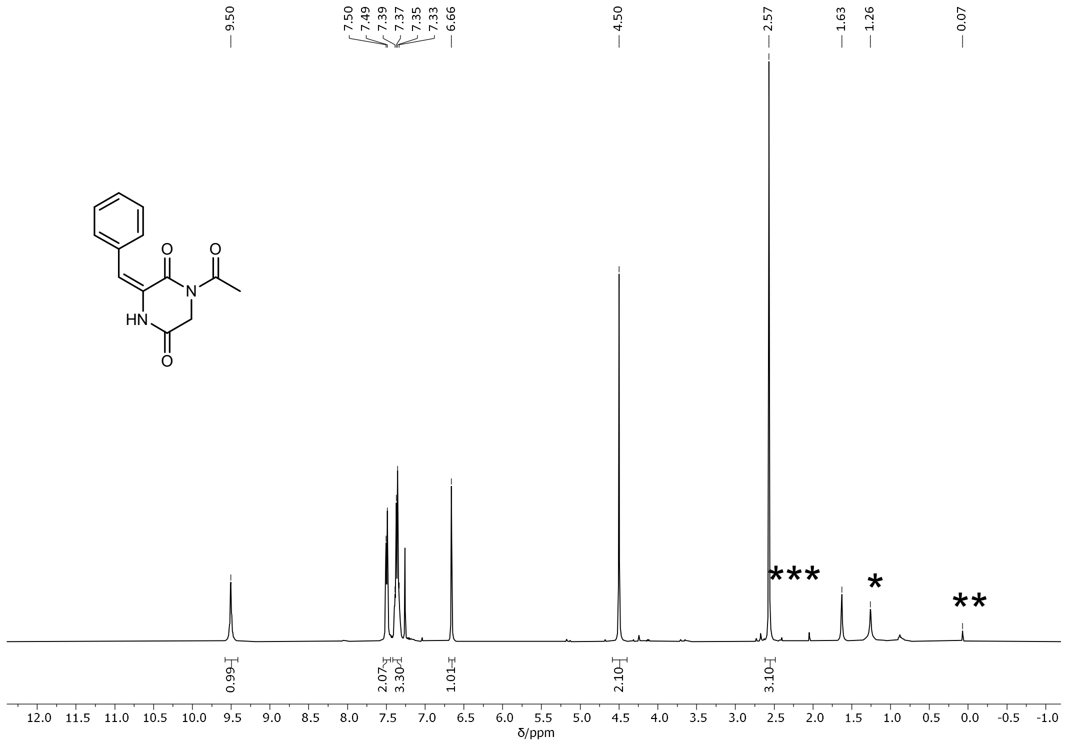

*Supplementary Figure 131: ^{1}H NMR spectrum (400 MHz, $CDCl_3$) of compound E-**7**. *undefined impurity, **silicon grease, ***ethyl acetate.*

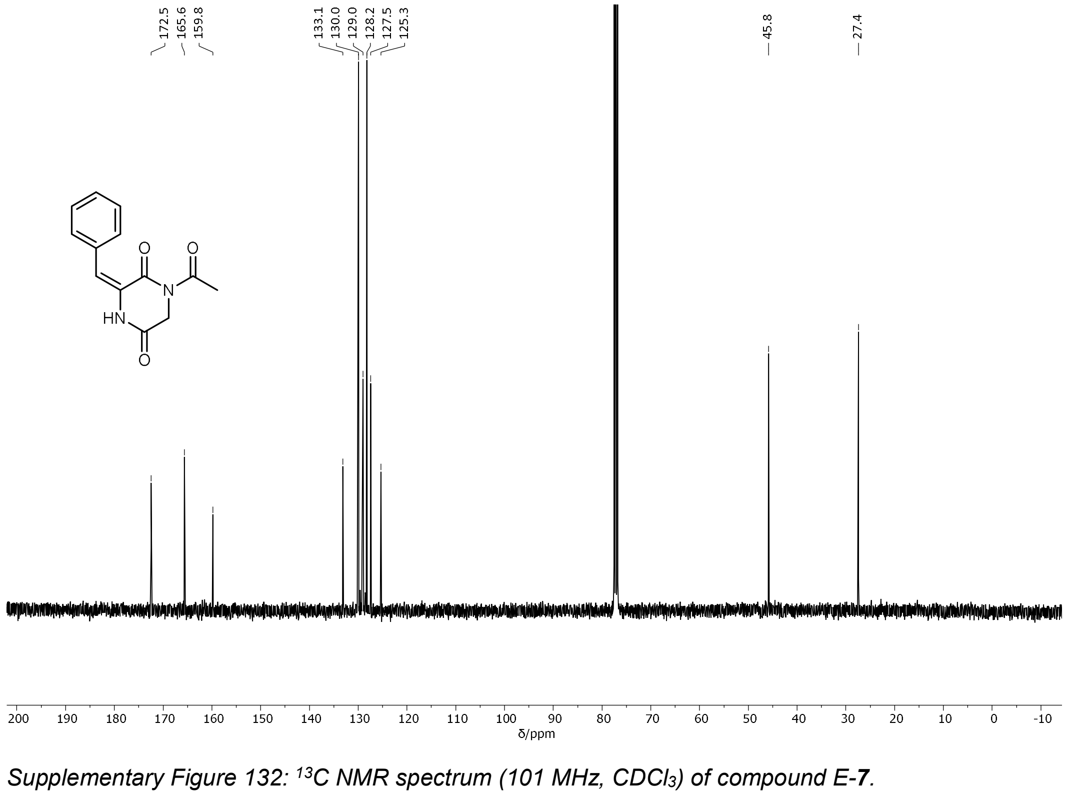

*Supplementary Figure 132: ^{13}C NMR spectrum (101 MHz, $CDCl_3$) of compound E-**7**.*

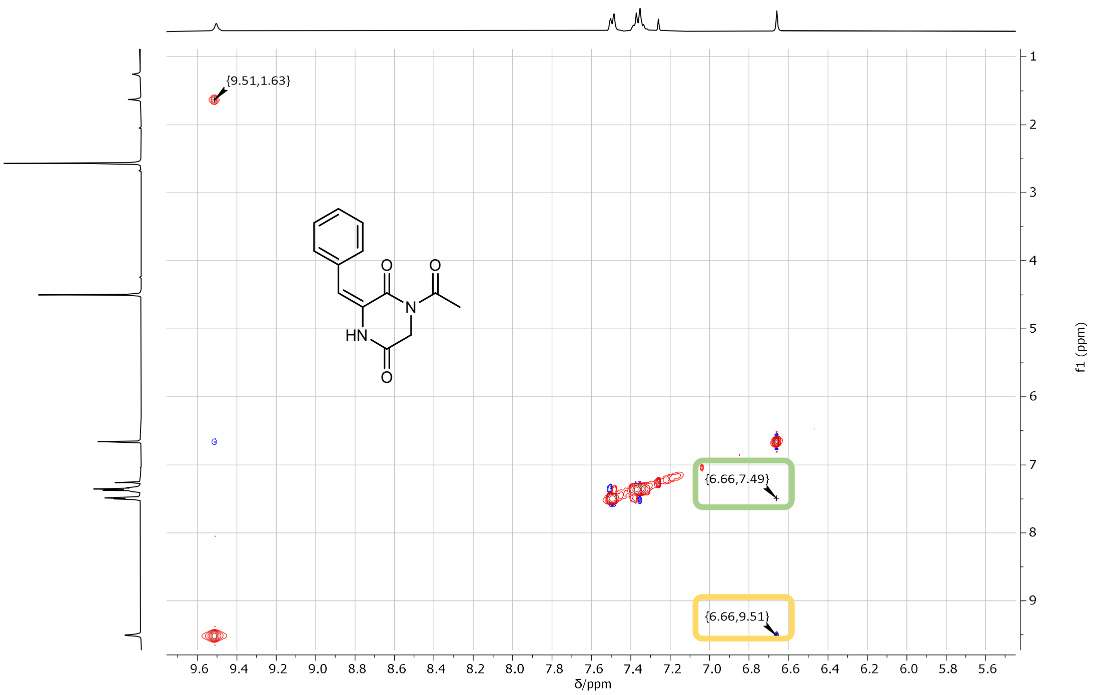

*Supplementary Figure 133: NOESY NMR spectrum (400 MHz, $CDCl_3$) of compound E-**7**.*

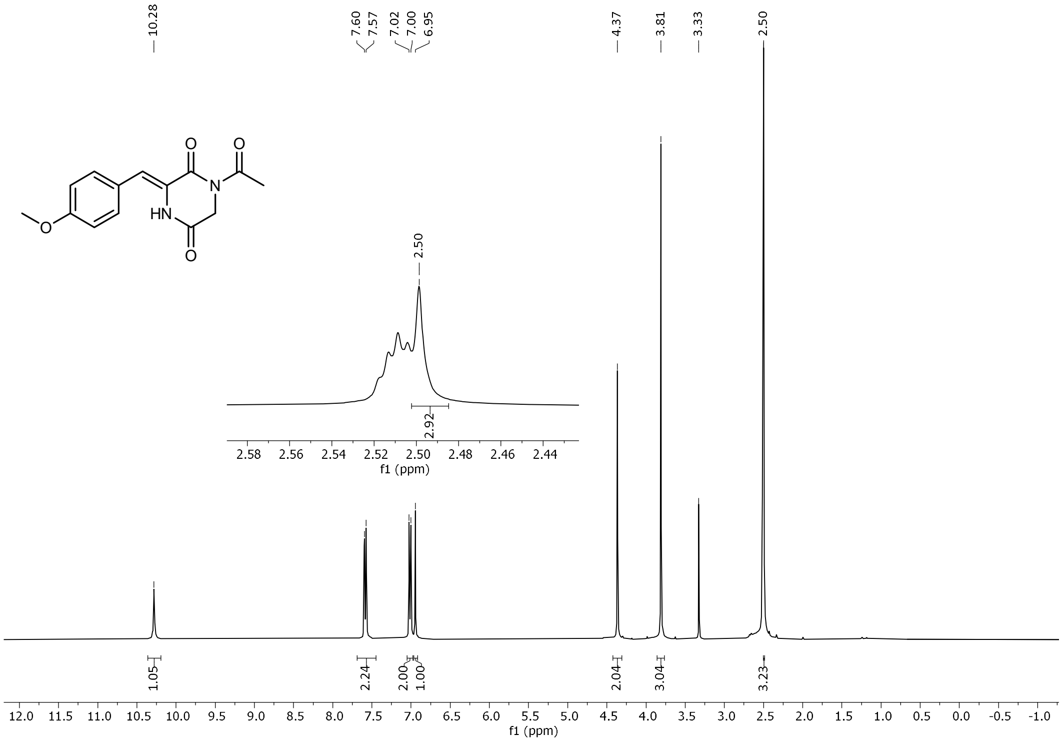

*Supplementary Figure 134: ^{1}H NMR spectrum (400 MHz, DMSO-d_6) of compound Z-**8**.*

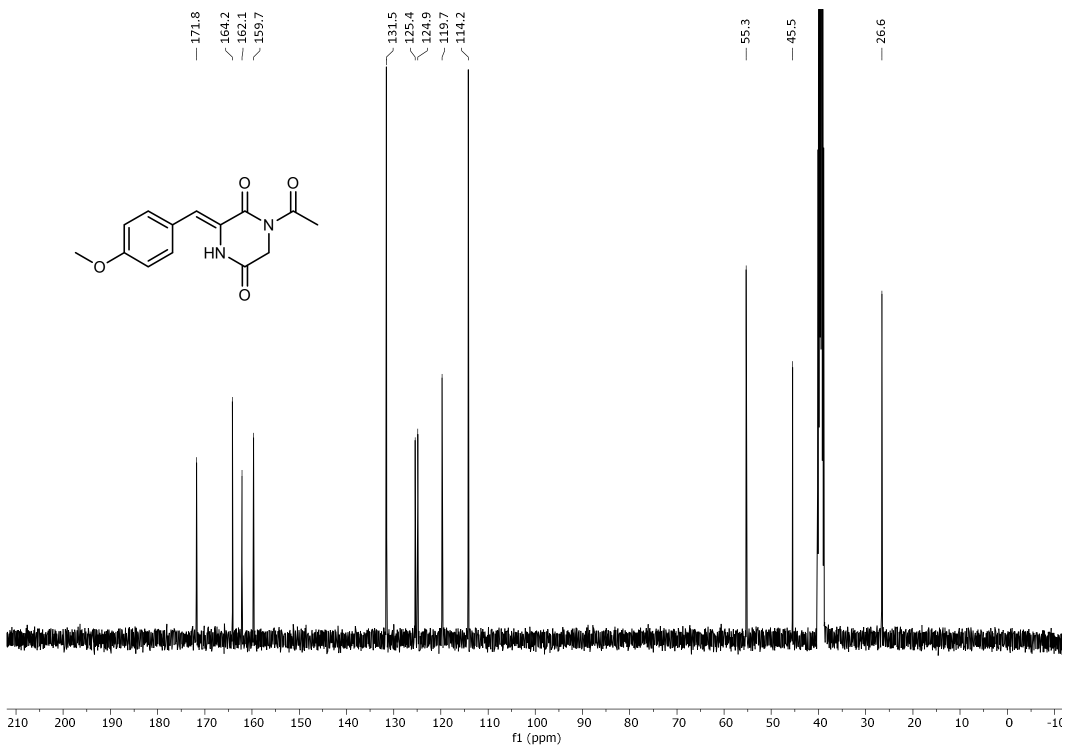

*Supplementary Figure 135: ^{13}C NMR spectrum (101 MHz, DMSO-d_6) of compound Z-**8**.*

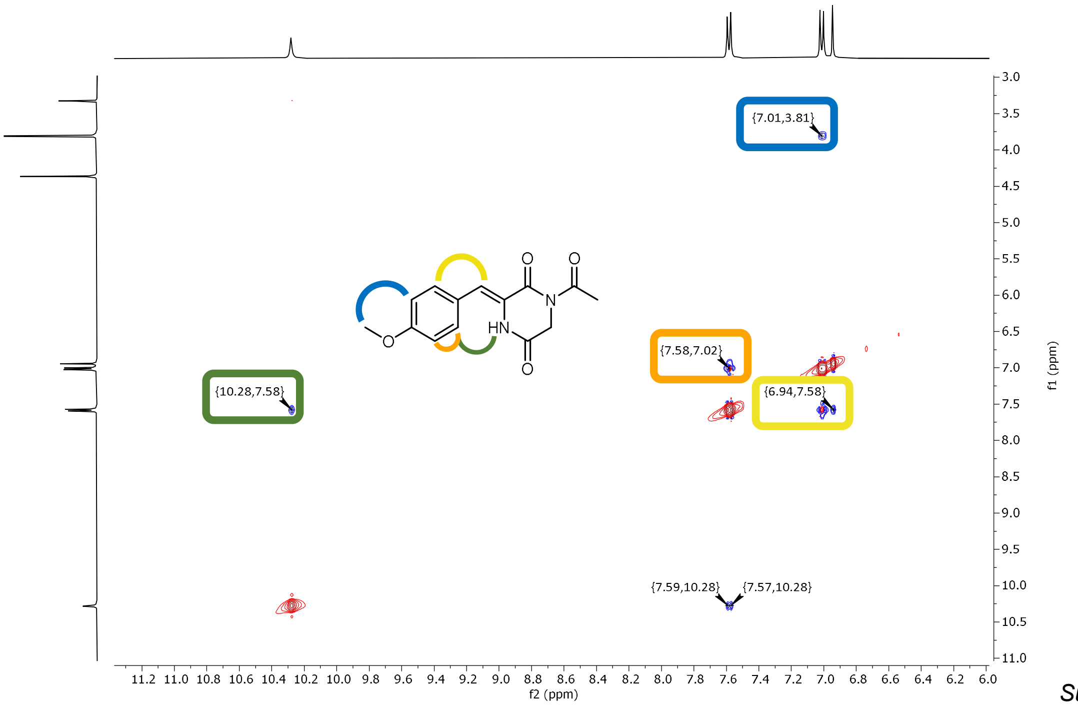

Supplementary Figure 136: NOESY NMR spectrum (400 MHz, DMSO-d6) of compound Z-**8**.

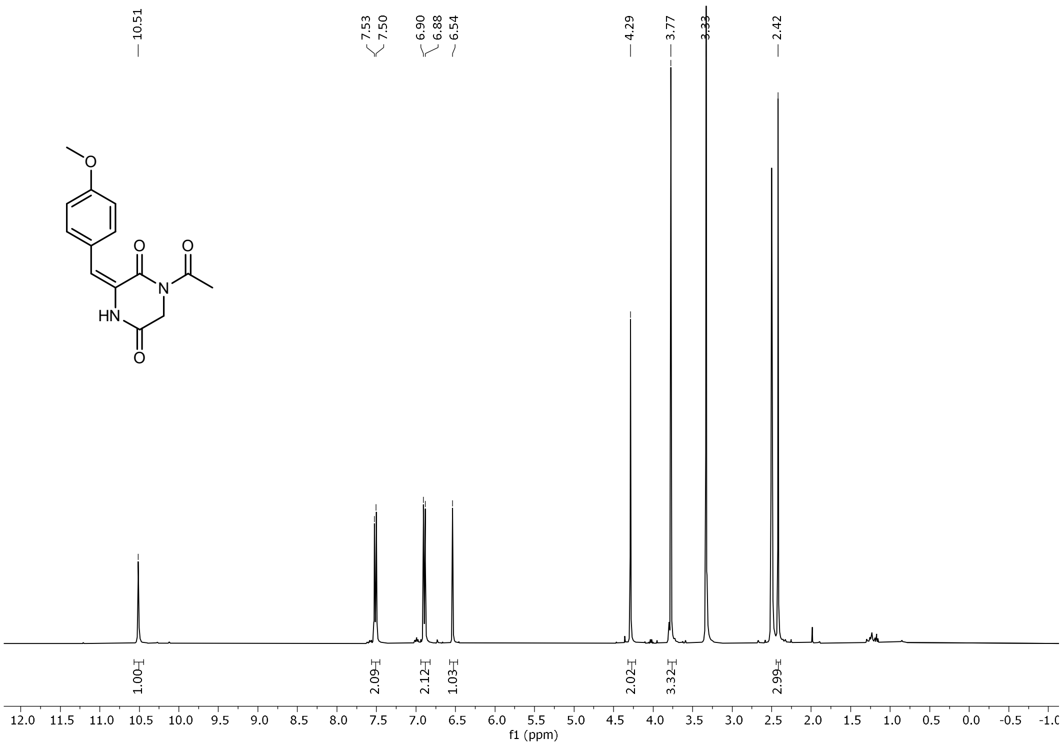

Supplementary Figure 137: ¹H NMR spectrum (400 MHz, DMSO-d6) of compound E-**8**.

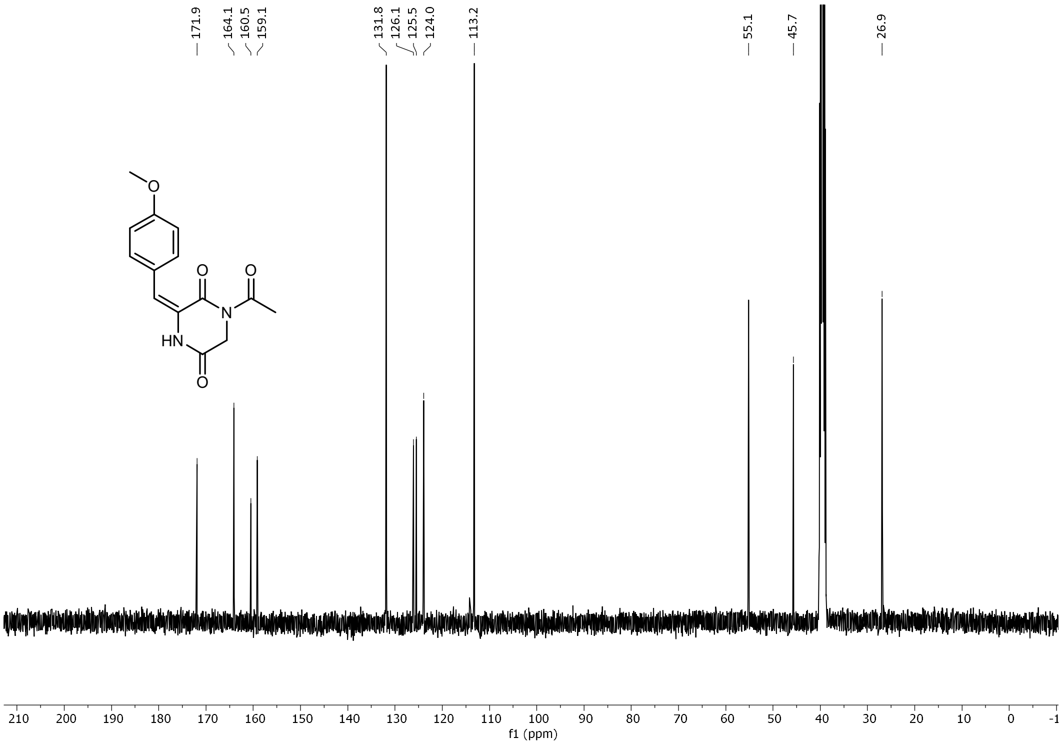

Supplementary Figure 138: ^{13}C NMR spectrum (101 MHz, DMSO-d_6) of compound *E*-**8**.

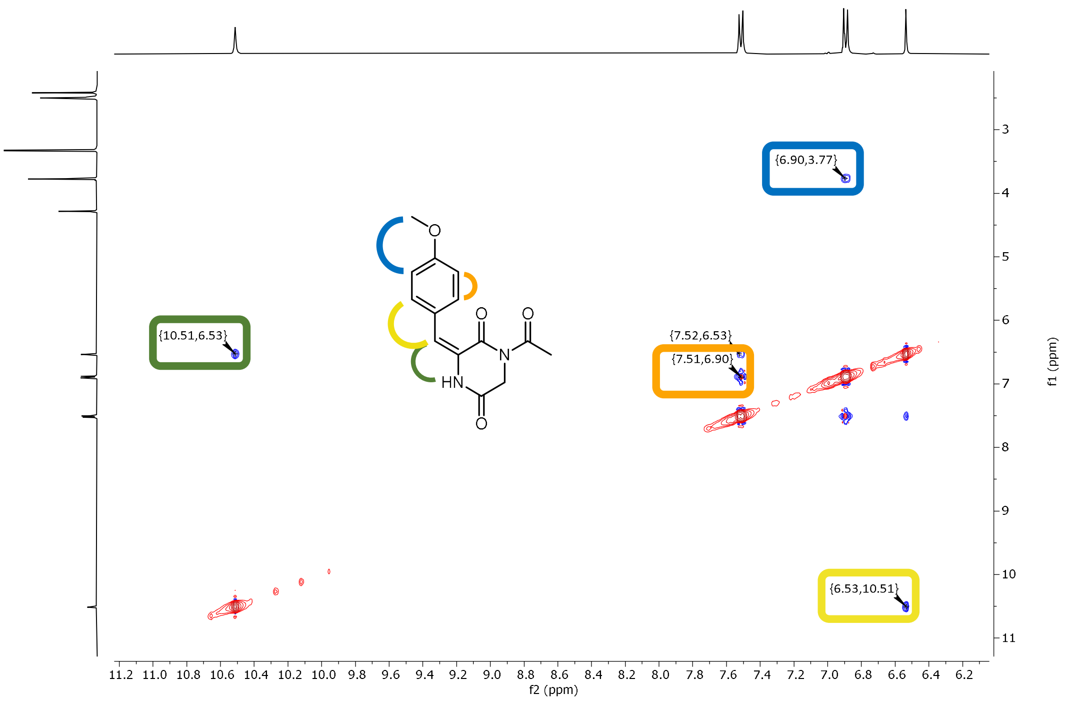

Supplementary Figure 139: NOESY NMR spectrum (400 MHz, DMSO-d_6) of compound *E*-**8**.

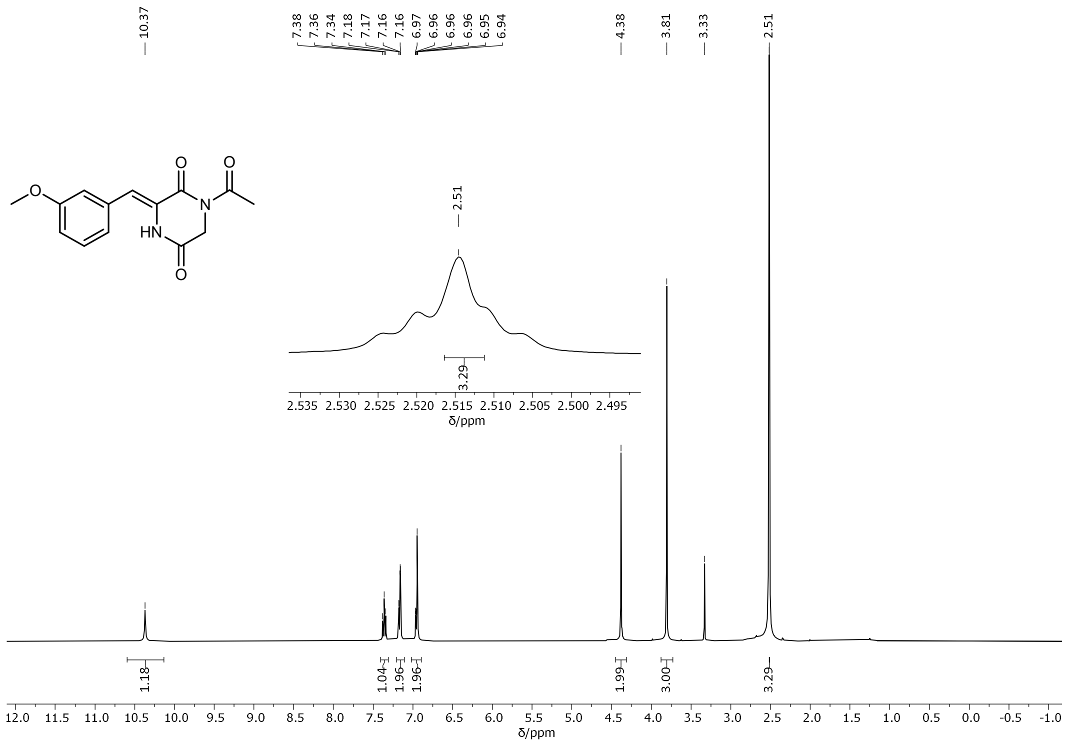

Supplementary Figure 140: ^{1}H NMR spectrum (400 MHz, DMSO-d_6) of compound Z-**9**.

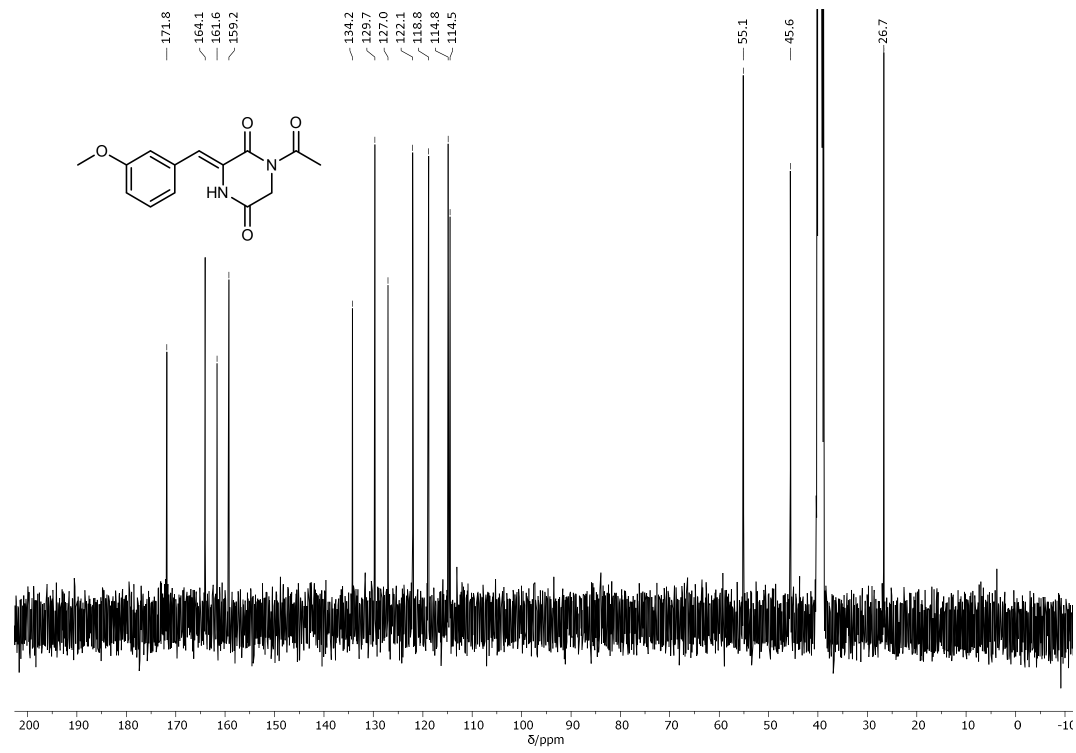

Supplementary Figure 141: ^{13}C NMR spectrum (101 MHz, DMSO-d_6) of compound Z-**9**.

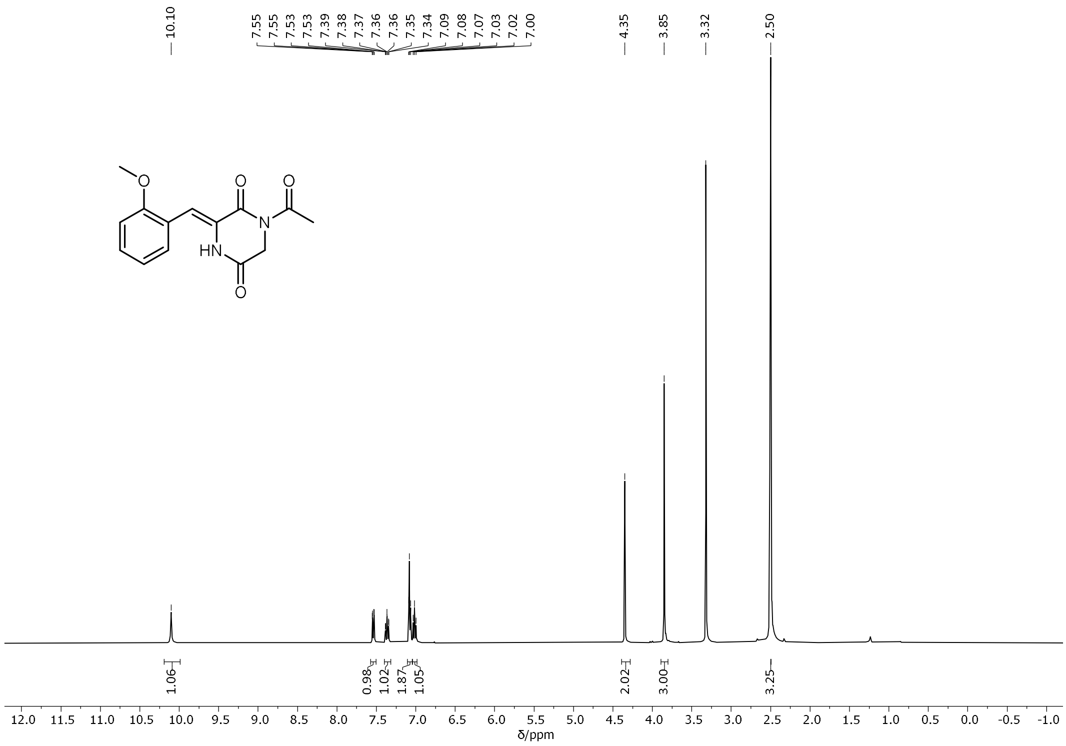

*Supplementary Figure 142: ^{1}H NMR spectrum (400 MHz, DMSO-d_6) of compound Z-**10**.*

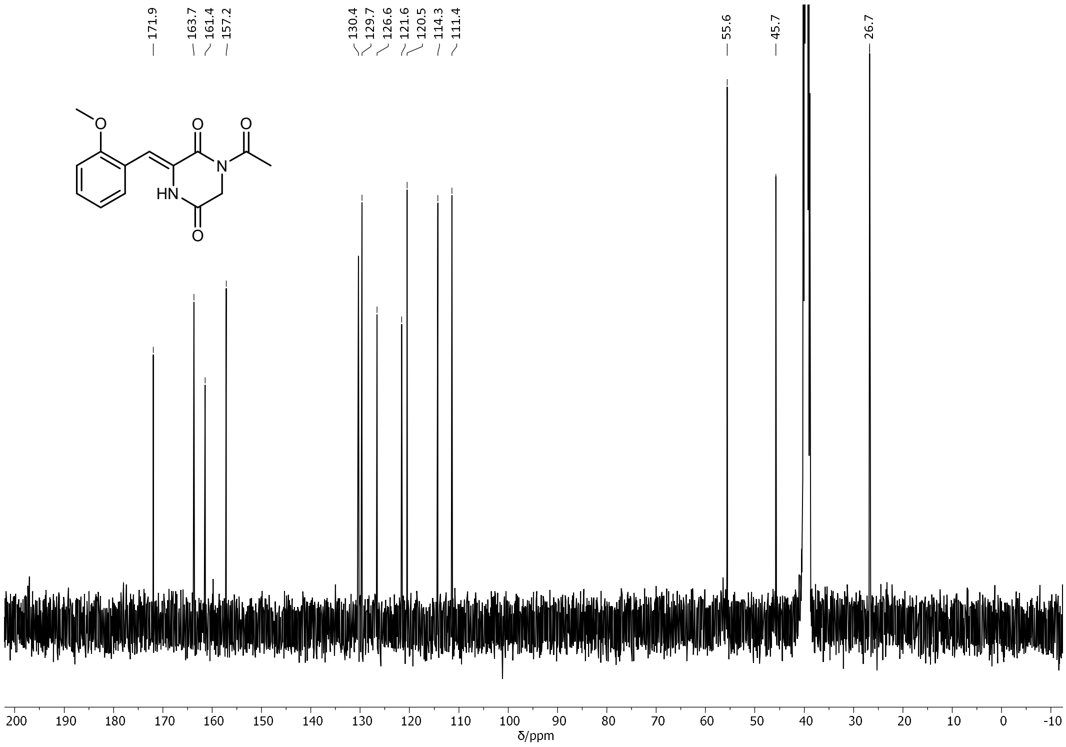

*Supplementary Figure 143: ^{13}C NMR spectrum (101 MHz, DMSO-d_6) of compound Z-**10**.*

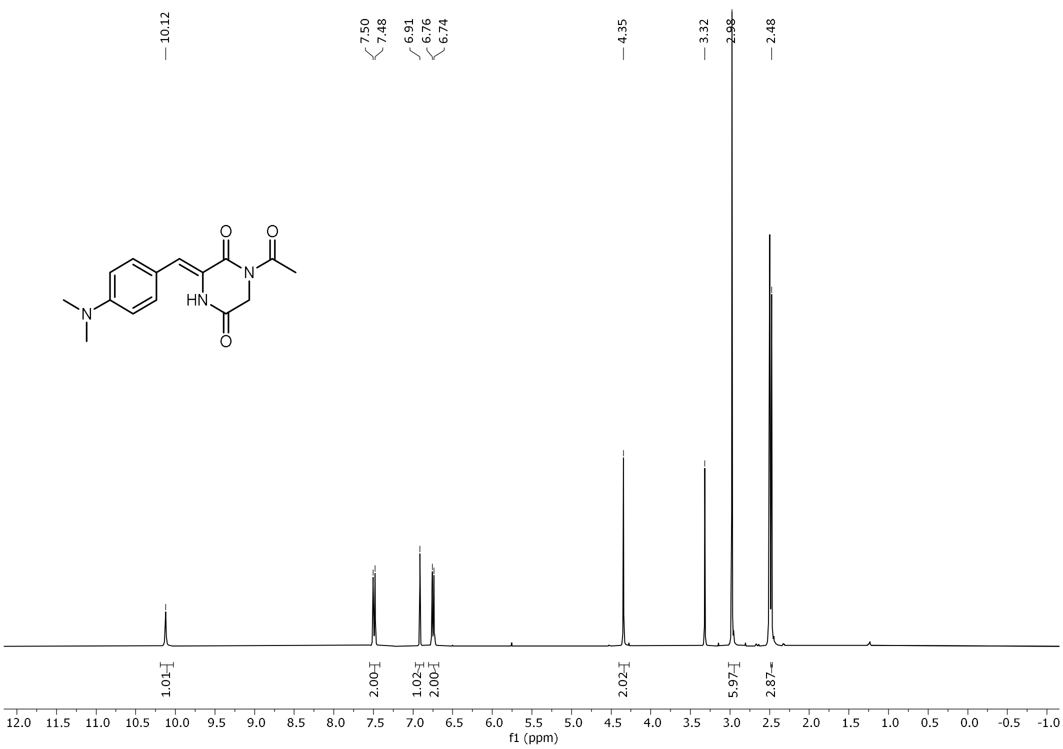

*Supplementary Figure 144: ^1H NMR spectrum (400 MHz, DMSO-d_6) of compound Z-**11**.*

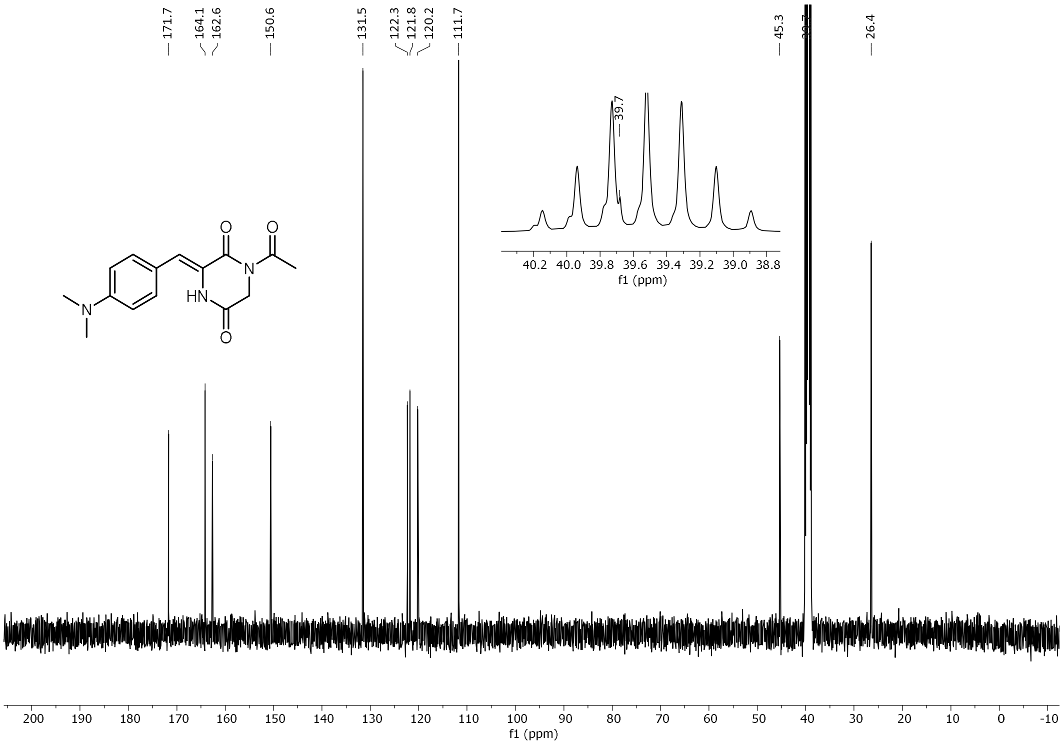

*Supplementary Figure 145: ^{13}C NMR spectrum (101 MHz, DMSO-d_6) of compound Z-**11**.*

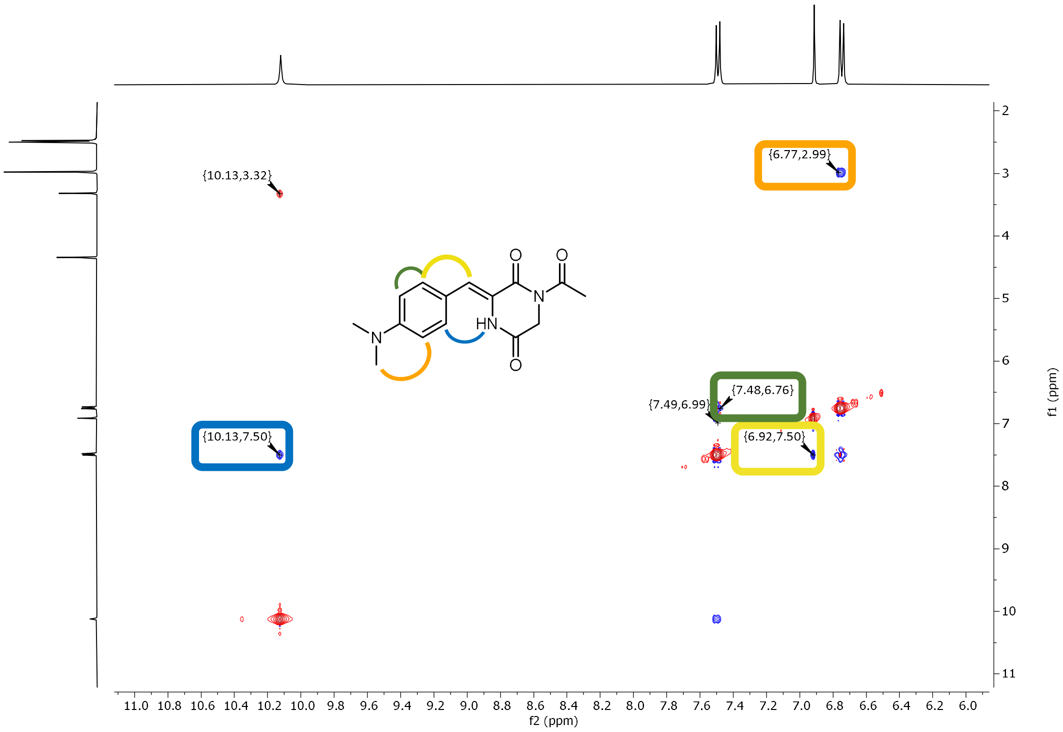

*Supplementary Figure 146: NOESY NMR spectrum (400 MHz, DMSO-d6) of compound Z-**11**.*

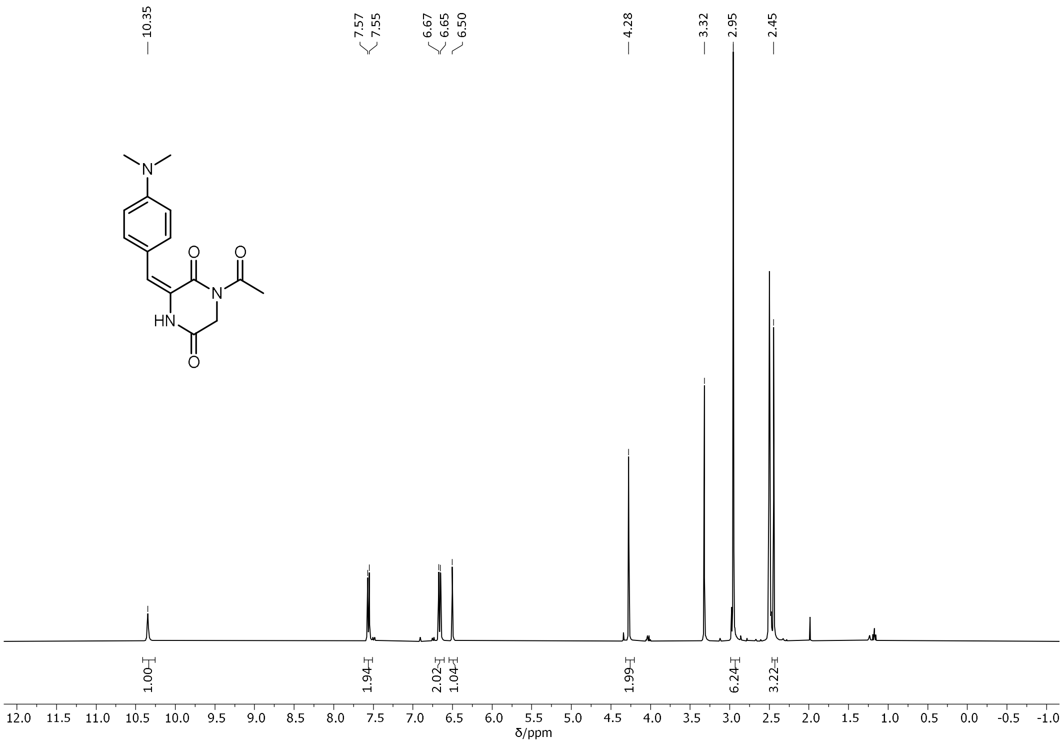

*Supplementary Figure 147: ^{1}H NMR spectrum (400 MHz, DMSO-d6) of compound E-**11**.*

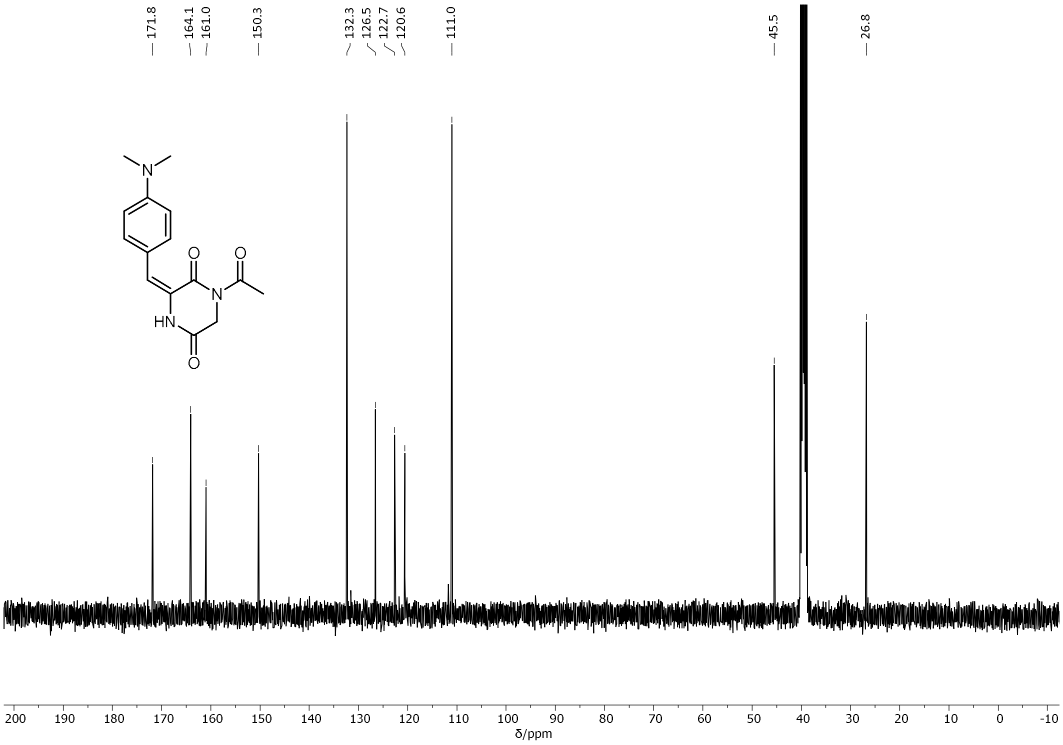

Supplementary Figure 148: ^{13}C NMR spectrum (101 MHz, DMSO-d_6) of compound *E*-**11**.

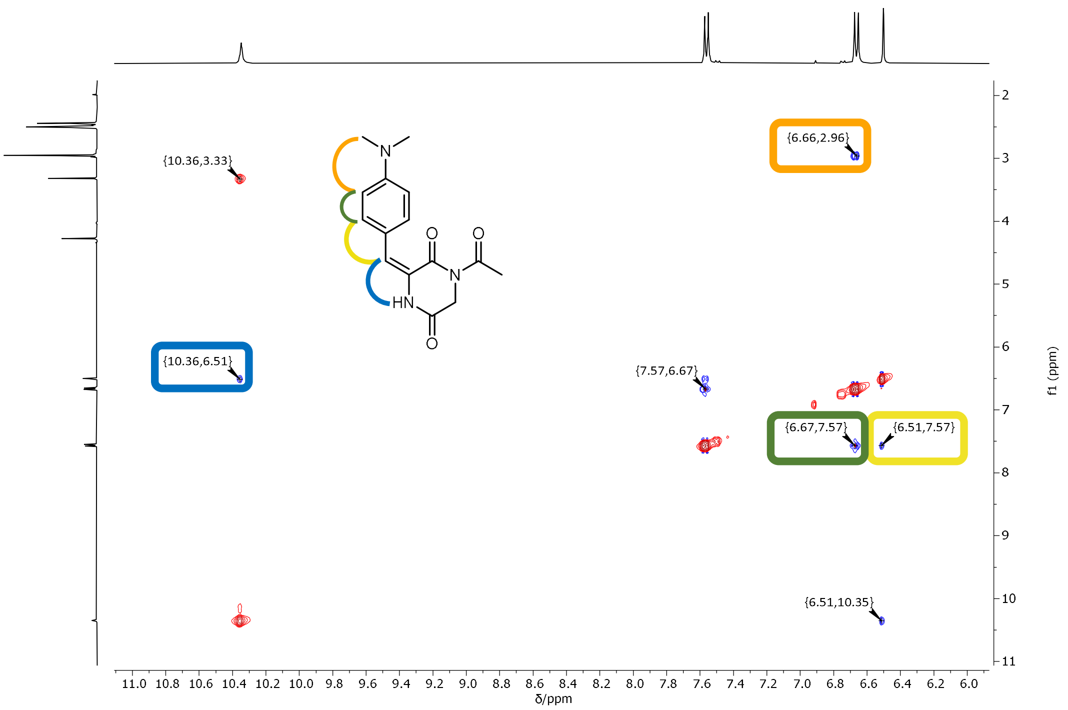

Supplementary Figure 149: NOESY NMR spectrum (400 MHz, DMSO-d_6) of compound *E*-**11**.

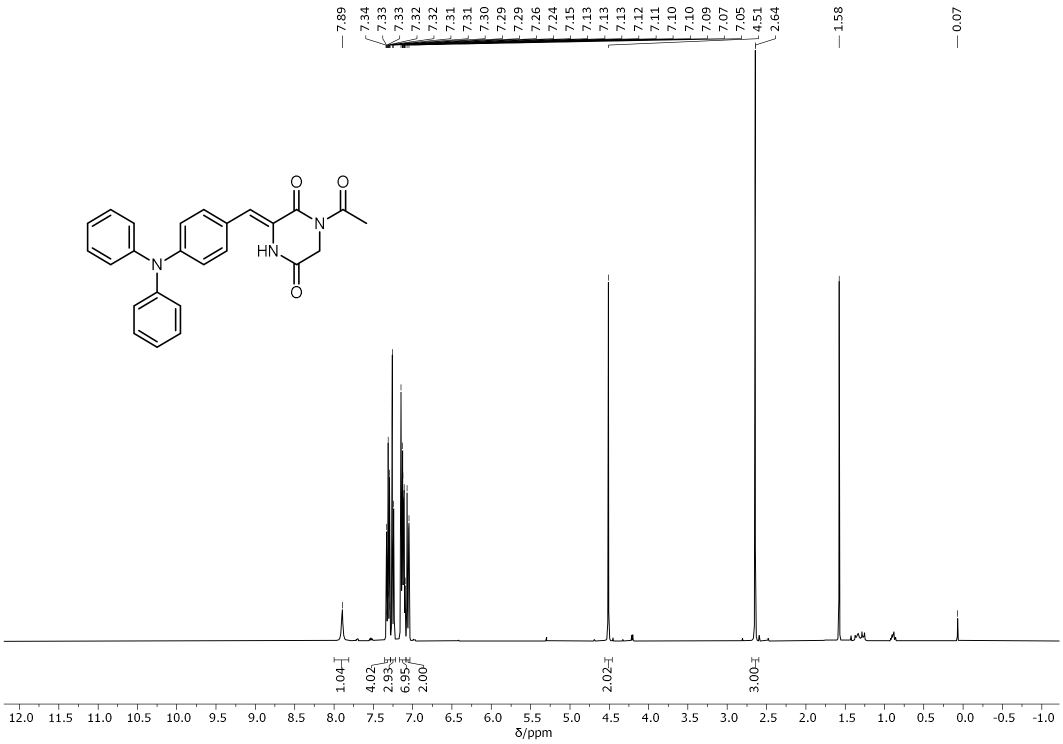

Supplementary Figure 150: ^{1}H NMR spectrum (400 MHz, $CDCl_3$) of compound Z-**12**.

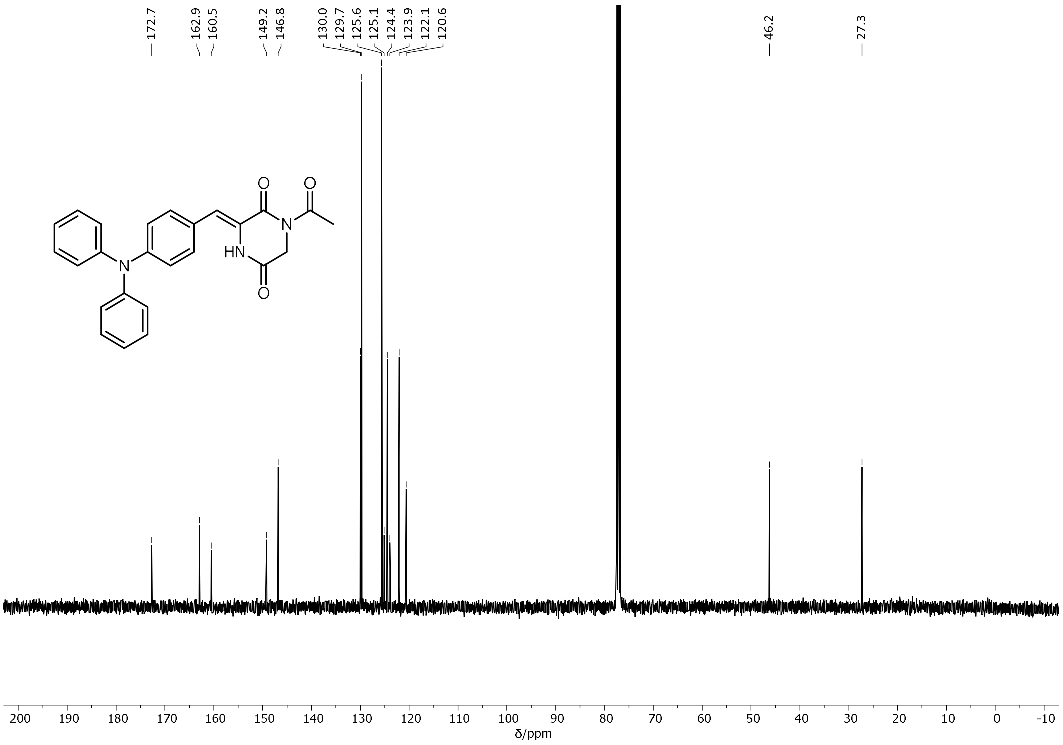

Supplementary Figure 151: ^{13}C NMR spectrum (101 MHz, $CDCl_3$) of compound Z-**12**.

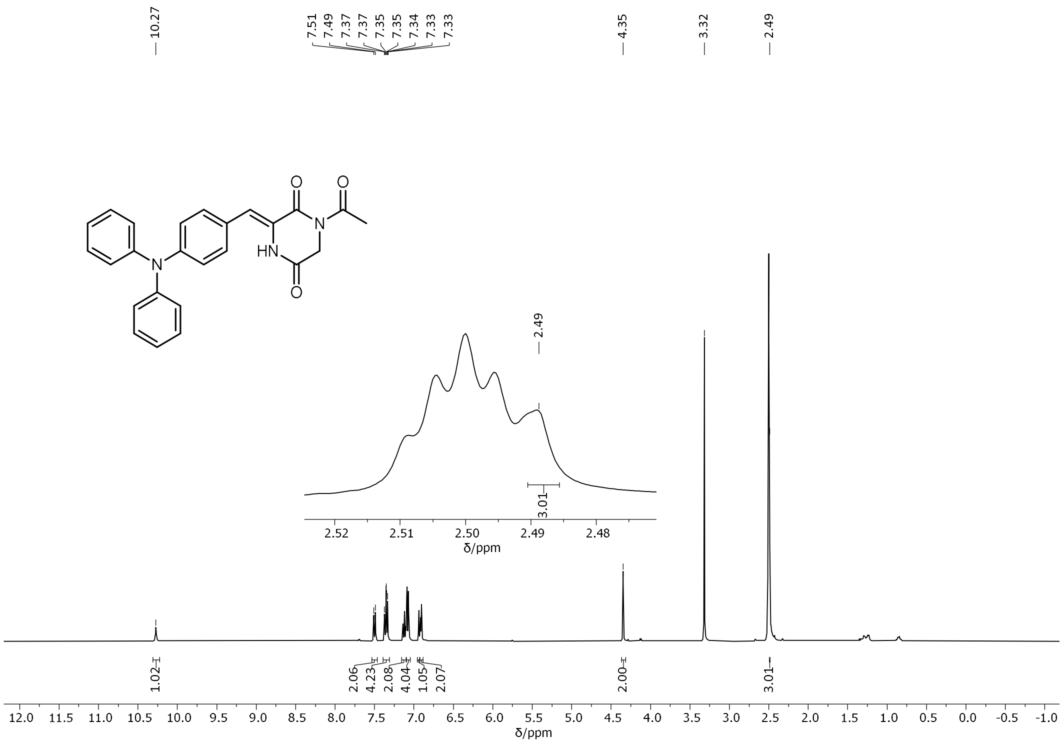

Supplementary Figure 152: ^{1}H NMR spectrum (400 MHz, DMSO-d_6) of compound Z-**12**.

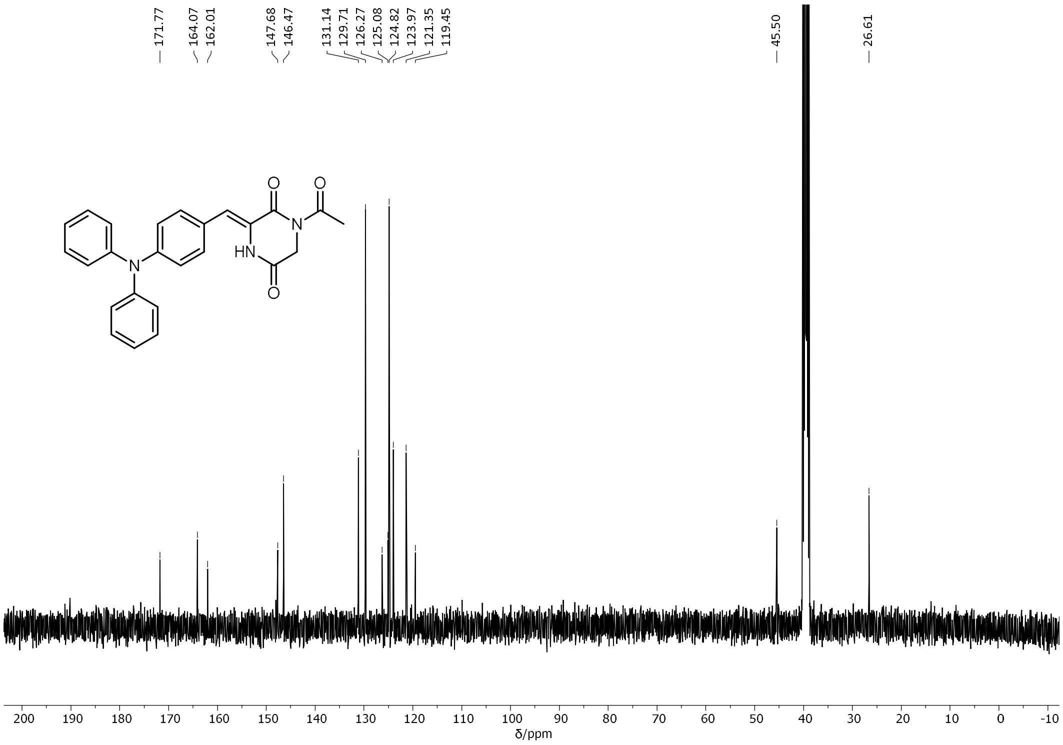

Supplementary Figure 153: ^{13}C NMR spectrum (101 MHz, DMSO-d_6) of compound Z-**12**.

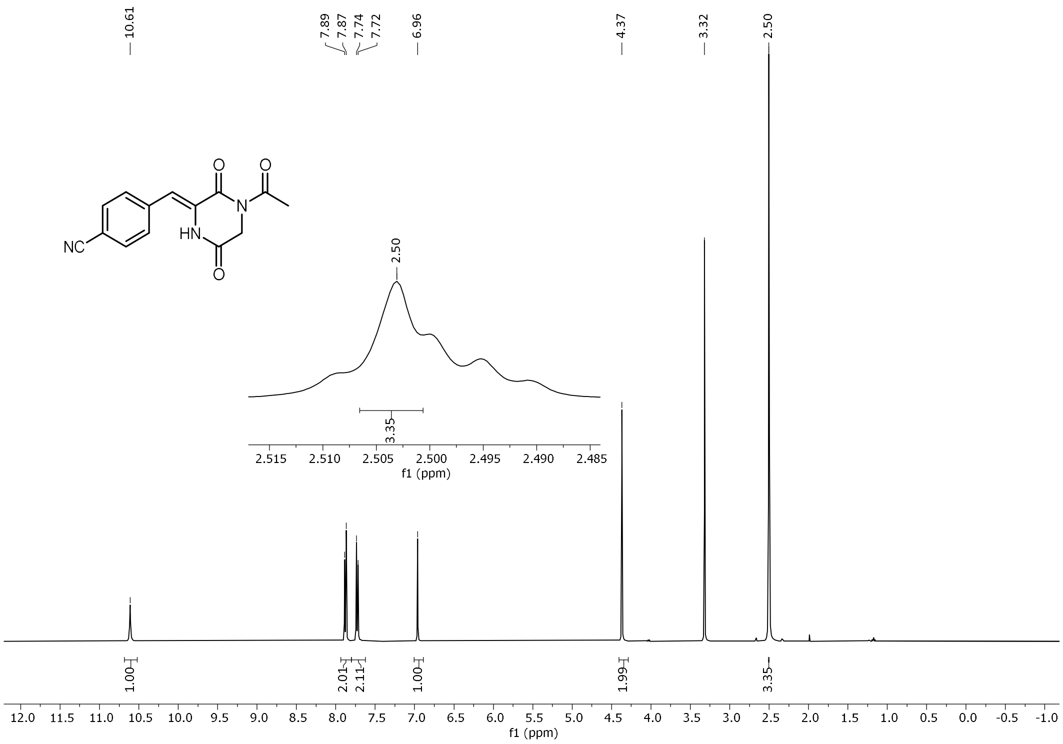

*Supplementary Figure 154: ^{1}H NMR spectrum (400 MHz, DMSO-d_6) of compound Z-**13**.*

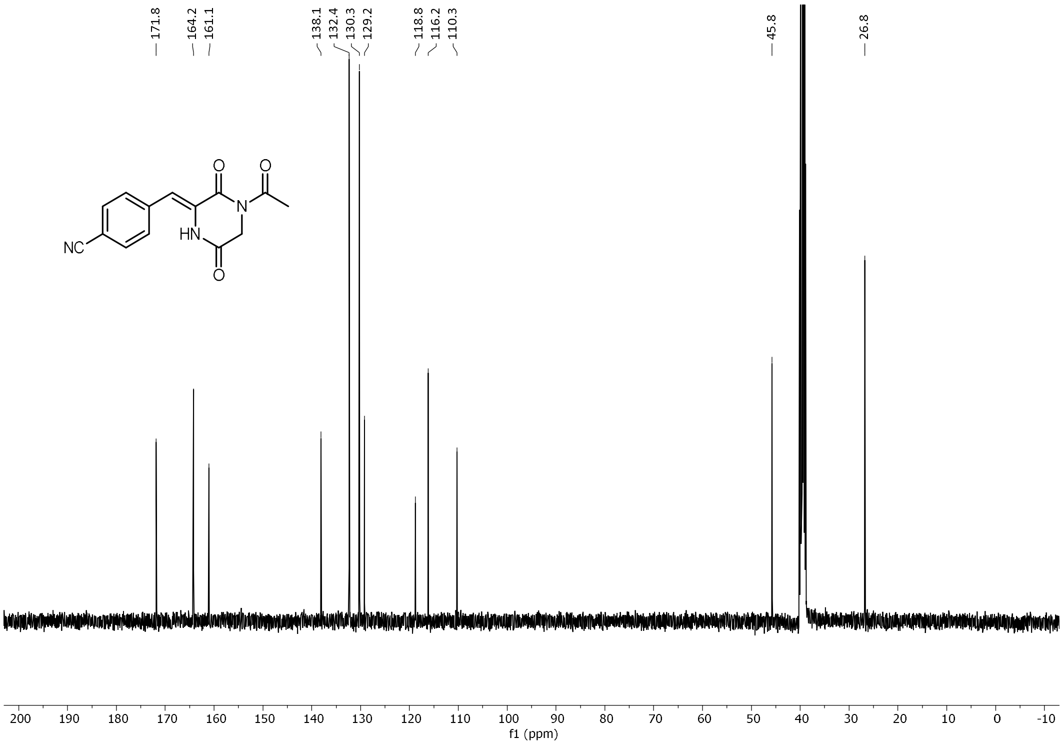

*Supplementary Figure 155: ^{13}C NMR spectrum (101 MHz, DMSO-d_6) of compound Z-**13**.*

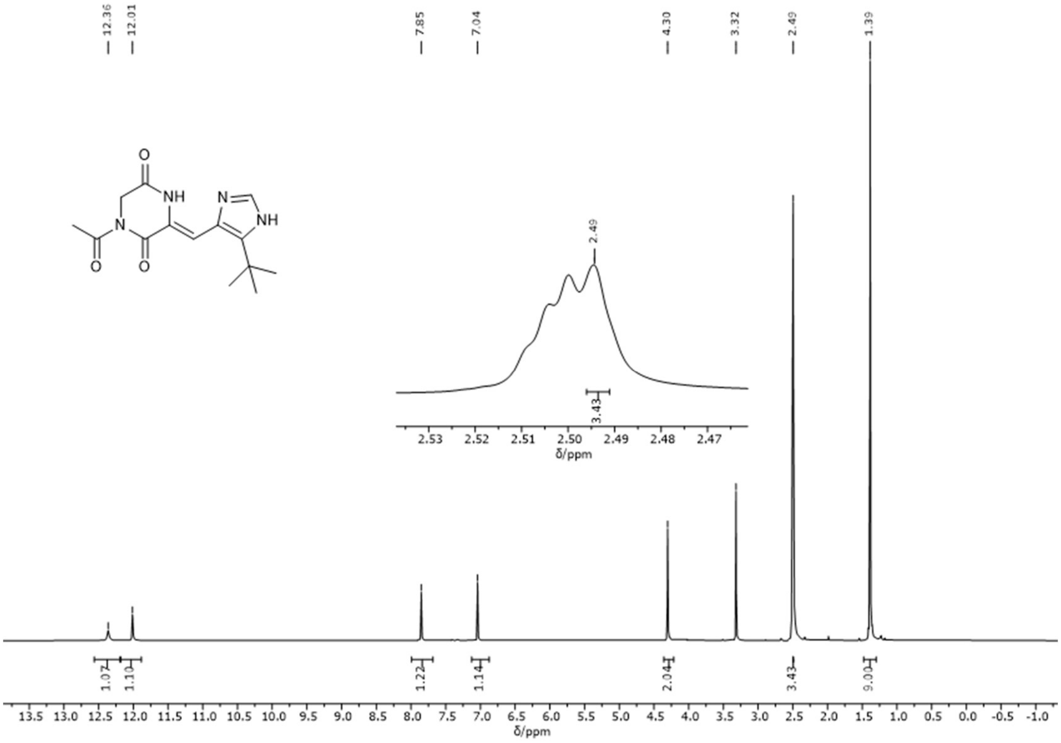

Supplementary Figure 156: ^{1}H NMR spectrum (400 MHz, DMSO-d_6) of compound Z-**14**.

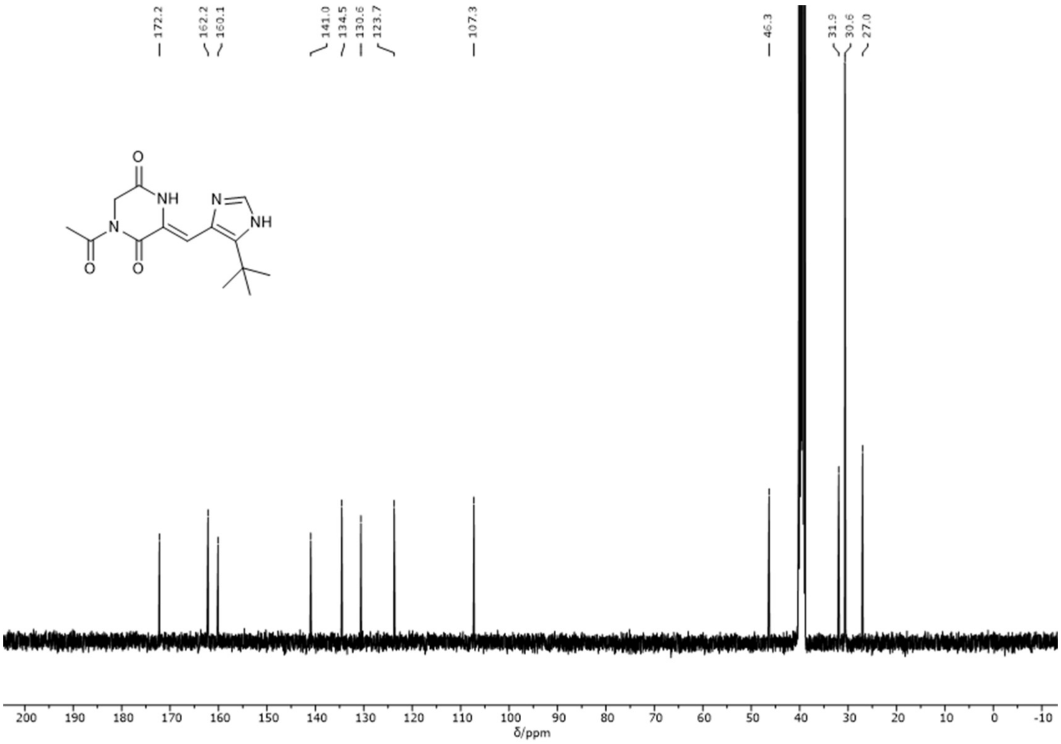

Supplementary Figure 157: ^{13}C NMR spectrum (101 MHz, DMSO-d_6) of compound Z-**14**.

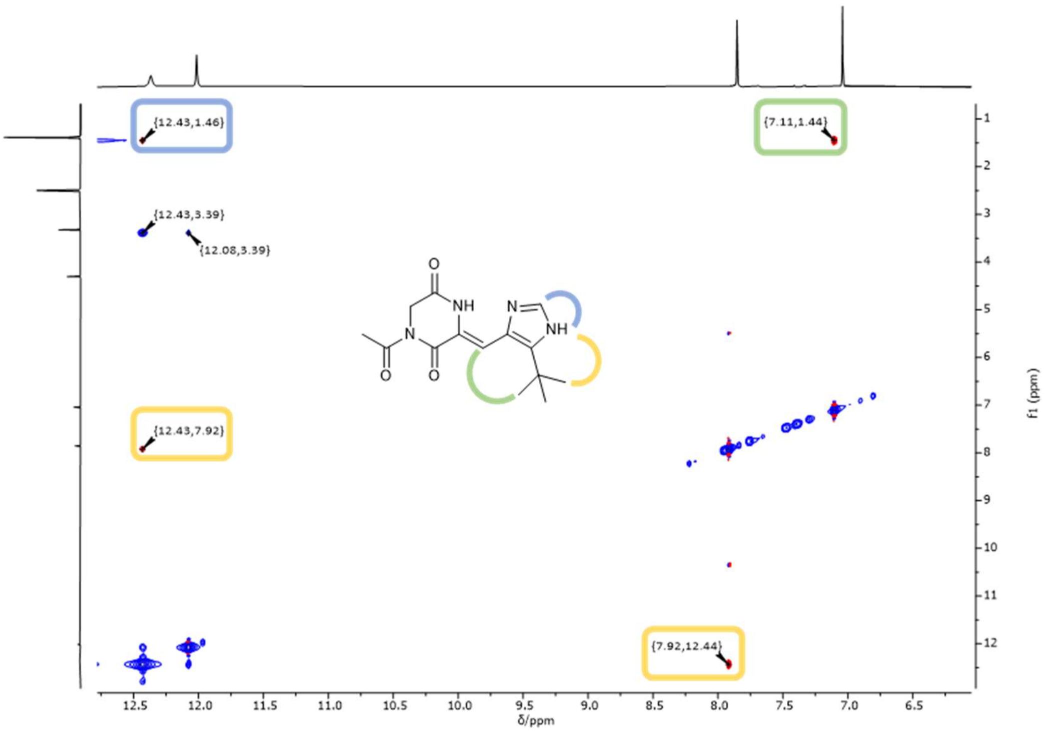

*Supplementary Figure 158: NOESY NMR spectrum (400 MHz, DMSO-d$_6$) of compound Z-**14**.*

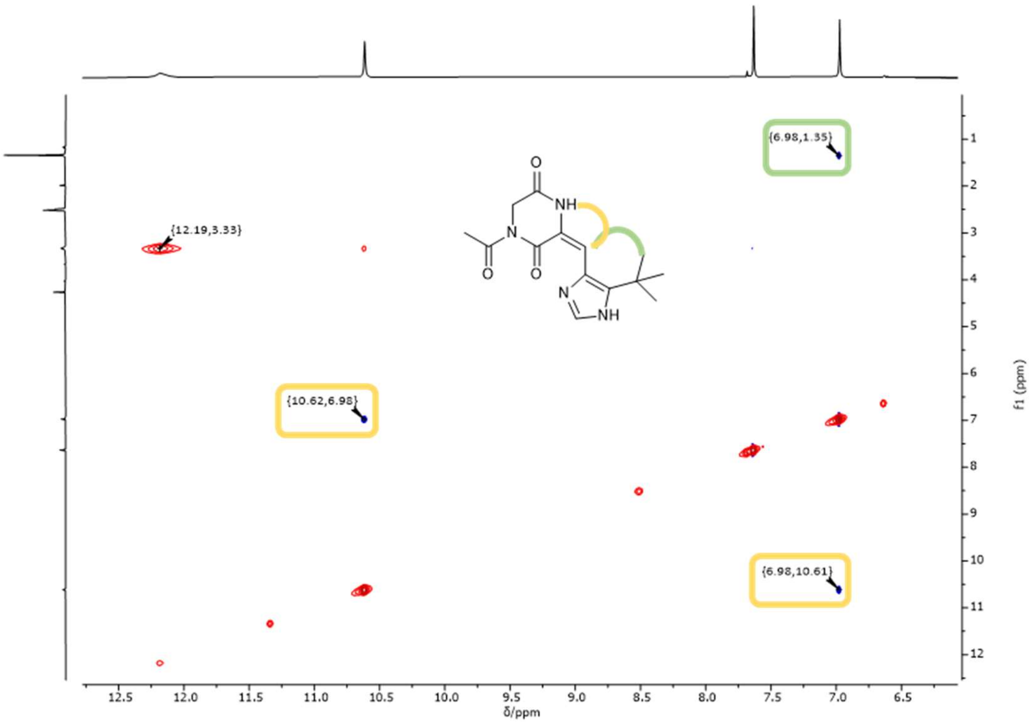

*Supplementary Figure 159: NOESY NMR spectrum (400 MHz, DMSO-d$_6$) of compound E-**14**.*

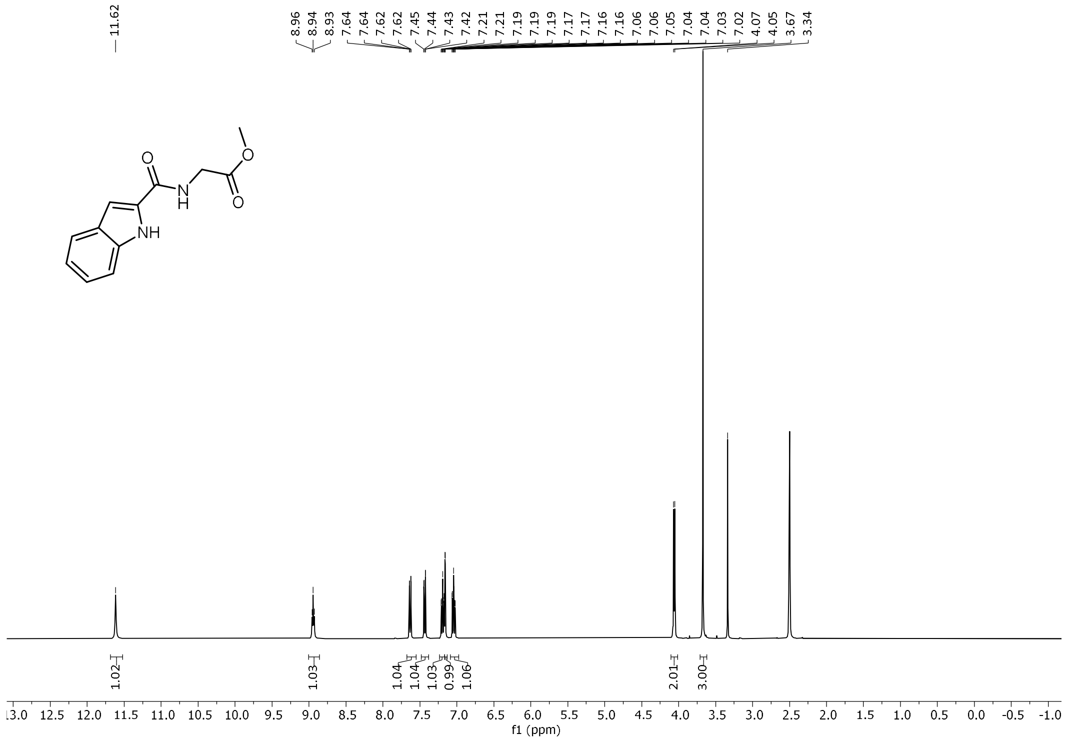

Supplementary Figure 160: ¹H NMR spectrum (400 MHz, DMSO-d₆) of methyl 2-(1H-indole-2-carbonylamino)acetate ***16***.

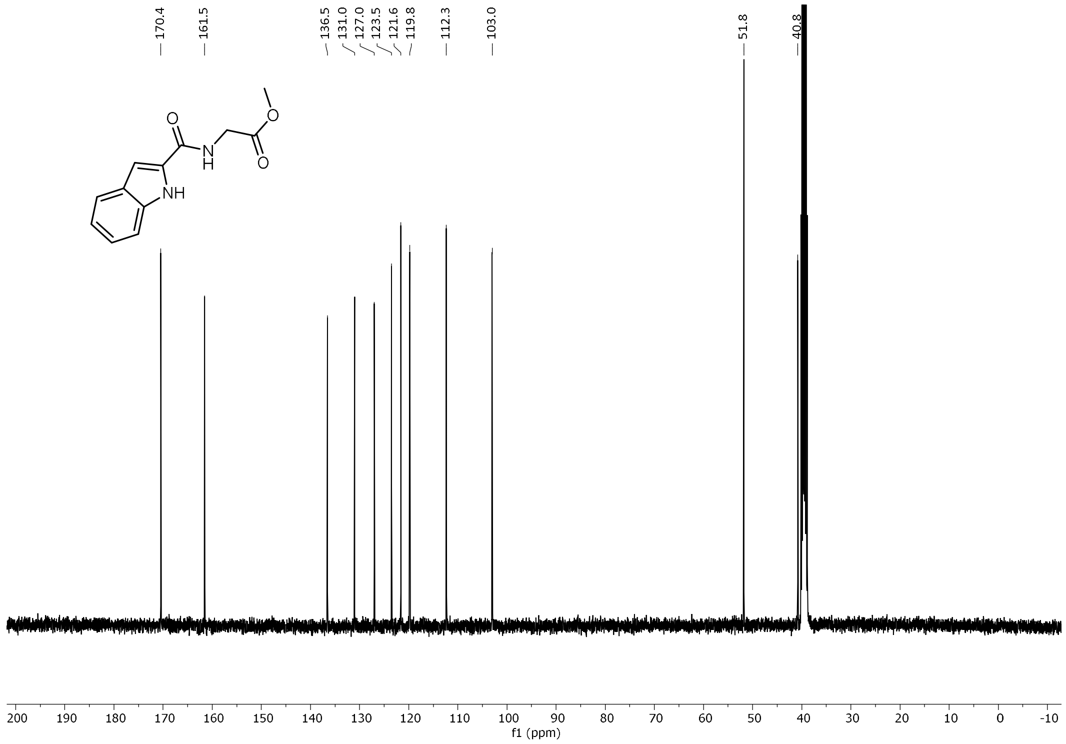

*Supplementary Figure 161: ^{13}C NMR spectrum (101 MHz, DMSO-d_6) of methyl 2-(1H-indole-2-carbonylamino)acetate **16**.*

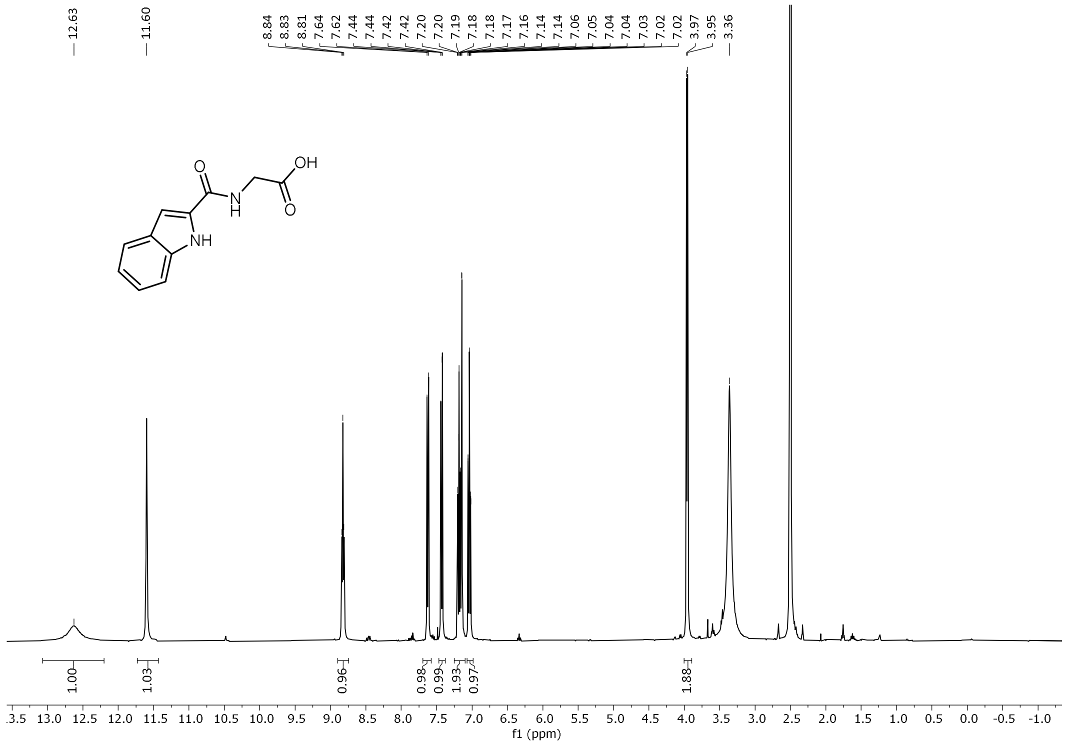

*Supplementary Figure 162: ^{1}H NMR spectrum (400 MHz, DMSO-d_6) of 2-(1H-indole-2-carbonylamino)acetic acid **17**.*

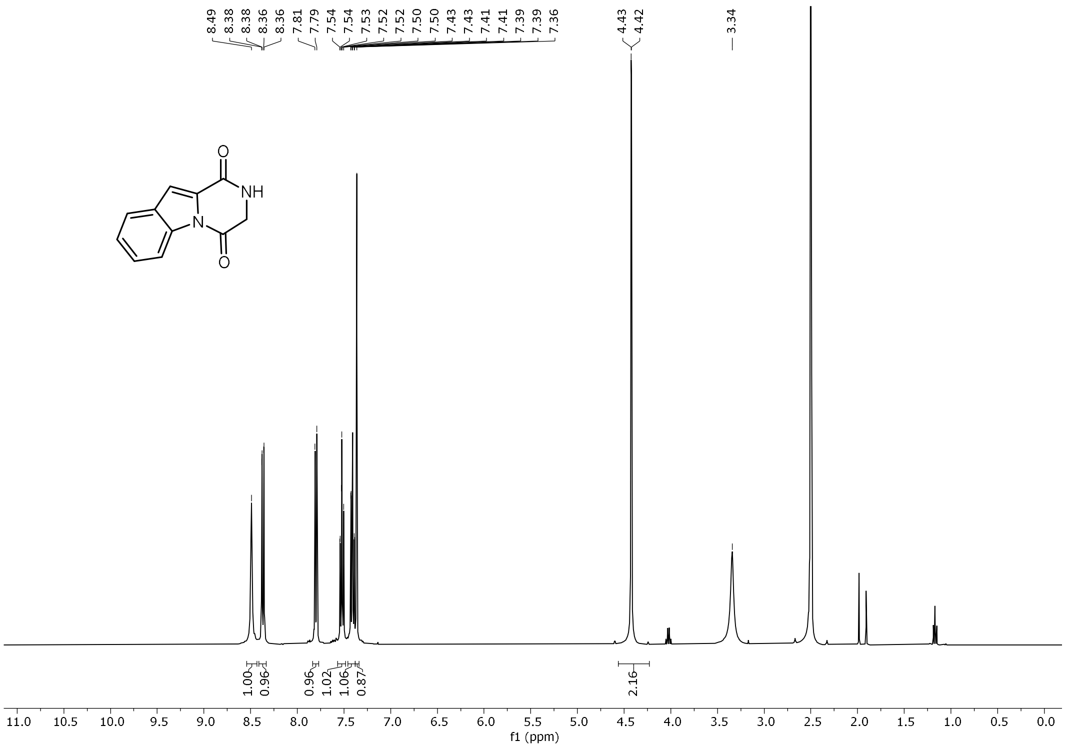

*Supplementary Figure 163: ^{1}H NMR spectrum (400 MHz, DMSO-d_6) of 2,3-dihydropyrazino[1,2-a]indole-1,4-dione **18**.*

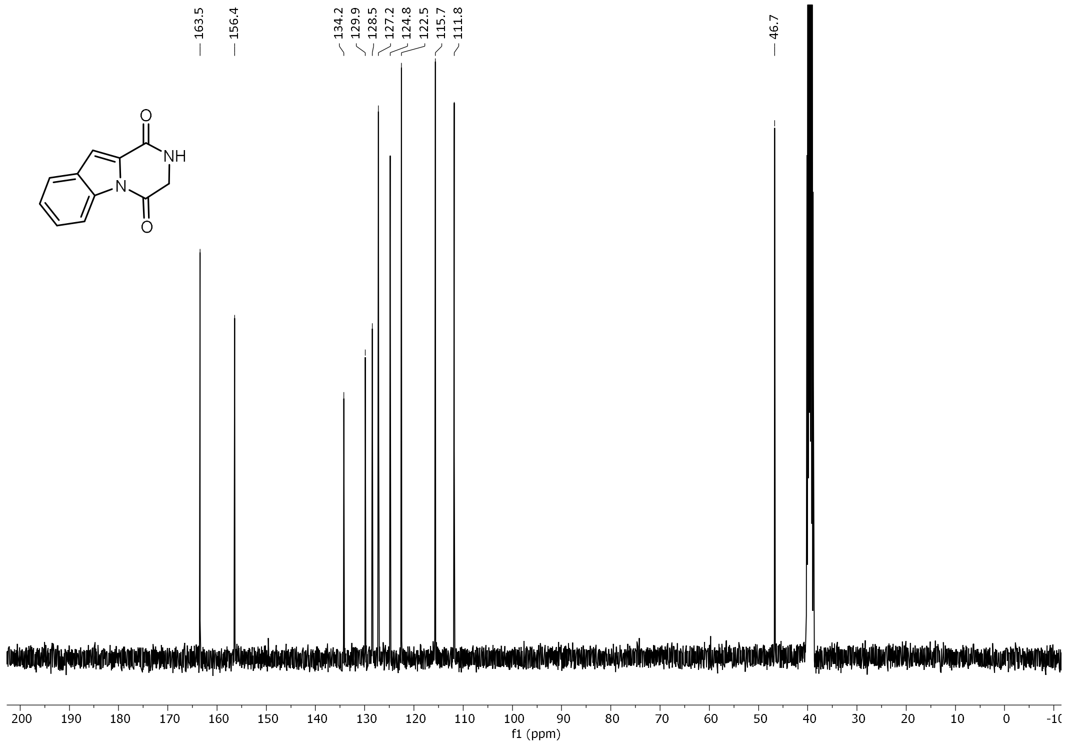

*Supplementary Figure 164: ^{13}C NMR spectrum (101 MHz, DMSO-d_6) of 2,3-dihydropyrazino[1,2-a]indole-1,4-dione **18**.*

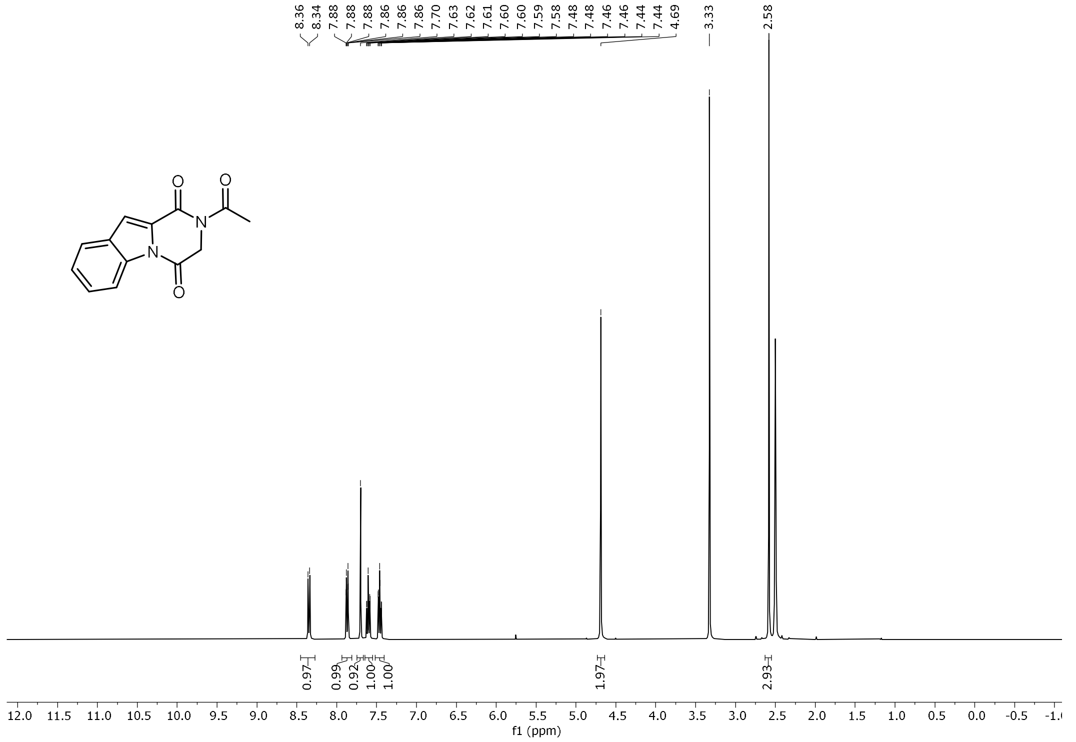

*Supplementary Figure 165: ^{1}H NMR spectrum (400 MHz, DMSO-d_6) of 2-acetyl-2,3-dihydropyrazino[1,2-a]indole-1,4-dione **19**.*

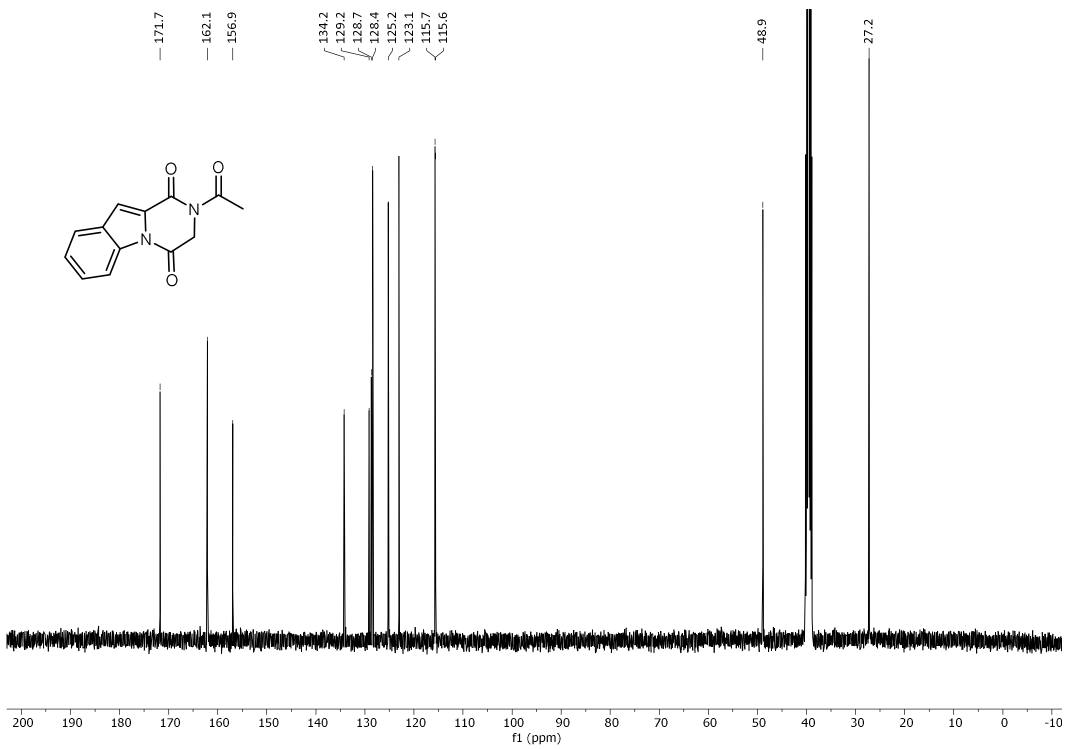

Supplementary Figure 166: ^{13}C NMR spectrum (101 MHz, DMSO-d_6) of 2-acetyl-2,3-dihydropyrazino[1,2-a]indole-1,4-dione **19**.

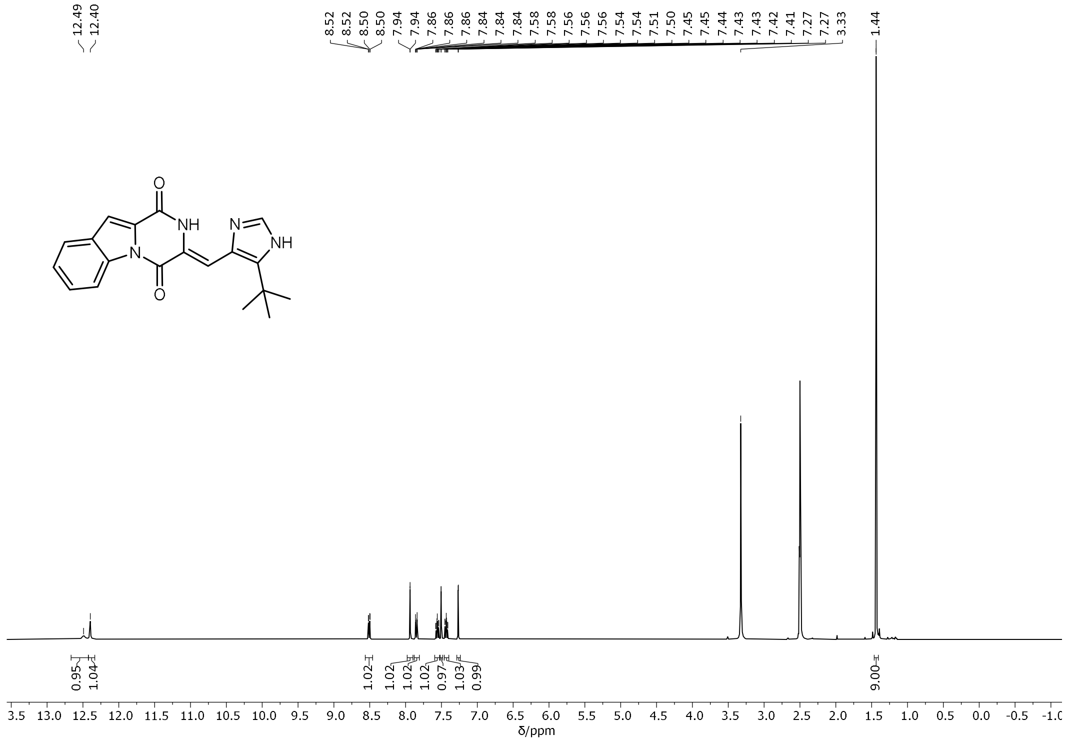

Supplementary Figure 167: ^{1}H NMR spectrum (400 MHz, DMSO-d_6) of compound Z-**15**.

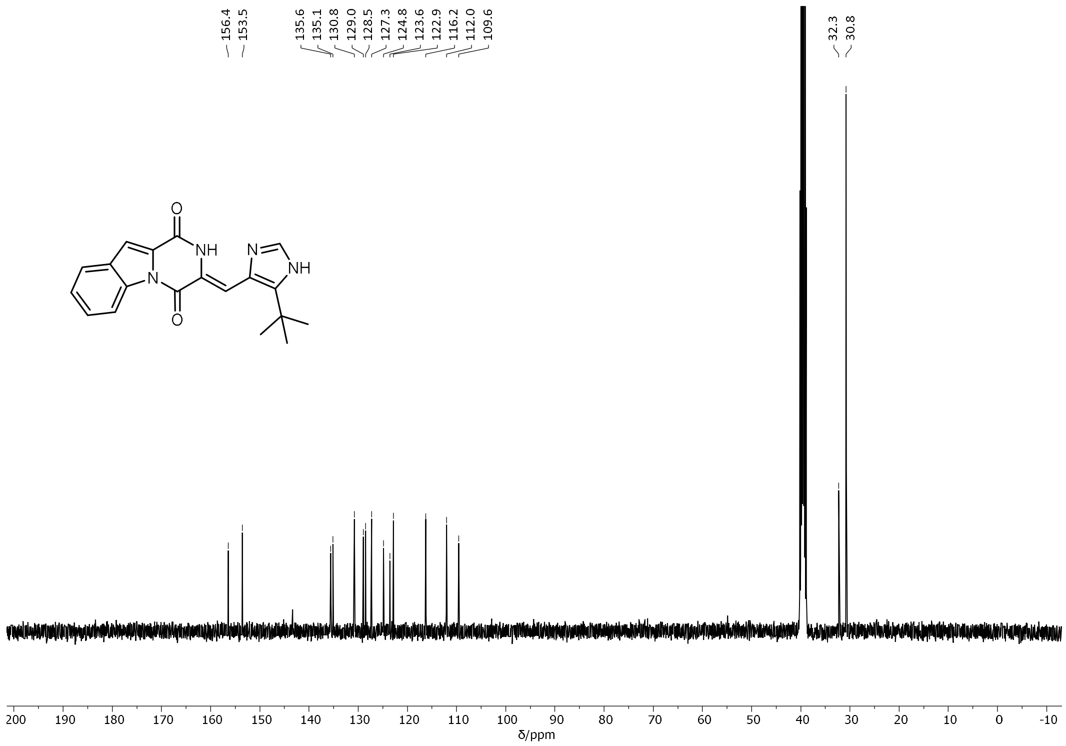

*Supplementary Figure 168: ^{13}C NMR spectrum (101 MHz, DMSO-d_6) of compound Z-**15**.*

Theoretically Obtained Geometries

All calculations were performed with the GAUSSIAN09 program package, employing the B3LYP-GD3BJ/6-311G(d,p) PCM(DMSO) level of theory (these parameters were successfully applied for calculations in another arylidene-substituted heterocyclic photochromic system – hemiindigos[34]). The global minima geometries were first optimized on the MPW1K/6-311G(d,p) and subsequently on the B3LYP-GD3BJ/6-311G(d,p). Frequency analysis confirmed all structures to be minimum structures since no imaginary frequencies have been found. Visualization of minimized structures, molecular orbitals, and calculated extinction spectra were done with GaussView 6.1.1.

Cartesian Coordinates of the optimized structures

The presented data were obtained and visualized with Avogadro 1.2.0.

Z-Ph-DKP-Ac (7)

C	0.01014	-0.25951	0.03984
N	0.04214	108.230	-0.36206
C	111.005	191.026	-0.21019

C	230.061	126.510	0.47215
N	245.572	-0.15556	0.11808
C	131.778	-0.97876	0.17524
O	136.902	-218.065	0.32721
O	110.948	307.763	-0.55863
C	-112.539	-0.96717	0.20852
C	374.057	-0.70190	-0.13105
O	387.757	-179.366	-0.63218
C	492.389	0.16076	0.24686
C	-251.118	-0.51741	0.12356
C	-349.068	-145.816	-0.24290
C	-482.790	-109.607	-0.34504
C	-522.222	0.21127	-0.06154
C	-426.879	114.834	0.33416
C	-292.775	0.79250	0.42568
H	-0.77902	147.865	-0.80240
H	317.274	182.410	0.15783
H	218.960	138.410	155.537
H	-0.97102	-202.537	0.38099
H	482.198	0.58722	124.602
H	581.010	-0.46798	0.20277
H	504.976	0.98273	-0.46277
H	-318.857	-247.722	-0.45578
H	-556.419	-183.437	-0.63954
H	-626.528	0.49428	-0.13631
H	-457.092	215.921	0.58058
H	-221.367	152.540	0.77807

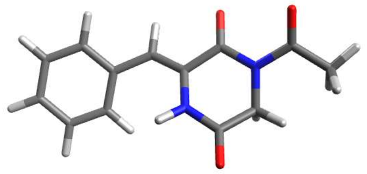

***E*-Ph-DKP-Ac (7)**

C	-0.08584	0.81837	-0.31780
N	-0.96407	191.055	-0.54488
C	-220.371	201.006	-0.00111
C	-260.200	0.77604	0.78569
N	-211.491	-0.45296	0.14039
C	-0.75711	-0.51221	-0.22479
O	-0.18519	-155.758	-0.45101
O	-292.054	299.015	-0.10953
C	124.055	109.532	-0.25838
C	-297.551	-156.766	-0.03643
O	-268.951	-248.271	-0.77260
C	-427.559	-154.349	0.73748
C	248.645	0.35497	-0.08142
C	363.185	115.486	0.12747
C	488.323	0.59212	0.33511
C	503.141	-0.79406	0.33117
C	391.543	-160.061	0.11375
C	265.888	-104.242	-0.09269
H	-0.60659	272.923	-102.070
H	-220.599	0.86147	180.317
H	-368.230	0.76930	0.83961
H	143.150	216.387	-0.32562
H	-498.275	-0.84000	0.28993
H	-470.343	-254.159	0.67876

H	-413.139	-126.361	178.207
H	352.715	223.397	0.13198
H	574.151	123.334	0.49675
H	600.632	-123.994	0.48886
H	402.390	-267.895	0.09849
H	180.849	-167.685	-0.27512

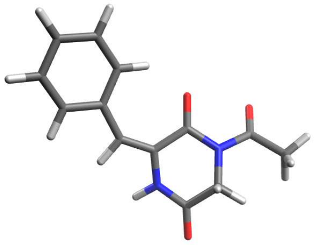

***Z-p*-OMe-Ph-DKP-Ac (8)**

C	347.744	112.595	-0.35417
C	443.380	0.14264	-0.05949
C	401.735	-117.443	0.16566
C	266.485	-148.395	0.10624
C	168.685	-0.50918	-0.15231
C	213.358	0.80782	-0.39700
C	0.29615	-0.92808	-0.19591
C	-0.83204	-0.20395	-0.02619
N	-0.84634	114.625	0.35772
C	-190.689	198.475	0.20493
C	-309.632	135.233	-0.48312
N	-326.384	-0.06832	-0.13640
C	-214.229	-0.90554	-0.14441
O	-221.858	-211.755	-0.23681
O	-189.359	315.160	0.55789
C	-459.521	-0.51703	0.02389

C	-484.529	-192.381	0.49704
O	-550.683	0.25911	-0.18762
O	572.179	0.55878	-0.02924
C	674.093	-0.40229	0.25907
H	381.734	213.415	-0.55537
H	473.149	-195.698	0.37839
H	235.409	-250.890	0.27360
H	143.178	158.410	-0.67096
H	0.12208	-198.668	-0.34729
H	-0.03212	152.760	0.82237
H	-399.315	188.220	-0.18358
H	-298.112	146.675	-156.631
H	-418.856	-220.103	132.067
H	-588.883	-197.656	0.80228
H	-465.869	-263.015	-0.31197
H	767.862	0.14776	0.23660
H	659.733	-0.84218	124.974
H	676.129	-119.239	-0.49653

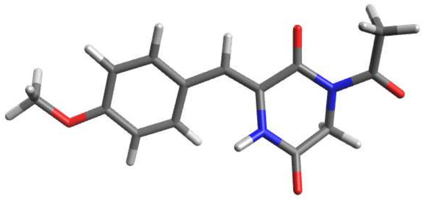

***E*-*p*-OMe-Ph-DKP-Ac (8)**

C	-315.280	-120.594	0.38847
C	-426.358	-0.37969	0.15743
C	-405.788	0.95758	-0.19667
C	-276.064	143.792	-0.31178
C	-162.952	0.62466	-0.10201

C	-186.705	-0.72019	0.25854
C	-0.34269	128.996	-0.22511
C	0.96589	0.92760	-0.21713
N	192.806	195.754	-0.39419
C	318.296	193.025	0.12161
C	350.445	0.63417	0.83325
N	287.879	-0.52057	0.17213
C	152.134	-0.45018	-0.16523
O	0.86015	-143.844	-0.42828
O	396.896	286.090	0.04500
C	368.571	-167.370	0.02298
C	316.316	-284.571	-0.76358
O	480.065	-167.756	0.50772
O	-547.739	-0.96063	0.30280
C	-664.370	-0.16566	0.07218
H	-332.886	-223.771	0.66728
H	-488.887	162.488	-0.37499
H	-261.745	248.043	-0.57281
H	-103.576	-138.436	0.42423
H	-0.46719	236.460	-0.33998
H	163.216	282.415	-0.82537
H	457.760	0.48430	0.83422
H	316.581	0.70574	187.231
H	269.373	-253.565	-169.623
H	401.041	-350.090	-0.95798
H	240.623	-337.799	-0.18740
H	-748.942	-0.82893	0.23711
H	-669.279	0.67230	0.77285

H	-666.641	0.20974	-0.95452

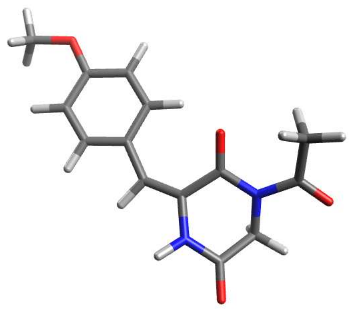

***Z-m*-OMe-Ph-DKP-Ac (9)**

C	335.422	203.008	-0.46303
C	444.179	122.304	-0.16475
C	425.265	-0.14982	0.04806
C	297.206	-0.69260	-0.03521
C	186.275	0.13033	-0.30955
C	206.699	150.032	-0.53487
C	0.55362	-0.51246	-0.38123
C	-0.66077	0.01183	-0.11709
N	-0.86178	130.191	0.38679
C	-204.937	196.725	0.37880
C	-318.460	121.565	-0.27797
N	-309.158	-0.24403	-0.11095
C	-185.588	-0.87696	-0.26040
O	-173.921	-206.791	-0.48100
O	-218.396	309.322	0.82468
C	-432.544	-0.92644	0.02924
C	-432.360	-240.097	0.32843
O	-535.715	-0.29061	-0.05783
O	537.374	-0.86697	0.32245
C	524.123	-227.239	0.54097

H	350.935	308.608	-0.64887
H	544.501	162.576	-0.10286
H	280.721	-174.960	0.11913
H	124.448	214.305	-0.81729
H	0.54373	-156.669	-0.62927
H	-0.07380	178.818	0.79710
H	-411.774	154.861	0.16218
H	-320.546	147.430	-134.218
H	-359.471	-266.180	109.469
H	-533.042	-266.160	0.64982
H	-406.170	-296.504	-0.56685
H	624.651	-263.617	0.74065
H	459.965	-247.926	140.248
H	484.014	-277.400	-0.34451

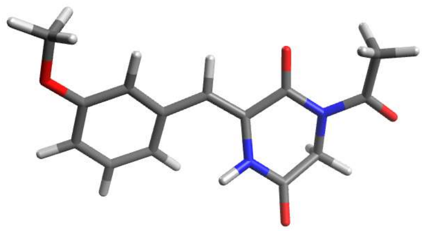

***E*-*m*-OMe-Ph-DKP-Ac (9)**

C	-292.573	-227.214	-0.13940
C	-413.474	-163.935	0.10903
C	-418.423	-0.24022	0.15935
C	-302.054	0.49644	-0.03770
C	-178.131	-0.14522	-0.27110
C	-175.123	-154.676	-0.33030
C	-0.65322	0.76692	-0.44558
C	0.70048	0.68791	-0.42393

N	142.337	187.694	-0.69890
C	260.945	220.139	-0.11904
C	314.859	111.174	0.78088
N	284.613	-0.22895	0.25861
C	154.661	-0.51398	-0.17443
O	115.203	-165.147	-0.34920
O	317.158	327.087	-0.28146
C	389.511	-117.979	0.33160
C	371.863	-252.987	-0.30915
O	493.105	-0.86660	0.88409
O	-540.877	0.30386	0.39917
C	-551.741	172.622	0.45223
H	-289.486	-335.428	-0.19063
H	-504.951	-219.980	0.25724
H	-304.730	157.653	-0.00609
H	-0.82373	-205.235	-0.53302
H	-0.99406	178.921	-0.59167
H	0.97216	259.904	-124.593
H	422.483	121.184	0.85631
H	272.160	123.520	178.170
H	329.313	-245.469	-130.908
H	470.206	-299.523	-0.34316
H	303.768	-314.220	0.28208
H	-656.626	193.557	0.65015
H	-490.431	214.060	125.814
H	-522.780	218.156	-0.49955

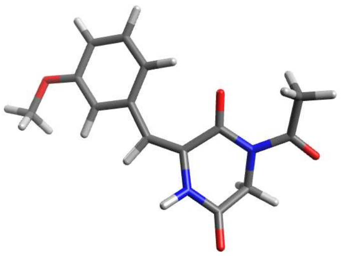

***Z-o*-OMe-Ph-DKP-Ac (10)**

C	422.552	0.33703	0.07527
C	491.771	-0.72522	-0.49908
C	424.774	-189.706	-0.83982
C	288.215	-198.630	-0.61235
C	213.858	-0.92114	-0.07140
C	285.142	0.24392	0.30092
C	0.70424	-114.989	0.08360
C	-0.36613	-0.32960	-0.00165
N	-0.30600	104.970	-0.21176
C	-132.496	180.826	-0.68695
C	-258.354	104.293	-102.716
N	-279.865	-0.13273	-0.16817
C	-171.708	-0.95479	0.15860
O	-185.130	-209.828	0.55483
O	-123.145	301.179	-0.87173
C	-414.825	-0.43585	0.13202
C	-445.948	-153.639	111.025
O	-502.539	0.23164	-0.38061
O	214.104	123.443	0.91734
C	283.496	237.711	143.512
H	476.392	123.036	0.35642
H	598.430	-0.63419	-0.66512

H	478.321	-273.302	-127.183
H	235.286	-289.642	-0.86993
H	0.43152	-218.908	0.22362
H	0.56710	151.123	0.03374
H	-343.301	170.691	-0.91323
H	-253.557	0.73955	-207.868
H	-381.309	-150.084	198.618
H	-550.341	-142.114	139.660
H	-430.863	-250.870	0.64111
H	207.241	298.425	191.682
H	358.300	207.052	216.907
H	330.734	294.715	0.63197

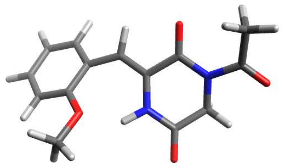

***E*-*o*-OMe-Ph-DKP-Ac (10)**

C	-338.453	-105.045	0.53777
C	-451.880	-0.52195	-0.07812
C	-447.790	0.73419	-0.67352
C	-328.627	145.302	-0.65912
C	-212.010	0.93470	-0.08566
C	-218.998	-0.32950	0.54751
C	-0.90571	175.548	-0.07981
C	0.36579	136.089	-0.25012
N	144.358	226.799	-0.15394
C	263.960	190.339	0.38401
C	269.432	0.43860	0.77927

N	199.706	-0.43635	-0.17802
C	0.76457	-0.02111	-0.68264
O	0.08796	-0.66743	-145.370
O	356.200	267.567	0.57884
C	261.462	-168.178	-0.45155
C	197.546	-262.170	-143.568
O	365.101	-195.932	0.11949
O	-106.057	-0.76087	116.754
C	-103.274	-207.781	171.534
H	-344.194	-201.889	101.402
H	-543.660	-109.802	-0.07816
H	-536.093	115.243	-114.032
H	-324.263	243.487	-111.724
H	-106.319	281.619	0.09853
H	128.470	325.412	-0.31978
H	373.013	0.12585	0.83120
H	225.144	0.33359	177.566
H	182.655	-214.173	-240.238
H	263.544	-348.185	-153.052
H	0.99119	-293.432	-108.671
H	-0.02209	-221.588	209.329
H	-174.745	-218.190	253.638
H	-124.223	-282.625	0.94637

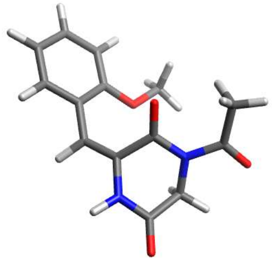

***Z-p*-NMe$_2$-Ph-DKP-Ac (11)**

C	318.890	0.91175	-0.22947
C	413.052	-0.12524	-0.00407
C	361.983	-144.212	0.12525
C	226.301	-168.402	0.06240
C	131.733	-0.65186	-0.11444
C	183.348	0.65251	-0.27468
C	-0.08138	-100.835	-0.16758
C	-119.265	-0.24672	-0.01468
N	-116.366	110.687	0.37071
C	-218.638	198.383	0.19440
C	-337.714	139.103	-0.52742
N	-360.969	-0.01445	-0.15858
C	-251.768	-0.89774	-0.15343
O	-264.696	-210.636	-0.25681
O	-214.425	314.867	0.55637
C	-495.417	-0.40402	0.01178
C	-525.908	-179.424	0.50376
O	-583.784	0.40416	-0.20547
N	546.882	0.13226	0.07321
C	641.818	-0.96121	0.22724
C	597.128	148.152	-0.14600
H	352.571	192.689	-0.38056

H	429.219	-227.544	0.26780
H	190.924	-270.471	0.15963
H	117.181	148.038	-0.49221
H	-0.29439	-206.031	-0.31962
H	-0.35448	144.977	0.87210
H	-426.236	196.014	-0.26832
H	-321.975	147.835	-160.797
H	-460.624	-209.100	132.363
H	-630.068	-179.960	0.82008
H	-511.121	-251.607	-0.29948
H	621.586	-153.652	113.560
H	639.322	-164.791	-0.62693
H	742.138	-0.54972	0.30578
H	575.838	183.742	-116.080
H	553.230	218.825	0.56446
H	704.882	148.490	-0.00168

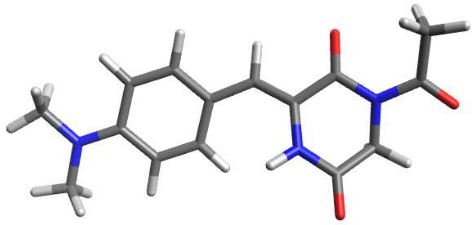

***E*-*p*-NMe$_2$-Ph-DKP-Ac (11)**

C	295.169	0.96995	0.07510
C	401.740	0.03310	0.05455
C	366.697	-133.410	-0.08527
C	234.633	-171.425	-0.18600
C	127.550	-0.78751	-0.15910
C	163.444	0.57319	-0.02937

C	-0.04832	-135.565	-0.24752
C	-134.306	-0.92246	-0.22652
N	-234.855	-191.682	-0.40809
C	-359.603	-186.023	0.11690
C	-386.827	-0.56672	0.85357
N	-320.392	0.57116	0.20387
C	-183.553	0.46217	-0.10792
O	-113.640	144.876	-0.27464
O	-441.681	-276.100	0.02349
C	-396.781	174.534	0.04719
C	-339.916	289.686	-0.74034
O	-508.926	179.190	0.51871
N	531.772	0.42899	0.15965
C	639.224	-0.55223	0.09415
C	564.947	184.185	0.28535
H	316.418	202.550	0.16629
H	443.320	-209.508	-0.11295
H	212.075	-277.065	-0.28609
H	0.85891	132.069	-0.02517
H	0.00360	-243.860	-0.34257
H	-209.230	-277.621	-0.87667
H	-493.532	-0.37758	0.86295
H	-352.603	-0.66616	188.939
H	-289.540	256.686	-164.777
H	-422.984	355.833	-0.98043
H	-266.216	343.193	-0.14144
H	639.189	-108.785	-0.86140
H	734.579	-0.04052	0.19695

H	631.167	-128.873	0.90017
H	518.463	228.382	117.226
H	672.735	194.478	0.38143
H	532.687	241.313	-0.59201

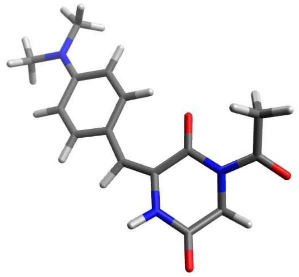

***Z*-*p*-NPh$_2$-Ph-DKP-Ac (12)**

C	-330.268	-0.34333	0.02277
N	-323.581	105.856	0.06087
C	-424.467	189.458	-0.30411
C	-547.215	118.442	-0.83025
N	-572.164	-0.09717	-0.15074
C	-465.071	-0.97516	0.05833
O	-480.035	-216.777	0.25823
O	-416.043	310.980	-0.24710
C	-221.781	-115.101	0.05920
C	-0.80752	-0.81622	0.02329
C	0.11579	-175.717	0.52107
C	147.615	-151.142	0.53104
C	198.780	-0.31051	0.00858
C	108.135	0.61926	-0.53135
C	-0.27971	0.37486	-0.51657
C	385.133	128.376	0.08834
N	336.303	-0.05051	0.01192
C	430.609	-111.417	-0.05772
C	415.923	-213.063	-100.696
C	508.873	-316.371	-107.013

C	618.132	-318.420	-0.20369
C	633.361	-216.344	0.73385
C	539.882	-113.608	0.81425
C	487.818	170.102	-0.76414
C	537.217	299.878	-0.67690
C	483.958	389.610	0.24790
C	381.253	348.035	109.480
C	332.518	217.935	102.517
O	-794.174	0.33433	-0.31028
C	-707.606	-0.41644	0.09686
C	-740.757	-164.741	0.89733
H	-239.773	149.459	0.42431
H	-633.483	182.255	-0.67738
H	-535.505	102.586	-190.776
H	-245.555	-220.189	0.17334
H	-0.25400	-269.064	0.92990
H	215.278	-224.718	0.94403
H	145.531	153.182	-0.97550
H	-0.93081	109.704	-0.99054
H	331.868	-210.606	-168.905
H	496.592	-394.682	-180.902
H	690.742	-398.607	-0.26019
H	717.724	-217.141	141.396
H	551.061	-0.34686	154.712
H	528.468	100.553	-148.763
H	616.822	331.209	-134.213
H	522.198	490.771	0.30966
H	339.785	416.645	182.395
H	253.825	185.197	169.287
H	-845.070	-156.157	119.655
H	-726.886	-253.972	0.28679
H	-676.416	-175.409	176.975

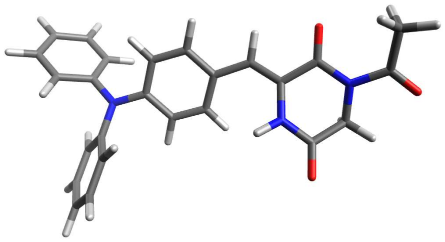

***E*-*p*-NPh$_2$-Ph-DKP-Ac (12)**

C	344.645	-0.84883	-0.54797
N	450.989	-165.887	-103.529
C	573.672	-174.580	-0.46610
C	591.324	-0.79554	0.70240
N	522.414	0.47906	0.45803
C	385.779	0.42129	0.09595
O	309.819	134.495	0.30872
O	661.054	-251.223	-0.83932
C	218.298	-131.015	-0.76727
C	0.82118	-0.86449	-0.54905
C	-0.18547	-181.151	-0.85313
C	-152.917	-154.180	-0.68895
C	-194.800	-0.28880	-0.20666
C	-0.96092	0.66738	0.09215
C	0.38334	0.39104	-0.07636
C	-378.829	133.394	-0.15240
N	-330.529	-0.00039	-0.03620
C	-424.137	-103.279	0.25337
C	-397.079	-197.628	124.965
C	-489.558	-297.682	153.032
C	-610.406	-303.607	0.83678
C	-637.806	-208.748	-0.14766

C	-545.077	-109.388	-0.44551
C	-466.115	184.581	0.81219
C	-515.267	314.163	0.68902
C	-476.906	394.231	-0.38605
C	-389.525	343.188	-134.566
C	-341.284	213.158	-123.790
O	540.864	275.607	0.23903
C	587.816	171.737	0.64710
C	720.765	167.706	136.978
H	431.646	-230.109	-179.304
H	553.037	-127.265	161.094
H	697.587	-0.63487	0.82546
H	219.460	-229.704	-122.521
H	0.10494	-278.299	-123.750
H	-226.415	-229.376	-0.94198
H	-125.854	163.869	0.46412
H	111.346	114.572	0.16096
H	-303.907	-192.039	179.834
H	-467.671	-370.317	230.418
H	-682.540	-381.190	106.301
H	-731.269	-212.600	-0.69477
H	-565.815	-0.36090	-121.503
H	-495.116	122.469	165.040
H	-582.872	352.960	144.186
H	-514.864	495.284	-0.47654
H	-359.825	404.246	-219.023
H	-274.567	172.927	-198.978
H	798.816	125.767	0.72946

H	747.560	270.361	160.867
H	716.322	108.502	228.529

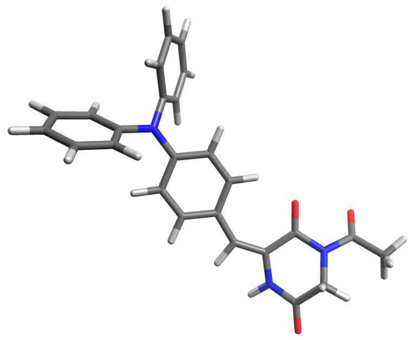

***Z-p*-CN-Ph-DKP-Ac (13)**

C	-367.659	0.89600	0.43456
C	-458.178	-0.08957	0.01634
C	-411.610	-136.868	-0.32247
C	-276.191	-164.543	-0.25301
C	-183.463	-0.65930	0.13166
C	-232.201	0.61168	0.48985
C	-0.42385	-102.424	0.18356
C	0.65820	-0.23603	0.01865
N	0.60266	110.778	-0.35418
C	163.283	199.487	-0.23385
C	287.713	143.055	0.41075
N	309.200	0.00342	0.12073
C	201.150	-0.87582	0.13533
O	212.908	-208.262	0.22532
O	154.435	315.910	-0.57631
C	444.866	-0.40062	0.00989
C	476.741	-181.036	-0.40580
O	531.755	0.42127	0.21893

C	-597.664	0.20427	-0.05030
N	-710.618	0.44247	-0.10511
H	-403.912	187.433	0.72144
H	-481.608	-213.354	-0.63210
H	-240.598	-263.607	-0.50790
H	-164.771	137.350	0.85644
H	-0.20092	-207.406	0.32623
H	-0.24853	146.648	-0.76917
H	373.407	198.430	0.04418
H	281.271	159.153	149.212
H	415.546	-213.587	-124.614
H	582.388	-183.402	-0.66661
H	457.173	-249.818	0.41697

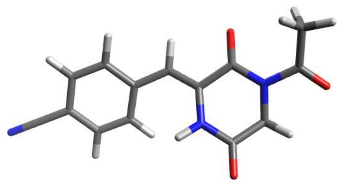

E*-*p-**CN-Ph-DKP-Ac (13)**

C	338.361	0.78489	-0.73559
C	432.383	0.13072	0.07348
C	396.562	-104.309	0.75124
C	268.119	-154.265	0.61777
C	171.422	-0.87552	-0.15673
C	209.545	0.28702	-0.84558
C	0.39902	-151.216	-0.26725
C	-0.86239	-102.671	-0.29426
N	-191.754	-192.631	-0.53446

C	-319.391	-177.517	-0.08443
C	-343.697	-0.49210	0.67409
N	-259.395	0.61966	0.21064
C	-124.315	0.40257	-0.07060
O	-0.43080	130.403	-0.12981
O	-406.024	-261.612	-0.24369
C	-320.268	190.193	0.23294
C	-247.612	307.811	-0.36055
O	-432.030	200.624	0.69639
C	564.734	0.65055	0.19427
N	671.904	107.225	0.29229
H	366.858	167.783	-127.660
H	469.269	-155.720	136.597
H	241.251	-245.861	113.005
H	138.049	0.79700	-147.009
H	0.45617	-259.743	-0.30698
H	-170.129	-280.993	-0.97900
H	-447.541	-0.20615	0.55069
H	-326.593	-0.68119	173.934
H	-201.072	283.289	-131.442
H	-320.868	387.384	-0.48329
H	-168.104	340.612	0.30913

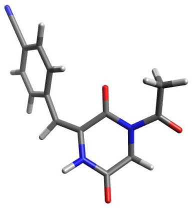

Molecular Orbitals

The orbital energies and the 3D representations (left: HOMO, right: LUMO) of the electron probability distributions were extracted with Avogadro 1.2.0.

*Supplementary Table 7. HOMO-1 (H-1), HOMO (H), LUMO (L) and LUMO+1 Kohn-Sham orbital energies (in eV) of the E-isomers and Z-isomers of the compounds **7-13** calculated on the TD-B3LYP-GD3BJ/6-311G(d,p)level of theory.*

**Compound**		ε_{H-1}	ε_{H}	ε_{L}	ε_{L+1}
**7**	*Z*	-7,218	-6,335	-2,281	-0,803
	E	-7,207	-6,287	-2,331	-0,829
**8**	*Z*	-7,318	-5,959	-2,197	-0,707
	E	-7,288	-5,896	-2,269	-0,691
**9**	*Z*	-6,709	-6,213	-2,296	-0,786
	E	-6,628	-6,159	-2,338	-0,806
**10**	*Z*	-6,848	-6,134	-2,192	-0,714
	E	-6,703	-6,085	-1,962	-0,752
**11**	*Z*	-6,844	-5,358	-2,088	-0,607
	E	-6,838	-5,327	-2,153	-0,582
**12**	*Z*	-6,573	-5,372	-2,252	-0,970
	E	-6,543	-5,356	-2,312	-0,911
**13**	*Z*	-7,604	-6,531	-2,669	-1,263
	E	-7,539	-6,545	-2,597	-1,314

Z-Ph-DKP-Ac (7)

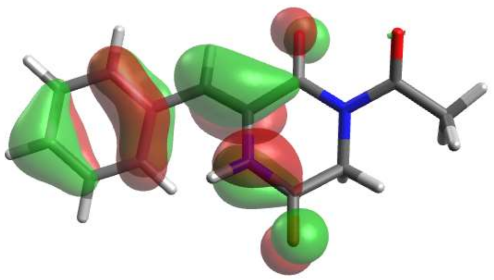

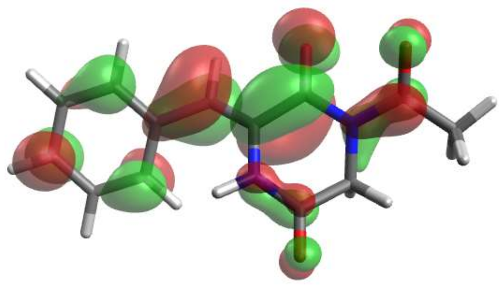

E-Ph-DKP-Ac (7)

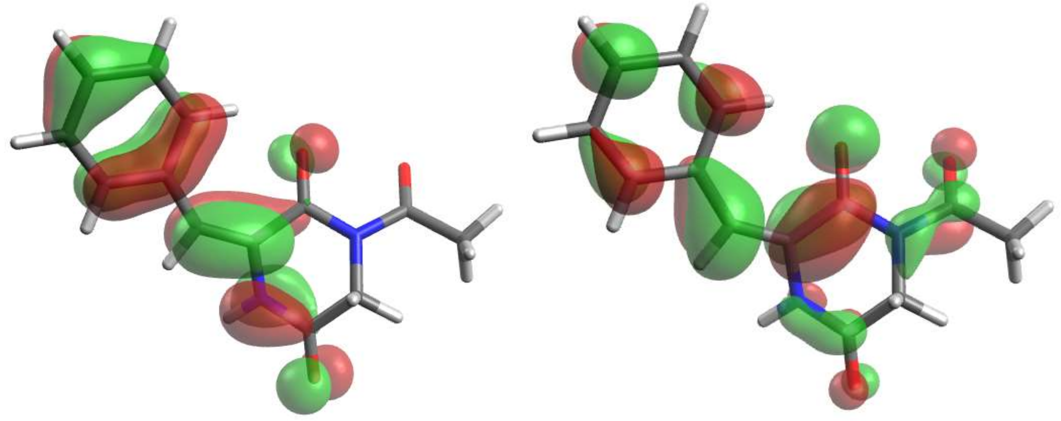

Z-p-OMe-Ph-DKP-Ac (8)

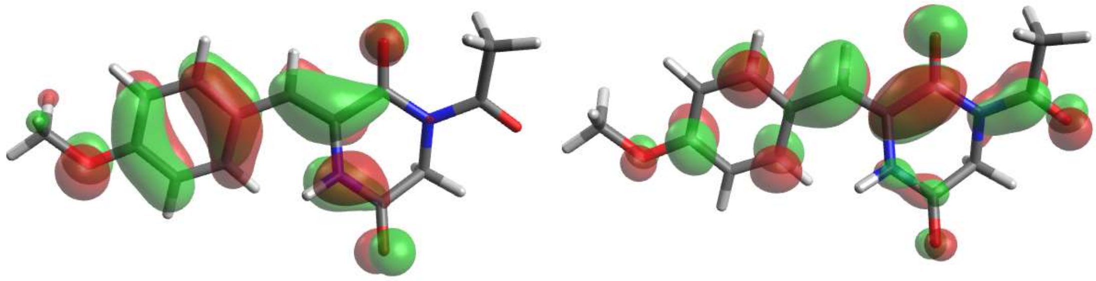

E-p-OMe-Ph-DKP-Ac (8)

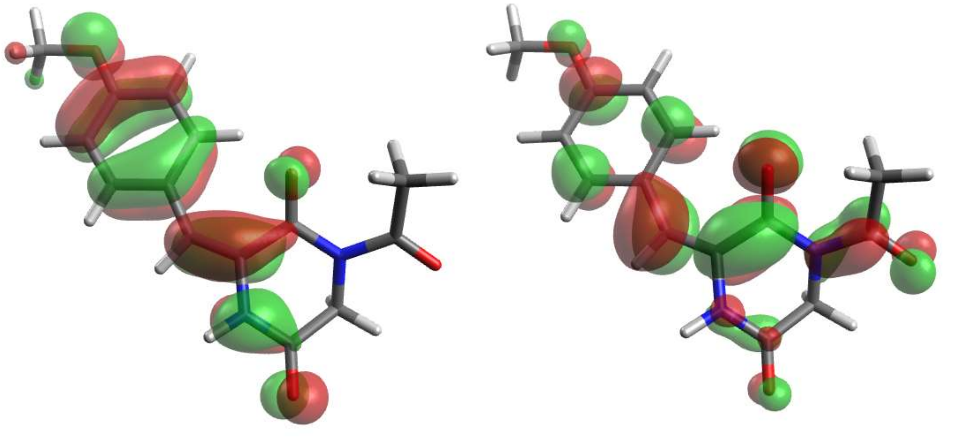

Z-m-OMe-Ph-DKP-Ac (9)

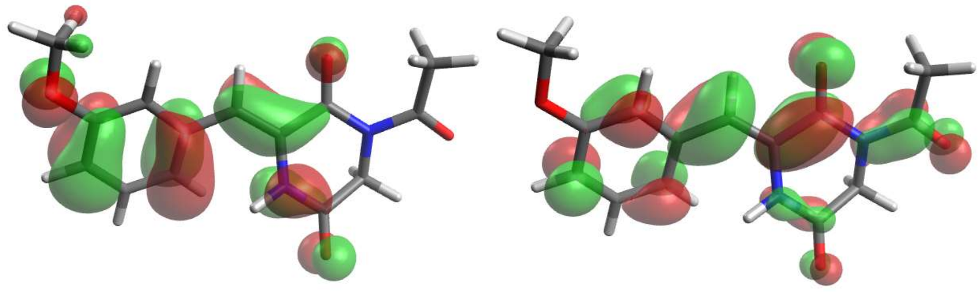

E-m-OMe-Ph-DKP-Ac (9)

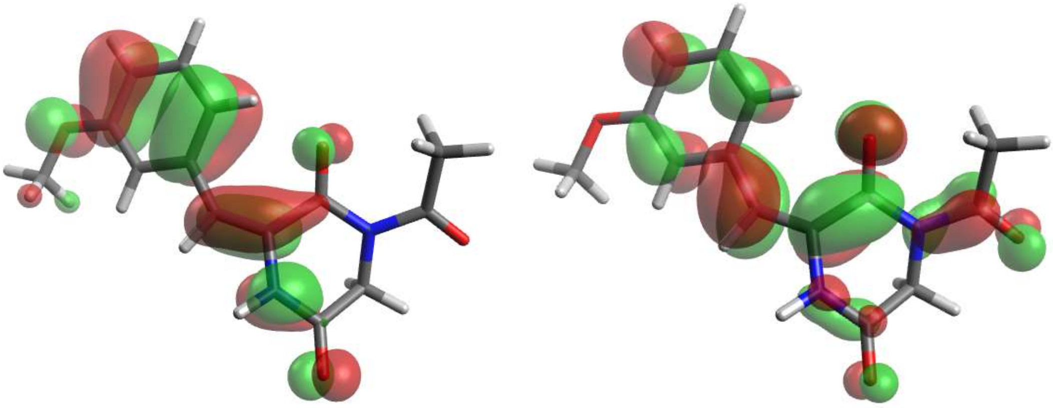

Z-o-OMe-Ph-DKP-Ac (10)

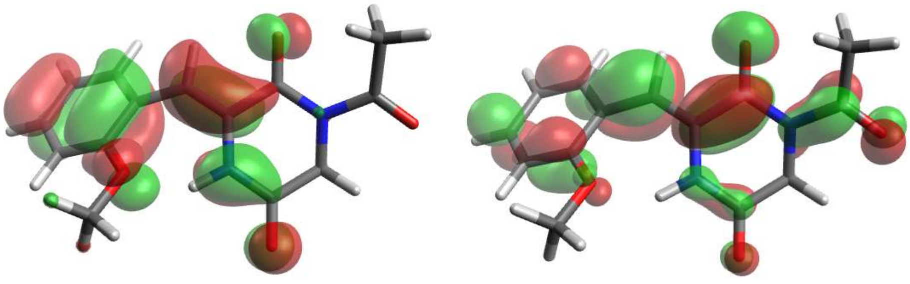

E-o-OMe-Ph-DKP-Ac (10)

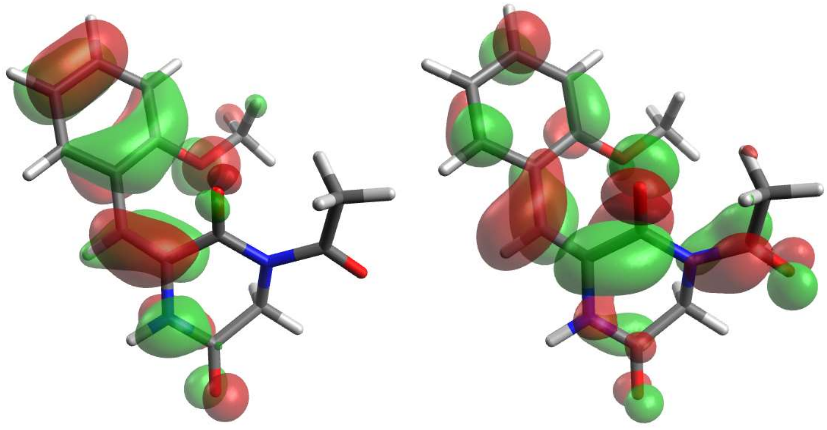

Z-p-NMe₂-Ph-DKP-Ac (11)

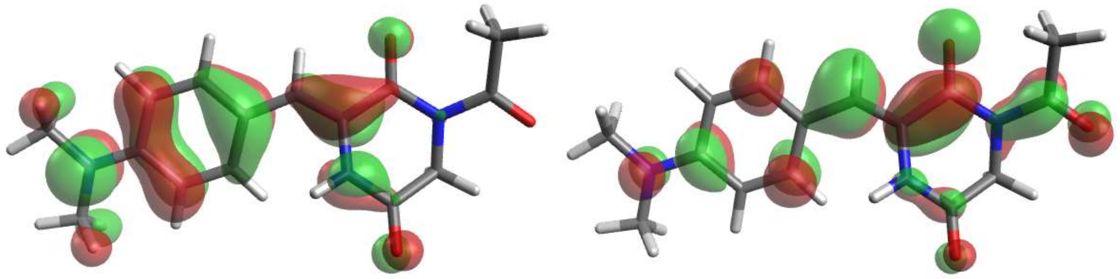

E-p-NMe₂-Ph-DKP-Ac (11)

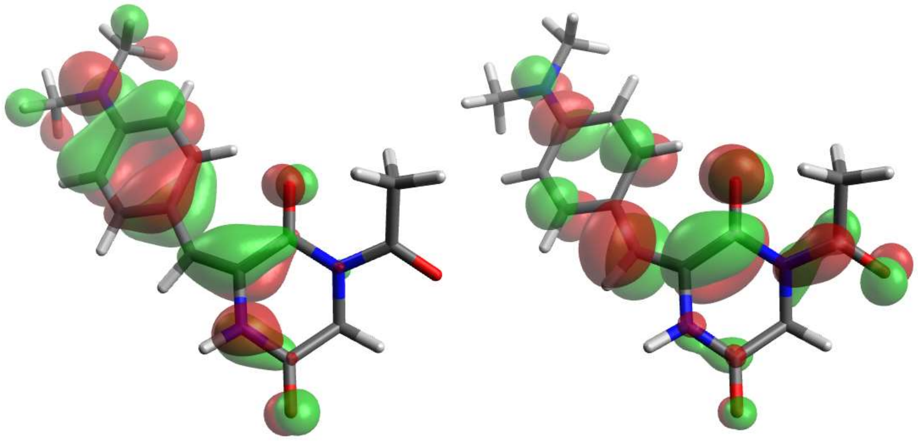

Z-p-NPh₂-Ph-DKP-Ac (12)

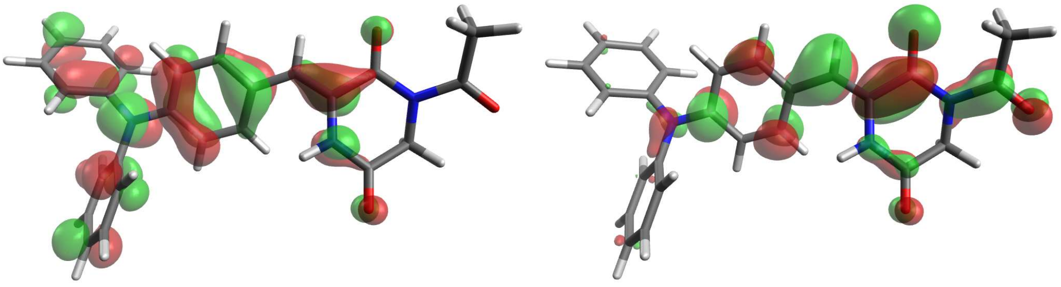

E-p-NPh$_2$-Ph-DKP-Ac (12)

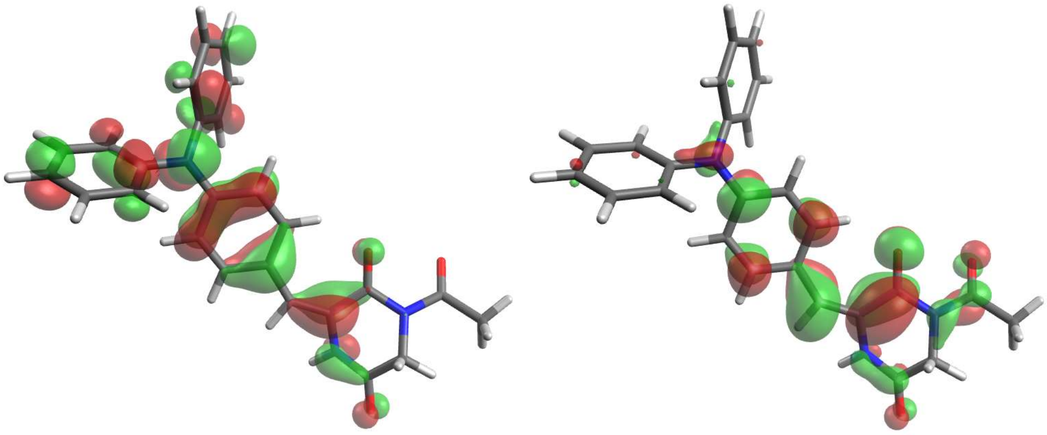

Z-p-CN-Ph-DKP-Ac (13)

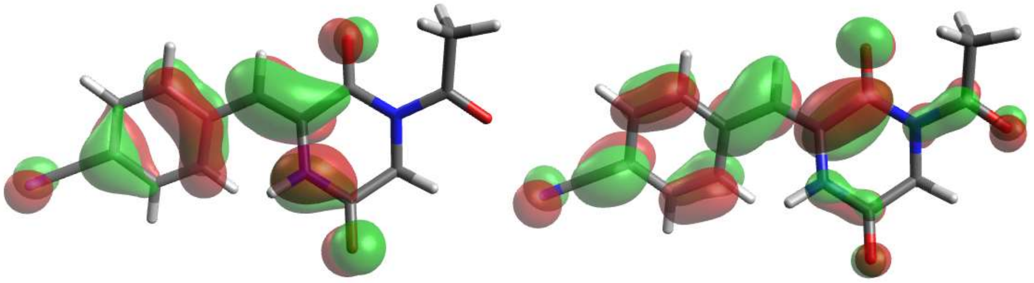

E-p-CN-Ph-DKP-Ac (13)

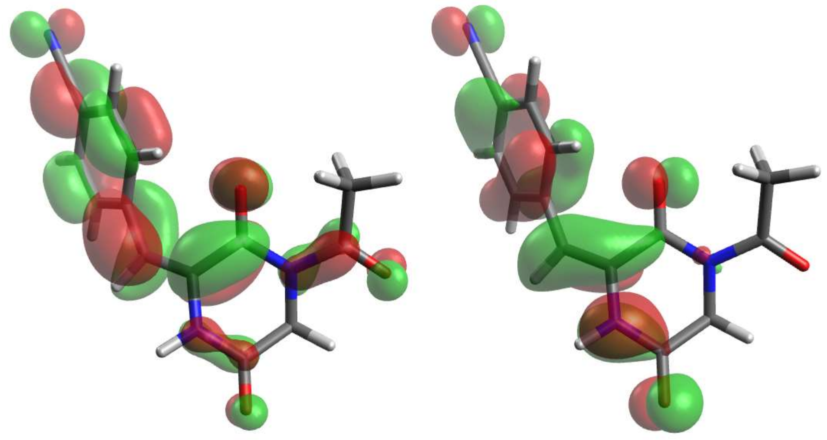

Excitation Energies and Calculated Absorption Spectra

Excitation Energies

Excitation energies were then calculated on TD-B3LYP-GD3BJ/6-311G(d,p) level of theory, using previously obtained structures. Solvent interactions were simulated using a polarizable continuum model (PCM), with parameters taken for DMSO. The five lowest transitions computed in the TD calculations (10 states in total) are reported. For each excited state the energy (in eV), the associated wavelength (in nm) and the oscillator strength of the transition is given. The numbers of the orbitals involved in the transition (with general formula occupied → unoccupied) and the associated (largest) coefficient in the CI expansion is presented.

***Z*-Ph-DKP-Ac (7)**

Excited State 1: 3.6659 eV 338.21 nm f=0.5614

63 -> 65   0.13464

64 -> 65   0.68835

Excited State   2: 3.8710 eV 320.29 nm f=0.0553

61 -> 65   0.16695

62 -> 65   -0.12144

63 -> 65   0.62555

63 -> 66   0.14669

64 -> 65   -0.14257

Excited State 3: 4.3807 eV 283.02 nm f=0.0048

61 -> 65 -0.17565

62 -> 65 0.60897

63 -> 65 0.17388

64 -> 67 -0.23825

Excited State 4: 4.4377 eV 279.39 nm f=0.0353

61 -> 65 0.63143

62 -> 65 0.19905

63 -> 65 -0.15136

Excited State 5: 4.8883 eV 253.64 nm f=0.0500

58 -> 65 0.34338

58 -> 66 0.10534

59 -> 65 0.22563

60 -> 65 0.28207

63 -> 66 -0.23256

63 -> 69 -0.11949

64 -> 66 0.37336

***E*-Ph-DKP-Ac (7)**

Excited State 1: 3.5756 eV 346.75 nm f=0.5345

63 -> 65 0.18410

64 -> 65 0.67042

Excited State 2: 3.8216 eV 324.43 nm f=0.0931

61 -> 65 0.15493

62 -> 65 0.19230

63 -> 65 0.59700

63 -> 66 0.14135

64 -> 65 -0.20573

Excited State 3: 4.2219 eV 293.67 nm f=0.0095

62 -> 65 0.64429

63 -> 65 -0.20771

64 -> 67 0.18412

Excited State 4: 4.3346 eV 286.03 nm f=0.0439

61 -> 65 0.66557

63 -> 65 -0.16952

Excited State 5: 4.8421 eV 256.05 nm f=0.1842

58 -> 65 0.19942

59 -> 65 -0.29870

63 -> 66 -0.13519

64 -> 66 0.56144

***Z-p*-OMe-Ph-DKP-Ac (8)**

Excited State 1: 3.4188 eV 362.66 nm f=0.7882

72 -> 73 0.70342

Excited State 2: 4.2986 eV 288.43 nm f=0.0233

67 -> 73 -0.32573

68 -> 73 -0.32261

71 -> 73 0.49482

Excited State 3: 4.3997 eV 281.80 nm f=0.0131

69 -> 73 0.24852

70 -> 73 -0.43173

72 -> 74 0.44380

72 -> 75 -0.18940

Excited State 4: 4.5054 eV 275.19 nm f=0.0025

68 -> 73 -0.14421

69 -> 73 0.46407

70 -> 73 0.42017

71 -> 73 -0.21480

72 -> 74 0.12723

Excited State 5: 4.7342 eV 261.89 nm f=0.1474

68 -> 73 0.10544

72 -> 74 0.21583

72 -> 75 0.64538

***E-p*-OMe-Ph-DKP-Ac (8)**

Excited State 1: 3.2811 eV 377.87 nm f=0.7129

72 -> 73 0.70320

Excited State 2: 4.1795 eV 296.65 nm f=0.0019

67 -> 73 -0.30644

68 -> 73 0.28669

69 -> 73 -0.15157

70 -> 73 0.31176

71 -> 73 0.42080

72 -> 75 0.10506

Excited State 3: 4.3188 eV 287.08 nm f=0.0032

67 -> 73 0.12218

68 -> 73 -0.13517

70 -> 73 0.53737

71 -> 73 -0.29196

72 -> 75 0.28718

Excited State 4: 4.4033 eV 281.57 nm f=0.0107

67 -> 73 0.13740

68 -> 73 -0.26550

69 -> 73 0.47813

71 -> 73 0.37451

72 -> 75 0.10067

Excited State 5: 4.6794 eV 264.96 nm f=0.2732

67 -> 73 -0.17381

69 -> 73 0.13680

72 -> 74 0.64568

72 -> 75 -0.10003

***Z-m*-OMe-Ph-DKP-Ac (9)**

Excited State 1: 3.4943 eV 354.82 nm f=0.3787

71 -> 73 0.11538

72 -> 73 0.69350

Excited State 2: 3.8829 eV 319.31 nm f=0.2400

71 -> 73 0.68326

72 -> 73 -0.11481

Excited State 3: 4.3248 eV 286.68 nm f=0.0468

66 -> 73 0.11215

67 -> 73 0.34337

68 -> 73 0.12955

69 -> 73 0.55509

70 -> 73 0.12009

Excited State 4: 4.4718 eV 277.26 nm f=0.0166

69 -> 73 -0.13567

70 -> 73 0.67050

Excited State 5: 4.7584 eV 260.56 nm f=0.0012

67 -> 73 -0.36847

68 -> 73 0.53035

68 -> 74 0.13439

70 -> 73 0.10096

70 -> 74 -0.11132

***E-m*-OMe-Ph-DKP-Ac (9)**

Excited State 1: 3.3839 eV 366.40 nm f=0.3224

71 -> 73 0.15348

72 -> 73 0.68376

Excited State 2: 3.7655 eV 329.27 nm f=0.2875

71 -> 73 0.67619

72 -> 73 -0.14705

Excited State 3: 4.2930 eV 288.80 nm f=0.0939

67 -> 73 -0.33967

69 -> 73 0.42826

70 -> 73 0.41285

Excited State 4: 4.3876 eV 282.58 nm f=0.0024

67 -> 73 0.16081

69 -> 73 -0.37488

70 -> 73 0.55112

Excited State 5: 4.7268 eV 262.30 nm f=0.0179

67 -> 73 0.47210

67 -> 74 0.10954

68 -> 73 0.33188

68 -> 74 0.10713

69 -> 73 0.26210

69 -> 74 0.12074

72 -> 74 -0.18479

***Z-o*-OMe-Ph-DKP-Ac (10)**

Excited State 1: 3.4915 eV 355.11 nm f=0.4938

72 -> 73 0.69329

Excited State 2: 4.0665 eV 304.89 nm f=0.0870

71 -> 73 0.67839

72 -> 75 -0.10584

Excited State 3: 4.4285 eV 279.97 nm f=0.0809

66 -> 73 0.13494

67 -> 73 0.37524

69 -> 73 0.53208

69 -> 74 0.11567

70 -> 73 0.12988

Excited State 4: 4.4625 eV 277.83 nm f=0.0080

69 -> 73 -0.13758

70 -> 73 0.67490

Excited State 5: 4.7785 eV 259.46 nm f=0.0254

66 -> 73 0.10871

67 -> 73 0.47992

67 -> 74 0.10644

68 -> 73 -0.12994

69 -> 73 -0.34232

69 -> 74 -0.13846

72 -> 74 0.23275

***E-o*-OMe-Ph-DKP-Ac (10)**

Excited State 1: 3.4567 eV 358.68 nm f=0.1699

71 -> 73 -0.11610

72 -> 73 0.69294

Excited State 2: 4.1009 eV 302.33 nm f=0.1563

67 -> 73 -0.10843

70 -> 73 0.10628

71 -> 73 0.65908

72 -> 73 0.11095

Excited State 3: 4.3919 eV 282.30 nm f=0.0066

67 -> 73 -0.22448

69 -> 73 -0.29037

70 -> 73 0.53223

71 -> 73 -0.15027

72 -> 74 -0.11635

72 -> 75 -0.10587

Excited State 4: 4.6849 eV 264.65 nm f=0.0445

69 -> 73 0.49962

70 -> 73 0.23901

72 -> 74 -0.39127

Excited State 5: 4.7441 eV 261.34 nm f=0.2603

67 -> 73 -0.10228

69 -> 73 0.32182

70 -> 73 0.23959

72 -> 74 0.54917

***Z*-*p*-NMe$_2$-Ph-DKP-Ac (11)**

Excited State 1: 3.0013 eV 413.11 nm f=0.9372

76 -> 77 0.70538

Excited State 2: 4.1325 eV 300.02 nm f=0.0165

74 -> 77 -0.23561

75 -> 77 -0.10829

76 -> 78 -0.31753

76 -> 79 0.56259

Excited State 3: 4.2123 eV 294.34 nm f=0.0115

71 -> 77 -0.21055

72 -> 77 -0.29105

75 -> 77 0.57360

Excited State 4: 4.3014 eV 288.24 nm f=0.1362

76 -> 78 0.61727

76 -> 79 0.31658

Excited State 5: 4.5330 eV 273.51 nm f=0.0023

72 -> 77 -0.26501

73 -> 77 0.51935

74 -> 77 -0.33592

75 -> 77 -0.12175

***E-p*-NMe$_2$-Ph-DKP-Ac (11)**

Excited State 1: 2.9135 eV 425.55 nm f=0.8714

76 -> 77 0.70512

Excited State 2: 4.1381 eV 299.62 nm f=0.0051

71 -> 77 0.20759

72 -> 77 -0.26265

73 -> 77 0.13476

74 -> 77 0.21015

75 -> 77 0.51400

76 -> 78 0.14576

76 -> 79 -0.16012

Excited State 3: 4.1915 eV 295.80 nm f=0.0291

71 -> 77 0.10028

72 -> 77 -0.13473

73 -> 77 0.13454

74 -> 77 -0.36792

75 -> 77 0.20765

76 -> 78 -0.25145

76 -> 79 0.44811

Excited State 4: 4.2953 eV 288.65 nm f=0.2551

72 -> 77 0.12387

74 -> 77 -0.20697

76 -> 78 0.61586

76 -> 79 0.19529

Excited State 5: 4.4170 eV 280.70 nm f=0.0101

71 -> 77 -0.23912

72 -> 77 0.39560

73 -> 77 -0.19842

74 -> 77 -0.12830

75 -> 77 0.40340

76 -> 78 -0.14252

76 -> 79 -0.13923

***Z-p*-NPh$_2$-Ph-DKP-Ac (12)**

Excited State 1: 2.7577 eV 449.60 nm f=0.8975

108 ->109 0.70398

Excited State 2: 3.7043 eV 334.70 nm f=0.0178

108 ->110 0.69127

Excited State 3: 3.9060 eV 317.42 nm f=0.1705

107 ->109 0.67985

Excited State 4: 3.9695 eV 312.34 nm f=0.1889

108 ->112 0.69138

Excited State 5: 4.0934 eV 302.89 nm f=0.0741

108 ->111 0.68039

***E-p*-NPh$_2$-Ph-DKP-Ac (12)**

Excited State 1: 2.6959 eV 459.89 nm f=0.8950

108 ->109 0.70404

Excited State 2: 3.7501 eV 330.62 nm f=0.0432

103 ->109 0.19029

104 ->109 -0.35147

105 ->109 -0.12455

106 ->109 -0.31698

107 ->109 0.41356

108 ->110 -0.11484

Excited State 3: 3.7564 eV 330.06 nm f=0.0242

104 ->109 -0.12971

108 ->110 0.67063

Excited State 4: 3.8920 eV 318.56 nm f=0.1522

103 ->109 -0.10679

104 ->109 0.31339

106 ->109 0.22729

107 ->109 0.54829

Excited State 5: 3.9657 eV 312.64 nm f=0.1728

108 ->112 0.68797

***Z*-*p*-CN-Ph-DKP-Ac (13)**

Excited State 1: 3.5225 eV 351.98 nm f=0.7568

70 -> 71 0.70090

Excited State 2: 4.2033 eV 294.97 nm f=0.0934

64 -> 71 0.10523

65 -> 71 0.27297

66 -> 71 -0.15079

67 -> 71 0.48409

67 -> 72 0.13969

68 -> 71 0.32219

Excited State 3: 4.2488 eV 291.81 nm f=0.0111

69 -> 71 0.68655

Excited State 4: 4.3963 eV 282.02 nm f=0.0081

67 -> 71 -0.32060

68 -> 71 0.50371

70 -> 72 -0.11198

70 -> 73 -0.34074

Excited State 5: 4.5957 eV 269.78 nm f=0.0039

65 -> 71 -0.26720

66 -> 71 0.53586

66 -> 72 0.13442

67 -> 71 0.25797

***E-p*-CN-Ph-DKP-Ac (13)**

Excited State 1: 3.4883 eV 355.42 nm f=0.5765

70 -> 71 0.68501

Excited State 2: 4.1753 eV 296.95 nm f=0.1761

64 -> 71 -0.10829

65 -> 71 0.22582

67 -> 71 -0.23517

68 -> 71 0.53756

69 -> 71 0.13260

70 -> 71 0.15266

70 -> 72	0.10768
70 -> 73	0.10297

Excited State 3: 4.2472 eV 291.92 nm f=0.0319

68 -> 71	-0.10236
69 -> 71	0.67620

Excited State 4: 4.4147 eV 280.84 nm f=0.0523

65 -> 71	-0.18713
66 -> 71	-0.12554
67 -> 71	0.47755
68 -> 71	0.35689
70 -> 72	-0.10895
70 -> 73	0.20666

Excited State 5: 4.5552 eV 272.18 nm f=0.0307

65 -> 71	0.15644
66 -> 71	0.35106
67 -> 71	0.33220
70 -> 72	0.44764

Calculated absorption spectra

The absorption spectra were simulated based on TD calculations (10 states in total) with Gaussview assuming a gaussian band shape (characterized by a standard deviation s = 0.2 eV) and the extracted data was plotted using OriginPro 2020 9.7.188 with the peaks, furnished by the calculation.

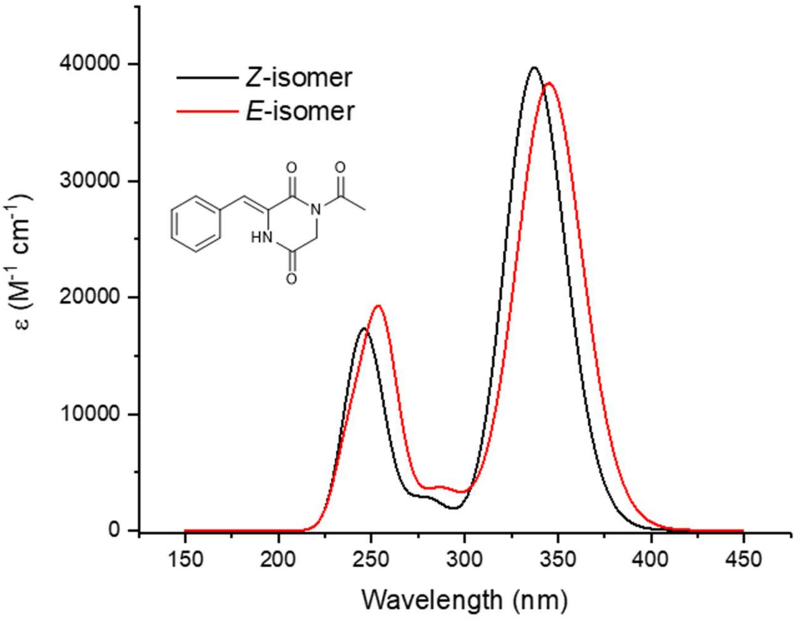

*Supplementary Figure 169: Simulated absorption spectrum of E-**7** and Z-**7** based on TD calculations (10 states in total).*

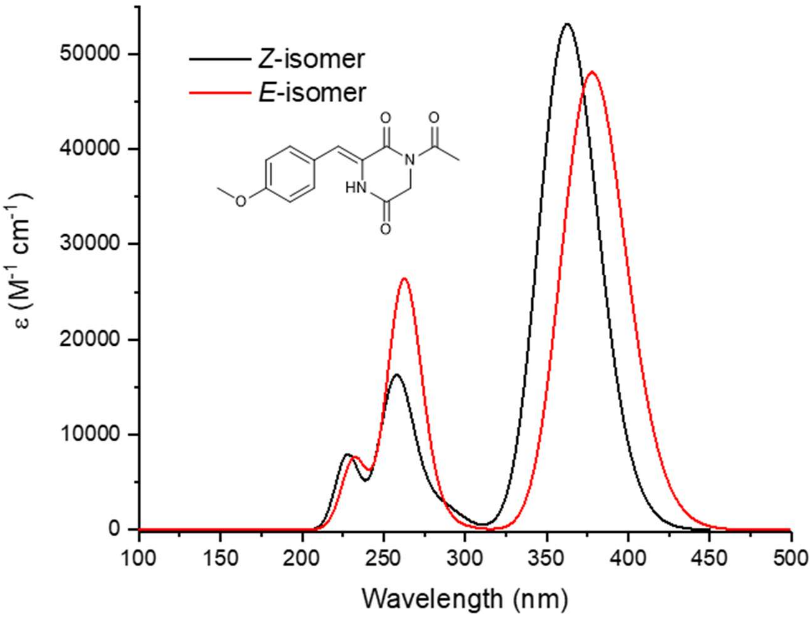

Supplementary Figure 170: *Simulated absorption spectrum of E-**8** and Z-**8** based on TD calculations (10 states in total).*

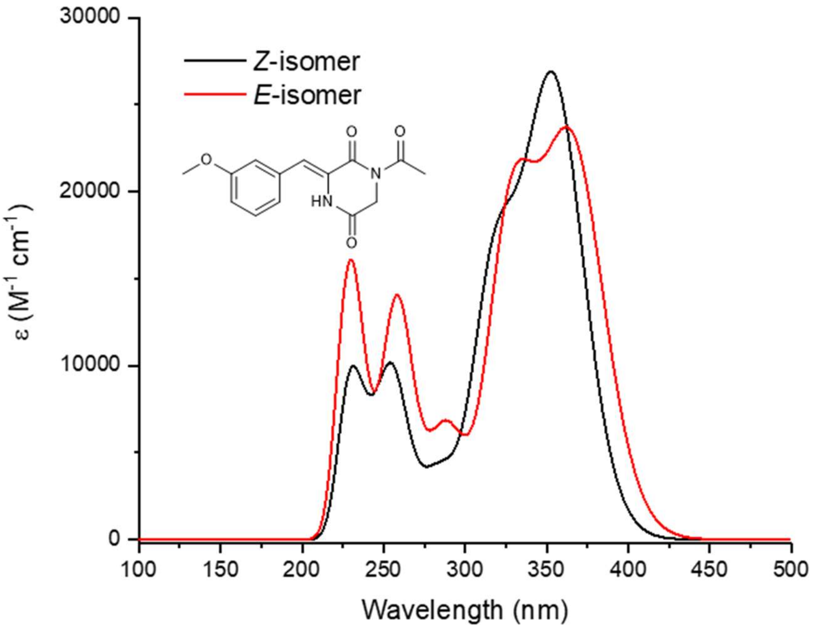

*Supplementary Figure 171: Simulated absorption spectrum of E-**9** and Z-**9** based on TD calculations (10 states in total).*

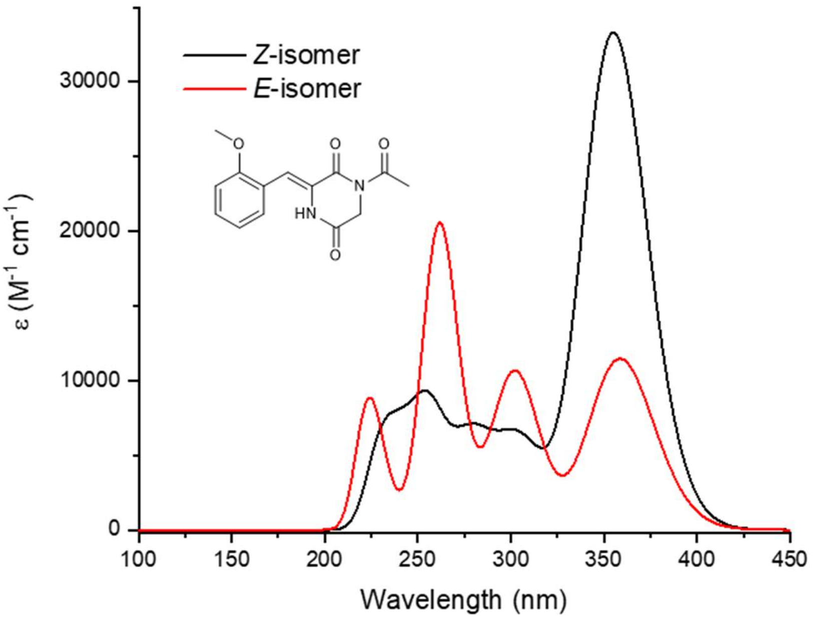

*Supplementary Figure 172: Simulated absorption spectrum of E-**10** and Z-**10** based on TD calculations (10 states in total).*

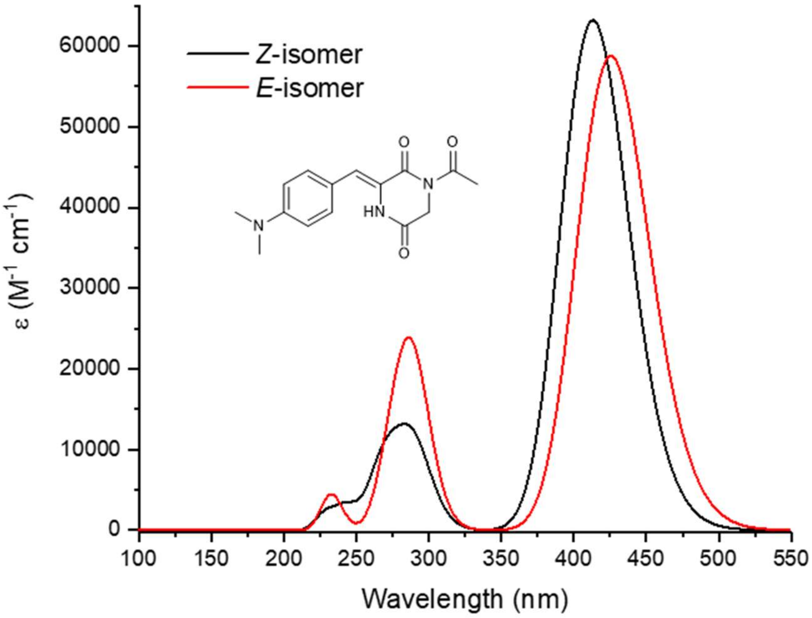

*Supplementary Figure 173: Simulated absorption spectrum of E-**11** and Z-**11** based on TD calculations (10 states in total).*

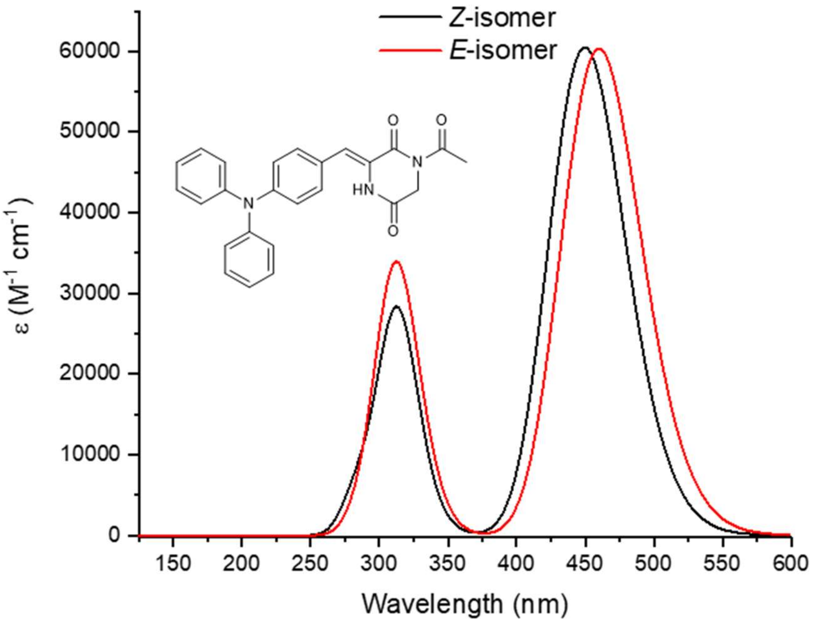

*Supplementary Figure 174: Simulated absorption spectrum of E-**12** and Z-**12** based on TD calculations (10 states in total).*

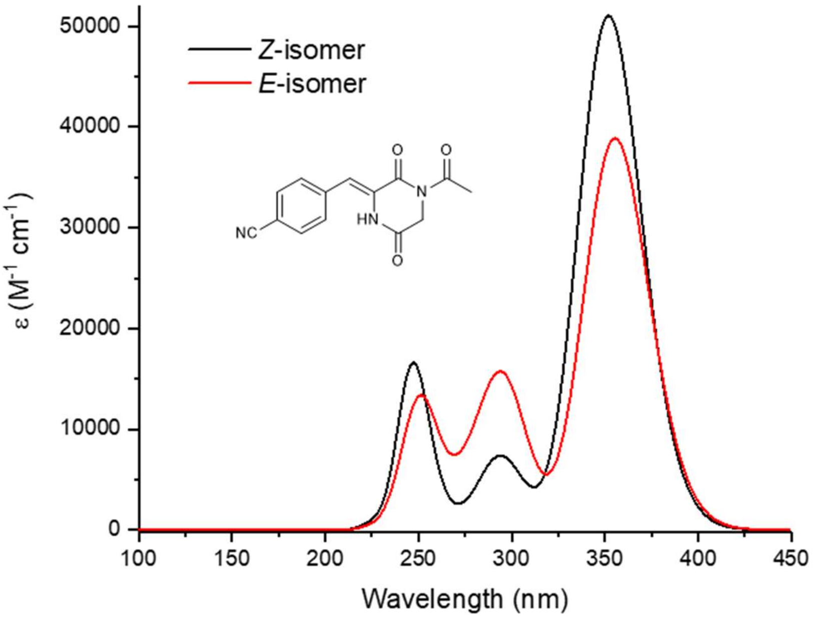

*Supplementary Figure 175: Simulated absorption spectrum of E-**13** and Z-**13** based on TD calculations (10 states in total).*

Supplementary References

1 Yamazaki, Y. *et al.* Synthesis and Structure–Activity Relationship Study of Antimicrotubule Agents Phenylahistin Derivatives with a Didehydropiperazine-2,5-dione Structure. *Journal of Medicinal Chemistry* **55**, 1056-1071, doi:10.1021/jm2009088 (2012).
