# Supplementary material for: Hemipiperazines as peptide-derived molecular photoswitches with low-nanomolar cytotoxicity
Source: Nat Commun. 2022 Oct 14;13:6066. doi: 10.1038/s41467-022-33750-7 (PMC9568564; doi:10.1038/s41467-022-33750-7)
Supplement: Supplementary file 2 — Description of Additional Supplementary Files [file 41467_2022_33750_MOESM2_ESM.pdf]

Supplementary Data 1: contains crystallographic data for the compounds 2-6 and a CheckCIF PDF file

Supplementary Data 2: contains crystallographic data for the compounds 7, 8, 11, 13, 15, and 19.
