# Supplementary material for: Hemipiperazines as peptide-derived molecular photoswitches with low-nanomolar cytotoxicity
Source: Nat Commun. 2022 Oct 14;13:6066. doi: 10.1038/s41467-022-33750-7 (PMC9568564; doi:10.1038/s41467-022-33750-7)
Supplement: Supplementary file 5 — Reporting Summary [file 41467_2022_33750_MOESM5_ESM.pdf]

nature portfolio

Corresponding author(s): Zbigniew Pianowski

Last updated by author(s): Sep 20, 2022

Reporting Summary

Nature Portfolio wishes to improve the reproducibility of the work that we publish. This form provides structure for consistency and transparency in reporting. For further information on Nature Portfolio policies, see our Editorial Policies and the Editorial Policy Checklist.

Statistics

For all statistical analyses, confirm that the following items are present in the figure legend, table legend, main text, or Methods section.

n/a	Confirmed	
☐	☒	The exact sample size (n) for each experimental group/condition, given as a discrete number and unit of measurement
☒	☐	A statement on whether measurements were taken from distinct samples or whether the same sample was measured repeatedly
☒	☐	The statistical test(s) used AND whether they are one- or two-sided<br>*Only common tests should be described solely by name; describe more complex techniques in the Methods section.*
☒	☐	A description of all covariates tested
☒	☐	A description of any assumptions or corrections, such as tests of normality and adjustment for multiple comparisons
☐	☒	A full description of the statistical parameters including central tendency (e.g. means) or other basic estimates (e.g. regression coefficient) AND variation (e.g. standard deviation) or associated estimates of uncertainty (e.g. confidence intervals)
☒	☐	For null hypothesis testing, the test statistic (e.g. F, t, r) with confidence intervals, effect sizes, degrees of freedom and P value noted<br>*Give P values as exact values whenever suitable.*
☒	☐	For Bayesian analysis, information on the choice of priors and Markov chain Monte Carlo settings
☒	☐	For hierarchical and complex designs, identification of the appropriate level for tests and full reporting of outcomes
☒	☐	Estimates of effect sizes (e.g. Cohen's d, Pearson's r), indicating how they were calculated

Our web collection on statistics for biologists contains articles on many of the points above.

Software and code

Policy information about availability of computer code

Data collection	Leica Application Suite X 3.5.7.23225 (Leica Microsystems CMS GmbH)<br>ChemStation for LC 3D systems (Agilent Technologies)<br>Chromeleon 7.2.9 (Thermo Fisher Scientific, Inc.)<br>PowerMax PC v2.1.0.0 (Coherent, Inc.)<br>FluorEssence v3.5
Data analysis	OriginPro 2019b 9.6.5.169 and 2020 9.7.188 (OriginLab Corporation), Microsoft Excel 2019 Version 1808 (Microsoft Corporation), GraphPad Prism Version 9.1.1 for Windows (GraphPad Software, Inc.), Mercury 2020.1 (CCDC), Chemdraw Professional 20.1.0.110 (PerkinElmer Informatics, Inc.), MestReNova Version 14.1.2 (Mestrelab Research S.L.)

For manuscripts utilizing custom algorithms or software that are central to the research but not yet described in published literature, software must be made available to editors and reviewers. We strongly encourage code deposition in a community repository (e.g. GitHub). See the Nature Portfolio guidelines for submitting code & software for further information.

Data

Policy information about availability of data

All manuscripts must include a data availability statement. This statement should provide the following information, where applicable:

- Accession codes, unique identifiers, or web links for publicly available datasets
- A description of any restrictions on data availability
- For clinical datasets or third party data, please ensure that the statement adheres to our policy

The data relating to the materials and methods, experimental procedures, NMR, MS and UV-Vis spectra, as well as calculations are available in the main text or in

the Supplementary Information files, including the raw data from cell viability assays (MTT assays, Source Data) and crystallographic data (Supplementary Data 1 for plinabulin derivatives 2-6, and Supplementary Data 2 for compounds 7, 8, 11, 13, 15, and 19), provided as the Supplementary Data files. The structure of plinabulin bound to beta-tubulin in alpha/beta-tubulin heterodimers is accessible in the Protein Data Bank (www.rcsb.org) under the accession code 5c8y (https://www.rcsb.org/structure/5C8Y) Crystal structures have been deposited at the Cambridge Structural Database (https://www.ccdc.cam.ac.uk/structures/) under the accession codes: 2076713 (Z-2), 2076714 (Z-3), 2076715 (E-3), 2076716 (Z-4), 2076717 (Z-5), 2076718 (Z-6), 2177720 (Z-7), 2177723 (Z-8), 2177722 (Z-11), 2177721 (Z-13), 2177725 (Z-15), and 2177724 (19). CheckCIF files are available for the crystallographic structures reported with this article. Crystal structures generated during this study are also accessible in the Chemotion repository https://www.chemotion-repository.net under the following links:
Z-2 https://dx.doi.org/10.14272/JORKNWYVOQPNQP-QOOFZUOPSA-N/CHMO0000156
Z-3 https://dx.doi.org/10.14272/LMVUWTHIXFBXBY-UOUVAZQYSA-N.1
E-3 https://dx.doi.org/10.14272/LMVUWTHIXFBXBY-GOBHWCIESA-N.2
Z-4 https://dx.doi.org/10.14272/PIABYVZMMACTIP-APGQMXJTSA-N.1
Z-5 https://dx.doi.org/10.14272/AIJQSTUPFONWLS-VULZFCBJSA-N.1
Z-6 https://dx.doi.org/10.14272/OTVHUVBFCLOWCA-KPJFGDCZSA-N.1
Z-7 https://dx.doi.org/10.14272/MJCOATFQVUUHFN-XFFZJAGNSA-N.1
Z-8 https://dx.doi.org/10.14272/DHTWSXQNUMZDAD-GHXNOFRVSA-N.1
Z-11 https://dx.doi.org/10.14272/SYFVNJGPALDWRA-JYRVWZFOSA-N.1
Z-13 https://dx.doi.org/10.14272/DDZMJSZYMNIGEY-SDQBBNPISA-N.1
Z-15 https://dx.doi.org/10.14272/HEBNVDMSJJEXNA-LCYFTJDESA-N/CHMO0000156
19 https://dx.doi.org/10.14272/XEFPBGSACIWODS-UHFFFAOYSA-N.1
Data is available from the corresponding author upon request.

Field-specific reporting

Please select the one below that is the best fit for your research. If you are not sure, read the appropriate sections before making your selection.

[x] Life sciences [ ] Behavioural & social sciences [ ] Ecological, evolutionary & environmental sciences

For a reference copy of the document with all sections, see nature.com/documents/nr-reporting-summary-flat.pdf

Life sciences study design

All studies must disclose on these points even when the disclosure is negative.

Sample size	For activity determination of the purified thermally stable isomers (compounds 1-6) by viability assays, we used at least three independent series of experiments performed on different days and by more than one researcher. Activity of mixtures (e.g. various photostationary states) was determined using single or double series of experiments and additionally corroborated with the actual ratio of photoisomers (measured with HPLC) with previously determined activities.
Data exclusions	Data points were only excluded from the analysis when errors were observed during execution of the experiment (e.g. pipetting to a wrong well). In the experiment where a sample of compound 6 was irradiated at 490 nm (Fig. S29), a complete row of wells (one concentration) was excluded. Otherwise only single wells were excluded.
Replication	All viability assays were performed with six technical replicates and the number of independent experiments is stated in the supporting information and captions of the respective figures. All attempts of replication were successful. Spectral analysis (NMR, MS, UV-Vis) was performed once, unless specified otherwise. The immunostaining visualized on the Figure 2 was perfomed once, to visualize the difference of activity between photoisomers of the compounds 1,2, and 3. The difference was previously quantified with corresponding MTT assays, and the overall inhibitory effect of plinabulin and its derivatives on microtubule dynamics has been demonstrated previously in literature.
Randomization	Randomization was not required, as no studies on more advanced organisms (like animals or humans) with distinct individual characteristics was performed.
Blinding	No blinding techniques have been used in our study. The experiments were performed using open label compounds.

Reporting for specific materials, systems and methods

We require information from authors about some types of materials, experimental systems and methods used in many studies. Here, indicate whether each material, system or method listed is relevant to your study. If you are not sure if a list item applies to your research, read the appropriate section before selecting a response.

Materials & experimental systems

n/a	Involved in the study
	[x] Antibodies
	[x] Eukaryotic cell lines
[x]	[ ] Palaeontology and archaeology
[x]	[ ] Animals and other organisms
[x]	[ ] Human research participants
[x]	[ ] Clinical data
[x]	[ ] Dual use research of concern

Methods

n/a	Involved in the study
[x]	[ ] ChIP-seq
[x]	[ ] Flow cytometry
[x]	[ ] MRI-based neuroimaging

Antibodies

Antibodies used	Anti-α-Tubulin–FITC antibody, Mouse monoclonal purified from hybridoma cell culture; Supplier: Sigma, product number F2168-2ML; clone: DM1A; batch/lot number: 028M4808V, IgG conc. 3 mg/mL, dilution for experiments 1:500
Validation	Statement from the vendor: "The product is Protein A purified Monoclonal Anti-α-Tubulin antibody conjugated to fluorescein isothiocyanate, isomer I. It is purified by gel filtration and contains no detectable free FITC."<br>The antibody was provided by the supplier with a certificate of analysis (available on the website https://www.sigmaaldrich.com/DE/en/product/sigma/f2168 upon indication of the lot number 028M4808V). It was used as such, without additional validation, before the recommended retest date.

Eukaryotic cell lines

Policy information about cell lines

Cell line source(s)	HT29 Cell Line human; Supplier: Sigma, product number 91072201-1VL.
Authentication	None of the cell lines used were authenticated
Mycoplasma contamination	the cell line was tested negative upon mycoplasma contamination with MycoStrip™ - Mycoplasma Detection Kit, Vendor: Invivogen, Cat. No: rep-mys-10
Commonly misidentified lines (See ICLAC register)	There were no misidentified lines used.
