# Supplementary material for: Hemipiperazines as peptide-derived molecular photoswitches with low-nanomolar cytotoxicity
Source: Nat Commun. 2022 Oct 14;13:6066. doi: 10.1038/s41467-022-33750-7 (PMC9568564; doi:10.1038/s41467-022-33750-7)

checkCIF/PLATON report

Structure factors have been supplied for datablock(s) sb1177_hy_CCDC-2076713, sb1192_hy_CCDC-2076714, sb1336_hy_CCDC-2076718, sb1342_hy_CCDC-2076717, sb1354_hy_CCDC-2076715, sb1373_hy_CCDC-2076716

THIS REPORT IS FOR GUIDANCE ONLY. IF USED AS PART OF A REVIEW PROCEDURE FOR PUBLICATION, IT SHOULD NOT REPLACE THE EXPERTISE OF AN EXPERIENCED CRYSTALLOGRAPHIC REFEREE.

No syntax errors found.   CIF dictionary   Interpreting this report

Datablock: sb1177_hy_CCDC-2076713

```
Bond precision:  C-C = 0.0020 A          Wavelength=1.54178

Cell:             a=7.0552(2)     b=9.6541(3)        c=26.1751(8)
                  alpha=90        beta=94.157(2)     gamma=90
Temperature:      123 K

                 Calculated                  Reported
Volume           1778.14(9)                  1778.14(9)
Space group      P 21/n                      P 21/n
Hall group       -P 2yn                      -P 2yn
Moiety formula   C19 H18 F2 N4 O2, H2 O      C19 H18 F2 N4 O2, H2 O
Sum formula      C19 H20 F2 N4 O3            C19 H20 F2 N4 O3
Mr               390.39                      390.39
Dx,g cm-3        1.458                       1.458
Z                4                           4
Mu (mm-1)        0.972                       0.972
F000             816.0                       816.0
F000'            818.88
h,k,lmax         8,11,32                     8,11,32
Nref             3509                        3502
Tmin,Tmax        0.849,0.981                 0.781,0.970
Tmin'            0.839

Correction method= # Reported T Limits: Tmin=0.781 Tmax=0.970
AbsCorr = MULTI-SCAN

Data completeness= 0.998          Theta(max)= 72.210

R(reflections)= 0.0407( 3133)     wR2(reflections)= 0.1018( 3502)

S = 1.087                  Npar= 268
```

```
The following ALERTS were generated. Each ALERT has the format
       test-name_ALERT_alert-type_alert-level.
Click on the hyperlinks for more details of the test.
```

Alert level B

```
PLAT420_ALERT_2_B D-H Bond Without Acceptor  O1W      --H1W2     .     Please Check
```

Author Response: weak OH...pi (2,6-difluorophenyl) interaction, C20 - c25 ... O1W2(i), with (i) = -x, 1-y, 1-z and H...Z(Ph) 277 pm

---

Alert level C

```
PLAT906_ALERT_3_C Large K Value in the Analysis of Variance ......      2.904 Check
PLAT911_ALERT_3_C Missing FCF Refl Between Thmin & STh/L=    0.600          6 Report
```

Alert level G

```
PLAT002_ALERT_2_G Number of Distance or Angle Restraints on AtSite          9 Note
PLAT172_ALERT_4_G The CIF-Embedded .res File Contains DFIX Records          3 Report
PLAT720_ALERT_4_G Number of Unusual/Non-Standard Labels ..........          2 Note
PLAT860_ALERT_3_G Number of Least-Squares Restraints .............          6 Note
PLAT912_ALERT_4_G Missing # of FCF Reflections Above STh/L=  0.600          1 Note
PLAT941_ALERT_3_G Average HKL Measurement Multiplicity ...........        4.4 Low
PLAT978_ALERT_2_G Number C-C Bonds with Positive Residual Density.         15 Info
```

```
   0 ALERT level A = Most likely a serious problem - resolve or explain
   1 ALERT level B = A potentially serious problem, consider carefully
   2 ALERT level C = Check. Ensure it is not caused by an omission or oversight
   7 ALERT level G = General information/check it is not something unexpected

   0 ALERT type 1 CIF construction/syntax error, inconsistent or missing data
   3 ALERT type 2 Indicator that the structure model may be wrong or deficient
   4 ALERT type 3 Indicator that the structure quality may be low
   3 ALERT type 4 Improvement, methodology, query or suggestion
   0 ALERT type 5 Informative message, check
```

Datablock: sb1192_hy_CCDC-2076714

```
Bond precision:   C-C = 0.0055 A           Wavelength=1.54178

Cell:             a=7.7969(3)      b=14.3794(6)      c=16.8804(7)
                  alpha=90         beta=90           gamma=90
Temperature:      123 K
```

	Calculated	Reported
Volume	1892.54(13)	1892.54(13)
Space group	P 21 21 21	P 21 21 21
Hall group	P 2ac 2ab	P 2ac 2ab
Moiety formula	C19 H18 Cl2 N4 O2	C19 H18 Cl2 N4 O2
Sum formula	C19 H18 Cl2 N4 O2	C19 H18 Cl2 N4 O2
Mr	405.27	405.27
Dx,g cm-3	1.422	1.422
Z	4	4
Mu (mm-1)	3.276	3.276
F000	840.0	840.0
F000'	845.05	
h,k,lmax	9,17,20	9,17,20
Nref	3742[2150]	3736
Tmin,Tmax	0.790,0.937	0.684,0.840
Tmin'	0.592	

Correction method= # Reported T Limits: Tmin=0.684 Tmax=0.840
AbsCorr = MULTI-SCAN

Data completeness= 1.74/1.00 Theta(max)= 72.153

R(reflections)= 0.0435(3264) wR2(reflections)= 0.1020(3736)

S = 1.058 Npar= 253

The following ALERTS were generated. Each ALERT has the format
test-name_ALERT_alert-type_alert-level.
Click on the hyperlinks for more details of the test.

Alert level C

```
PLAT242_ALERT_2_C Low    'MainMol' Ueq as Compared to Neighbors of        C20 Check
PLAT340_ALERT_3_C Low Bond Precision on  C-C Bonds ...............    0.00547 Ang.
PLAT480_ALERT_4_C Long H...A H-Bond Reported H17      ..CL9      .       2.99 Ang.
```

Alert level G

```
PLAT002_ALERT_2_G Number of Distance or Angle Restraints on AtSite          6 Note
PLAT172_ALERT_4_G The CIF-Embedded .res File Contains DFIX Records          1 Report
PLAT860_ALERT_3_G Number of Least-Squares Restraints .............          3 Note
PLAT978_ALERT_2_G Number C-C Bonds with Positive Residual Density.          7 Info
```

---

0 **ALERT level A** = Most likely a serious problem - resolve or explain
0 **ALERT level B** = A potentially serious problem, consider carefully
3 **ALERT level C** = Check. Ensure it is not caused by an omission or oversight
4 **ALERT level G** = General information/check it is not something unexpected

0 ALERT type 1 CIF construction/syntax error, inconsistent or missing data

3 ALERT type 2 Indicator that the structure model may be wrong or deficient
2 ALERT type 3 Indicator that the structure quality may be low
2 ALERT type 4 Improvement, methodology, query or suggestion
0 ALERT type 5 Informative message, check

Datablock: sb1354_hy_CCDC-2076715

Bond precision: C-C = 0.0034 A Wavelength=1.54178

Cell:	a=27.8992(10)	b=10.2709(4)	c=14.6321(5)
	alpha=90	beta=116.603(2)	gamma=90
Temperature:	298 K		

	Calculated	Reported
Volume	3748.9(2)	3748.9(2)
Space group	C 2/c	C 2/c
Hall group	-C 2yc	-C 2yc
Moiety formula	C19 H18 Cl2 N4 O2	C19 H18 Cl2 N4 O2
Sum formula	C19 H18 Cl2 N4 O2	C19 H18 Cl2 N4 O2
Mr	405.27	405.27
Dx,g cm-3	1.436	1.436
Z	8	8
Mu (mm-1)	3.308	3.308
F000	1680.0	1680.0
F000’	1690.11	
h,k,lmax	34,12,18	34,12,18
Nref	3717	3705
Tmin,Tmax	0.820,0.906	0.730,0.915
Tmin’	0.609	

Correction method= # Reported T Limits: Tmin=0.730 Tmax=0.915
AbsCorr = MULTI-SCAN

Data completeness= 0.997 Theta(max)= 72.382

R(reflections)= 0.0411(3057) wR2(reflections)= 0.1034(3705)

S = 1.035 Npar= 253

The following ALERTS were generated. Each ALERT has the format
test-name_ALERT_alert-type_alert-level.
Click on the hyperlinks for more details of the test.

Alert level C
PLAT906_ALERT_3_C Large K Value in the Analysis of Variance ...... 2.864 Check

---

Alert level G

```
PLAT002_ALERT_2_G Number of Distance or Angle Restraints on AtSite          6 Note
PLAT003_ALERT_2_G Number of Uiso or Uij Restrained non-H Atoms ...         27 Report
PLAT128_ALERT_4_G Alternate Setting for Input Space Group     C2/c       I2/a Note
PLAT172_ALERT_4_G The CIF-Embedded .res File Contains DFIX Records          3 Report
PLAT187_ALERT_4_G The CIF-Embedded .res File Contains RIGU Records          1 Report
PLAT860_ALERT_3_G Number of Least-Squares Restraints .............        219 Note
PLAT912_ALERT_4_G Missing # of FCF Reflections Above STh/L=  0.600         12 Note
PLAT978_ALERT_2_G Number C-C Bonds with Positive Residual Density.          8 Info
```

```
   0 ALERT level A = Most likely a serious problem - resolve or explain
   0 ALERT level B = A potentially serious problem, consider carefully
   1 ALERT level C = Check. Ensure it is not caused by an omission or oversight
   8 ALERT level G = General information/check it is not something unexpected

   0 ALERT type 1 CIF construction/syntax error, inconsistent or missing data
   3 ALERT type 2 Indicator that the structure model may be wrong or deficient
   2 ALERT type 3 Indicator that the structure quality may be low
   4 ALERT type 4 Improvement, methodology, query or suggestion
   0 ALERT type 5 Informative message, check
```

Datablock: sb1373_hy_CCDC-2076716

Bond precision: C-C = 0.0017 A Wavelength=1.54178

Cell:	a=7.2824(2)	b=14.1512(4)	c=18.4592(5)
	alpha=90	beta=94.740(1)	gamma=90
Temperature:	173 K		

	Calculated	Reported
Volume	1895.80(9)	1895.80(9)
Space group	P 21/c	P 21/c
Hall group	-P 2ybc	-P 2ybc
Moiety formula	C21 H25 N5 O2	C21 H25 N5 O2
Sum formula	C21 H25 N5 O2	C21 H25 N5 O2
Mr	379.46	379.46
Dx,g cm-3	1.329	1.329
Z	4	4
Mu (mm-1)	0.713	0.713
F000	808.0	808.0
F000’	810.35	
h,k,lmax	8,17,22	8,17,22
Nref	3726	3722
Tmin,Tmax	0.926,0.979	0.876,0.970
Tmin’	0.899	

Correction method= # Reported T Limits: Tmin=0.876 Tmax=0.970
AbsCorr = MULTI-SCAN

```
Data completeness= 0.999          Theta(max)= 72.135

R(reflections)= 0.0356( 3420)     wR2(reflections)= 0.0929( 3722)

S = 1.027                  Npar= 265
```

The following ALERTS were generated. Each ALERT has the format
test-name_ALERT_alert-type_alert-level.
Click on the hyperlinks for more details of the test.

Alert level B

```
PLAT414_ALERT_2_B Short Intra D-H..H-X       H1       ..H63             1.87 Ang.
                                                  x,y,z  =      1_555 Check
```

Author Response: due to the planarity of the molecule short intramolecular N1-H1...H63-C63 distance

---

Alert level C

```
PLAT420_ALERT_2_C D-H Bond Without Acceptor  N1       --H1       .     Please Check
```

Author Response: due to the planarity of the molecule and the short intramolecular N1-H1...H63-C63 distance no strong hydrogen bond possible

---

Alert level G

```
PLAT002_ALERT_2_G Number of Distance or Angle Restraints on AtSite          6 Note
PLAT172_ALERT_4_G The CIF-Embedded .res File Contains DFIX Records          1 Report
PLAT860_ALERT_3_G Number of Least-Squares Restraints .............          3 Note
PLAT912_ALERT_4_G Missing # of FCF Reflections Above STh/L=  0.600          3 Note
PLAT978_ALERT_2_G Number C-C Bonds with Positive Residual Density.         15 Info
```

---

0 **ALERT level A** = Most likely a serious problem - resolve or explain
1 **ALERT level B** = A potentially serious problem, consider carefully
1 **ALERT level C** = Check. Ensure it is not caused by an omission or oversight
5 **ALERT level G** = General information/check it is not something unexpected

0 ALERT type 1 CIF construction/syntax error, inconsistent or missing data
4 ALERT type 2 Indicator that the structure model may be wrong or deficient
1 ALERT type 3 Indicator that the structure quality may be low
2 ALERT type 4 Improvement, methodology, query or suggestion
0 ALERT type 5 Informative message, check

Datablock: sb1342_hy_CCDC-2076717

```
Bond precision:  C-C = 0.0020 A          Wavelength=1.54178
```

Cell:	a=11.5196(2)	b=14.6170(2)	c=11.8196(2)
	alpha=90	beta=101.496(1)	gamma=90
Temperature:	298 K		

	Calculated	Reported
Volume	1950.28(5)	1950.28(5)
Space group	P 21/c	P 21/c
Hall group	-P 2ybc	-P 2ybc
Moiety formula	C20 H22 N4 O3	C20 H22 N4 O3
Sum formula	C20 H22 N4 O3	C20 H22 N4 O3
Mr	366.42	366.41
Dx,g cm-3	1.248	1.248
Z	4	4
Mu (mm-1)	0.701	0.701
F000	776.0	776.0
F000’	778.38	
h,k,lmax	14,18,14	14,18,14
Nref	3849	3836
Tmin,Tmax	0.817,0.919	0.786,0.915
Tmin’	0.777	

Correction method= # Reported T Limits: Tmin=0.786 Tmax=0.915
AbsCorr = MULTI-SCAN

Data completeness= 0.997 Theta(max)= 72.202

R(reflections)= 0.0437(3513) wR2(reflections)= 0.1301(3836)

S = 1.026 Npar= 318

The following ALERTS were generated. Each ALERT has the format
test-name_ALERT_alert-type_alert-level.
Click on the hyperlinks for more details of the test.

Alert level C

ABSTY02_ALERT_1_C An _exptl_absorpt_correction_type has been given without
a literature citation. This should be contained in the
_exptl_absorpt_process_details field.
Absorption correction given as multi-scan
PLAT250_ALERT_2_C Large U3/U1 Ratio for Average U(i,j) Tensor .... 2.4 Note
PLAT911_ALERT_3_C Missing FCF Refl Between Thmin & STh/L= 0.600 12 Report

---

Alert level G

PLAT002_ALERT_2_G Number of Distance or Angle Restraints on AtSite 21 Note
PLAT003_ALERT_2_G Number of Uiso or Uij Restrained non-H Atoms ... 34 Report
PLAT172_ALERT_4_G The CIF-Embedded .res File Contains DFIX Records 1 Report
PLAT175_ALERT_4_G The CIF-Embedded .res File Contains SAME Records 4 Report
PLAT178_ALERT_4_G The CIF-Embedded .res File Contains SIMU Records 2 Report
PLAT187_ALERT_4_G The CIF-Embedded .res File Contains RIGU Records 1 Report
PLAT300_ALERT_4_G Atom Site Occupancy of O64 Constrained at 0.5 Check

```
PLAT300_ALERT_4_G Atom Site Occupancy of O64A       Constrained at        0.5 Check
PLAT300_ALERT_4_G Atom Site Occupancy of C63        Constrained at        0.5 Check
PLAT300_ALERT_4_G Atom Site Occupancy of C63A       Constrained at        0.5 Check
PLAT300_ALERT_4_G Atom Site Occupancy of C64        Constrained at        0.5 Check
PLAT300_ALERT_4_G Atom Site Occupancy of C64A       Constrained at        0.5 Check
PLAT300_ALERT_4_G Atom Site Occupancy of C65        Constrained at        0.5 Check
PLAT300_ALERT_4_G Atom Site Occupancy of C65A       Constrained at        0.5 Check
PLAT300_ALERT_4_G Atom Site Occupancy of C66        Constrained at        0.5 Check
PLAT300_ALERT_4_G Atom Site Occupancy of C66A       Constrained at        0.5 Check
PLAT300_ALERT_4_G Atom Site Occupancy of C67        Constrained at        0.5 Check
PLAT300_ALERT_4_G Atom Site Occupancy of C67A       Constrained at        0.5 Check
PLAT300_ALERT_4_G Atom Site Occupancy of C68        Constrained at        0.5 Check
PLAT300_ALERT_4_G Atom Site Occupancy of C68A       Constrained at        0.5 Check
PLAT300_ALERT_4_G Atom Site Occupancy of H63        Constrained at        0.5 Check
PLAT300_ALERT_4_G Atom Site Occupancy of H63A       Constrained at        0.5 Check
PLAT300_ALERT_4_G Atom Site Occupancy of H65        Constrained at        0.5 Check
PLAT300_ALERT_4_G Atom Site Occupancy of H65A       Constrained at        0.5 Check
PLAT300_ALERT_4_G Atom Site Occupancy of H66        Constrained at        0.5 Check
PLAT300_ALERT_4_G Atom Site Occupancy of H66A       Constrained at        0.5 Check
PLAT300_ALERT_4_G Atom Site Occupancy of H67        Constrained at        0.5 Check
PLAT300_ALERT_4_G Atom Site Occupancy of H67A       Constrained at        0.5 Check
PLAT300_ALERT_4_G Atom Site Occupancy of H68A       Constrained at        0.5 Check
PLAT300_ALERT_4_G Atom Site Occupancy of H68B       Constrained at        0.5 Check
PLAT300_ALERT_4_G Atom Site Occupancy of H68C       Constrained at        0.5 Check
PLAT300_ALERT_4_G Atom Site Occupancy of H68D       Constrained at        0.5 Check
PLAT300_ALERT_4_G Atom Site Occupancy of H68E       Constrained at        0.5 Check
PLAT300_ALERT_4_G Atom Site Occupancy of H68F       Constrained at        0.5 Check
PLAT301_ALERT_3_G Main Residue  Disorder ..............(Resd  1  )        26% Note
PLAT414_ALERT_2_G Short Intra D-H..H-X       H1       ..H63A             2.12 Ang.
                                                      x,y,z  =      1_555 Check
PLAT811_ALERT_5_G No ADDSYM Analysis: Too Many Excluded Atoms ....          ! Info
PLAT860_ALERT_3_G Number of Least-Squares Restraints .............        646 Note
PLAT912_ALERT_4_G Missing # of FCF Reflections Above STh/L=  0.600          1 Note
PLAT913_ALERT_3_G Missing # of Very Strong Reflections in FCF ....          2 Note
PLAT933_ALERT_2_G Number of OMIT Records in Embedded .res File ...          5 Note
PLAT978_ALERT_2_G Number C-C Bonds with Positive Residual Density.          6 Info
PLAT992_ALERT_5_G Repd & Actual _reflns_number_gt Values Differ by          4 Check
```

---

0 **ALERT level A** = Most likely a serious problem - resolve or explain
0 **ALERT level B** = A potentially serious problem, consider carefully
3 **ALERT level C** = Check. Ensure it is not caused by an omission or oversight
43 **ALERT level G** = General information/check it is not something unexpected

1 ALERT type 1 CIF construction/syntax error, inconsistent or missing data
6 ALERT type 2 Indicator that the structure model may be wrong or deficient
4 ALERT type 3 Indicator that the structure quality may be low
33 ALERT type 4 Improvement, methodology, query or suggestion
2 ALERT type 5 Informative message, check

Datablock: sb1336_hy_CCDC-2076718

Bond precision: C-C = 0.0015 A   Wavelength=1.54178

Cell:	a=11.2851(4)	b=15.2971(5)	c=11.6703(4)
	alpha=90	beta=99.474(1)	gamma=90
Temperature:	123 K		

	Calculated	Reported
Volume	1987.16(12)	1987.16(12)
Space group	P 21/c	P 21/c
Hall group	-P 2ybc	-P 2ybc
Moiety formula	C21 H24 N4 O4	C21 H24 N4 O4
Sum formula	C21 H24 N4 O4	C21 H24 N4 O4
Mr	396.44	396.44
Dx,g cm-3	1.325	1.325
Z	4	4
Mu (mm-1)	0.767	0.767
F000	840.0	840.0
F000'	842.64	
h,k,lmax	13,18,14	13,18,14
Nref	3932	3924
Tmin,Tmax	0.912,0.955	0.879,0.958
Tmin'	0.898	

Correction method= # Reported T Limits: Tmin=0.879 Tmax=0.958
AbsCorr = MULTI-SCAN

Data completeness= 0.998 Theta(max)= 72.284

R(reflections)= 0.0323(3736) wR2(reflections)= 0.0853(3924)

S = 1.030 Npar= 274

The following ALERTS were generated. Each ALERT has the format
test-name_ALERT_alert-type_alert-level.
Click on the hyperlinks for more details of the test.

Alert level C

PLAT911_ALERT_3_C Missing FCF Refl Between Thmin & STh/L= 0.600 4 Report

---

Alert level G

PLAT002_ALERT_2_G Number of Distance or Angle Restraints on AtSite 6 Note
PLAT172_ALERT_4_G The CIF-Embedded .res File Contains DFIX Records 1 Report
PLAT860_ALERT_3_G Number of Least-Squares Restraints .... 3 Note
PLAT912_ALERT_4_G Missing # of FCF Reflections Above STh/L= 0.600 5 Note
PLAT913_ALERT_3_G Missing # of Very Strong Reflections in FCF .... 1 Note
PLAT978_ALERT_2_G Number C-C Bonds with Positive Residual Density. 19 Info

---

0 **ALERT level A** = Most likely a serious problem - resolve or explain
0 **ALERT level B** = A potentially serious problem, consider carefully
1 **ALERT level C** = Check. Ensure it is not caused by an omission or oversight
6 **ALERT level G** = General information/check it is not something unexpected

0 ALERT type 1 CIF construction/syntax error, inconsistent or missing data

```
2 ALERT type 2 Indicator that the structure model may be wrong or deficient
3 ALERT type 3 Indicator that the structure quality may be low
2 ALERT type 4 Improvement, methodology, query or suggestion
0 ALERT type 5 Informative message, check
```

It is advisable to attempt to resolve as many as possible of the alerts in all categories. Often the minor alerts point to easily fixed oversights, errors and omissions in your CIF or refinement strategy, so attention to these fine details can be worthwhile. In order to resolve some of the more serious problems it may be necessary to carry out additional measurements or structure refinements. However, the purpose of your study may justify the reported deviations and the more serious of these should normally be commented upon in the discussion or experimental section of a paper or in the "special_details" fields of the CIF. checkCIF was carefully designed to identify outliers and unusual parameters, but every test has its limitations and alerts that are not important in a particular case may appear. Conversely, the absence of alerts does not guarantee there are no aspects of the results needing attention. It is up to the individual to critically assess their own results and, if necessary, seek expert advice.

Publication of your CIF in IUCr journals

A basic structural check has been run on your CIF. These basic checks will be run on all CIFs submitted for publication in IUCr journals (*Acta Crystallographica*, *Journal of Applied Crystallography*, *Journal of Synchrotron Radiation*); however, if you intend to submit to *Acta Crystallographica Section C* or *E* or *IUCrData*, you should make sure that full publication checks are run on the final version of your CIF prior to submission.

Publication of your CIF in other journals

Please refer to the *Notes for Authors* of the relevant journal for any special instructions relating to CIF submission.

PLATON version of 22/03/2021; check.def file version of 19/03/2021

Datablock sb1177_hy_CCDC-2076713 - ellipsoid plot

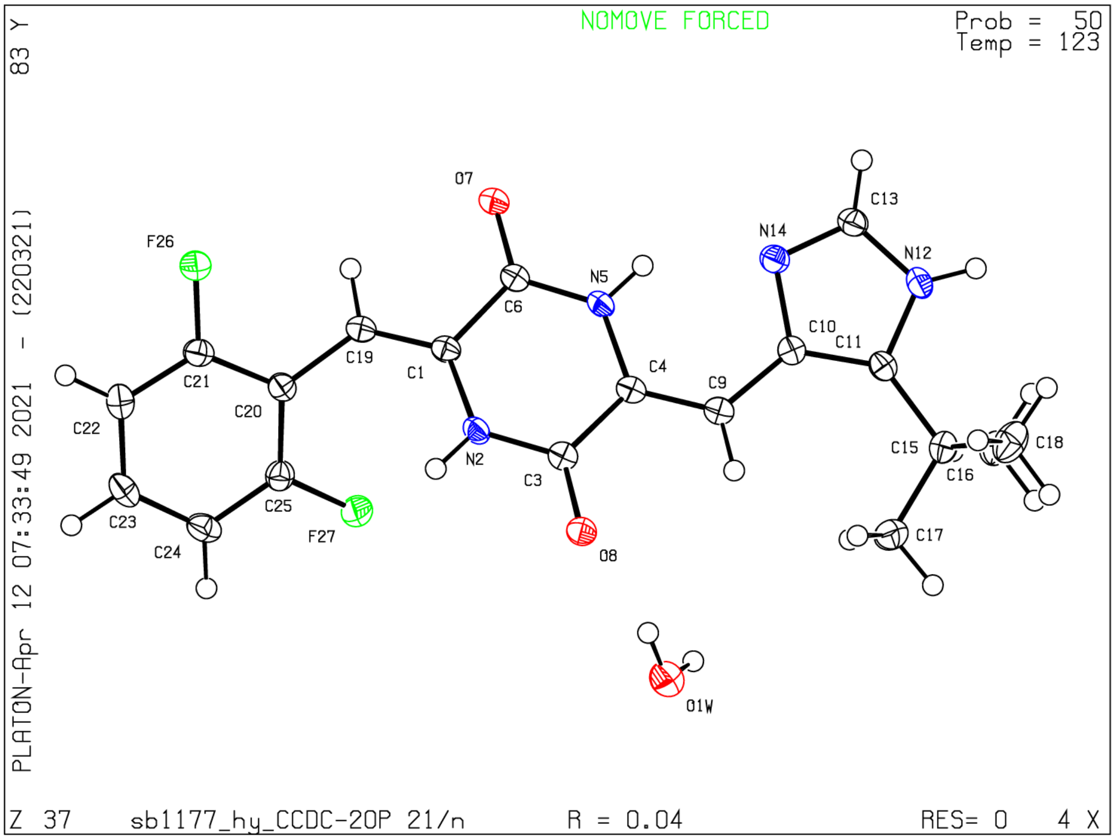

Datablock sb1192_hy_CCDC-2076714 - ellipsoid plot

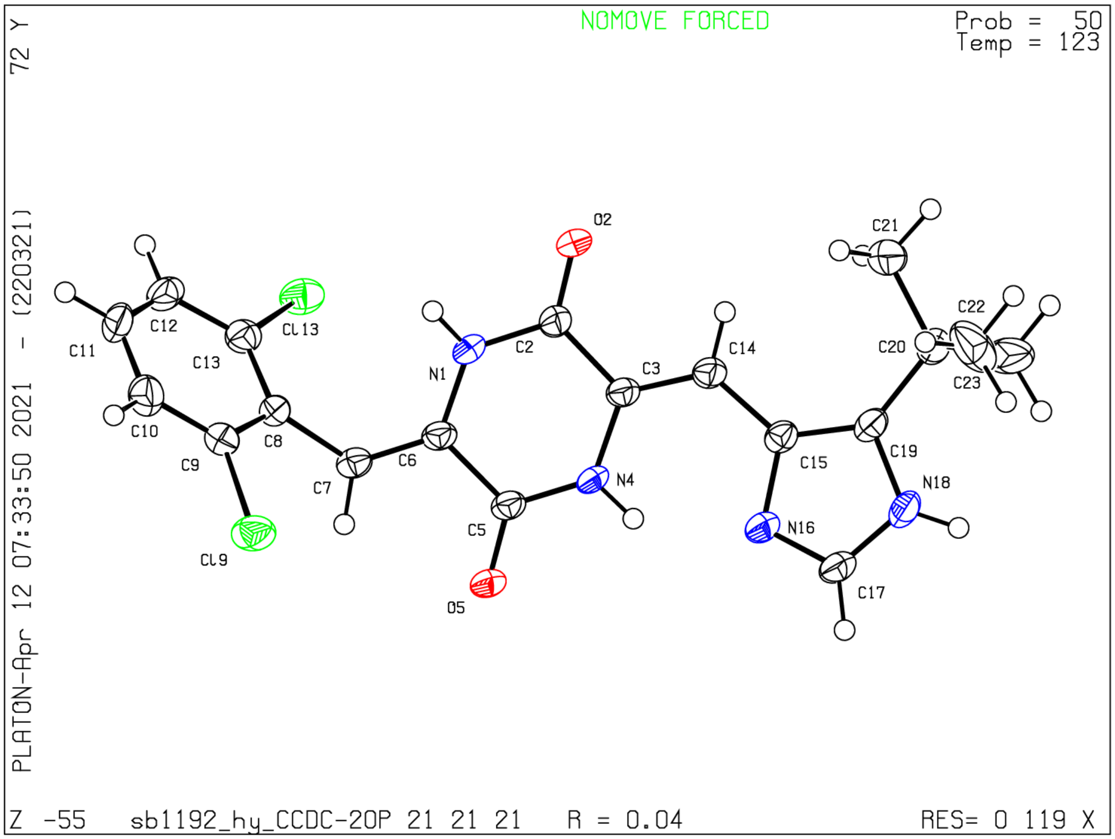

Datablock sb1354_hy_CCDC-2076715 - ellipsoid plot

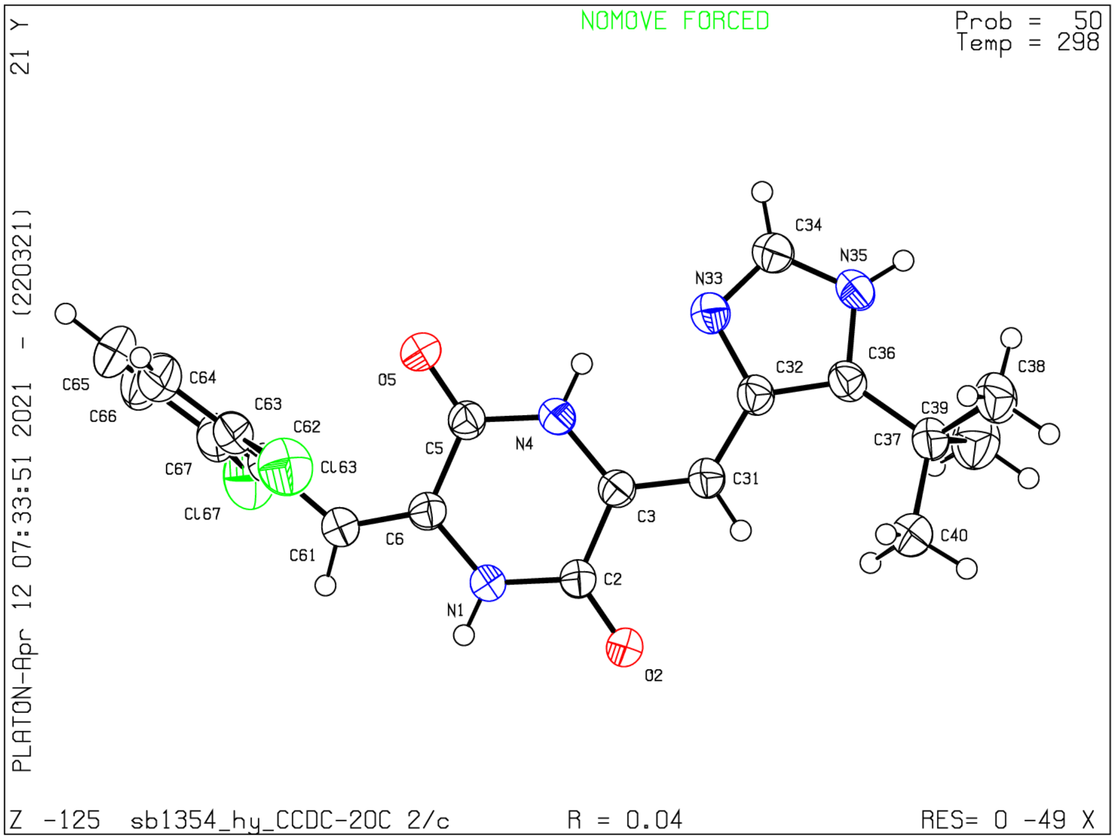

Datablock sb1373_hy_CCDC-2076716 - ellipsoid plot

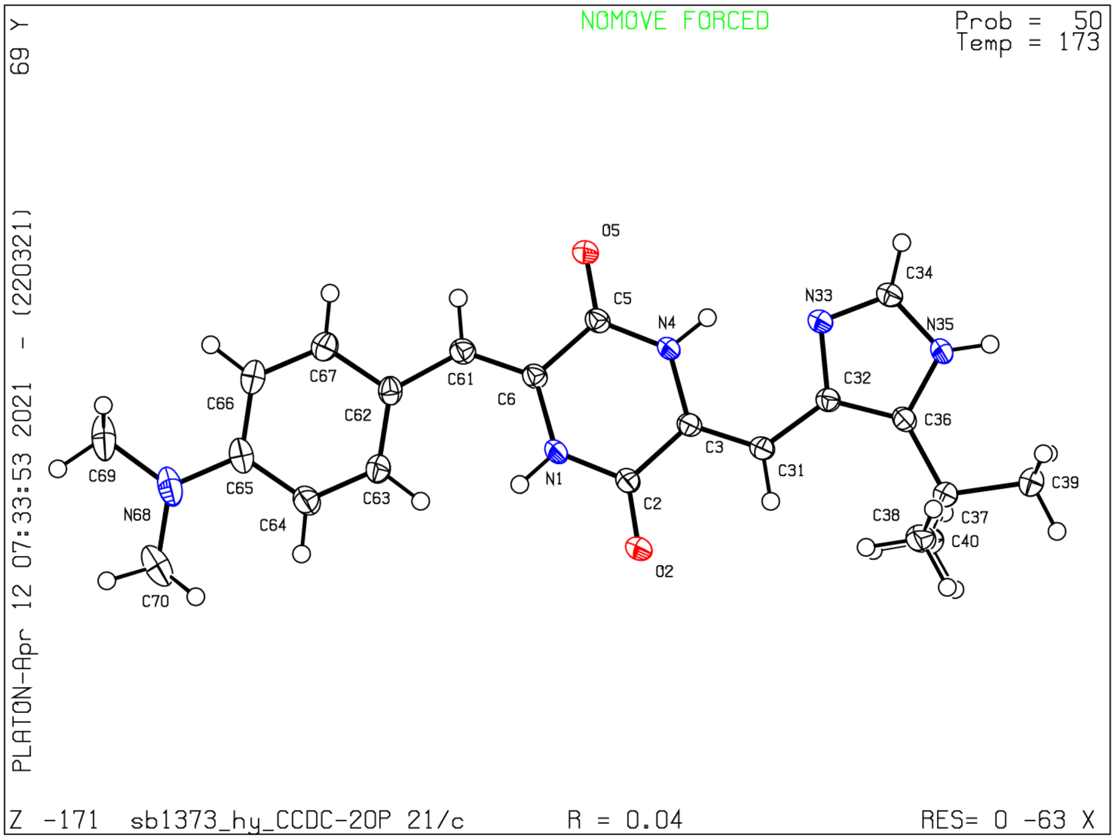

Datablock sb1342_hy_CCDC-2076717 - ellipsoid plot

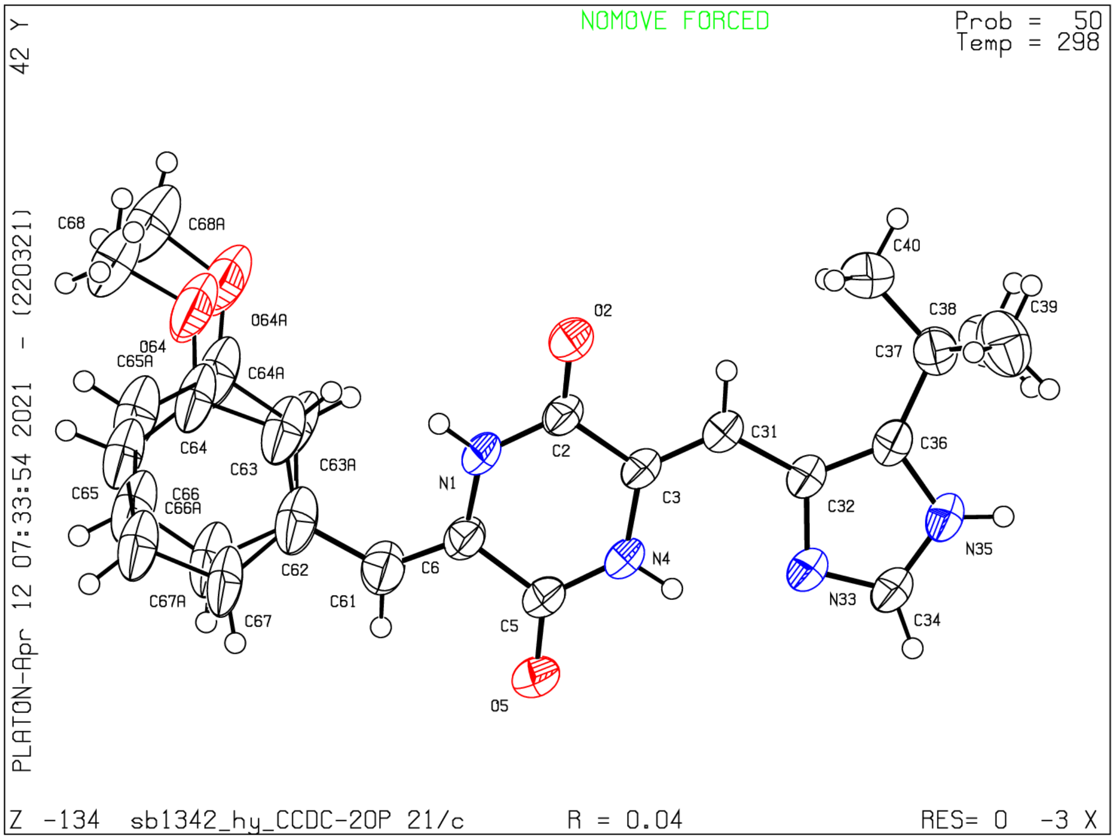

Datablock sb1336_hy_CCDC-2076718 - ellipsoid plot

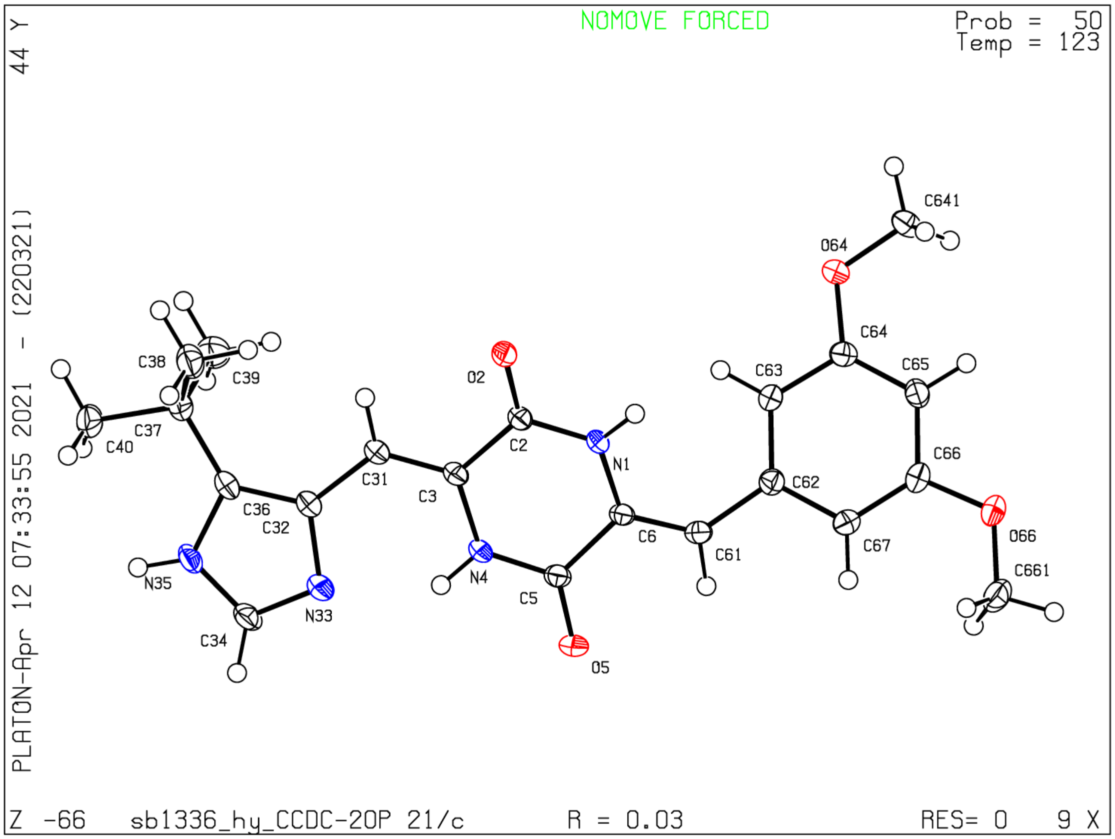

Supplement: Supplementary file 3 — Supplementary Data 1 [file 41467_2022_33750_MOESM3_ESM.pdf]
