# Supplementary material for: Hemipiperazines as peptide-derived molecular photoswitches with low-nanomolar cytotoxicity
Source: Nat Commun. 2022 Oct 14;13:6066. doi: 10.1038/s41467-022-33750-7 (PMC9568564; doi:10.1038/s41467-022-33750-7)

checkCIF/PLATON report

Structure factors have been supplied for datablock(s) sb1368_tw_CCDC-2177725, sb1376_hy_CCDC-2177724, sb1393_hy_CCDC-2177722, sb1440_hy_CCDC-2177723, suk130_CCDC-2177721, suk170_CCDC-2177720

THIS REPORT IS FOR GUIDANCE ONLY. IF USED AS PART OF A REVIEW PROCEDURE FOR PUBLICATION, IT SHOULD NOT REPLACE THE EXPERTISE OF AN EXPERIENCED CRYSTALLOGRAPHIC REFEREE.

No syntax errors found. CIF dictionary Interpreting this report

Datablock: suk170_CCDC-2177720

```
Bond precision:   C-C = 0.0107 A          Wavelength=1.54178

Cell:              a=7.2931(5)     b=23.1044(16)      c=13.5985(9)
                   alpha=90        beta=90.424(4)     gamma=90
Temperature:       298 K

                 Calculated                        Reported
Volume           2291.3(3)                         2291.3(3)
Space group      P 21                              P 21
Hall group       P 2yb                             P 2yb
Moiety formula   C13 H12 N2 O3                     C13 H12 N2 O3
Sum formula      C13 H12 N2 O3                     C13 H12 N2 O3
Mr               244.25                            244.25
Dx,g cm-3        1.416                             1.416
Z                8                                 8
Mu (mm-1)        0.850                             0.850
F000             1024.0                            1024.0
F000'            1027.37
h,k,lmax         9,28,16                           9,28,16
Nref             9134[ 4685]                       8936
Tmin,Tmax        0.832,0.950                       0.614,0.925
Tmin'            0.736

Correction method= # Reported T Limits: Tmin=0.614 Tmax=0.925
AbsCorr = MULTI-SCAN

Data completeness= 1.91/0.98       Theta(max)= 73.057
```

R(reflections)= 0.0602(8174) wR2(reflections)= 0.1650(8936)

S = 1.057 Npar= 654

The following ALERTS were generated. Each ALERT has the format
test-name_ALERT_alert-type_alert-level.
Click on the hyperlinks for more details of the test.

Alert level B

PLAT340_ALERT_3_B Low Bond Precision on C-C Bonds .... 0.01066 Ang.

Author Response: due to the bad quality of the data (twinning) due to the bad quality of the data (twinning) due to the bad quality of the data (twinning)

Alert level C

PLAT089_ALERT_3_C Poor Data / Parameter Ratio (Zmax < 18) .... 7.05 Note
PLAT230_ALERT_2_C Hirshfeld Test Diff for N61 --C67 . 6.3 s.u.
PLAT242_ALERT_2_C Low 'MainMol' Ueq as Compared to Neighbors of C7 Check
PLAT242_ALERT_2_C Low 'MainMol' Ueq as Compared to Neighbors of C27 Check
PLAT911_ALERT_3_C Missing FCF Refl Between Thmin & STh/L= 0.600 21 Report

---

Alert level G

PLAT003_ALERT_2_G Number of Uiso or Uij Restrained non-H Atoms ... 72 Report
PLAT007_ALERT_5_G Number of Unrefined Donor-H Atoms .... 4 Report
PLAT187_ALERT_4_G The CIF-Embedded .res File Contains RIGU Records 1 Report
PLAT860_ALERT_3_G Number of Least-Squares Restraints .... 541 Note
PLAT870_ALERT_4_G ALERTS Related to Twinning Effects Suppressed .. ! Info
PLAT912_ALERT_4_G Missing # of FCF Reflections Above STh/L= 0.600 52 Note
PLAT916_ALERT_2_G Hooft y and Flack x Parameter Values Differ by . 0.14 Check
PLAT931_ALERT_5_G CIFcalcFCF Twin Law (1 0 0) Est.d BASF 0.15 Check
PLAT933_ALERT_2_G Number of HKL-OMIT Records in Embedded .res File 15 Note
PLAT992_ALERT_5_G Repd & Actual _reflns_number_gt Values Differ by 6 Check

---

0 **ALERT level A** = Most likely a serious problem - resolve or explain
1 **ALERT level B** = A potentially serious problem, consider carefully
5 **ALERT level C** = Check. Ensure it is not caused by an omission or oversight
10 **ALERT level G** = General information/check it is not something unexpected

0 ALERT type 1 CIF construction/syntax error, inconsistent or missing data
6 ALERT type 2 Indicator that the structure model may be wrong or deficient
4 ALERT type 3 Indicator that the structure quality may be low
3 ALERT type 4 Improvement, methodology, query or suggestion
3 ALERT type 5 Informative message, check

Datablock: suk130_CCDC-2177721

Bond precision: C-C = 0.0046 A Wavelength=1.54178

Cell:	a=7.4710(17)	b=8.0460(17)	c=12.747(3)
	alpha=80.540(15)	beta=74.438(15)	gamma=86.632(15)
Temperature:	298 K		

	Calculated	Reported
Volume	728.0(3)	728.0(3)
Space group	P -1	P -1
Hall group	-P 1	-P 1
Moiety formula	C14 H11 N3 O3, 2(H2 O)	C14 H11 N3 O3, 2(H2 O)
Sum formula	C14 H15 N3 O5	C14 H15 N3 O5
Mr	305.29	305.29
Dx,g cm-3	1.393	1.393
Z	2	2
Mu (mm-1)	0.909	0.909
F000	320.0	320.0
F000'	321.12	
h,k,lmax	9,9,15	9,9,15
Nref	2878	2736
Tmin,Tmax	0.916,0.964	0.723,0.970
Tmin'	0.865	

Correction method= # Reported T Limits: Tmin=0.723 Tmax=0.970
AbsCorr = MULTI-SCAN

Data completeness= 0.951 Theta(max)= 72.374

R(reflections)= 0.0771(2100) wR2(reflections)= 0.2210(2736)

S = 1.083 Npar= 212

The following ALERTS were generated. Each ALERT has the format
test-name_ALERT_alert-type_alert-level.
Click on the hyperlinks for more details of the test.

Alert level C

PLAT029_ALERT_3_C	_diffrn_measured_fraction_theta_full value Low .	0.969	Why?
PLAT340_ALERT_3_C	Low Bond Precision on C-C Bonds	0.00458	Ang.
PLAT906_ALERT_3_C	Large K Value in the Analysis of Variance	14.656	Check
PLAT911_ALERT_3_C	Missing FCF Refl Between Thmin & STh/L= 0.600	81	Report

Alert level G

PLAT002_ALERT_2_G	Number of Distance or Angle Restraints on AtSite	6	Note

```
PLAT007_ALERT_5_G Number of Unrefined Donor-H Atoms .............          1 Report
PLAT154_ALERT_1_G The s.u.'s on the Cell Angles are Equal ..(Note)      0.015 Degree
PLAT172_ALERT_4_G The CIF-Embedded .res File Contains DFIX Records          2 Report
PLAT180_ALERT_4_G Check Cell Rounding: # of Values Ending with 0 =          3 Note
PLAT720_ALERT_4_G Number of Unusual/Non-Standard Labels ..........          4 Note
PLAT860_ALERT_3_G Number of Least-Squares Restraints .............          6 Note
PLAT912_ALERT_4_G Missing # of FCF Reflections Above STh/L=  0.600         61 Note
PLAT913_ALERT_3_G Missing # of Very Strong Reflections in FCF ....          1 Note
PLAT933_ALERT_2_G Number of HKL-OMIT Records in Embedded .res File         18 Note
PLAT941_ALERT_3_G Average HKL Measurement Multiplicity ...........        1.7 Low
PLAT978_ALERT_2_G Number C-C Bonds with Positive Residual Density.          1 Info
```

---

   0 **ALERT level A** = Most likely a serious problem - resolve or explain
   0 **ALERT level B** = A potentially serious problem, consider carefully
   4 **ALERT level C** = Check. Ensure it is not caused by an omission or oversight
  12 **ALERT level G** = General information/check it is not something unexpected

   1 ALERT type 1 CIF construction/syntax error, inconsistent or missing data
   3 ALERT type 2 Indicator that the structure model may be wrong or deficient
   7 ALERT type 3 Indicator that the structure quality may be low
   4 ALERT type 4 Improvement, methodology, query or suggestion
   1 ALERT type 5 Informative message, check

Datablock: sb1393_hy_CCDC-2177722

Bond precision: C-C = 0.0017 A Wavelength=1.54178

Cell:	a=11.9836(3)	b=7.9926(2)	c=15.3272(4)
	alpha=90	beta=104.010(1)	gamma=90
Temperature:	173 K		

	Calculated	Reported
Volume	1424.37(6)	1424.37(6)
Space group	P 21/n	P 21/n
Hall group	-P 2yn	-P 2yn
Moiety formula	C15 H17 N3 O3	C15 H17 N3 O3
Sum formula	C15 H17 N3 O3	C15 H17 N3 O3
Mr	287.32	287.32
Dx,g cm-3	1.340	1.340
Z	4	4
Mu (mm-1)	0.784	0.784
F000	608.0	608.0
F000'	609.93	
h,k,lmax	14,9,18	14,9,18
Nref	2806	2789
Tmin,Tmax	0.828,0.954	0.790,0.958
Tmin'	0.828	

```
Correction method= # Reported T Limits: Tmin=0.790 Tmax=0.958
AbsCorr = MULTI-SCAN

Data completeness= 0.994          Theta(max)= 72.289

                                                    wR2(reflections)=
R(reflections)= 0.0368( 2644)                       0.1023( 2789)

S = 1.048                   Npar= 196
```

The following ALERTS were generated. Each ALERT has the format
test-name_ALERT_alert-type_alert-level.
Click on the hyperlinks for more details of the test.

Alert level C

```
PLAT220_ALERT_2_C NonSolvent   Resd 1  C   Ueq(max)/Ueq(min) Range        3.2 Ratio
PLAT911_ALERT_3_C Missing FCF Refl Between Thmin & STh/L=    0.600         14 Report
PLAT913_ALERT_3_C Missing # of Very Strong Reflections in FCF ....          4 Note
```

Alert level G

```
PLAT002_ALERT_2_G Number of Distance or Angle Restraints on AtSite          2 Note
PLAT172_ALERT_4_G The CIF-Embedded .res File Contains DFIX Records          1 Report
PLAT432_ALERT_2_G Short Inter X...Y Contact  O2       ..C11      .       3.01 Ang.
                                       3/2-x,-1/2+y,3/2-z  =      2_646 Check
PLAT432_ALERT_2_G Short Inter X...Y Contact  O5       ..C37      .       3.01 Ang.
                                              1-x,1-y,1-z  =      3_666 Check
PLAT860_ALERT_3_G Number of Least-Squares Restraints ............           1 Note
PLAT910_ALERT_3_G Missing # of FCF Reflection(s) Below Theta(Min).          1 Note
PLAT912_ALERT_4_G Missing # of FCF Reflections Above STh/L=  0.600          3 Note
PLAT933_ALERT_2_G Number of HKL-OMIT Records in Embedded .res File          1 Note
PLAT941_ALERT_3_G Average HKL Measurement Multiplicity ..........         4.8 Low
PLAT978_ALERT_2_G Number C-C Bonds with Positive Residual Density.         12 Info
```

---

   0 **ALERT level A** = Most likely a serious problem - resolve or explain
   0 **ALERT level B** = A potentially serious problem, consider carefully
   3 **ALERT level C** = Check. Ensure it is not caused by an omission or oversight
  10 **ALERT level G** = General information/check it is not something unexpected

   0 ALERT type 1 CIF construction/syntax error, inconsistent or missing data
   6 ALERT type 2 Indicator that the structure model may be wrong or deficient
   5 ALERT type 3 Indicator that the structure quality may be low
   2 ALERT type 4 Improvement, methodology, query or suggestion
   0 ALERT type 5 Informative message, check

Datablock: sb1440_hy_CCDC-2177723

```
Bond precision:   C-C = 0.0019 A          Wavelength=1.54178
```

Cell:	a=6.6984(2)	b=7.3720(2)	c=13.9541(3)
	alpha=82.063(1)	beta=89.009(1)	gamma=74.886(1)
Temperature:	298 K		

	Calculated	Reported
Volume	658.73(3)	658.73(3)
Space group	P -1	P -1
Hall group	-P 1	-P 1
Moiety formula	C14 H14 N2 O4	C14 H14 N2 O4
Sum formula	C14 H14 N2 O4	C14 H14 N2 O4
Mr	274.27	274.27
Dx,g cm-3	1.383	1.383
Z	2	2
Mu (mm-1)	0.859	0.859
F000	288.0	288.0
F000'	288.98	
h,k,lmax	8,9,17	8,9,17
Nref	2596	2584
Tmin,Tmax	0.921,0.966	0.888,0.970
Tmin'	0.872	

Correction method= # Reported T Limits: Tmin=0.888 Tmax=0.970
AbsCorr = MULTI-SCAN

Data completeness= 0.995 Theta(max)= 72.305

R(reflections)= 0.0381(2336) wR2(reflections)= 0.1103(2584)

S = 1.076 Npar= 186

The following ALERTS were generated. Each ALERT has the format
test-name_ALERT_alert-type_alert-level.
Click on the hyperlinks for more details of the test.

Alert level C

PLAT911_ALERT_3_C Missing FCF Refl Between Thmin & STh/L= 0.600 4 Report

---

Alert level G

PLAT002_ALERT_2_G Number of Distance or Angle Restraints on AtSite 2 Note
PLAT154_ALERT_1_G The s.u.'s on the Cell Angles are Equal ..(Note) 0.001 Degree
PLAT172_ALERT_4_G The CIF-Embedded .res File Contains DFIX Records 1 Report
PLAT860_ALERT_3_G Number of Least-Squares Restraints .... 1 Note
PLAT912_ALERT_4_G Missing # of FCF Reflections Above STh/L= 0.600 8 Note
PLAT913_ALERT_3_G Missing # of Very Strong Reflections in FCF .... 2 Note
PLAT933_ALERT_2_G Number of HKL-OMIT Records in Embedded .res File 1 Note

```
PLAT941_ALERT_3_G Average HKL Measurement Multiplicity ...........        3.8 Low
PLAT978_ALERT_2_G Number C-C Bonds with Positive Residual Density.          9 Info
PLAT992_ALERT_5_G Repd & Actual _reflns_number_gt Values Differ by          5 Check
```

```
   0 ALERT level A = Most likely a serious problem - resolve or explain
   0 ALERT level B = A potentially serious problem, consider carefully
   1 ALERT level C = Check. Ensure it is not caused by an omission or oversight
  10 ALERT level G = General information/check it is not something unexpected

   1 ALERT type 1 CIF construction/syntax error, inconsistent or missing data
   3 ALERT type 2 Indicator that the structure model may be wrong or deficient
   4 ALERT type 3 Indicator that the structure quality may be low
   2 ALERT type 4 Improvement, methodology, query or suggestion
   1 ALERT type 5 Informative message, check
```

Datablock: sb1376_hy_CCDC-2177724

Bond precision: C-C = 0.0022 A Wavelength=1.54178

Cell:	a=15.4147(2)	b=6.1889(1)	c=23.7010(3)
	alpha=90	beta=90	gamma=90
Temperature:	298 K		

	Calculated	Reported
Volume	2261.08(6)	2261.08(5)
Space group	P b c a	P b c a
Hall group	-P 2ac 2ab	-P 2ac 2ab
Moiety formula	C13 H10 N2 O3	C13 H10 N2 O3
Sum formula	C13 H10 N2 O3	C13 H10 N2 O3
Mr	242.23	242.23
Dx,g cm-3	1.423	1.423
Z	8	8
Mu (mm-1)	0.861	0.861
F000	1008.0	1008.0
F000’	1011.37	
h,k,lmax	19,7,29	19,7,29
Nref	2225	2223
Tmin,Tmax	0.883,0.983	0.849,0.970
Tmin’	0.856	

Correction method= # Reported T Limits: Tmin=0.849 Tmax=0.970
AbsCorr = MULTI-SCAN

Data completeness= 0.999 Theta(max)= 72.096

R(reflections)= 0.0384(1986) wR2(reflections)= 0.1034(2223)

S = 1.049 Npar= 164

The following ALERTS were generated. Each ALERT has the format
test-name_ALERT_alert-type_alert-level.
Click on the hyperlinks for more details of the test.

Alert level C

```
PLAT242_ALERT_2_C Low    'MainMol' Ueq as Compared to Neighbors of        C11 Check
PLAT906_ALERT_3_C Large K Value in the Analysis of Variance ......      2.709 Check
```

Alert level G

```
PLAT432_ALERT_2_G Short Inter X...Y Contact  O4       ..C4       .       2.90 Ang.
                                           1-x,1/2+y,1/2-z  =      3_655 Check
PLAT432_ALERT_2_G Short Inter X...Y Contact  O4       ..C3       .       3.00 Ang.
                                           1-x,1/2+y,1/2-z  =      3_655 Check
PLAT910_ALERT_3_G Missing # of FCF Reflection(s) Below Theta(Min).          1 Note
PLAT912_ALERT_4_G Missing # of FCF Reflections Above STh/L=  0.600          1 Note
PLAT913_ALERT_3_G Missing # of Very Strong Reflections in FCF ....          1 Note
PLAT953_ALERT_1_G Reported (CIF) and Actual (FCF) Hmax Differ by .          1 Units
PLAT955_ALERT_1_G Reported (CIF) and Actual (FCF) Lmax Differ by .          1 Units
PLAT978_ALERT_2_G Number C-C Bonds with Positive Residual Density.          3 Info
```

---

0 **ALERT level A** = Most likely a serious problem - resolve or explain
0 **ALERT level B** = A potentially serious problem, consider carefully
2 **ALERT level C** = Check. Ensure it is not caused by an omission or oversight
8 **ALERT level G** = General information/check it is not something unexpected

2 ALERT type 1 CIF construction/syntax error, inconsistent or missing data
4 ALERT type 2 Indicator that the structure model may be wrong or deficient
3 ALERT type 3 Indicator that the structure quality may be low
1 ALERT type 4 Improvement, methodology, query or suggestion
0 ALERT type 5 Informative message, check

Datablock: sb1368_tw_CCDC-2177725

Bond precision:	C-C = 0.0043 A		Wavelength=1.54178
Cell:	a=7.1588(3)	b=15.0290(6)	c=14.9109(6)
	alpha=90	beta=95.951(2)	gamma=90
Temperature:	173 K		

	Calculated	Reported
Volume	1595.61(11)	1595.61(11)
Space group	P 21/c	P 21/c
Hall group	-P 2ybc	-P 2ybc
Moiety formula	C19 H18 N4 O2	C19 H18 N4 O2
Sum formula	C19 H18 N4 O2	C19 H18 N4 O2
Mr	334.37	334.37
Dx,g cm-3	1.392	1.392
Z	4	4
Mu (mm-1)	0.757	0.757
F000	704.0	704.0
F000'	706.11	
h,k,lmax	8,18,18	8,18,18
Nref	3156	3119
Tmin,Tmax	0.964,0.985	0.784,0.970
Tmin'	0.873	

Correction method= # Reported T Limits: Tmin=0.784 Tmax=0.970
AbsCorr = MULTI-SCAN

Data completeness= 0.988 Theta(max)= 72.296

R(reflections)= 0.0676(2743) wR2(reflections)= 0.1629(3119)

S = 1.175 Npar= 233

The following ALERTS were generated. Each ALERT has the format
test-name_ALERT_alert-type_alert-level.
Click on the hyperlinks for more details of the test.

Alert level C

```
PLAT340_ALERT_3_C Low Bond Precision on  C-C Bonds ...............    0.00429 Ang.
PLAT906_ALERT_3_C Large K Value in the Analysis of Variance ......      7.904 Check
PLAT906_ALERT_3_C Large K Value in the Analysis of Variance ......      2.058 Check
PLAT911_ALERT_3_C Missing FCF Refl Between Thmin & STh/L=    0.600         14 Report
```

Alert level G

```
PLAT002_ALERT_2_G Number of Distance or Angle Restraints on AtSite          4 Note
PLAT172_ALERT_4_G The CIF-Embedded .res File Contains DFIX Records          1 Report
PLAT860_ALERT_3_G Number of Least-Squares Restraints .............          2 Note
PLAT870_ALERT_4_G ALERTS Related to Twinning Effects Suppressed ..          ! Info
PLAT912_ALERT_4_G Missing # of FCF Reflections Above STh/L=  0.600         23 Note
PLAT931_ALERT_5_G CIFcalcFCF  Twin Law ( 0 0 1)        Est.d BASF        0.21 Check
PLAT933_ALERT_2_G Number of HKL-OMIT Records in Embedded .res File          7 Note
PLAT941_ALERT_3_G Average HKL Measurement Multiplicity ..........         1.0 Low
```

```
0 ALERT level A = Most likely a serious problem - resolve or explain
0 ALERT level B = A potentially serious problem, consider carefully
4 ALERT level C = Check. Ensure it is not caused by an omission or oversight
8 ALERT level G = General information/check it is not something unexpected

0 ALERT type 1 CIF construction/syntax error, inconsistent or missing data
2 ALERT type 2 Indicator that the structure model may be wrong or deficient
6 ALERT type 3 Indicator that the structure quality may be low
3 ALERT type 4 Improvement, methodology, query or suggestion
1 ALERT type 5 Informative message, check
```

It is advisable to attempt to resolve as many as possible of the alerts in all categories. Often the minor alerts point to easily fixed oversights, errors and omissions in your CIF or refinement strategy, so attention to these fine details can be worthwhile. In order to resolve some of the more serious problems it may be necessary to carry out additional measurements or structure refinements. However, the purpose of your study may justify the reported deviations and the more serious of these should normally be commented upon in the discussion or experimental section of a paper or in the "special_details" fields of the CIF. checkCIF was carefully designed to identify outliers and unusual parameters, but every test has its limitations and alerts that are not important in a particular case may appear. Conversely, the absence of alerts does not guarantee there are no aspects of the results needing attention. It is up to the individual to critically assess their own results and, if necessary, seek expert advice.

Publication of your CIF in IUCr journals

A basic structural check has been run on your CIF. These basic checks will be run on all CIFs submitted for publication in IUCr journals (*Acta Crystallographica*, *Journal of Applied Crystallography*, *Journal of Synchrotron Radiation*); however, if you intend to submit to *Acta Crystallographica Section C* or *E* or *IUCrData*, you should make sure that full publication checks are run on the final version of your CIF prior to submission.

Publication of your CIF in other journals

Please refer to the *Notes for Authors* of the relevant journal for any special instructions relating to CIF submission.

PLATON version of 18/05/2022; check.def file version of 17/05/2022

Datablock suk170_CCDC-2177720 - ellipsoid plot

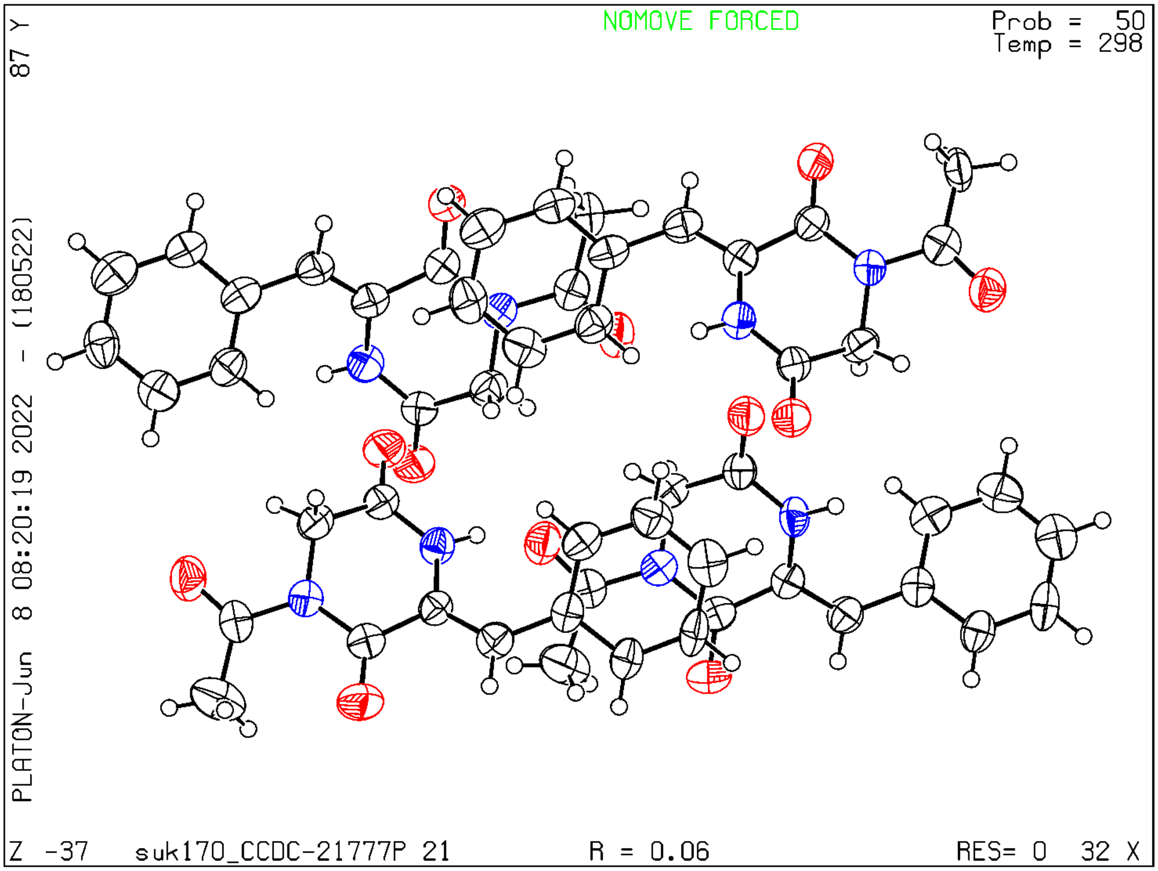

Datablock suk130_CCDC-2177721 - ellipsoid plot

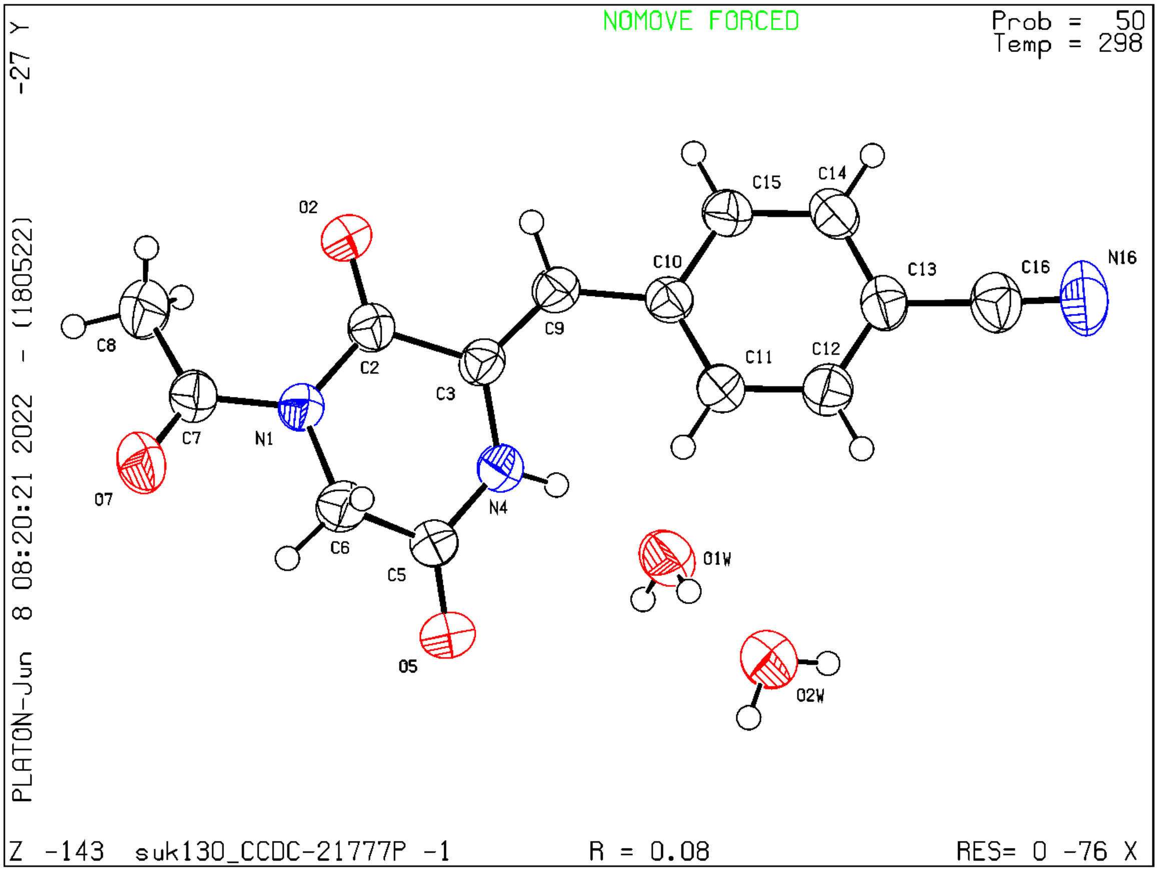

Datablock sb1393_hy_CCDC-2177722 - ellipsoid plot

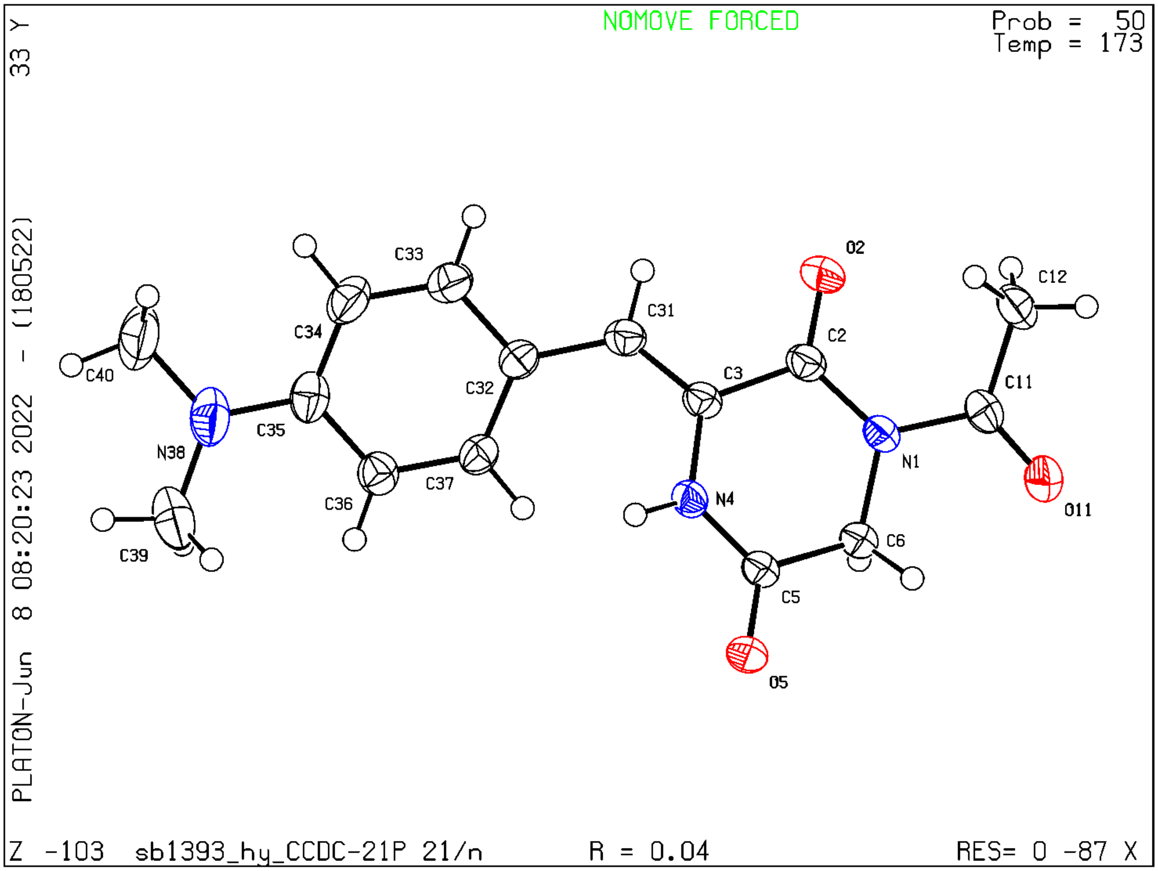

Datablock sb1440_hy_CCDC-2177723 - ellipsoid plot

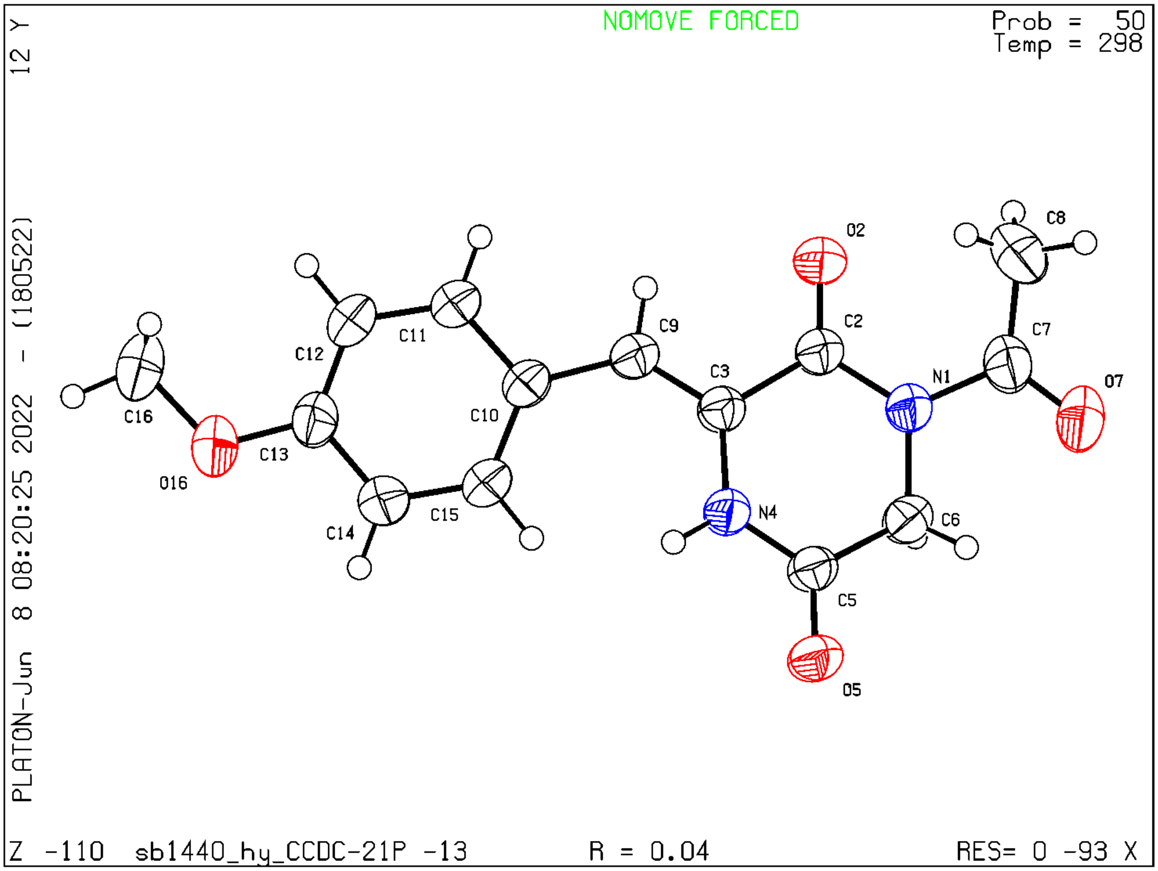

Datablock sb1376_hy_CCDC-2177724 - ellipsoid plot

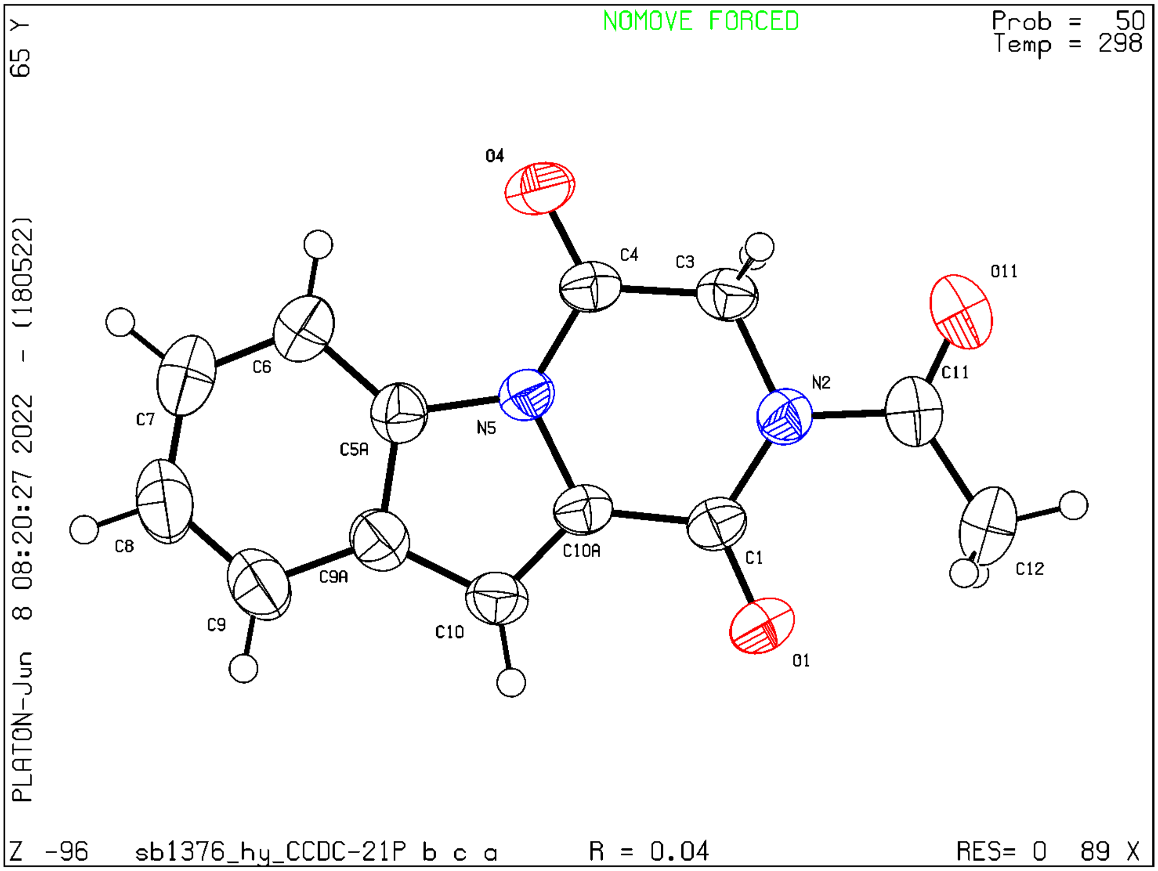

Datablock sb1368_tw_CCDC-2177725 - ellipsoid plot

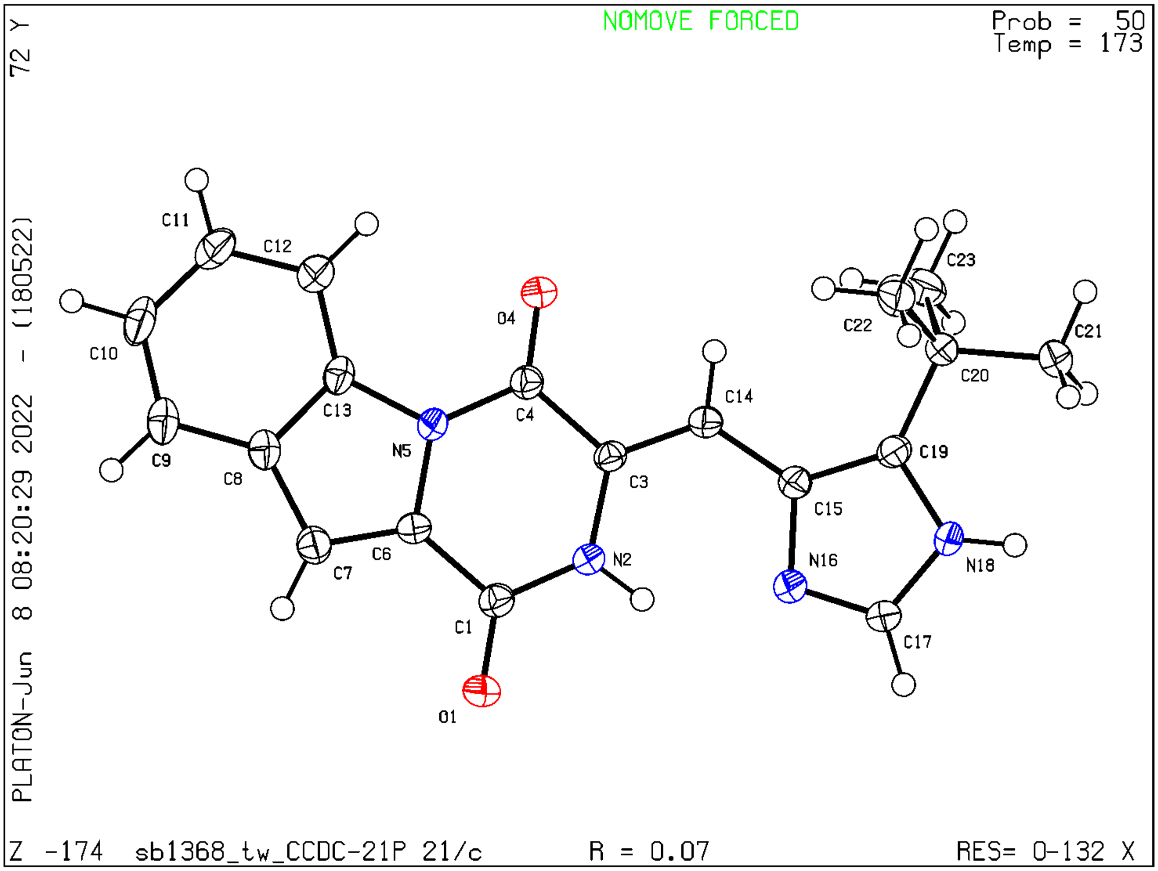

Supplement: Supplementary file 4 — Supplementary Data 2 [file 41467_2022_33750_MOESM4_ESM.pdf]
